# Annexin A1 contributes to pancreatic cancer cell phenotype, behaviour and metastatic potential independently of Formyl Peptide Receptor pathway

**DOI:** 10.1038/srep29660

**Published:** 2016-07-14

**Authors:** Raffaella Belvedere, Valentina Bizzarro, Giovanni Forte, Fabrizio Dal Piaz, Luca Parente, Antonello Petrella

**Affiliations:** 1 Department of Pharmacy, University of Salerno, via Giovanni Paolo II 132, 84084 Fisciano (SA) Italy

## Abstract

Annexin A1 (ANXA1) is a Ca^2+^-binding protein over-expressed in pancreatic cancer (PC). We recently reported that extracellular ANXA1 mediates PC cell motility acting on Formyl Peptide Receptors (FPRs). Here, we describe other mechanisms by which intracellular ANXA1 could mediate PC progression. We obtained ANXA1 Knock-Out (KO) MIA PaCa-2 cells using the CRISPR/Cas9 genome editing technology. LC-MS/MS analysis showed altered expression of several proteins involved in cytoskeletal organization. As a result, ANXA1 KO MIA PaCa-2 partially lost their migratory and invasive capabilities with a mechanism that appeared independent of FPRs. The acquisition of a less aggressive phenotype has been further investigated *in vivo*. Wild type (WT), PGS (scrambled) and ANXA1 KO MIA PaCa-2 cells were engrafted orthotopically in SCID mice. No differences were found about PC primary mass, conversely liver metastatization appeared particularly reduced in ANXA1 KO MIA PaCa-2 engrafted mice. In summary, we show that intracellular ANXA1 is able to preserve the cytoskeleton integrity and to maintain a malignant phenotype *in vitro.* The protein has a relevant role in the metastatization process *in vivo,* as such it appears attractive and suitable as prognostic and therapeutic marker in PC progression.

Pancreatic cancer (PC) is the fourth leading cause of death in the West World countries with a 5-year survival rate of only 3% and a median survival of less than 6 months^1^. Due to a lack of specific symptoms and limitations in diagnostic methods, the disease often eludes detection during its formative stages^2^,^3^. The aetiology of PC remains poorly defined, although important clues of disease pathogenesis emerged from epidemiological and genomic studies.

Numerous disturbances of biological pathways have been found in PC insurgence leading to tumour development and progression. Comparisons of protein profiles between PC and normal pancreas highlighted several proteomic alterations including the over-expression of annexin A1 (ANXA1) protein^4^,^5^,^6^.

ANXA1 is a member of the annexin family, comprising 12 other members. Its structural core is constituted by four homologous segments and is surrounded by a C-term, which accommodates the Ca^2+^-binding sites cations, and a N-term domain likely responsible for the main biological effects especially following protein proteolytic cleavage and/or secretion outside cells. In the last decades, many research groups focused on the specific roles played by ANXA1 in cancers relatively to its extracellular localization, particularly once formyl peptide receptors (FPRs) were uncovered as interactors of the protein^7^. Ever since, the ANXA1/FPR complex has been involved in the progression of several types of cancer including colon rectal, gastric, prostate, breast and melanoma^8^,^9^,^10^,^11^,^12^,^13^,^14^.

ANXA1 is a calcium- and phospholipid-binding protein involved in many membrane-related events, such as membrane organization domains and membrane-cytoskeleton signaling^15^. Although ANXA1 capability to mediate cytoskeletal dynamics interacting with proteins such as profilin, F-actin and K8/18^16^,^17^,^18^ was one of the first described characteristics of the protein, the physiopathological relevance of this property in cancer has been, with some exception, largely neglected.

We have recently reported a role for secreted ANXA1 in promoting PC cell motility as FPR ligand *in vitro*^19^.

In the present study, we have investigated the mechanisms by which intracellular ANXA1 contributes to PC cell phenotype, behaviour and metastatic potential independently of FPR pathway. Through genome-wide CRISPR (clustered, regularly interspaced, short palindromic repeat)/Cas9 (CRISPR associated protein 9) knockout (KO) technology, a genomic deletion of the protein in MIA PaCa-2 PC cell line (ANXA1 KO MIA PaCa-2) was obtained. The comparison of proteomic profiles of ANXA1 KO with MIA PaCa-2 control cells by liquid chromatography (LC)-tandem mass spectrometry (MS/MS), allowed the identification of several dysregulated proteins involved in proliferation, trafficking, metabolism and motility pathways. Functional experiments confirmed the disturbance of cytoskeletal dynamics showing a reduced capability of migration and invasiveness of ANXA1 KO MIA PaCa-2 cells. Finally, orthotopic xenografts obtained by engrafting WT, PGS and ANXA1 KO MIA PaCa-2 cells in the pancreas of SCID (Severe Combined Immunodeficiency) mice showed that the absence of intracellular ANXA1 did not affected the tumour mass but strongly decreased the metastatization process.

## Results

### Generation and validation of ANXA1 KO MIA PaCa-2 cell line and proteomic analysis

**Figure 1:**
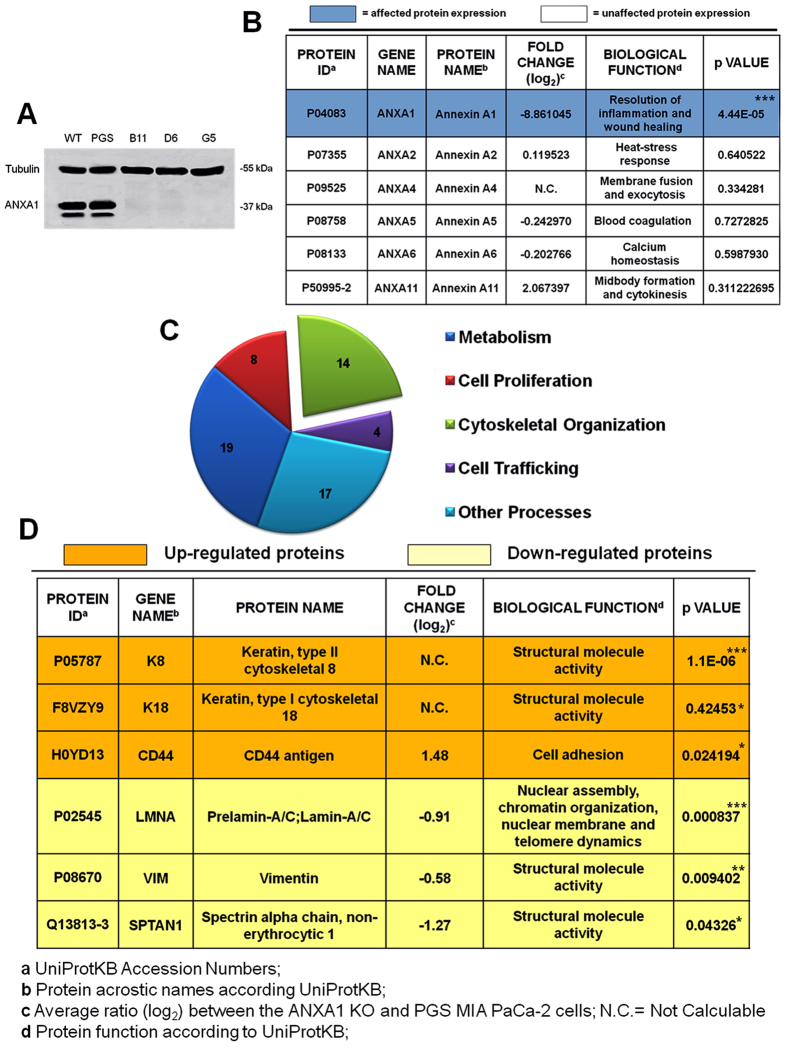
(**A**) Western blot showing B11, D6 and G5 KO clones for ANXA1. ANXA1 expression has been compared with WT and PGS MIA PaCa-2 and normalized on tubulin levels. (**B**) Proteins belonging to annexin superfamily identified by LC-MS/MS. ***p < 0.001. (**C**) Pie chart showing the absolute number of proteins identified as differentially expressed in ANXA1 KO MIA PaCa-2 cells by LC-MS/MS. The proteins were grouped based on their main reported function according to UniProtKB. (**D**) Proteins identified as differentially expressed by LC-MS/MS and involved in the process of cytoskeleton organization. *p < 0.05, **p < 0.01, ***p < 0.001.

In order to evaluate the effects of ANXA1 genomic deletion, we used CRISPR/Cas9 genome editing system^20^. By this technique 12 ANXA1 KO clones were derived from MIA PaCa-2 cells, chosen because of their aggressive phenotype and marked *in vivo* tumorigenicity. ANXA1 deletion was assessed by Western blotting and normalized against tubulin levels. In Fig. 1A, three of ANXA1 KO clones are reported and compared to WT and PGS MIA PaCa-2 ones, containing empty plasmid control.

Next, the proteins from cell lines were examined by LC-MS/MS to identify differences in protein expression co-existing with ANXA1 removal. Results showed that all the revealed annexins besides ANXA1 (ANXA2, ANXA4, ANXA5, ANXA6 and ANXA11) were not significantly affected in their expression by CRISPR/Cas9 genome editing technique (Fig. 1B).

Analysis of the LC-MS/MS results identified significant differences in the expression of 62 proteins; of these 26 appeared down-modulated and 36 were over-expressed in ANXA1 KO MIA PaCa-2 clones (see Supplementary Tables S1–S5). As represented in the pie chart (Fig. 1C), 4 are involved in cell trafficking; 8 in cell proliferation; 19 in metabolism; 14 in regulating cytoskeleton arrangement and 17 are proteins involved in other processes. We focused our attention on some of those implicated in cell shape remodelling because of ANXA1 ability to contribute to the cytoskeletal dynamics and to the establishment of a migratory and invasive phenotype^10^,^19^,^21^,^22^. These proteins are reported in Fig. 1D and are specified by protein ID (UniProtKB accession numbers), gene name (protein acrostic names according to UniProtKB), protein name, fold change (only average ratio between the ANXA1 KO and PGS MIA PaCa-2 cells are reported), biological function (according to UniProtKB) and relative p value.

### Validation of proteins identified as differentially expressed by LC-MS/MS analysis

Next, we performed experiments to validate some of the proteins identified as dissimilarly expressed by LC-MS/MS between ANXA1 KO and control MIA PaCa-2 cells.

**Figure 2:**
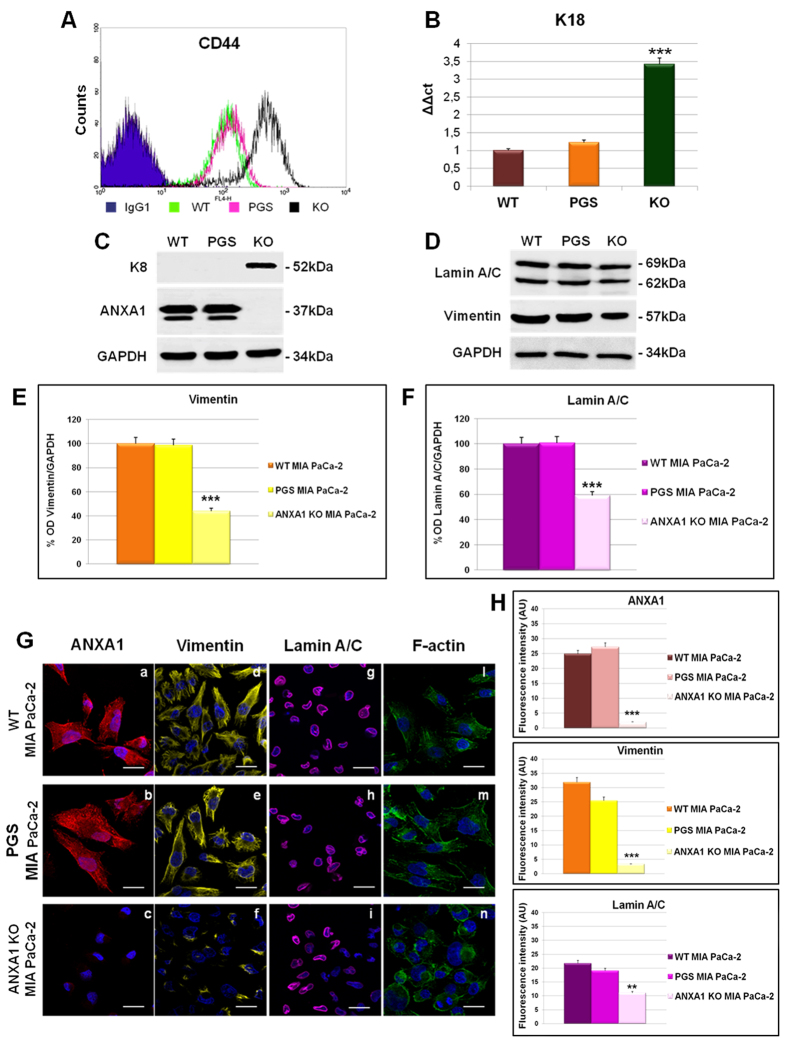
(**A**) Cell surface expression of CD44 was analyzed by flow cytometry. The violet area in the plot is relative to human IgG1; CD44 signals are showed in green for WT MIA PaCa-2, in pink for PGS MIA PaCa-2 and in black for ANXA1 KO MIA PaCa-2. (**B**) RT-PCR for K18 mRNA expression measured on levels of HPRT. Values are expressed using the delta-delta Ct method to derive relative fold change. ***p < 0.001. (**C,D**) Western blots showing K8, ANXA1, lamin A/C, vimentin and GAPDH expression in WT, PGS and ANXA1 KO MIA PaCa-2 cells. (**E,F**) Vimentin and lamin A/C relative expression was analyzed by densitometry. The optical density of the protein bands was normalized on GAPDH levels giving to the control band an arbitrary value of 100. The blots were exposed to Las4000 (GE Healthcare Life Sciences) and the relative intensities of bands were determined using ImageQuant software (GE Healthcare Life Sciences). (**G**) Immunofluorescence analysis to detect ANXA1 (red; panels a, b, c), vimentin (yellow; panels d, e, f), lamin A/C (purple; panel g, h, i) and F-actin (green; panels l, m, n) in WT, PGS and ANXA1 KO MIA PaCa-2. Nuclei were stained with DAPI. Magnification 63 × 1.4 NA. Bar = 10 μm. (**H**) Fluorescence intensity for ANXA1, vimentin and lamin A/C signals (arbitrary units, A.U.) using ImageJ software; determined on 150 cells (for three independent experiments). The results relative to ANXA1 KO MIA PaCa-2 are representative ± SEM of almost three analyzed clones with a similar behaviour.

Validation of increased CD44 expression that has a particular importance in cell adhesion, was performed by FACS technique^24^,^25^,^26^. In Fig. 2A, the purple, the green and the blue lines refer to the CD44 expression in PGS, WT and ANXA1 KO MIA PaCa-2 cells, respectively; the violet area refers to the APC-conjugated human IgG1 used as technical control.

Keratin 8 (K8) and 18 (K18) have numerous roles in simple epithelia^27^,^28^,^29^ and an inversely correlation between K18 and ANXA1 expression has been reported^30^. In Fig. 2B, we show the increase of K18 by RT-PCR using the protein hypoxanthine phosphoribosyltransferase 1 (HPRT) as house-keeping gene. K8 increase was proved by Western blot results in Fig. 2C.

ANXA1 KO MIA PaCa-2 also showed reduced expression of vimentin, a protein of the intermediate filaments (IFs). We confirmed the down-modulation of this protein by Western blot and by immunofluorescence (Fig. 2D,E,G panels d–f; 2H). Moreover, we observed the down-modulation of lamin A/C (Fig. 2D,F,G panels g–i; 2H), another protein belonging to the IFs, which appears deregulated in some cancers^23^.

Finally, differently from basal conditions in which F-actin is well spread in filamentous and organized in bundles protruding towards the plasma membrane (Fig. 2G, panels l–m) and as expected from LS/MS-MS results, ANXA1 KO MIA PaCa-2 cells showed a disorganized cytoskeleton where F-actin appeared concentrated all around plasma membrane and not organized in the cytosol (Fig. 2G, panel n).

### Effects of ANXA1 KO on MIA PaCa-2 migration and invasion

**Figure 3:**
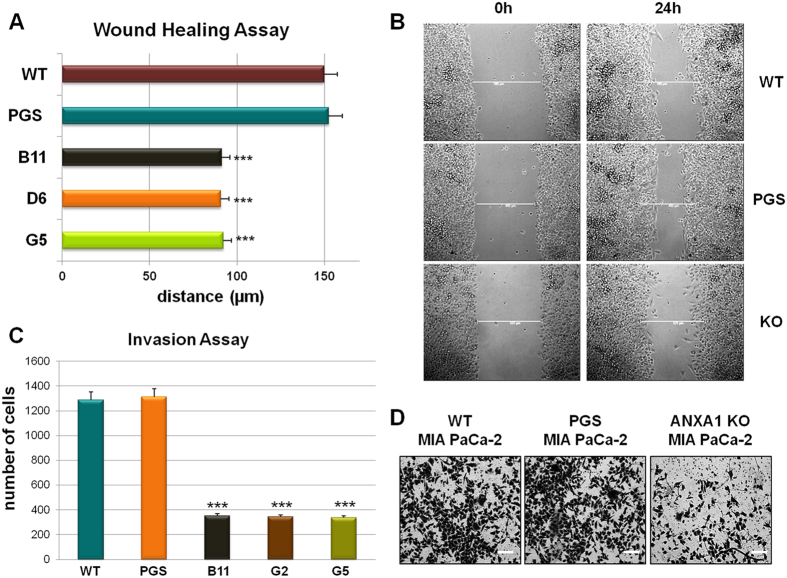
(**A**) Results of Wound healing assay on ANXA1 KO MIA PaCa-2; ***p < 0.01 vs WT and PGS MIA PaCa-2. The migration rate was determined by measuring the distances covered by individual cells from the initial time to the selected time-points (bar of distance tool, Leica ASF software). The data are representative of 5 independent experiments ± SEM. (**B**) Representative images captured by TIME LAPSE microscope of WT, PGS and ANXA1 KO MIA PaCa-2 at 0 h and 24 h from produced wounds. Magnification 10×. (**C**) Results of the invasion assay on ANXA1 KO, PGS and WT MIA PaCa-2 cells. Data represent mean cell counts of 12 separate fields per well ± SEM of 5 experiments. ***p < 0.001 vs WT and PGS MIA PaCa-2. (**D**) Representative images of analyzed fields of invasion assay. Magnification 20x. Bar = 150 μm

Several studies gave insights on the significant relationship between IFs and cell migration and its relationship to metastasis^31^. Based on our LC-MS/MS results and in order to confirm our previous observations with ANXA1 siRNA^19^, we performed some functional assays to assess the migration and invasion ability of ANXA1 KO MIA PaCa-2 cells. Both Wound healing and invasion assays were carried out on three ANXA1 KO clones compared with WT and PGS cells. In Fig. 3B, it is possible to observe a significant reduction of migration (about 40%) while a stronger loss of invasion capability (about 80%) is shown in Fig. 3C,D.

### Effects of ANXA1 KO on MIA PaCa-2 cell proliferation and apoptosis

**Figure 4:**
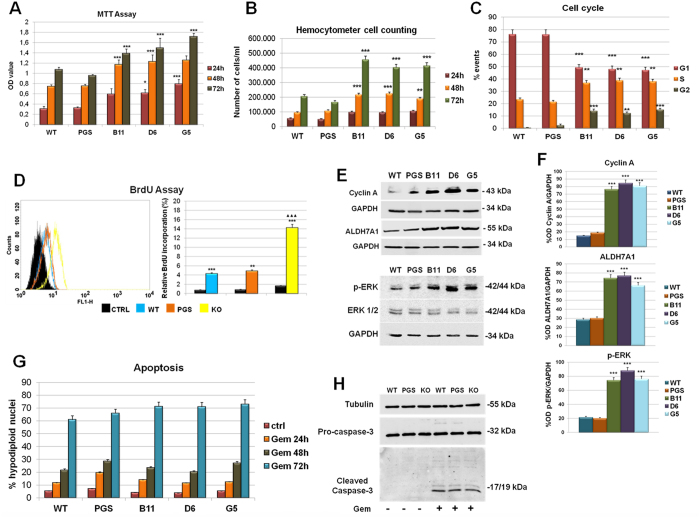
(**A**) MTT assay at 24, 48 and 72 h on WT, PGS and ANXA1 KO MIA PaCa-2 (clones B11, D6 and G5). (**B**) Hemocytometer cell counts of WT, PGS and ANXA1 KO at 24, 48 and 72 h of cell culture. **p < 0.01; ***p < 0.001.(**C**) Cell cycle analysis with PI staining, the graph is representative of 72 h of culture, after 24 h of serum starvation. (**D**) Quantitation of BrdU-positive cells. Representative forward scatter histograms and statistical analyses of BrdU incorporation by WT, PGS and ANXA1 KO cells. ***p < 0.001 vs control; ^▲▲▲^p < 0.001 vs WT cells. (**E**) Western blot of Cyclin A, ALDH7A1, Phospho-ERK and ERK on WT, PGS and ANXA1 KO MIA PaCa-2 clones as B11, D6 and G5. All protein levels are normalized on GAPDH levels. Data are representative of 5 experiments with similar results. (**F**) Densitometry for cyclin A, ALDH7A1 and p-ERK expression in WT, PGS and ANXA1 KO MIA PaCa-2. Protein bands were normalized on GAPDH levels. The blots were exposed to Las4000 (GE Healthcare Life Sciences) and the relative intensities of bands were determined using ImageQuant software (GE Healthcare Life Sciences). ***p < 0.001. (**G**) Analysis of hypodiploid (apoptotic) nuclei by cytofluorimetric assay of the effect of gemcitabine 10 μM at 24, 48 and 72 h on WT, PGS and ANXA1 KO MIA PaCa-2 (three clones B11, D6, G5). The data are representative of 5 experiments with similar results. (**H**) Western blot for pro- and cleaved caspase-3. WT, PGS and ANXA1 KO MIA PaCa-2 clones were treated or not with gemcitabine 10 μM for 72 h. Protein bands were normalized on tubulin levels. Image is representative of three independent experiments performed on WT, PGS, B11, D6 and G5 ANXA1 KO clones.

Based on the information obtained from proteomic analysis, we also analyzed cell growth and proliferation rate. ANXA1 KO MIA PaCa-2 propagate faster with respect to WT and PGS MIA PaCa-2 cells, as recognized by MTT assay (Fig. 4A). The major proliferative capacity of these cells is also confirmed by hemocytometer cell counting (Fig. 4B), by a significant increase of cell cycle S/G2 phases (Fig. 4C) and by Bromodeoxyuridine (BrdU) Incorporation Assay (Fig. 4D). For this reason we analyzed the expression of proteins that play a critical role in cell cycle^32^ and we found the increase of cyclin A (Fig. 4E,F).

In addition, Western blot analysis (Fig. 4E) and densitometry (Fig. 4F) also showed the increase of aldehyde dehydrogenase7A1 (ALDH7A1), that is involved in the regulation of cell cycle when has a cytosolic localization^33^.

**Figure 5:**
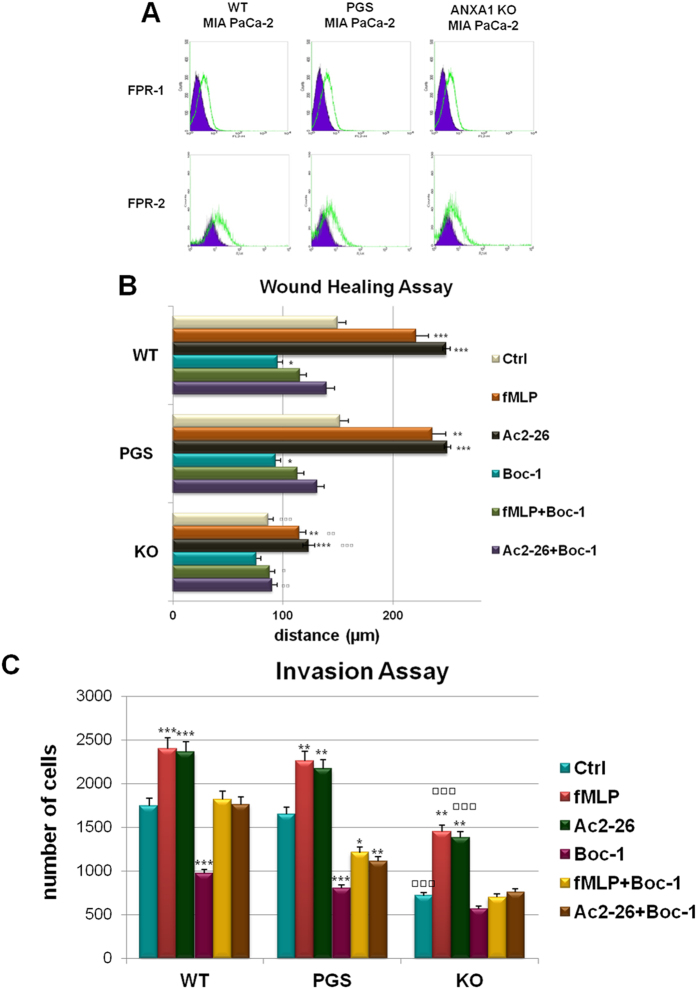
(**A**) Cell surface expression of FPR-1 and FPR-2 in WT, PGS and ANXA1 KO MIA PaCa-2 cells was analyzed by flow cytometry. The violet areas in the plots are relative to secondary antibody alone. FPR-1 and FPR-2 signals are showed in green. (**B**) Wound Healing assay on ANXA1 KO, PGS and WT MIA PaCa-2 treated or not with fMLP (50 nM), Ac2-26 (1 μM), Boc-1 (10 μM) fMLP + Boc-1 and Ac2-26 + Boc-1. The migration rate has been calculated as reported in Methods section, *p < 0.05, **p < 0.01 and ***p < 0.001 vs untreated controls; ^▫^p < 0.05, ^▫▫^p < 0.01 and ^▫▫▫^p < 0.001 vs WT and PGS MIA PaCa-2 relative experimental points. Invasiveness rates were measured in (**C**) *p < 0.05, **p < 0.01 and ***p < 0.001 vs untreated control; ▫p < 0.05, ▫▫p < 0.01 and ▫▫▫p < 0.001 vs WT and PGS MIA PaCa-2 relative experimental points. Data represent mean cell counts of 12 separate fields per well ± SEM of 5 independent experiments. The data relative to ANXA1 KO are representative of three analyzed clones.

On the other hand, the classical ERK family (p42/44 MAPK) is known to be an intracellular checkpoint for cellular mitogenesis^34^,^35^. The increase of the phosphorylated isoform of ERK is reported in the Western blot analysis of Fig. 5E and densitometry quantification of Fig. 4F.

In tumoral or inflammatory processes, ANXA1 has been described to be involved in apoptosis mechanisms. In PC there are no evidences about this aspect. However, we investigated the apoptosis induced by gemcitabine, to test the sensitivity of ANXA1 KO compared to WT and PGS MIA PaCa-2. As reported in Fig. 4G,H, MIA PaCa-2 cells showed a clear sensitivity to 10 μM gemcitabine only at 72 h and there were no significant changes in response to the drug in WT, PGS and ANXA1 KO cells. These data confirm that ANXA1 in PC is not involved in apoptotic process.

### ANXA1 KO MIA PaCa-2 cells partially respond to the pro-migratory and pro-invasive effects of extracellular ANXA1

The proteomic study has not identified any modification in the expression of the main proteins involved in the intracellular signalling triggered by FPRs. Furthermore, both FPR-1 and FPR-2 expression is retained in ANXA1 KO MIA PaCa-2 compared with WT and PGS cells, as shown by cytofluorimetric assay in Fig. 5A. In order to evaluate the activation of FPR pathways in ANXA1 KO MIA PaCa-2 clones, we used fMLP (specific receptor agonist), Ac2-26 (that mimics the ANXA1 N-terminal domain) and Boc-1 (receptor pan-antagonist) in migration and invasion assays. Both Wound healing (Fig. 5B) and cell invasion (Fig. 5C) assays confirmed that Ac2-26 and nFPR interaction induced an increase of migration and invasion rate in WT, PGS and KO MIA PaCa-2 cells. However, when treated with FPR agonists, ANXA1 KO MIA PaCa-2 clones migrate and invade significantly less (p < 0.001) if compared with WT and PGS MIA PaCa-2 cells, as evident in the histogram in Fig. 5C. Similarly to WT and PGS MIA PaCa-2 cells, Boc-1 is able to reduce fMLP and Ac2-26 effects also in KO clones. These findings highlight a crucial role for intracellular ANXA1 and for the protein/cytoskeleton interaction in PC progression, independently of FPR pathway activation.

### ANXA1 KO decreases the metastatic potential of MIA PaCa-2 cells *in vivo*

**Figure 6:**
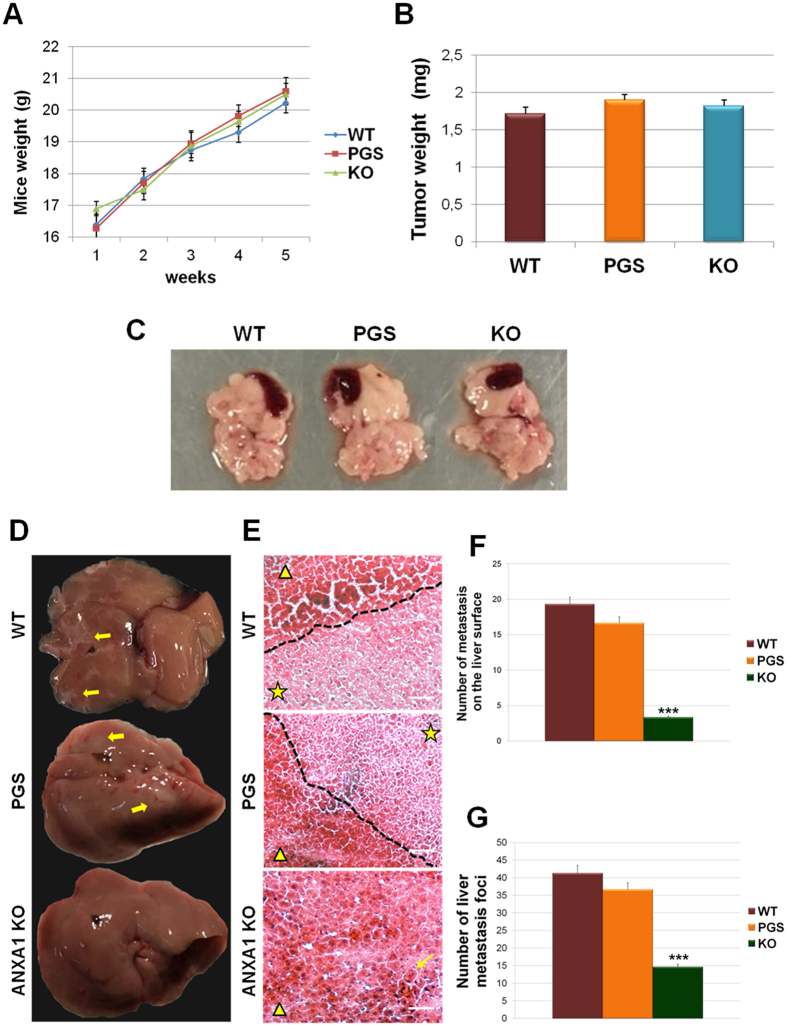
(**A**) Average body weight of mice measured weekly from the implantation until the sacrifice. (**B**) Histogram of tumour weights. (**C**) An exemplar image, including also the spleens, of the tumour volumes generated in pancreas by WT, PGS and ANXA1 KO MIA PaCa-2. (**D**) Photos of mice liver. Metastatic lesions were indicated by yellow arrows. (**E**) Liver sections have been stained through H&E. Metastatic foci were labelled by stars; normal tissue areas were marked by triangles. Bar = 100 μm. (**F**) Quantitative analyses of metastasis number on liver surface. ***p < 0.001. (**G**) Metastasis foci number on H&E tissue sections. ***p < 0.001.

In order to validate the data collected *in vitro*, the generated cell lines were implanted directly into the pancreas of female SCID mice as reported in Methods section. The animal wellness has been checked during all the experimental period evaluating their motility and measuring the weight once a week: no significant weight loss was found, (Fig. 6A). After 5 weeks from the implantation, mice were sacrificed and the tumours generated in the pancreas were evaluated. ANXA1 KO had no effect on primary cancer growth. As shown in Fig. 6, the tumour mass in mice implanted with ANXA1 KO MIA PaCa-2 did not appear significantly smaller if compared with those extracted from control mice, as confirmed by both the evaluation of tumour weight (panel B) and the macroscopic observation (panel C). We also determined whether ANXA1 depletion from highly invasive MIA PaCa-2 cells could reduce metastasis formation. Therefore, we analyzed the livers of the animals, which represent the first affected organs by the PC metastatic process. The livers harvested from mice injected with WT and PGS MIA PaCa-2 presented numerous metastasis (Fig. 6F), which were particularly notable as white areas on the surface of a brick-red organ (Fig. 6D, yellow arrows). Additionally, these livers lost their own physiological integrity with indented profiles and reduced compactness. On the other hand, the livers extracted from the animals implanted with ANXA1 KO MIA PaCa-2 retained their characteristic colour and tissue density and showed much less metastatic lesions (Fig. 6F). Moreover, H&E staining (Fig. 6E) revealed distinctly areas, compatible with metastatic lesions, in the livers of WT and PGS MIA PaCa-2 mice where it is possible to observe smaller cells and a different morphology (star signed areas of the relative images). The number of these areas was significantly lower in livers of mice implanted with ANXA1 KO MIA PaCa-2 cells (Fig. 6G). The number of the liver metastasis was quantified as described in Methods section.

## Discussion

ANXA1 protein can perform numerous functions in cancer acting as either a tumour suppressor or an oncogene, depending on the cancer type^36^,^37^,^38^,^39^. In tissues from patients with PC, ANXA1 protein is over-expressed and has been correlated with poor differentiation and prognosis^5^,^6^ although its role in the PC malignant transformation remains inadequately defined.

In the present study, we report that ANXA1 has a role in PC cell cytoskeleton dynamics *in vitro* and that it is involved in metastatic processes *in vivo*.

Among the other cells, MIA PaCa-2 PC cells are commonly used to induce tumour xenografts in mice because of their strong capability to develop the largest tumoral mass and metastasis^40^. Hence, we have selected this cell line to obtain ANXA1 KO cells generated by the CRISPR/Cas9 genome editing technology^41^,^42^.

Thus, a proteomic analysis was performed to evaluate the expression of proteins possibly affected by the lack of ANXA1 in MIA PaCa-2 cells. Changes in expression of other components of the annexin family of proteins were not observed confirming the absence of important off target effects. Among the 26 down-modulated and 36 over-expressed detected proteins (see Supplementary Tables S1–S5), of particular interest were some proteins implicated in the maintenance of the cytoskeleton stability and plasticity and well known to have aberrant expression in cancers.

One of these is the adhesion molecule CD44 that we found significantly up-regulated in ANXA1 KO compared to WT and PGS MIA PaCa-2 cells. This protein attracted great interest since the expression of CD44v (an alternative isoform of CD44) was associated with a metastatic phenotype in primary tumours^43^. In several cells, CD44 interacts with the cytoskeleton through two adaptors: ezrin/radixin/moesin (ERM) proteins which directly interact with actin filaments^44^,^45^, and ankyrin^46^ which directly interacts with spectrin^47^. The latter cooperates with short actin oligomers, accessory proteins, and phosphatidyl-inositol lipids to form sub-membrane structures^48^. Spectrin and/or ankyrin loss in cells caused modifications of the actin cytoskeleton, such as leak of stress fibres, alterations of focal adhesions and altered expression of several integrins^49^. Interestingly, our LC-MS/MS results showed in parallel with CD44 up-regulation, a significant spectrin (p = 0.04326) down-regulation, suggesting the disruption of the CD44 signalling pathway.

Critical structural proteins we found down-regulated by proteomic approach in ANXA1 KO MIA PaCa-2, were lamin A/C and vimentin, both validated by Western blot and immunofluorescence analysis. Lamin A/C belongs to the family of lamins, IF proteins representing the most important components of the cell nucleus, and necessary for regulating the nuclear organization and the circulation of macromolecules to and from the nucleus. An increasing number of human pathologies have been associated to defects in nuclear envelope-specific proteins. Interestingly, some of these disorders revealed that many of these proteins have important roles in cytoskeletal organization and dynamics^50^. Notably, in normal human pancreas, very weak expression of lamin A/C was observed in acinar and in the ductal cells. In contrast PC retains a larger expression of lamin A/C^51^. Vimentin is reported as an important mesenchymal marker, and plays a central role in EMT in malignant tumours including cellular adhesion and migration, cytoplasmic microtubule assembly and cytoskeleton remodelling^51^.

Many cancer cell lines exhibit both a vimentin-based and a keratin-based IF network that underlines distinct intracellular organization and regulation^31^. The keratin profile can be actually used to distinguish the several phases of physiological morphogenesis, both in the embryonic development and in case of damage, as well as in pathological tumour development^52^. In the pancreatic ducts that are composed of simple epithelia with a peculiar keratin profile, the basal cells exhibit K7, 19, 20 expression while the luminal ones express K8 and 18. Cells with a basal phenotype have a higher proclivity to undergo invasion therefore “basalness” has now been related to a high malignant phenotype. In our ANXA1 KO MIA PaCa-2 cells, we found a significant up-regulation of the keratin couple K8/18, a data that was particularly consistent with their observed less aggressive phenotype. These data demonstrate a likely inverse relationship between ANXA1 expression and the degree of tumour differentiation.

Disturbance of cytoskeletal dynamics in ANXA1 KO MIA PaCa-2 cells was confirmed by examination of F-actin staining: although the protein expression was not modified, in ANXA1 KO MIA PaCa-2 cells F-actin appeared disordered in the cytosol and there is an evident lack of lamellipodia and stress fibres, some of the sub-cellular structures which are known to be assigned to cell migration^53^.

Since cell motility is driven by the assembly of both protrusive and contractile actin filaments, the disruption of this organization led us to investigate the migration and invasion processes that were consistently reduced in ANXA1 KO MIA PaCa-2. These results show that ANXA1 directly and/or indirectly mediates the cytoskeleton integrity and distribution, controlling the rate of the actin filament assembly and disassembly.

The claim that ANXA1 could on the whole contribute to the maintenance of a more aggressive phenotype of PC cells is further confirmed by other data. Particularly, we found a greater proliferation rate of ANXA1 KO MIA PaCa-2, a typical feature of more differentiated tumours, which could be, in this way, more easily attacked by chemotherapic agents. The precise mechanism by which ANXA1 regulates cellular proliferation remains to be fully determined. However, it has been shown that cells over-expressing ANXA1 proliferate at a slower rate^54^. Tumor growth and invasion are two distinct processes directed by different molecular signaling pathways. Particularly in glioma cells, experimental evidence suggests that there may be an inherent and inverse correlation between cell motility and proliferation^55^. This antagonistic action of cell movement and mitotic activity is referred as a migration/proliferation dichotomy or “Go-or-Grow” behavior. Cell density or non-permissive substrates that inhibit cell motility favor a more proliferative phenotype, conversely, active migration suppresses cell proliferation^56^,^57^.

Nevertheless, ANXA1 is not involved in the apoptosis process mediated by gemcitabine, the main anti-tumoral agent still used in the clinical practice.

The regulation of cancer cell behaviour by stroma (endothelial, fibroblast and immune cells) and *vice-versa* is one of the important aspect of tumour development^58^. This cross-talk is carried out by secreted molecules such as peptides, chemo-attractants and growth factors from all compartments^59^.

It has been reported that the extracellular form of ANXA1 positively affects migration and invasiveness through FPR activation in several tumours^8^,^9^,^10^,^11^,^12^,^13^,^14^. Recently, we found that MIA PaCa-2 cells secrete high levels of full length (37 kDa) and cleaved (33 and 3 kDa) extracellular ANXA1 and that these forms work as promoting factors in migration and invasion processes^19^.

Here, we show that although ANXA1 KO MIA PaCa-2 cells acquired a more rapid capability of migrating and invading in the presence of the FPR agonists, the achieved migration and invasion rate is considerably reduced compared to that of WT and PGS MIA PaCa-2 cells.

These data led us to hypothesize a central role of the intracellular ANXA1 as essential counterpart promoting malignant potential of PC tumour cells.

Several studies including one using ANXA1-KO mice, showed that the specific inhibition of the stroma-derived-ANXA1 expression is able to significantly reduce tumour growth, angiogenesis and metastasis^60^,^61^.

Therefore, we created orthotopic xenografts with WT, PGS and ANXA1 KO MIA PaCa-2 cells in SCID mice which keep stroma-derived ANXA1, to investigate *in vivo* the relevance of intracellular ANXA1 in tumour progression. In accordance with our previous results, ANXA1 KO did not affect primary tumour growth, but significantly reduced the number of metastasis, above all those on the livers, which represent the first organs affected by PC metastatization. The livers harvested from mice implanted with WT and PGS MIA PaCa-2 cells lost their tissue compactness and present lots of white areas, relative to metastatic lesions. The heterogeneous morphology has been further confirmed by the presence of smaller cells as revealed by H&E staining. Differently, the same organs collected from mice with ANXA1 KO MIA PaCa-2 xenografts showed a homogeneous cell distribution with much less metastatic lesions.

The correlation with ANXA1 and the malignant transformation of PC has been well reported and as in other tumour models, the molecular mechanism has been generally ascribed to its interaction, as an extracellular factor, with FPRs^7^,^8^,^9^. Although intra- and extracellular ANXA1 forms should be considered as two faces of the same aspect, the cytosolic counterpart functions have not been extensively examined at molecular level with the exception of breast cancer where the protein is able to induce EMT process via up-regulation of TGF-β signaling^62^ and malignant squamous epithelial cells where conversely, ANXA1 induces cell proliferation by EGFR stabilization, EGF production and cPLA2 activation^63^.

In this work, we highlight for the first time the effects of intracellular ANXA1 loss on cell motility and metastatic potential of PC cells as its knocking out alters expression profiles of several structural proteins involved in cytoskeletal dynamics *in vitro* and has an important role in raising metastatization *in vivo* independently of FPR pathway activation. If these ANXA1 effects on cytoskeletal elements are direct or not remain a very interesting issue for further investigations.

## Methods

### Cell Cultures

MIA PaCa-2 cells were purchased from ATCC (ATCC CRL-1420; USA) and cultured following provider’s instructions (www.lgcstandards-atcc.org).

### Western blotting

Protein expression was examined by Western blot, as previously described^19^. Proteins were visualized using the chemioluminescence detection system (Amersham) after incubation with primary antibodies against ANXA1 (rabbit polyclonal; 1:10000; 71–3400, Invitrogen), α-tubulin (clone DM1A; 1:1000; Sigma-Aldrich), K8 (mouse monoclonal, clone M20; 1:1000; Abcam), lamin A/C (mouse monoclonal, clone A-5; 1:1000; Santa Cruz Biotechnologies), vimentin (clone E-5; 1:1000; Santa Cruz Biotechnologies), GAPDH (mouse monoclonal, clone G-9; 1:1000 Santa Cruz Biotechnologies), cyclin A (rabbit polyclonal, clone H-432; 1:1000; Santa Cruz Biotechnologies), ALDH7A1 (rabbit monoclonal, clone EP1935Y; 1:1000; Abcam), ERK and p-ERK (mouse monoclonal, clone MK12; rabbit monoclonal, clone Aw39; 1:1000; Cells Signalling), pro-caspase-3 (rabbit polyclonal; #9662; 1:1000; Cell Signaling) and cleaved caspase-3 (rabbit polyclonal; #9664; 1:1000; Cell Signaling). The blots were exposed to Las4000 (GE Healthcare Life Sciences) and the relative band intensities were determined using ImageQuant software (GE Healthcare Life Sciences). Results were considered significant if p < 0.01.

### Confocal Microscopy

WT, PGS and ANXA1 KO MIA PaCa-2, fixed in p-formaldehyde (4% v/v in PBS; Lonza), were permeabilized or not with Triton X-100 (0.4% v/v in PBS; Lonza), blocked with goat serum (20% v/v in PBS; Lonza) and then incubated with anti-ANXA1 antibody (rabbit polyclonal; 1:100; 71–3400, Invitrogen), anti-vimentin (mouse monoclonal, clone E-5; 1:500; Santa Cruz Biotechnologies), anti-lamin A/C (mouse monoclonal, clone 636; 1:450; Novocastra) overnight at 4 °C. After two washing steps, cells were incubated with anti-rabbit and/or anti-mouse AlexaFluor (488 and/or 555; 1:1000; Molecular Probes) for 2 hours at room temperature (RT), then with FITC-conjugated anti-F-actin (5 μg/ml; Phalloidin-FITC, Sigma-Aldrich) for 30 minutes at RT in the dark. To detect nucleus, samples were excited with a 458 nm Ar laser. A 488 nm Ar or a 555 nm He-Ne laser was used to detect emission signals from target stains. Samples were vertically scanned from the bottom of the coverslip with a total depth of 5 μm and a 63X (1.40 NA) Plan-Apochromat oil-immersion objective. Images and scale bars were generated with Zeiss ZEN Confocal Software (Carl Zeiss MicroImaging GmbH) and presented as single stack. Images were processed using ImageJ software (NIH, Bethesda, MD, USA), Adobe Photoshop CS version 5.0, and figures assembled using Microsoft PowerPoint (Microsoft Corporation). Fluorescence intensity analyses were performed using ImageJ software (NIH, Bethesda, MD, USA) as following described. Briefly, ten field images from a single coverslip were randomly selected and registered for each experimental condition identifying distinct cells by DAPI nuclear staining. Then, individual cell total area was selected using area selection tool and fluorescence intensity value was measured (ImageJ Arbitrary Units; A.U.) subtracting background. The obtained mean value was used to compare experimental groups.

### Flow cytometry

WT, PGS and ANXA1 KO MIA PaCa-2 cells were harvested at a number of 1 × 10^6^ and analyzed for FPR-1, FPR-2 and CD44 proteins as previously described^19^. Briefly, pellets were incubated on ice for 1 hour in PBS containing a primary antibody against FPR-1 (rabbit polyclonal, clone H-230; 1:500, Santa Cruz Biotechnology) or a primary antibody against FPR-2 (mouse monoclonal, clone GM-1D6; 1:100, Genovac). Then, cells incubated on ice for 1 hour in 100 μl of PBS containing AlexaFluor 488 anti-rabbit (1:1000; Molecular Probes) or Alexa-Fluor 488 anti-mouse (1:1000; Molecular Probes). To evaluate CD44 expression, cells were incubated on ice for 30 minutes in 100 μl of PBS containing APC-conjugated CD44 anti-human antibody (mouse monoclonal, clone G44–26; BD Pharmigen), APC-conjugated human IgG1 (BD Pharmigen) was used as scrambled. The cells were analyzed with Becton Dickinson FACScan flow cytometer using the Cells Quest program.

### RNA isolation and quantitative RT-PCR

mRNA levels of WT, PGS and ANXA1 KO MIA PaCa-2 were analysed by Real- time PCR using the Light Cycler 480 II instrument (Roche). 1 μg of total RNA extracted from cells was reverse transcribed into cDNA with Transcriptor First Strand cDNA Synthesis Kit (Roche). cDNAs were processed using Light Cycler 480 Probes Master mix and Real Time Ready Catalog Assay primers (Roche) for K18 (5′-AATGGGAGGCATCCAGAACGAGAA-3′, 3′-TTCTTCTCCAAGTGCTCCCGGATT-5′) and HPRT1 (5′-GACCAGTCAACAGGGGACAT-3′, 3′-CCTGACCAAGGAAAGCAAAG-5′) following the manufacturer’s protocols (http://www.lifescience.roche.com). Results were analyzed using the Delta-Delta CT method.

### *In vitro* Wound-Healing

A wound was produced on the confluent monolayer of WT, PGS and ANXA1 KO MIA PaCa-2 by scraping the cells with a pipette tip. Next, cells were incubated or not with fMLP (50 nM; Sigma-Aldrich), Ac2-26 (1 μM; Tocris Bioscience), Boc-1 (10 μM; Bachem AG) or in growth medium as control. The wounded cells were then incubated at 37 °C in a humidified and equilibrated (5% v/v CO_2_) incubation chamber of an Integrated Live Cell Workstation Leica AF-6000 LX. A 10x phase contrast objective was used to record cell movements with a frequency of acquisition of 10 minutes. The migration rate of individual cells was determined by measuring the distances covered from the initial time to the selected time-points (bar of distance tool, Leica ASF software) at 24 hours after seeding or treatments. For each condition five independent experiments were performed. For each wound five different positions were registered, and for each position ten different cells were randomly selected on both side of the scratch to measure the migration distances. All transiently amplifying cells were excluded from statistical analyses.

### Invasion Assay

MIA PaCa-2 invasiveness was studied using the Trans-well Cell Culture (12 mm diameter, 8.0-fim pore size) purchased form Corning Incorporated (USA), as previously described^19^. Briefly, at 24 hours after seeding or treatments, the Trans-well Cell Culture chambers were washed twice with PBS and fixed with 4% p-formaldehyde for 10 minutes, then with 100% methanol for 20 minutes. Following, the fixed cells were stained with crystal violet (0, 5% w/v in a v/v solution of 20% methanol/distilled water; Merck Chemicals) for 15 minutes. After that, the chambers were washed again in PBS and cleaned with a cotton bud to remove crystal violet exceedance. The number of cells that had migrated to the lower surface was counted in twelve random fields using EVOS light microscope (10X) (Life technologies Corporation).

### MTT assay

WT, PGS and ANXA1 KO MIA PaCa-2 cells were harvested at the indicated times (24, 48 and 72 hours) and cell viability was calculated as previously described^10^. The optical density (OD) of each well was measured with a spectrophotometer (Titertek Multiskan MCC/340) equipped with a 620 nm filter.

### Hemocytometer counting

2 × 10^4^ WT, PGS and ANXA1 KO MIA PaCa-2 cells were seeded in a 12 well-plate. After 12 hours of serum starvation to obtained cell cycle synchronization, cells were harvested at 24, 48 and 72 hours. Equal volumes of 0.4% trypan blue stain and of the cell suspension were mixed. Approximately 10 μl of trypan blue/cell mix were put at the edge of the cover-slip of the Burker chamber and the haemocytometer grid was visualized under the optical microscope Axiovert 40 CFL (Carl Zeiss MicroImaging GmbH, 10x). To calculate the viable cells/ml, the average number of cells in one large square has been multiplied by the dilution factor (2) and then by 10^4^.

### BrdU incorporation assay

1.5 × 10^5^ WT, PGS and ANXA1 KO MIA PaCa-2 cells were seeded in a 12 well-plate, after 12 hours of serum starvation to obtained cell cycle synchronization, BrdU (10 μM; eBioscience) was administered in complemented growth medium for 6 hours. Cells were harvested, washed twice and fixed with Foxp3Fix/Perm buffer 1x (Becton Dickinson) for 1 hours at 4 °C in the dark. Then 30 μg DNase I (Sigma Aldrich) was added for 1 hours at 37 °C in the dark. Next, cells were washed twice and incubated with an anti-BrdU FITC-conjugated antibody (mouse monoclonal; clone BU20A; 1:200; eBioscience). Finally, cells were washed twice and analyzed with Becton Dickinson FACScan flow cytometer using the Cells Quest program.

### Apoptosis detection

The effect of gemcitabine 10 μM (Sigma-Aldrich) on cell death was checked by propidium iodide (PI) (Sigma-Aldrich) staining and flow cytometry at 24, 48 or 72 hours from the drug administration. The percentage of the cells in the hypodiploid nuclei was analyzed and calculated with FACScan cytometer (Becton-Dickinson) by Cell Quest program. Cellular debris were excluded from the analysis by raising the forward scatter threshold and the DNA content of the nuclei was registered on logarithmic scale. The percentage of the cells in the hypodiploid region was calculated.

### Cell cycle analysis

After 24 hours serum starvation, WT, PGS and ANXA1 KO MIA PaCa-2 cells were grown in complemented medium for 24, 48 and 72 hours. Cell cycle profiles were evaluated by DNA staining with PI solution using a FACScan cytometer (Becton Dickinson) using Cell Quest evaluation program. The distinct cell cycle phases were determinate using ModFit LT analysis software (Becton Dickinson).

### CRISPR-Cas9 plasmid and selection of ANXA1 KO MIAPaCa-2 clones

The optimized gRNA construct, targeting ANXA1, and the Cas9 expression construct, pGS-gRNA- Cas9-Neo, were obtained from GenScript (USA). The ANXA1 exon located at the third coding exon was selected for guide RNA design. The gRNA recognizes the following target sequence 5′-GATCAGCGGTGAGCCCCTA-3′. For the establishment of ANXA1 KO cell lines, pGS-gRNA-Cas9-Neo containing neomycin resistance gene (Genscript, USA) was used. MIA PaCa-2 cells were transfected using Lipofectamine 2000 Reagent (Life technologies), according to the manufacturer’s instructions (http://www.lifetechnologies.com). One week later, the cells were subject to selection by 700 μg/ml neomycin (Euroclone). Cell clones were obtained following the limit dilution. Single cell colonies were selected, gradually expanded and analyzed by Western blotting to pick ANXA1 KO clones.

### LC-MS/MS

Three different protein extracts for each cell line were prepared; each extract was analyzed in triplicate. The final comparison was performed considering 8 experiments for each cell line, since technical problems occurred for two samples. This sample set was large enough to provide statistically significant results. To perform the proteomic analysis, 30 μg of proteins were resolved on a 1D SDS-PAGE. Each resulting gel line was divided in 10 bands that were reduced to smaller pieces, washed with water and dehydrated in acetonitrile, reduced with dithiothreitol and alkylated with iodoacetamide. All bands underwent over-night in-gel digestion using trypsin. Resulting peptide were analyzed by LC/MS/MS using an Orbitrap XL instrument (Thermo Fisher, Waltham, MA, USA) equipped with a nano-ESI source coupled with a nano-ACQUITY capillary UPLC (Waters): peptide separation was performed on a capillary BEH C18 column (0.075 mm × 100 mm, 1.7 μm, Waters) using aqueous 0.1% formic acid (A) and CH_3_CN containing 0.1% formic acid (B) as mobile phases. Peptides were eluted by means of a linear gradient from 5% to 50% of B in 90 minutes and a 300 nL min^−1^ flow rate. Mass spectra were acquired over an *m/z* range from 400 to 1800.

To achieve protein identification, MS and MS/MS data underwent MxQuant software (version 1.4.1.2) analysis: Andromeda search engine was used on UniProt human protein database (UP000005640, 70220 sequences). Parameters sets were: trypsin cleavage; carbamidomethylation of cysteine as a fixed modification and methionine oxidation as a variable modification; a maximum of two missed cleavages; false discovery rate (FDR), calculated by searching the decoy database, 0.05. Label-free quantitation (LFQ) was performed using MaxQuant software. The minimum ratio count for LFQ was set to 2, and the match-between-runs option was enabled. Significance p-values were calculated using Perseus software (version 1.4.1.35) applying the Student’s *t*-test (for comparing two value sets). P < 0.05 was considered statistically significant. Proteins showing a change in levels > 2-fold and a p-value < 0.05 were selected as biologically regulated proteins; these proteins also underwent GO analysis.

The differentially expressed proteins were subjected to PANTHER classification system, version 9.0 (http://www.pantherdb.org/), for molecular function-based gene ontology analysis. Genes were categorized into multiple different functional groups. Mass spectrometry proteomics raw data are available in Supplementary Table S6.

### Orthotopic xenografts in immunodeficient mice

SCID mice (6–8 week-old females) were obtained from Charles River (Italy) and bred under pathogen-free conditions in the Animal Facility of the University of Salerno. Animal experiments were approved by the Italian Health Ministry (authorization no. 819/2015-PR) and performed according to Italian law 26/2014. The orthotopic implantation was performed as previously reported^64^. Briefly, mice were anesthetized by inhalation of isoflurane. The entire operation was done in a hood, with sterile technique maintained throughout. A 1-cm incision was made in the left upper quadrant of the abdomen, and the pancreas was exposed by retraction of the spleen. 1 × 10^6^ WT, PGS and ANXA1 KO MIA PaCa-2 were resuspended in a mixture of PBS and matrigel (Becton Dickinson) (1:1) and injected directly into the pancreas using a 29 gauge needle of a Hamilton syringe. The peritoneum was then closed with 5.0 dissolvable suture (AgnTho’s AB) and the skin incision closed with wound clips (Azlet). After 5 weeks from the implantation, mice were sacrificed and organs were excised, weighed and analyzed. Metastases lesions on the liver surface were observed and quantified by gross anatomy using a dissecting microscope.

### H&E tissue staining

The livers were harvested, washed and fixed in a solution of p-formaldehyde. Then they were incubated in a sucrose solution to guarantee the cryoprotection. The frozen organ sections were cut on a Leica CM 1950 cryostat at 10–12 μm, mounted directly on super frost slides (Thermo Scientific), and processed for haematoxylin and eosin (H&E) staining. Briefly, cryostat sections were dehydrated for 5 minutes with cold acetone and then rehydrated. Next, slides were placed in haematoxylin stain for 9 minutes, rinsed in alcoholic acid, differentiated in 80% alcohol and stained with eosin for 2.5 minutes, rinsed in 95% ethanol, dehydrated with absolute ethanol and cleared in xylenes for 4 minutes. The images were taken through the Axio Observer microscope (4, 10 and 40X) (Carl Zeiss MicroImaging GmbH). Six sections from 9 animals (three for each condition) were selected and the number of metastatic foci was counted using ImageJ software (NIH, Bethesda, MD, USA).

### Statistical analysis

Data analyses and statistical evaluations were carried out using Microsoft Excel; the number of independent experiments, standard deviations/errors, and p-values are indicated in the figure legends. All results are the mean ± S.E.M. of at least 3 experiments performed in triplicate. Statistical comparisons between groups were made using two-way ANOVA or unpaired, two-tailed t-test comparing two variables. Differences were considered significant if p < 0.05, p < 0.01 and p < 0.001.

## Additional Information

**How to cite this article**: Belvedere, R. *et al*. Annexin A1 contributes to pancreatic cancer cell phenotype, behaviour and metastatic potential independently of Formyl Peptide Receptor pathway. *Sci. Rep.* **6**, 29660; doi: 10.1038/srep29660 (2016).

## Supplementary Material

Supplementary Information

## References

[b1] Siegel R. L., Miller K. D. & Jemal A. Cancer statistics. CA Cancer J Clin 2015. 65, 5–29 (2015). 10.3322/caac.21254 25559415

[b2] Ferlay J. . Cancer incidence and mortality worldwide: sources, methods and major patterns in GLOBOCAN 2012. Int J Cancer. 136, E359–386 (2015). 25220842 10.1002/ijc.29210

[b3] Bond-Smith G., Banga N., Hammond T. M. & Imber C. J. Pancreatic adenocarcinoma. BMJ. 344, e2476 (2012). 22592847 10.1136/bmj.e2476

[b4] Yoshida K. . Proteomic differential display analysis for TS-1-resistant and -sensitive pancreatic cancer cells using two-dimensional gel electrophoresis and mass spectrometry. Anticancer Res. 31, 2103–2108 (2011). 21737628

[b5] Chen C. Y., Shen J. Q., Wang F., Wan R. & Wang X. P. Prognostic significance of annexin A1 expression in pancreatic ductal adenocarcinoma. Asian Pac J Cancer Prev. 13, 4707–4712 (2012). 23167407 10.7314/apjcp.2012.13.9.4707

[b6] Bai X. F. . Overexpression of annexin 1 in pancreatic cancer and its clinical significance. World J Gastroenterol. 10, 1466–1470 (2004). 15133855 10.3748/wjg.v10.i10.1466 PMC4656286

[b7] Walther A., Riehemann K. & Gerke V. A novel ligand of the formyl peptide receptor: annexin I regulates neutrophil extravasation by interacting with the FPR. Mol Cell. 5, 831–40 (2000). 10882119 10.1016/s1097-2765(00)80323-8

[b8] Babbin B. A. . Annexin I regulates SKCO-15 cell invasion by signaling through formyl peptide receptors. J Biol Chem. 281, 19588–19599 (2006). 16675446 10.1074/jbc.M513025200

[b9] Cheng T. Y. . Annexin A1 is associated with gastric cancer survival and promotes gastric cancer cell invasiveness through the formyl peptide receptor/extracellular signal-regulated kinase/integrins beta-1-binding protein 1 pathway. Cancer. 118, 5757–57677 (2012). 22736399 10.1002/cncr.27565

[b10] Bizzarro V. . Annexin A1 is involved in the acquisition and maintenance of a stem cell-like/aggressive phenotype in prostate cancer cells with acquired resistance to zoledronic acid. Oncotarget. 6, 25076–25092 (2015). 26312765 10.18632/oncotarget.4725 PMC4694816

[b11] Khau T. . Annexin-1 signals mitogen-stimulated breast tumor cell proliferation by activation of the formyl peptide receptors (FPRs) 1 and 2. FASEB J. 25, 483–496 (2011). 20930115 10.1096/fj.09-154096

[b12] Rondepierre F. . Proteomic studies of B16 lines: involvement of annexin A1 in melanoma dissemination. Biochim Biophys Acta. 1794, 61–69 (2009). 18952200 10.1016/j.bbapap.2008.09.014

[b13] Boudhraa Z. . Annexin A1 in primary tumors promotes melanoma dissemination. Clin Exp Metastasis. 31, 749–760 (2014). 24997993 10.1007/s10585-014-9665-2

[b14] Boudhraa Z. . Characterization of pro-invasive mechanisms and N-terminal cleavage of ANXA1 in melanoma. Arch Dermatol Res. 306, 903–914 (2014). 25362540 10.1007/s00403-014-1517-z

[b15] Gerke V., Creutz C. E. & Moss S. E. Annexins: linking Ca^2+^ signalling to membrane dynamics. Nat Rev Mol Cell Biol. 6, 449–61 (2005). 15928709 10.1038/nrm1661

[b16] Alvarez-Martinez M. T., Porte F., Liautard J. P. & Sri Widada J. Effects of profilin-annexin I association on some properties of both profilin and annexin I: modification of the inhibitory activity of profilin on actin polymerization and inhibition of the self-association of annexin I and its interactions with liposomes. Biochim Biophys Acta. 1339, 331–40 (1997). 9187254 10.1016/s0167-4838(97)00018-6

[b17] Hayes M. J., Rescher U., Gerke V. & Moss S. E. Annexin-actin interactions. Traffic. 5, 571–576 (2004). 15260827 10.1111/j.1600-0854.2004.00210.x

[b18] Croxtall J. D. . Lipocortin 1 co-associates with cytokeratins 8 and 18 in A549 cells via the N-terminal domain. Biochim Biophys. 1401, 39–51 (1998). 10.1016/s0167-4889(97)00120-1 9459484

[b19] Belvedere R. . Role of intracellular and extracellular annexin A1 in migration and invasion of human pancreatic carcinoma cells. BMC Cancer. 14, 961 (2014). 25510623 10.1186/1471-2407-14-961 PMC4301448

[b20] Bauer D. E., Canver M. C. & Orkin S. H. Generation of genomic deletions in mammalian cell lines via CRISPR/Cas9. J Vis Exp. (95) (2014). 10.3791/52118 PMC4279820 25549070

[b21] Bizzarro V. . Annexin A1 N-terminal derived peptide Ac2-26 stimulates fibroblast migration in high glucose conditions. PLoS One. 7, e45639 (2012). 23029153 10.1371/journal.pone.0045639 PMC3448638

[b22] Bizzarro V., Belvedere R., Dal Piaz F., Parente L. & Petrella A. Annexin A1 induces skeletal muscle cell migration acting through formyl peptide receptors. PLoS One. 7, e48246 (2012). 23144744 10.1371/journal.pone.0048246 PMC3483218

[b23] Dittmer T. A. & Misteli T. The lamin protein family. Genome Biol. 12, 222 (2011). 21639948 10.1186/gb-2011-12-5-222 PMC3219962

[b24] Sugahara K. N. . Tumor cells enhance their own CD44 cleavage and motility by generating hyaluronan fragments. J Biol Chem. 281, 5861–5868 (2006). 16407205 10.1074/jbc.M506740200

[b25] Sugahara K. N. . Hyaluronan oligosaccharides induce CD44 cleavage and promote cell migration in CD44-expressing tumor cells. J Biol Chem. 278, 32259–32265 (2003). 12801931 10.1074/jbc.M300347200

[b26] Nagano O. & Saya H. Mechanism and biological significance of CD44 cleavage. Cancer Sci. 95, 930–935 (2004). 15596040 10.1111/j.1349-7006.2004.tb03179.x PMC11159127

[b27] Lau A. T. & Chiu J. F. The possible role of cytokeratin 8 in cadmium-induced adaptation and carcinogenesis. Cancer Res. 67, 2107–2113 (2007). 17332340 10.1158/0008-5472.CAN-06-3771

[b28] Ku N. O. & Omary M. B. A disease- and phosphorylation-related nonmechanical function for keratin 8. J Cell Biol. 174, 115–125 (2006). 16818723 10.1083/jcb.200602146 PMC2064169

[b29] Linder S., Havelka A. M., Ueno T. & Shoshan M. C. Determining tumor apoptosis and necrosis in patient serum using cytokeratin 18 as a biomarker. Cancer Lett. 214, 1–9 (2004). 15331168 10.1016/j.canlet.2004.06.032

[b30] Araujo T. G. . Dynamic dialog between cytokeratin 18 and annexin A1 in breast cancer: a transcriptional disequilibrium. Acta Histochem. 116, 1178–1184 (2014). 25028131 10.1016/j.acthis.2014.06.008

[b31] Chung B. M., Rotty J. D. & Coulombe P. A. Networking galore: intermediate filaments and cell migration. Curr Opin Cell Biol. 25, 600–612 (2013). 23886476 10.1016/j.ceb.2013.06.008 PMC3780586

[b32] Yam C. H., Fung T. K. & Poon R. Y. Cyclin A in cell cycle control and cancer. Cell Mol Life Sci. 59, 1317–1326 (2002). 12363035 10.1007/s00018-002-8510-y PMC11337442

[b33] Chan C. L., Wong J. W., Wong C. P., Chan M. K. & Fong W. P. Human antiquitin: structural and functional studies. Chem Biol Interact. 191, 165–70 (2011). 21185811 10.1016/j.cbi.2010.12.019

[b34] Zhang W. & Liu H. T. MAPK signal pathways in the regulation of cell proliferation in mammalian cells. Cell Res. 12, 9–18 (2002). 11942415 10.1038/sj.cr.7290105

[b35] Mebratu Y. & Tesfaigzi Y. How ERK1/2 activation controls cell proliferation and cell death: Is subcellular localization the answer? Cell Cycle. 8, 1168–1175 (2009). 19282669 10.4161/cc.8.8.8147 PMC2728430

[b36] Wang C. . Regulatory mechanisms of annexin-induced chemotherapy resistance in cisplatin resistant lung adenocarcinoma. Asian Pac J Cancer Prev. 15, 3191–3194 (2014). 24815469 10.7314/apjcp.2014.15.7.3191

[b37] Su N. . Increased expression of annexin A1 is correlated with K-ras mutation in colorectal cancer. Tohoku J Exp Med. 222, 243–250 (2010). 21127395 10.1620/tjem.222.243

[b38] Kang H., Ko J. & Jang S. W. The role of annexin A1 in expression of matrix metalloproteinase-9 and invasion of breast cancer cells. Biochem Biophys Res Commun. 423, 188–194 (2012). 22640735 10.1016/j.bbrc.2012.05.114

[b39] Sato Y. . Up-regulated Annexin A1 expression in gastrointestinal cancer is associated with cancer invasion and lymph node metastasis. Exp Ther Med. 2, 239–243 (2011). 22977491 10.3892/etm.2011.210 PMC3440640

[b40] Deer E. L. . Phenotype and genotype of pancreatic cancer cell lines. Pancreas. 39, 425–435 (2010). 20418756 10.1097/MPA.0b013e3181c15963 PMC2860631

[b41] Wade M. High-Throughput Silencing Using the CRISPR-Cas9 System: A Review of the Benefits and Challenges. J Biomol Screen. 20, 1027–1039 (2015). 26001564 10.1177/1087057115587916

[b42] Vidigal J. A. & Ventura A. Rapid and efficient one-step generation of paired gRNA CRISPR-Cas9 libraries. Nat Commun. 6, 8083 (2015). 26278926 10.1038/ncomms9083 PMC4544769

[b43] Günthert U. . A new variant of glycoprotein CD44 confers metastatic potential to rat carcinoma cells. Cell. 65, 13–24 (1991). 1707342 10.1016/0092-8674(91)90403-l

[b44] Yonemura S. . Ezrin/radixin/moesin (ERM) proteins bind to a positively charged amino acid cluster in the juxta-membrane cytoplasmic domain of CD44, CD43, and ICAM-2. J Cell Biol. 140, 885–895 (1998). 9472040 10.1083/jcb.140.4.885 PMC2141743

[b45] Legg J. W. & Isacke C. M. Identification and functional analysis of the ezrin-binding site in the hyaluronan receptor, CD44. Curr Biol. 8, 705–708 (1998). 9637922 10.1016/s0960-9822(98)70277-5

[b46] Thorne R. F., Legg J. W. & Isacke C. M. The role of the CD44 transmembrane and cytoplasmic domains in co-ordinating adhesive and signalling events. J Cell Sci. 117, 373–380 (2004). 14702383 10.1242/jcs.00954

[b47] Lokeshwar V. B., Fregien N. & Bourguignon L. Y. Ankyrin-binding domain of CD44(GP85) is required for the expression of hyaluronic acid-mediated adhesion function. J Cell Biol. 126, 1099–1109 (1994). 7519619 10.1083/jcb.126.4.1099 PMC2120123

[b48] Bennett V. & Healy J. Membrane domains based on ankyrin and spectrin associated with cell-cell interactions. Cold Spring Harb Perspect Biol. 1, a003012 (2009). 20457566 10.1101/cshperspect.a003012 PMC2882121

[b49] Machnicka B., Grochowalska R., Bogusławska D. M. & Sikorski A. F., Lecomte M. C. Spectrin-based skeleton as an actor in cell signaling. Cell Mol Life Sci. 69, 191–201 (2012). 21877118 10.1007/s00018-011-0804-5 PMC3249148

[b50] Burke B. & Stewart C. L. The nuclear lamins: Flexibility in function. Nat Rev Mol Cell Biolb. 14,13–24 (2012). 10.1038/nrm3488 23212477

[b51] Moss S. F. . Decreased and aberrant nuclear lamin expression in gastrointestinal tract neoplasms. Gut. 45, 723–729 (1999). 10517909 10.1136/gut.45.5.723 PMC1727735

[b52] Bouwens L. Cytokeratins and cell differentiation in the pancreas. J Pathol. 184, 234–239 (1998). 9614373 10.1002/(SICI)1096-9896(199803)184:3<234::AID-PATH28>3.0.CO;2-D

[b53] Vallenius T. Actin stress fibre subtypes in mesenchymal-migrating cells. Open Biol. 3, 130001 (2013). 23782578 10.1098/rsob.130001 PMC3718327

[b54] Alldridge L. C. & Bryant C. E. Annexin 1 regulates cell proliferation by disruption of cell morphology and inhibition of cyclin D1 expression through sustained activation of the ERK1/2 MAPK signal. Exp Cell Res. 290, 93–107 (2003). 14516791 10.1016/s0014-4827(03)00310-0

[b55] Böttger K., Hatzikirou H., Chauviere A. & Deutsch A. Investigation of the Migration/Proliferation Dichotomy and its Impact on Avascular Glioma Invasion. Math Model Nat Phenom. 1, 105–135 (2012).

[b56] Giese A. . Dichotomy of astrocytoma migration and proliferation. Int J Cancer. 67, 275–282 (1996). 8760599 10.1002/(SICI)1097-0215(19960717)67:2<275::AID-IJC20>3.0.CO;2-9

[b57] Massagué J. & Obenauf A. C. Metastatic colonization by circulating tumour cells. Nature. 529, 298–306 (2016). 26791720 10.1038/nature17038 PMC5029466

[b58] Nielsen M. F., Mortensen M. B. & Detlefsen S. Key players in pancreatic cancer-stroma interaction: Cancer-associated fibroblasts, endothelial and inflammatory cells. World J Gastroenterol. 22, 2678–2700 (2016). 26973408 10.3748/wjg.v22.i9.2678 PMC4777992

[b59] Liotta L. A. & Kohn E. C. The microenvironment of the tumour-host interface. Nature. 411, 375–379 (2001). 11357145 10.1038/35077241

[b60] Yi M. & Schnitzer J. E. Impaired tumor growth, metastasis, angiogenesis and wound healing in annexin A1-null mice. Proc Natl Acad Sci USA. 106, 17886–17891 (2009). 19805119 10.1073/pnas.0901324106 PMC2764877

[b61] Geary L. A. . CAF-secreted annexin A1 induces prostate cancer cells to gain stem cell like features. Mol Cancer Res 12, 607–621 (2014). 24464914 10.1158/1541-7786.MCR-13-0469 PMC3989391

[b62] de Graauw M. . Annexin A1 regulates TGF-beta signaling and promotes metastasis formation of basal-like breast cancer cells. Proc Natl Acad Sci USA. 107, 6340–6345 (2010). 20308542 10.1073/pnas.0913360107 PMC2852023

[b63] Boudhraa Z., Bouchon B., Viallard C., D’Incan M. & Degoul F. Annexin A1 localization and its relevance to cancer. Clin Sci. (Lond). 130, 205–220 (2016). 26769657 10.1042/CS20150415

[b64] Kim M. P., Evans D. B., Wang H., Abbruzzese J. L., Fleming J. B. & Gallick G. E. Generation of orthotopic and heterotopic human pancreatic cancer xenografts in immunodeficient mice. Nat Protoc. 4, 1670–1680 (2009). 19876027 10.1038/nprot.2009.171 PMC4203372

