# Supplementary material for: Annexin A1 contributes to pancreatic cancer cell phenotype, behaviour and metastatic potential independently of Formyl Peptide Receptor pathway
Source: Sci Rep. 2016 Jul 14;6:29660. doi: 10.1038/srep29660 (PMC4944142; doi:10.1038/srep29660)
Supplement: Supplementary Information [file srep29660-s1.pdf]

SUPPLEMENTARY INFORMATION

Annexin A1 contributes to pancreatic cancer cell phenotype, behaviour and metastatic potential independently of Formyl Peptide Receptor pathway.

Raffaella Belvedere, Valentina Bizzarro, Giovanni Forte, Fabrizio Dal Piaz, Luca Parente & Antonello Petrella *.

Department of Pharmacy, University of Salerno, via Giovanni Paolo II 132, 84084 Fisciano (SA) Italy.

*** Corresponding Author:**

Antonello Petrella, Associate Professor, Department of Pharmacy, University of Salerno, Via Giovanni Paolo II, 132 - 84084 Fisciano (Salerno), Italy.

Phone: +39 089 969762; Fax: +39 089 969602; e-mail: apetrella@unisa.it

Supplementary tables from S1 to S5.

All the dysregulated proteins revealed by LC-MS/MS in ANXA1 KO MIA PaCa-2 grouped according to the pathways in which they are involved.

Table S1: Proteins involved in cell proliferation and identified as differentially expressed in ANXA1 KO MIA PaCa-2 cells by LC-MS/MS

Cell Proliferation					
PROTEIN ID[a]	GENE NAME	PROTEIN NAME[b]	FOLD CHANGE[c]	BIOLOGICAL FUNCTION[d]	p VALUE
B5MCX3	SEPT2	Septin-2	0	Cell division	0.019908
Q08945	SSRP1	FACT complex subunit SSRP1	0	Chromatin remodeling	0.020796
P13010	XRCC5	X-ray repair cross-complementing protein 5	2.301589853	Cell proliferation	0.038707
P23396	RPS3	40S ribosomal protein S3	2.127076489	Cell division	0.008957
Q8N163	KIAA1967	DBIRD complex subunit KIAA1967	0.431186	Cell cycle	0.025403
P12270	TPR	Nucleoprotein TPR	0.517343	Cell division	0.006167
P55072	VCP	Transitional endoplasmic reticulum ATPase	0.614778	Mitosis-associated vesiculation	0.010769

a UniProtKB Accession Numbers;
b Protein acrostic names according UniProtKB;
c Average ratio between the ANXA1 KO and PGS MIA PaCa-2 cells are reported;
d Protein function according to UniProtKB;

[orange box] = upregulated proteins [pale yellow box] = downregulated proteins

Table S2: Proteins involved in cell trafficking and identified as differentially expressed in ANXA1 KO MIA PaCa-2 cells by LC-MS/MS

PROTEIN ID[a]	GENE NAME	PROTEIN NAME[b]	FOLD CHANGE[c]	BIOLOGICAL FUNCTION[d]	p VALUE
Q15758	SLC1A5	Neutral amino acid transporter B(0)	6.064931411	Amino acid transport	0.046059
Q9UBS4	DNAJB11	DnaJ homolog subfamily B member 11	0	Co-chaperone	0.007254
P27797	CALR	Calreticulin	0.566725	Calcium binding chaperone	0.024343
Q09666	AHNAK	Neuroblast differentiation-associated protein AHNAK	0.604693	Protein oligomerization	0.000977

a UniProtKB Accession Numbers;
b Protein acrostic names according UniProtKB;
c Average ratio between the ANXA1 KO and PGS MIA PaCa-2 cells are reported;
d Protein function according to UniProtKB;

[orange box] = upregulated proteins [pale yellow box] = downregulated proteins

Table S3: Proteins involved in metabolic processes and identified as differentially expressed in ANXA1 KO MIA PaCa-2 cells by LC-MS/MS

PROTEIN ID[a]	GENE NAME	PROTEIN NAME[b]	FOLD CHANGE[c]	BIOLOGICAL FUNCTION[d]	p VALUE
P19623	SRM	Spermidine synthase	0	Polyamine metabolic process	0.005564
P21980	TGM2	Protein-glutamine gamma-glutamyltransferase 2	0	Catalytic activity; cell adhesion	0.016302
P23381	WARS	Tryptophan--tRNA ligase	0	Catalytic activity; anti-angiogenic activity	0.020087
P02792	FTL	Ferritin light chain	1.07203065	Cellular iron ion homeostasis	0.009598
B4DLR8	NQO1	NAD(P)H dehydrogenase [quinone] 1	3.86739188	NAD(P)H dehydrogenase	3.95E-05
Q9H845	ACAD9	Acyl-CoA dehydrogenase family member 9, mitochondrial	3.02919013	Lipid homeostasis	0.040886
P49419-2	ALDH7A1	Alpha-aminoadipic semialdehyde dehydrogenase	2.506037792	Cellular aldehyde metabolic process	0.001015
P15880	RPS2	40S ribosomal protein S2	2.335978055	Cellular protein metabolic process	0.0118
P62424	RPL7A	60S ribosomal protein L7a	2.227868741	Cellular protein metabolic process	0.040631
P14618	PKM	Pyruvate kinase	1.826774384	Glycolysis	0.013222
P30041	PRDX6	Peroxiredoxin-6	1.637017035	Antioxidant activity	0.029797
P23526	AHCY	Adenosylhomocysteinase	1.604107092	Catalytic activity	0.041839
P00352	ALDH1A1	Retinal dehydrogenase 1	1.509394815	Retinol metabolism	0.026243
P16615-5	ATP2A2	Sarcoplasmic/endoplasmic reticulum calcium ATPase 2	0	ATPase activity	0.02673
E9PKD5	PSMC3	26S protease regulatory subunit 6A	0.14125	Proteasome activity	0.034945
A0A087X054	HYOU1	Hypoxia up-regulated protein 1	0.316505	ATP-binding	9.27E-06
P11021	HSPA5	78 kDa glucose-regulated protein	0.340958	Protein folding and degradation	5.03E-06
P07237	P4HB	Protein disulfide-isomerase	0.410515	Catalytic activity	0.004959
P30101	PDIA3	Protein disulfide-isomerase A3	0.631386	Catalytic activity	0.005858

a UniProtKB Accession Numbers;
b Protein acrostic names according UniProtKB;
c Average ratio between the ANXA1 KO and PGS MIA PaCa-2 cells are reported;
d Protein function according to UniProtKB;

= upregulated proteins (rows P19623–P00352) = downregulated proteins (rows P16615-5–P30101)

Table S4: Proteins involved in cytoskeletal organization and identified as differentially expressed in ANXA1 KO MIA PaCa-2 cells by LC-MS/MS

Cytoskeletal organization					
PROTEIN ID[a]	GENE NAME	PROTEIN NAME[b]	FOLD CHANGE[c]	BIOLOGICAL FUNCTION[d]	p VALUE
P53621	COPA	Coatomer subunit alpha;Xenin;Proxenin	7.740548847	Structural molecule activity	0.013284
P48643-2	CCT5	T-complex protein 1 subunit epsilon	3.47838083	Microtubule organization	0.035743
O75369-2	FLNB	Filamin-B	1.579505107	Connection of cell membrane constituents to the actin cytoskeleton	0.000111
P43243	MATR3	Matrin-3	0.303521	Structural molecule activity	0.017219
P14625	HSP90B1	Endoplasmin	0.390034	Actin rod assembly	0.000784
P12814-2	ACTN1	Alpha-actinin-1	0.390837	Actin filament organization	0.002918
Q13263	TRIM28	Transcription intermediary factor 1-beta	0.561538	Nucleosome remodeling	0.044216

a UniProtKB Accession Numbers;
b Protein acrostic names according UniProtKB;
c Average ratio between the ANXA1 KO and PGS MIA PaCa-2 cells are reported;
d Protein function according to UniProtKB;

(orange) = upregulated proteins (light yellow) = downregulated proteins

Table S5: Proteins involved in other processes and identified as differentially expressed in ANXA1 KO MIA PaCa-2 cells by LC-MS/MS

Other Processes					
PROTEIN ID[a]	GENE NAME	PROTEIN NAME[b]	FOLD CHANGE[c]	BIOLOGICAL FUNCTION[d]	p VALUE
O43390	HNRNPR	Heterogeneous nuclear ribonucleoprotein R	0	mRNA processing, mRNA splicing	0.023029
P31942	HNRNPH3	Heterogeneous nuclear ribonucleoprotein H3	0	mRNA processing; cell differentiation	0.023057
O95433	AHSA1	Activator of HSP90 ATPase homolog 1	6.624361944	Stress response	0.026754
E7ER77	ERMP1	Endoplasmic reticulum metallopeptidase 1	4.973783186	Metallopeptidase activity	0.00342
E9PKG1	PRMT1	Protein arginine N-methyltransferase 1	4.756975455	Regulation of transcription	0.002524
P17987	TCP1	T-complex protein 1 subunit alpha	4.710964973	Protein folding	0.002887
P62136	PPP1CA	Serine/threonine-protein phosphatase PP1-alpha catalytic subunit	4.398599871	Phosphatase activity	0.018955
P78527-2	PRKDC	DNA-dependent protein kinase catalytic subunit	2.414339969	Serine/threonine-protein kinase; Cell lineage; Tissue development; Apoptosis	0.000102
P12956	XRCC6	X-ray repair cross-complementing protein 6	1.647574521	DNA repair	0.041851
Q13838	DDX39B	Spliceosome RNA helicase DDX39B	1.492748057	DNA repair	0.049003
C9J9K3	RPSA	40S ribosomal protein SA	1.237329158	Structural constituent of ribosome	0.024141
Q9Y4W6	AFG3L2	AFG3-like protein 2	0	Metalloprotease	0.013201
P35637-2	FUS	RNA-binding protein FUS	0	Cellular response to calcium ion	0.008668
P08621-3	SNRNP70	U1 small nuclear ribonucleoprotein 70 kDa	0	mRNA processing	0.037021
P13667	PDIA4	Protein disulfide-isomerase A4	0.189525	Protein folding	7.31E-08
P09874	PARP1	Poly [ADP-ribose] polymerase 1	0.436174	DNA repair	0.03311
Q92598-2	HSPH1	Heat shock protein 105 kDa	0.515871	Stress response	0.041804

a UniProtKB Accession Numbers;
b Protein acrostic names according UniProtKB;
c Average ratio between the ANXA1 KO and PGS MIA PaCa-2 cells are reported;
d Protein function according to UniProtKB;

= upregulated proteins (orange) **= downregulated proteins** (light yellow)

Supplementary Tables S6.

Raw data concerning all the dysregulated proteins revealed by LC-MS/MS in ANXA1 KO MIA PaCa-2.

TABLE S6: Mass Spectrometry Proteomics Raw Data

Protein Ids	KO I	KO II	KO III	KO IV	KO V	KO VI	KO VII	KO VIII	PGS I	PGS II	PGS III	PGS IV	PGS V	PGS VI	PGS VII	PGS VIII	Mean	Mean KO	Mean PGS	SD	SD KO	SD PGS	p value
P13667	7.63E+07	2.88E+07	4.24E+07	8.31E+07	5.29E+07	6.84E+07	0.00E+00	4.75E+07	2.93E+08	2.43E+08	2.31E+08	3.32E+08	2.19E+08	2.90E+08	1.72E+08	2.31E+08	1.51E+08	4.99E+07	2.51E+08	1.11E+08	2.71E+07	5.08E+07	1.06E-07
P05787;P05787-2	2.00E+07	3.34E+07	2.57E+07	3.38E+07	4.36E+07	1.80E+07	1.69E+07	2.12E+07	0.00E+00	0.00E+00	0.00E+00	0.00E+00	0.00E+00	0.00E+00	0.00E+00	0.00E+00	1.33E+07	2.66E+07	0.00E+00	1.52E+07	9.47E+06	0.00E+00	1.50E-06
P11021	6.34E+08	4.97E+08	5.92E+08	8.21E+08	7.61E+08	5.80E+08	3.74E+07	4.55E+08	1.92E+09	1.45E+09	1.47E+09	2.14E+09	1.56E+09	1.79E+09	1.18E+09	1.20E+09	1.07E+09	5.47E+08	1.59E+09	6.08E+08	2.40E+08	3.41E+08	5.64E-06
A0A087X054;Q9Y4L1;E9PJ21;A0A087WWI4;K7EQK2;Q9Y4L1-2	8.15E+07	7.85E+07	6.61E+07	3.67E+07	7.25E+07	0.00E+00	5.36E+07	7.08E+07	1.76E+08	1.43E+08	1.08E+08	2.43E+08	1.74E+08	2.28E+08	2.00E+08	1.61E+08	1.18E+08	5.75E+07	1.79E+08	7.21E+07	2.74E+07	4.42E+07	1.16E-05
B4DLR8;P15559-3;P15559-2;H3BNV2;H3BRK3;P15559	3.35E+07	4.95E+07	5.06E+07	3.50E+07	4.36E+07	2.81E+07	3.50E+07	3.46E+07	0.00E+00	0.00E+00	0.00E+00	1.92E+07	2.64E+07	1.72E+07	0.00E+00	1.74E+07	2.44E+07	3.87E+07	1.00E+07	1.76E+07	8.16E+06	1.11E+07	3.89E-05
P04083;Q5T3N1	1.66E+08	1.78E+08	1.37E+08	1.71E+08	1.92E+08	2.12E+08	2.22E+08	1.83E+08	1.31E+08	1.33E+08	1.31E+08	1.01E+08	1.06E+08	1.20E+08	7.03E+07	1.35E+08	1.49E+08	1.83E+08	1.16E+08	4.20E+07	2.67E+07	2.26E+07	9.36E-05
O75369-2;O75369-9;O75369;O75369-8;O75369-6;O75369-3;E7EN95;O75369-7;O75369-5;O75369-4	1.68E+08	1.35E+08	1.67E+08	1.37E+08	1.70E+08	1.45E+08	1.95E+08	2.13E+08	5.23E+07	4.55E+07	7.01E+07	2.73E+07	1.35E+08	3.89E+07	4.48E+07	1.37E+08	1.18E+08	1.66E+08	6.88E+07	6.12E+07	2.75E+07	4.31E+07	9.56E-05
P78527-2;P78527	2.59E+07	1.08E+07	1.66E+07	2.50E+07	1.48E+07	1.72E+07	0.00E+00	2.57E+07	0.00E+00	0.00E+00	1.40E+07	0.00E+00	0.00E+00	0.00E+00	0.00E+00	0.00E+00	9.37E+06	1.70E+07	1.75E+06	1.05E+07	8.87E+06	4.94E+06	8.11E-04
E7ER77;Q7Z2K6	1.23E+08	3.84E+08	5.17E+08	3.69E+08	2.03E+08	5.19E+08	3.90E+08	4.59E+08	5.68E+08	6.66E+08	6.88E+08	7.16E+08	6.60E+08	5.17E+08	4.96E+08	6.09E+08	4.93E+08	3.70E+08	6.15E+08	1.69E+08	1.42E+08	8.11E+07	8.46E-04
Q09666	1.26E+08	4.86E+08	3.23E+08	8.42E+08	6.11E+08	0.00E+00	6.69E+08	4.60E+08	1.37E+09	9.44E+08	9.31E+08	1.93E+09	1.04E+09	1.55E+09	8.05E+08	8.25E+08	8.06E+08	4.39E+08	1.17E+09	5.06E+08	2.81E+08	4.04E+08	8.51E-04
P14625	6.69E+07	5.69E+07	6.92E+07	9.79E+07	1.19E+08	6.51E+07	0.00E+00	1.06E+08	1.44E+08	1.36E+08	1.70E+08	1.59E+08	9.66E+07	1.21E+08	1.29E+08	1.37E+08	1.05E+08	7.26E+07	1.37E+08	4.43E+07	3.69E+07	2.25E+07	9.07E-04
P02545;P02545-3;P02545-6;P02545-5;P02545-4	1.53E+08	9.29E+07	1.70E+08	1.92E+08	1.25E+08	1.34E+08	6.68E+07	1.27E+08	1.12E+08	6.07E+07	0.00E+00	9.96E+07	5.30E+07	2.80E+07	3.69E+07	3.45E+07	9.29E+07	1.33E+08	5.31E+07	5.56E+07	4.03E+07	3.74E+07	1.11E-03
P49419-2;P49419;F8VS02;P49419-4;P49419-3	1.75E+07	1.12E+07	1.01E+07	1.45E+07	1.53E+07	9.98E+06	1.62E+07	0.00E+00	0.00E+00	9.55E+06	0.00E+00	0.00E+00	0.00E+00	1.04E+07	0.00E+00	0.00E+00	7.16E+06	1.18E+07	2.49E+06	6.90E+06	5.56E+06	4.61E+06	2.59E-03
E9PKG1;Q99873-3;Q99873-2;Q99873-4;Q99873;H7C2I1;E9PIX6;E9PQ98;H0YDE4;A0A087X1W2	1.23E+07	1.29E+07	1.56E+07	1.46E+07	1.91E+07	2.19E+07	0.00E+00	1.84E+07	0.00E+00	1.53E+07	0.00E+00	0.00E+00	0.00E+00	0.00E+00	0.00E+00	9.08E+06	8.69E+06	1.43E+07	3.04E+06	8.41E+06	6.65E+06	5.87E+06	2.88E-03
P17987;E7EQR6;F5H282;E7ERF2	0.00E+00	3.47E+07	0.00E+00	2.44E+07	2.12E+07	0.00E+00	2.87E+07	2.76E+07	2.78E+07	3.02E+07	5.60E+07	4.93E+07	3.07E+07	3.49E+07	3.36E+07	6.02E+07	2.87E+07	1.71E+07	4.03E+07	1.79E+07	1.47E+07	1.28E+07	4.48E-03
P12814-2;P12814;P12814-3;P12814-4;H9KV75	6.92E+07	3.26E+07	1.36E+08	1.71E+08	1.05E+08	3.42E+07	8.50E+07	3.26E+07	2.63E+08	2.43E+08	2.30E+08	2.95E+08	2.30E+08	3.85E+07	1.30E+08	1.69E+08	1.42E+08	8.32E+07	2.00E+08	9.01E+07	5.17E+07	8.33E+07	4.60E-03
P07237;H7BZ94;F5H8J2;I3L312;I3L398	4.04E+06	0.00E+00	3.95E+06	0.00E+00	5.89E+06	5.08E+06	0.00E+00	6.31E+06	0.00E+00	0.00E+00	0.00E+00	0.00E+00	0.00E+00	0.00E+00	0.00E+00	0.00E+00	1.58E+06	3.16E+06	0.00E+00	2.48E+06	2.74E+06	0.00E+00	5.64E-03
P19623	8.67E+07	7.04E+07	8.24E+07	8.46E+07	6.40E+07	1.30E+08	7.86E+07	7.41E+07	1.69E+08	1.39E+08	1.16E+08	1.80E+08	1.41E+08	5.82E+07	1.29E+08	1.23E+08	1.08E+08	8.39E+07	1.32E+08	3.79E+07	2.03E+07	3.69E+07	6.27E-03
P30101	2.74E+07	3.15E+07	3.01E+07	2.18E+07	1.66E+07	2.17E+07	3.54E+07	3.18E+07	6.64E+07	3.80E+07	5.05E+07	7.16E+07	2.45E+07	7.95E+07	2.48E+07	6.17E+07	3.96E+07	2.70E+07	5.21E+07	1.99E+07	6.40E+06	2.12E+07	6.33E-03
P12270;P12270-2	7.68E+08	1.06E+09	7.71E+08	1.18E+09	1.21E+09	7.99E+08	1.25E+09	5.45E+08	1.45E+09	1.53E+09	2.03E+09	1.59E+09	1.22E+09	8.89E+08	1.29E+09	1.28E+09	1.18E+09	9.48E+08	1.41E+09	3.74E+08	2.60E+08	3.32E+08	7.86E-03
P08670;B0YJC4;B0YJC5	6.36E+07	5.68E+07	7.30E+07	3.88E+07	4.49E+07	5.71E+07	5.23E+07	3.41E+07	0.00E+00	0.00E+00	0.00E+00	2.23E+07	5.28E+07	2.92E+07	5.50E+07	3.33E+07	3.83E+07	5.26E+07	2.41E+07	2.32E+07	1.29E+07	2.28E+07	8.27E-03
P23396;E9PPU1;P23396-2;E9PL09;F2Z2S8;H0YCJ7;H0YEU2;H0YF32	0.00E+00	0.00E+00	0.00E+00	0.00E+00	0.00E+00	0.00E+00	0.00E+00	0.00E+00	0.00E+00	1.11E+07	1.78E+07	0.00E+00	9.15E+06	0.00E+00	7.58E+06	1.55E+07	3.82E+06	0.00E+00	7.64E+06	6.26E+06	0.00E+00	7.12E+06	8.85E-03

P35637-2;P35637;H3BPE7	0.00E+00	0.00E+00	0.00E+00	0.00E+00	0.00E+00	0.00E+00	0.00E+00	0.00E+00	0.00E+00	1.89E+07	0.00E+00	1.99E+07	1.34E+07	0.00E+00	8.96E+06	9.01E+06	4.39E+06	0.00E+00	8.77E+06	7.24E+06	0.00E+00	8.27E+06	9.53E-03
Q9UBS4	1.03E+07	0.00E+00	9.28E+06	1.56E+07	1.30E+07	8.00E+06	0.00E+00	2.46E+07	0.00E+00	8.04E+06	0.00E+00	0.00E+00	0.00E+00	0.00E+00	0.00E+00	0.00E+00	5.56E+06	1.01E+07	1.00E+06	7.51E+06	8.09E+06	2.84E+06	9.54E-03
P02792;A0A087X1B9	2.30E+07	3.71E+07	3.68E+07	3.46E+07	3.53E+07	3.95E+07	3.42E+07	2.03E+07	0.00E+00	0.00E+00	0.00E+00	1.89E+07	4.53E+07	2.04E+07	0.00E+00	2.74E+07	2.33E+07	3.26E+07	1.40E+07	1.58E+07	7.00E+06	1.69E+07	1.22E-02
P15880;H0YEN5;E9PQD7;E9PPT0;E9PMM9	2.87E+08	4.28E+08	5.26E+08	3.20E+08	4.24E+08	2.16E+08	1.74E+08	3.48E+08	9.86E+07	2.67E+08	2.88E+08	6.89E+07	1.93E+08	5.35E+07	2.90E+08	2.30E+08	2.63E+08	3.41E+08	1.86E+08	1.32E+08	1.17E+08	9.91E+07	1.29E-02
P14618;P14618-2;P14618-3;B4DNK4;H3BTN5;H3BQ34;H3BR70	2.35E+07	0.00E+00	2.75E+07	1.88E+07	0.00E+00	8.76E+06	0.00E+00	1.03E+07	0.00E+00	0.00E+00	0.00E+00	0.00E+00	0.00E+00	0.00E+00	0.00E+00	0.00E+00	5.56E+06	1.11E+07	0.00E+00	9.49E+06	1.11E+07	0.00E+00	1.31E-02
Q9Y4W6	9.88E+06	2.28E+07	5.68E+06	0.00E+00	1.60E+07	0.00E+00	8.76E+06	1.07E+07	0.00E+00	5.72E+06	0.00E+00	0.00E+00	3.74E+06	0.00E+00	0.00E+00	0.00E+00	5.21E+06	9.23E+06	1.18E+06	6.88E+06	7.71E+06	2.25E+06	1.33E-02
P53621;P53621-2	9.63E+07	6.65E+07	3.67E+07	7.72E+07	5.65E+07	0.00E+00	1.28E+08	9.97E+07	1.19E+08	1.01E+08	8.88E+07	1.42E+08	1.05E+08	1.07E+08	1.21E+08	1.12E+08	9.11E+07	7.01E+07	1.12E+08	3.65E+07	3.99E+07	1.59E+07	1.52E-02
P55072	4.40E+07	5.21E+07	7.31E+07	2.21E+07	0.00E+00	2.11E+07	0.00E+00	0.00E+00	0.00E+00	0.00E+00	0.00E+00	0.00E+00	0.00E+00	0.00E+00	0.00E+00	0.00E+00	1.33E+07	2.66E+07	0.00E+00	2.33E+07	2.75E+07	0.00E+00	1.62E-02
P21980	0.00E+00	0.00E+00	0.00E+00	0.00E+00	0.00E+00	0.00E+00	2.26E+07	1.78E+07	2.44E+07	1.64E+07	1.22E+07	1.93E+07	5.57E+06	1.21E+07	1.29E+07	2.91E+07	1.08E+07	5.05E+06	1.65E+07	1.02E+07	9.44E+06	7.55E+06	1.82E-02
P43243;A8MXP9;D6REM6;D6R991	7.45E+06	0.00E+00	9.22E+06	0.00E+00	4.85E+06	4.98E+06	8.06E+06	5.82E+06	0.00E+00	0.00E+00	0.00E+00	3.07E+06	6.20E+06	0.00E+00	0.00E+00	0.00E+00	3.10E+06	5.05E+06	1.16E+06	3.48E+06	3.46E+06	2.30E+06	1.92E-02
P62136;P62136-2;F8VYE8;P36873;F8W0W8;P36873-2;E9PMD7;F8VR82;P62136-3;E7ETD8	6.23E+07	6.62E+07	5.79E+07	5.83E+07	6.18E+07	5.78E+07	5.36E+07	7.55E+07	6.47E+07	4.40E+07	5.45E+07	4.77E+07	3.68E+07	3.35E+07	5.74E+07	5.85E+07	5.57E+07	6.17E+07	4.96E+07	1.08E+07	6.73E+06	1.10E+07	1.95E-02
C9J9K3;P08865;A6NE09	6.93E+06	7.33E+06	9.37E+06	0.00E+00	0.00E+00	0.00E+00	6.98E+06	0.00E+00	0.00E+00	0.00E+00	0.00E+00	0.00E+00	0.00E+00	0.00E+00	0.00E+00	0.00E+00	1.91E+06	3.83E+06	0.00E+00	3.46E+06	4.16E+06	0.00E+00	2.09E-02
Q08945	3.94E+06	0.00E+00	4.18E+06	0.00E+00	3.86E+06	2.44E+06	0.00E+00	0.00E+00	0.00E+00	0.00E+00	0.00E+00	0.00E+00	0.00E+00	0.00E+00	0.00E+00	0.00E+00	9.01E+05	1.80E+06	0.00E+00	1.65E+06	1.99E+06	0.00E+00	2.29E-02
O43390;O43390-3;O43390-2;B4DT28;O43390-4	1.87E+06	0.00E+00	0.00E+00	3.35E+06	3.01E+06	0.00E+00	0.00E+00	3.02E+06	0.00E+00	0.00E+00	0.00E+00	0.00E+00	0.00E+00	0.00E+00	0.00E+00	0.00E+00	7.04E+05	1.41E+06	0.00E+00	1.29E+06	1.56E+06	0.00E+00	2.33E-02
P31942-3;P31942-2;P31942;P31942-6;P31942-5;P31942-4	1.24E+08	6.04E+07	1.32E+08	1.21E+08	0.00E+00	9.13E+07	5.20E+07	4.02E+07	3.93E+07	3.20E+07	3.50E+07	0.00E+00	0.00E+00	0.00E+00	8.80E+07	2.73E+07	5.26E+07	7.76E+07	2.77E+07	4.59E+07	4.71E+07	2.96E+07	2.38E-02
H0YD13;H0Y2P0;P16070-18;P16070-12;P16070-14;P16070-13;P16070-11;P16070-10;P16070-16;P16070-8;P16070-17;P16070-6;P16070-4;P16070-3;P16070-7;P16070-5;P16070;H0YE40	1.49E+08	2.36E+08	2.29E+08	1.21E+08	1.94E+08	1.36E+08	1.60E+08	2.06E+08	9.91E+07	1.19E+08	1.42E+08	1.50E+08	1.76E+08	0.00E+00	1.32E+08	1.30E+08	1.49E+08	1.79E+08	1.19E+08	5.61E+07	4.33E+07	5.30E+07	2.61E-02
P00352	1.21E+08	8.04E+07	6.99E+07	4.54E+07	4.22E+07	9.12E+07	1.03E+08	3.12E+07	1.08E+08	1.18E+08	1.46E+08	2.04E+08	1.57E+08	1.88E+07	1.36E+08	1.30E+08	1.00E+08	7.30E+07	1.27E+08	5.05E+07	3.17E+07	5.28E+07	2.61E-02
P27797;K7EJB9	8.31E+07	1.11E+08	1.37E+08	4.86E+07	8.67E+07	1.29E+08	1.74E+08	1.40E+08	6.61E+07	8.16E+07	6.30E+07	8.40E+07	6.32E+07	0.00E+00	1.05E+08	9.11E+07	9.15E+07	1.14E+08	6.92E+07	4.16E+07	3.98E+07	3.16E+07	2.65E-02
P30041	0.00E+00	1.12E+07	0.00E+00	0.00E+00	1.03E+07	8.92E+06	1.55E+07	1.38E+07	0.00E+00	0.00E+00	0.00E+00	0.00E+00	0.00E+00	9.03E+06	0.00E+00	0.00E+00	4.30E+06	7.48E+06	1.13E+06	5.94E+06	6.51E+06	3.19E+06	2.67E-02
O95433;G3V438;O95433-2;H0YJ63;H0YJG7	0.00E+00	0.00E+00	0.00E+00	0.00E+00	0.00E+00	0.00E+00	0.00E+00	0.00E+00	4.98E+06	0.00E+00	6.55E+06	9.10E+06	4.81E+06	0.00E+00	0.00E+00	0.00E+00	1.59E+06	0.00E+00	3.18E+06	2.98E+06	0.00E+00	3.64E+06	2.69E-02
P16615-5;P16615-2;P16615-3;P16615;H7C5W9;P16615-4	2.24E+07	1.40E+07	0.00E+00	0.00E+00	0.00E+00	0.00E+00	2.04E+07	2.54E+07	2.92E+07	2.77E+07	0.00E+00	2.45E+07	2.02E+07	3.13E+07	2.68E+07	2.93E+07	1.69E+07	1.03E+07	2.36E+07	1.25E+07	1.14E+07	1.01E+07	2.70E-02
Q8N163;Q8N163-2;H0YB24	1.13E+07	3.15E+07	1.92E+07	1.11E+07	1.48E+07	6.39E+06	0.00E+00	1.16E+07	9.71E+06	1.75E+07	0.00E+00	0.00E+00	0.00E+00	0.00E+00	0.00E+00	0.00E+00	8.32E+06	1.32E+07	3.40E+06	9.31E+06	9.29E+06	6.62E+06	2.87E-02
P48643-2;E7ENZ3;B7ZAR1;E9PCA1;P48643	3.52E+07	5.83E+07	4.06E+07	2.66E+07	5.97E+07	3.44E+07	0.00E+00	3.81E+07	0.00E+00	0.00E+00	1.51E+07	2.42E+07	3.95E+07	0.00E+00	1.30E+07	3.47E+07	2.62E+07	3.66E+07	1.58E+07	1.99E+07	1.88E+07	1.58E+07	3.11E-02

P62424;Q5T8U2;Q5T8U3	4.52E+07	5.46E+07	2.91E+07	4.71E+07	5.70E+07	3.66E+07	4.37E+07	4.23E+07	3.84E+07	2.69E+07	4.80E+07	0.00E+00	4.62E+07	2.23E+07	0.00E+00	3.31E+07	3.57E+07	4.44E+07	2.69E+07	1.69E+07	9.01E+06	1.87E+07	3.14E-02
P23526;P23526-2	0.00E+00	5.03E+07	0.00E+00	1.51E+07	0.00E+00	0.00E+00	8.95E+07	3.81E+07	6.29E+07	7.14E+07	6.76E+07	3.89E+07	3.72E+07	2.94E+07	6.00E+07	7.34E+07	3.96E+07	2.41E+07	5.51E+07	3.00E+07	3.29E+07	1.72E+07	3.33E-02
P09874	0.00E+00	0.00E+00	0.00E+00	0.00E+00	5.28E+06	0.00E+00	0.00E+00	0.00E+00	5.37E+06	5.75E+06	0.00E+00	5.85E+06	0.00E+00	1.21E+07	0.00E+00	8.02E+06	2.65E+06	6.60E+05	4.64E+06	3.86E+06	1.87E+06	4.40E+06	3.36E-02
E9PKD5;E9PM69;R4GNH3;P17980;E9PMD8	0.00E+00	0.00E+00	0.00E+00	0.00E+00	1.18E+07	3.00E+07	3.11E+07	1.65E+07	0.00E+00	0.00E+00	0.00E+00	0.00E+00	0.00E+00	0.00E+00	0.00E+00	0.00E+00	5.59E+06	1.12E+07	0.00E+00	1.09E+07	1.35E+07	0.00E+00	3.49E-02
P23381;P23381-2	0.00E+00	0.00E+00	0.00E+00	0.00E+00	0.00E+00	0.00E+00	0.00E+00	0.00E+00	6.43E+06	0.00E+00	0.00E+00	3.56E+06	0.00E+00	3.88E+06	0.00E+00	2.06E+06	9.95E+05	0.00E+00	1.99E+06	1.96E+06	0.00E+00	2.44E+06	3.66E-02
P08621-3;P08621-2;P08621	8.51E+07	6.36E+07	9.57E+07	2.54E+07	1.92E+07	2.99E+07	3.01E+07	3.44E+07	1.40E+07	4.31E+07	2.69E+07	0.00E+00	0.00E+00	1.32E+07	3.71E+07	3.01E+07	3.42E+07	4.79E+07	2.05E+07	2.70E+07	2.94E+07	1.63E+07	3.72E-02
P13010	7.13E+06	0.00E+00	9.83E+06	1.07E+07	6.45E+06	1.06E+07	0.00E+00	6.69E+06	8.51E+06	0.00E+00	5.02E+06	0.00E+00	0.00E+00	0.00E+00	0.00E+00	3.50E+06	4.28E+06	6.43E+06	2.13E+06	4.31E+06	4.32E+06	3.24E+06	4.09E-02
Q9H845;H0Y8Z9	3.61E+07	4.73E+07	3.12E+07	3.22E+07	3.26E+07	5.43E+07	0.00E+00	4.51E+07	1.28E+07	1.81E+07	1.91E+07	1.29E+07	2.47E+07	2.31E+07	3.07E+07	2.57E+07	2.79E+07	3.48E+07	2.09E+07	1.40E+07	1.64E+07	6.30E+06	4.12E-02
P12956;B1AHC9;P12956-2	0.00E+00	5.08E+07	0.00E+00	2.45E+07	4.34E+07	0.00E+00	4.81E+07	3.15E+07	2.33E+07	5.12E+07	7.71E+07	4.03E+07	6.27E+07	2.75E+07	6.60E+07	3.72E+07	3.65E+07	2.48E+07	4.81E+07	2.34E+07	2.22E+07	1.93E+07	4.12E-02
Q92598-2;Q92598;Q92598-3;Q92598-4;A0A0A0MSM0	0.00E+00	0.00E+00	0.00E+00	1.70E+07	2.25E+07	0.00E+00	4.55E+06	1.36E+07	0.00E+00	0.00E+00	0.00E+00	0.00E+00	0.00E+00	0.00E+00	0.00E+00	0.00E+00	3.60E+06	7.21E+06	0.00E+00	7.27E+06	9.15E+06	0.00E+00	4.27E-02
F8VZY9;P05783	0.00E+00	7.54E+06	0.00E+00	0.00E+00	5.85E+06	0.00E+00	1.27E+07	1.00E+07	2.01E+07	6.86E+06	1.12E+07	8.40E+06	9.88E+06	1.42E+07	0.00E+00	1.59E+07	7.66E+06	4.51E+06	1.08E+07	6.38E+06	5.20E+06	6.13E+06	4.35E-02
Q13813-3;Q13813;Q13813-2	0.00E+00	4.48E+07	2.04E+07	3.47E+07	0.00E+00	0.00E+00	4.29E+07	3.13E+07	4.56E+07	2.87E+07	3.76E+07	5.67E+07	4.18E+07	3.18E+07	2.76E+07	3.99E+07	3.02E+07	2.18E+07	3.87E+07	1.72E+07	1.95E+07	9.66E+06	4.46E-02
Q13263;Q13263-2;M0R0K9	2.62E+07	7.98E+06	1.43E+07	1.19E+07	0.00E+00	0.00E+00	9.95E+06	0.00E+00	0.00E+00	0.00E+00	0.00E+00	5.52E+06	6.11E+06	0.00E+00	0.00E+00	0.00E+00	5.13E+06	8.81E+06	1.45E+06	7.51E+06	9.09E+06	2.70E+06	4.58E-02
Q15758	4.49E+07	3.19E+07	4.35E+07	4.44E+07	4.64E+07	4.39E+07	2.96E+07	3.42E+07	4.00E+07	4.39E+07	1.58E+07	4.38E+07	0.00E+00	2.92E+07	2.77E+07	1.27E+07	3.32E+07	3.99E+07	2.66E+07	1.37E+07	6.76E+06	1.60E+07	4.95E-02
Q13838;Q13838-2;Q5STU3;H0Y400;F6UJC5;O00148;F6WLT2	0.00E+00	0.00E+00	0.00E+00	0.00E+00	0.00E+00	0.00E+00	2.35E+07	1.72E+07	2.40E+07	2.62E+07	3.68E+07	0.00E+00	0.00E+00	0.00E+00	3.36E+07	5.10E+07	1.33E+07	5.08E+06	2.15E+07	1.71E+07	9.56E+06	1.95E+07	5.13E-02
Q15029-2;Q15029;Q15029-3;K7EP67	9.58E+07	5.12E+08	6.04E+08	3.28E+08	3.56E+08	6.98E+07	5.82E+08	4.36E+08	5.40E+08	3.74E+08	3.99E+08	8.16E+08	6.14E+08	6.23E+08	5.21E+08	5.74E+08	4.65E+08	3.73E+08	5.57E+08	1.94E+08	2.04E+08	1.39E+08	5.30E-02
P19338;H7BY16	0.00E+00	3.53E+07	0.00E+00	3.86E+07	2.86E+07	0.00E+00	4.46E+07	0.00E+00	0.00E+00	0.00E+00	0.00E+00	0.00E+00	2.01E+07	0.00E+00	0.00E+00	0.00E+00	1.04E+07	1.84E+07	2.51E+06	1.67E+07	2.01E+07	7.10E+06	5.41E-02
Q2TB90;Q2TB90-2;Q2TB90-3	2.14E+07	3.11E+07	3.14E+07	4.47E+07	4.18E+07	3.71E+07	2.69E+07	2.66E+07	2.23E+07	3.61E+07	2.36E+07	1.97E+07	3.34E+07	0.00E+00	2.52E+07	2.13E+07	2.77E+07	3.26E+07	2.27E+07	1.05E+07	8.01E+06	1.09E+07	5.73E-02
P29692;P29692-3;E9PK01;P29692-2;E9PRY8;A0A087X1X7;E9PMW7;E9PPR1;E9PL12;E9PQ49;E9PI39;E9PQZ1;P29692-4	2.35E+07	8.84E+07	3.34E+07	0.00E+00	9.03E+07	0.00E+00	1.40E+08	1.14E+08	6.42E+07	1.98E+08	1.51E+08	7.17E+07	9.98E+07	6.00E+07	1.18E+08	1.58E+08	8.81E+07	6.12E+07	1.15E+08	5.75E+07	5.38E+07	5.03E+07	5.75E-02
Q00839-2;Q00839	1.91E+07	3.02E+07	0.00E+00	4.74E+07	3.61E+07	0.00E+00	4.22E+07	3.04E+07	3.81E+07	3.47E+07	2.65E+07	7.11E+07	4.68E+07	4.60E+07	3.34E+07	3.94E+07	3.38E+07	2.57E+07	4.20E+07	1.75E+07	1.80E+07	1.35E+07	5.92E-02
P05556;P05556-2;P05556-5;P05556-4;P05556-3	0.00E+00	0.00E+00	6.82E+06	5.54E+06	9.62E+06	7.79E+06	0.00E+00	0.00E+00	0.00E+00	0.00E+00	4.24E+06	0.00E+00	0.00E+00	0.00E+00	0.00E+00	0.00E+00	2.13E+06	3.72E+06	5.30E+05	3.43E+06	4.13E+06	1.50E+06	5.93E-02
Q9NSD9;Q9NSD9-2	2.54E+06	2.23E+06	0.00E+00	0.00E+00	2.55E+06	0.00E+00	0.00E+00	0.00E+00	0.00E+00	0.00E+00	0.00E+00	0.00E+00	0.00E+00	0.00E+00	0.00E+00	0.00E+00	4.58E+05	9.15E+05	0.00E+00	9.86E+05	1.27E+06	0.00E+00	6.03E-02
E7EU96;P68400;Q5U5J2;Q8NEV1	0.00E+00	0.00E+00	0.00E+00	0.00E+00	7.39E+06	0.00E+00	7.46E+06	8.64E+06	0.00E+00	0.00E+00	0.00E+00	0.00E+00	0.00E+00	0.00E+00	0.00E+00	0.00E+00	1.47E+06	2.94E+06	0.00E+00	3.17E+06	4.07E+06	0.00E+00	6.06E-02
B5MCX3;Q15019;Q15019-3;Q15019-2;C9J2Q4;H7C2Y0	0.00E+00	0.00E+00	0.00E+00	0.00E+00	0.00E+00	0.00E+00	0.00E+00	0.00E+00	2.91E+06	3.26E+06	2.59E+06	0.00E+00	0.00E+00	0.00E+00	0.00E+00	0.00E+00	5.48E+05	0.00E+00	1.10E+06	1.18E+06	0.00E+00	1.52E+06	6.12E-02
Q9H2G2-2;Q9H2G2	0.00E+00	0.00E+00	0.00E+00	0.00E+00	0.00E+00	0.00E+00	0.00E+00	0.00E+00	0.00E+00	9.28E+06	0.00E+00	0.00E+00	7.47E+06	0.00E+00	0.00E+00	7.36E+06	1.51E+06	0.00E+00	3.01E+06	3.26E+06	0.00E+00	4.20E+06	6.18E-02
Q13151	0.00E+00	0.00E+00	0.00E+00	0.00E+00	0.00E+00	0.00E+00	0.00E+00	0.00E+00	0.00E+00	0.00E+00	0.00E+00	8.66E+06	1.08E+07	0.00E+00	0.00E+00	8.55E+06	1.75E+06	0.00E+00	3.50E+06	3.79E+06	0.00E+00	4.88E+06	6.18E-02
H0YMZ1;H0YN18;H0YL69;P25789;H0YLC2;H0YKT8;H0YMA1;H0YLS6;P25789-2	1.01E+07	1.17E+07	0.00E+00	0.00E+00	8.79E+06	0.00E+00	0.00E+00	0.00E+00	0.00E+00	0.00E+00	0.00E+00	0.00E+00	0.00E+00	0.00E+00	0.00E+00	0.00E+00	1.91E+06	3.82E+06	0.00E+00	4.14E+06	5.33E+06	0.00E+00	6.20E-02
O14776-2;O14776;G3V220	2.27E+07	0.00E+00	0.00E+00	3.19E+07	2.27E+07	2.56E+07	2.92E+07	2.95E+07	2.17E+07	2.96E+07	3.52E+07	1.22E+08	4.24E+07	0.00E+00	7.15E+07	6.63E+07	3.44E+07	2.02E+07	4.86E+07	3.08E+07	1.29E+07	3.75E+07	6.26E-02

G3V576;P07910-4;G3V4W0;B4DY08;B2R5W2;G3V4C1;P07910-2;G3V2Q1;P07910;G3V5X6;G3V3K6;G3V251;B4DSU6;G3V555;G3V575;P0DMR1;O60812;B7ZW38;B2RXH8;G3V4M8;P07910-3	0.00E+00	0.00E+00	0.00E+00	0.00E+00	0.00E+00	0.00E+00	0.00E+00	7.01E+06	1.48E+07	0.00E+00	0.00E+00	1.29E+07	7.20E+06	0.00E+00	1.50E+07	0.00E+00	3.56E+06	8.76E+05	6.25E+06	5.83E+06	2.48E+06	7.09E+06	6.27E-02
P50213-2;P50213;H0YL72;H0YMU3;H0YLI6	2.99E+06	2.50E+06	3.46E+06	0.00E+00	0.00E+00	0.00E+00	0.00E+00	0.00E+00	0.00E+00	0.00E+00	0.00E+00	0.00E+00	0.00E+00	0.00E+00	0.00E+00	0.00E+00	5.59E+05	1.12E+06	0.00E+00	1.22E+06	1.57E+06	0.00E+00	6.27E-02
P47897-2;P47897;C9JSG9;C9J165	0.00E+00	8.00E+06	0.00E+00	0.00E+00	0.00E+00	0.00E+00	5.77E+06	7.71E+06	0.00E+00	0.00E+00	0.00E+00	0.00E+00	0.00E+00	0.00E+00	0.00E+00	0.00E+00	1.34E+06	2.68E+06	0.00E+00	2.92E+06	3.76E+06	0.00E+00	6.31E-02
D6R9X8;P26006;P26006-1	2.30E+08	5.32E+07	9.60E+07	7.04E+08	6.21E+08	4.34E+08	3.76E+08	4.60E+08	1.27E+09	7.41E+08	1.13E+09	4.35E+08	1.34E+08	1.20E+09	2.99E+08	5.78E+08	5.47E+08	3.72E+08	7.22E+08	3.82E+08	2.33E+08	4.34E+08	6.36E-02
P35527;K7EQQ3	0.00E+00	0.00E+00	0.00E+00	0.00E+00	0.00E+00	0.00E+00	0.00E+00	0.00E+00	0.00E+00	0.00E+00	0.00E+00	3.87E+06	0.00E+00	0.00E+00	2.99E+06	2.59E+06	5.90E+05	0.00E+00	1.18E+06	1.29E+06	0.00E+00	1.67E+06	6.49E-02
P82930;C9JJ19;A0A087WUZ8	0.00E+00	0.00E+00	0.00E+00	0.00E+00	0.00E+00	6.16E+06	3.83E+06	4.71E+06	0.00E+00	0.00E+00	0.00E+00	0.00E+00	0.00E+00	0.00E+00	0.00E+00	0.00E+00	9.19E+05	1.84E+06	0.00E+00	2.02E+06	2.61E+06	0.00E+00	6.66E-02
D6RAA6;P57088;H0Y8N0	2.53E+07	0.00E+00	0.00E+00	0.00E+00	0.00E+00	0.00E+00	2.83E+07	3.41E+07	3.69E+07	2.59E+07	1.89E+07	1.84E+07	0.00E+00	3.55E+07	2.75E+07	3.45E+07	1.78E+07	1.10E+07	2.47E+07	1.52E+07	1.53E+07	1.23E+07	6.76E-02
P78347-2;P78347-4;P78347-3;P78347	2.94E+07	3.26E+07	2.19E+07	9.51E+06	1.39E+07	3.33E+07	2.39E+07	1.55E+07	8.16E+06	1.32E+07	1.68E+07	2.29E+07	1.72E+07	0.00E+00	2.23E+07	1.42E+07	1.84E+07	2.25E+07	1.43E+07	9.03E+06	8.95E+06	7.51E+06	6.84E-02
A6NLN1;P26599;P26599-2;P26599-3	0.00E+00	0.00E+00	0.00E+00	0.00E+00	0.00E+00	6.81E+06	1.08E+07	7.09E+06	0.00E+00	0.00E+00	0.00E+00	0.00E+00	0.00E+00	0.00E+00	0.00E+00	0.00E+00	1.54E+06	3.09E+06	0.00E+00	3.42E+06	4.42E+06	0.00E+00	6.85E-02
P61160;P61160-2;F5H6T1	0.00E+00	0.00E+00	0.00E+00	0.00E+00	0.00E+00	3.69E+06	3.77E+06	6.06E+06	0.00E+00	0.00E+00	0.00E+00	0.00E+00	0.00E+00	0.00E+00	0.00E+00	0.00E+00	8.45E+05	1.69E+06	0.00E+00	1.88E+06	2.44E+06	0.00E+00	7.04E-02
Q8NCW5-2;Q8NCW5	6.04E+06	0.00E+00	7.82E+06	1.88E+07	1.07E+07	1.26E+07	0.00E+00	1.46E+07	7.84E+06	0.00E+00	0.00E+00	0.00E+00	0.00E+00	1.11E+07	0.00E+00	6.68E+06	6.01E+06	8.82E+06	3.20E+06	6.27E+06	6.71E+06	4.59E+06	7.11E-02
Q16698-2;Q16698	4.90E+07	5.07E+07	3.19E+07	0.00E+00	3.10E+07	0.00E+00	3.64E+07	5.09E+07	3.26E+07	3.66E+07	3.94E+07	6.91E+07	4.81E+07	5.46E+07	4.54E+07	5.52E+07	3.94E+07	3.12E+07	4.76E+07	1.85E+07	2.09E+07	1.19E+07	7.45E-02
O75533	1.57E+07	1.59E+07	1.80E+07	0.00E+00	0.00E+00	0.00E+00	1.71E+07	9.03E+06	0.00E+00	1.03E+07	0.00E+00	0.00E+00	0.00E+00	1.22E+07	0.00E+00	0.00E+00	6.14E+06	9.46E+06	2.82E+06	7.52E+06	8.28E+06	5.24E+06	7.58E-02
Q92973-2;Q92973;Q92973-3	1.28E+07	2.49E+07	0.00E+00	0.00E+00	1.34E+07	4.62E+06	0.00E+00	7.93E+06	0.00E+00	0.00E+00	0.00E+00	0.00E+00	0.00E+00	5.92E+06	3.93E+06	4.43E+06	4.87E+06	7.95E+06	1.78E+06	7.01E+06	8.79E+06	2.52E+06	7.72E-02
P16403;P16402	0.00E+00	8.94E+06	0.00E+00	0.00E+00	1.01E+07	0.00E+00	4.44E+06	0.00E+00	0.00E+00	0.00E+00	0.00E+00	0.00E+00	0.00E+00	0.00E+00	0.00E+00	0.00E+00	1.47E+06	2.93E+06	0.00E+00	3.33E+06	4.35E+06	0.00E+00	7.72E-02
I3L2B0;K7EIG1;O75153	0.00E+00	0.00E+00	0.00E+00	9.70E+06	3.71E+07	0.00E+00	1.07E+07	1.04E+07	0.00E+00	0.00E+00	0.00E+00	0.00E+00	0.00E+00	0.00E+00	0.00E+00	0.00E+00	4.24E+06	8.47E+06	0.00E+00	9.66E+06	1.26E+07	0.00E+00	7.82E-02
P43490	4.68E+07	2.69E+07	8.33E+07	6.91E+07	0.00E+00	6.70E+07	0.00E+00	5.15E+07	3.02E+07	0.00E+00	3.22E+07	0.00E+00	0.00E+00	2.17E+07	2.65E+07	4.25E+07	3.11E+07	4.31E+07	1.91E+07	2.73E+07	3.14E+07	1.69E+07	7.89E-02
P14314-2;P14314;K7ELL7	9.43E+06	7.59E+06	1.65E+07	0.00E+00	0.00E+00	0.00E+00	0.00E+00	0.00E+00	0.00E+00	0.00E+00	0.00E+00	0.00E+00	0.00E+00	0.00E+00	0.00E+00	0.00E+00	2.10E+06	4.19E+06	0.00E+00	4.82E+06	6.31E+06	0.00E+00	8.12E-02
P33992;B1AHB1	0.00E+00	1.03E+07	0.00E+00	0.00E+00	0.00E+00	0.00E+00	1.13E+07	1.00E+07	1.06E+07	0.00E+00	1.31E+07	1.27E+07	0.00E+00	1.23E+07	1.17E+07	1.34E+07	6.59E+06	3.96E+06	9.21E+06	6.07E+06	5.47E+06	5.75E+06	8.21E-02
P33991;E5RG31	6.30E+07	6.04E+07	6.52E+07	4.76E+07	6.36E+07	0.00E+00	3.63E+07	5.02E+07	7.53E+07	2.99E+07	4.54E+07	1.13E+08	7.17E+07	9.11E+07	7.36E+07	6.37E+07	5.94E+07	4.83E+07	7.04E+07	2.57E+07	2.19E+07	2.56E+07	8.40E-02
Q86UP2-2;Q86UP2-4;Q86UP2;Q86UP2-3;G3V4Y7	0.00E+00	5.21E+07	0.00E+00	8.14E+07	6.97E+07	1.09E+07	1.02E+08	9.50E+07	1.32E+08	6.25E+07	6.05E+07	9.89E+07	7.56E+07	1.07E+08	7.77E+07	5.84E+07	6.77E+07	5.13E+07	8.41E+07	3.81E+07	4.24E+07	2.64E+07	8.46E-02
Q14697;Q14697-2;F5H6X6;E9PKU7	9.12E+06	0.00E+00	1.45E+07	8.49E+06	9.45E+06	0.00E+00	0.00E+00	9.87E+06	0.00E+00	0.00E+00	0.00E+00	0.00E+00	0.00E+00	8.10E+06	4.14E+06	5.25E+06	4.31E+06	6.42E+06	2.19E+06	4.93E+06	5.62E+06	3.21E+06	8.52E-02
P16401	4.43E+07	1.48E+07	2.17E+07	4.01E+07	1.90E+07	2.57E+07	1.72E+07	2.66E+07	3.65E+07	6.72E+07	0.00E+00	1.12E+08	4.86E+07	1.14E+08	0.00E+00	6.48E+07	4.08E+07	2.62E+07	5.54E+07	3.43E+07	1.07E+07	4.38E+07	8.78E-02
P04181;P04181-2	0.00E+00	0.00E+00	0.00E+00	0.00E+00	0.00E+00	1.23E+07	1.15E+07	0.00E+00	1.10E+07	0.00E+00	0.00E+00	2.33E+07	1.25E+07	0.00E+00	1.37E+07	1.78E+07	6.38E+06	2.98E+06	9.79E+06	7.99E+06	5.52E+06	8.94E+06	8.81E-02
P21912;A0A087WXX8;A0A087WWT1	0.00E+00	0.00E+00	0.00E+00	0.00E+00	0.00E+00	0.00E+00	1.02E+07	0.00E+00	0.00E+00	0.00E+00	0.00E+00	6.95E+06	2.29E+07	0.00E+00	1.50E+07	1.76E+07	4.54E+06	1.28E+06	7.81E+06	7.67E+06	3.62E+06	9.41E+06	8.85E-02
E7EPB3;P50914	0.00E+00	0.00E+00	0.00E+00	0.00E+00	0.00E+00	0.00E+00	9.95E+06	9.66E+06	0.00E+00	0.00E+00	0.00E+00	3.25E+07	1.48E+07	0.00E+00	3.18E+07	1.78E+07	7.28E+06	2.45E+06	1.21E+07	1.14E+07	4.54E+06	1.43E+07	8.99E-02
P61106;X6RFL8	6.45E+07	2.54E+07	4.96E+07	0.00E+00	1.39E+07	3.55E+07	0.00E+00	3.69E+07	0.00E+00	0.00E+00	0.00E+00	0.00E+00	3.65E+07	3.02E+07	0.00E+00	1.66E+07	1.93E+07	2.82E+07	1.04E+07	2.10E+07	2.30E+07	1.53E+07	8.99E-02
Q07021;I3L3Q7;I3L3B0	1.32E+07	0.00E+00	2.28E+07	1.57E+07	3.23E+07	3.53E+07	2.21E+07	2.26E+07	0.00E+00	1.87E+07	0.00E+00	1.47E+07	1.03E+07	0.00E+00	2.55E+07	1.80E+07	1.57E+07	2.05E+07	1.09E+07	1.13E+07	1.11E+07	9.96E+06	9.05E-02

P52907	0.00E+00	0.00E+00	0.00E+00	0.00E+00	2.32E+06	0.00E+00	0.00E+00	0.00E+00	0.00E+00	0.00E+00	0.00E+00	2.75E+06	2.51E+06	2.46E+06	0.00E+00	4.31E+06	8.97E+05	2.90E+05	1.50E+06	1.44E+06	8.20E+05	1.71E+06	9.14E-02
O75915;F8WF90;C9JQU6;F8WF33	2.24E+07	1.16E+07	9.67E+06	1.73E+07	0.00E+00	0.00E+00	2.75E+07	1.82E+07	2.72E+07	2.56E+07	2.29E+07	1.36E+07	1.29E+07	2.09E+07	1.66E+07	2.49E+07	1.70E+07	1.33E+07	2.06E+07	8.65E+06	9.95E+06	5.56E+06	9.31E-02
P08195-2;P08195-3;F5GZS6;P08195;J3KPF3;P08195-4	4.60E+07	3.71E+07	6.38E+07	0.00E+00	0.00E+00	2.91E+06	0.00E+00	0.00E+00	2.78E+06	1.24E+07	0.00E+00	0.00E+00	0.00E+00	0.00E+00	0.00E+00	0.00E+00	1.03E+07	1.87E+07	1.89E+06	2.01E+07	2.61E+07	4.34E+06	9.35E-02
Q96QK1	0.00E+00	0.00E+00	0.00E+00	0.00E+00	1.13E+07	0.00E+00	0.00E+00	1.02E+07	8.16E+06	8.75E+06	0.00E+00	1.22E+07	7.06E+06	1.29E+07	0.00E+00	7.63E+06	4.89E+06	2.69E+06	7.08E+06	5.27E+06	5.00E+06	4.84E+06	9.60E-02
P31930	5.21E+08	3.75E+08	3.34E+08	5.38E+08	4.67E+08	6.39E+08	2.61E+07	4.52E+08	5.30E+08	6.07E+08	5.30E+08	5.90E+08	5.15E+08	6.06E+08	4.47E+08	5.00E+08	4.80E+08	4.19E+08	5.40E+08	1.46E+08	1.85E+08	5.65E+07	9.80E-02
P38646	3.55E+08	4.44E+08	3.79E+08	2.64E+08	4.50E+08	4.51E+08	4.06E+08	5.08E+08	3.35E+08	4.26E+08	0.00E+00	2.97E+08	3.54E+08	4.81E+08	2.13E+07	3.74E+08	3.47E+08	4.07E+08	2.86E+08	1.47E+08	7.49E+07	1.79E+08	9.90E-02
P06733;P06733-2	4.05E+08	7.34E+08	6.07E+08	4.06E+08	5.47E+08	8.76E+08	5.10E+08	4.37E+08	5.16E+08	7.16E+08	2.69E+08	3.57E+08	5.71E+08	1.47E+08	3.72E+08	3.44E+08	4.88E+08	5.65E+08	4.12E+08	1.86E+08	1.68E+08	1.80E+08	9.98E-02
Q9BQE3;F5H5D3;F8VVB9;Q9NY65-2;Q9NY65;C9J2C0	1.51E+07	1.97E+07	1.70E+07	0.00E+00	0.00E+00	7.13E+06	1.15E+07	1.84E+07	0.00E+00	0.00E+00	0.00E+00	0.00E+00	1.46E+07	0.00E+00	1.25E+07	1.04E+07	7.90E+06	1.11E+07	4.70E+06	7.78E+06	7.93E+06	6.58E+06	1.01E-01
P20618	8.24E+06	8.87E+06	4.81E+06	0.00E+00	0.00E+00	0.00E+00	4.65E+06	3.31E+06	0.00E+00	0.00E+00	0.00E+00	0.00E+00	3.30E+06	4.50E+06	0.00E+00	2.11E+06	2.49E+06	3.73E+06	1.24E+06	3.04E+06	3.60E+06	1.82E+06	1.02E-01
Q01081-2;Q01081;M0QYK5;Q01081-3;M0R2N4;Q8WU68-3;Q8WU68-2;Q8WU68;K7EJH3;K7EJM7	7.51E+07	0.00E+00	4.32E+07	3.57E+08	3.16E+08	2.94E+08	1.87E+08	2.00E+08	4.87E+08	3.36E+08	6.31E+08	1.94E+08	6.41E+07	4.65E+08	1.39E+08	3.11E+08	2.56E+08	1.84E+08	3.28E+08	1.77E+08	1.34E+08	1.92E+08	1.04E-01
P35908	1.97E+07	0.00E+00	1.84E+07	1.99E+07	1.63E+07	7.60E+06	1.52E+07	1.52E+07	0.00E+00	1.42E+07	0.00E+00	0.00E+00	1.10E+07	1.71E+07	8.42E+06	1.33E+07	1.10E+07	1.40E+07	8.00E+06	7.43E+06	6.88E+06	7.07E+06	1.05E-01
C9J3L8;C9J5W0;P43307;E9PAL7;C9IZQ1;P43307-2	8.57E+07	1.64E+08	1.14E+08	5.41E+07	1.59E+08	8.70E+07	1.63E+08	1.98E+08	1.09E+08	8.58E+07	1.05E+08	8.11E+07	9.77E+07	1.23E+08	1.31E+07	1.13E+08	1.09E+08	1.28E+08	9.09E+07	4.56E+07	4.99E+07	3.43E+07	1.06E-01
P00558;P00558-2	0.00E+00	0.00E+00	0.00E+00	0.00E+00	0.00E+00	0.00E+00	0.00E+00	1.50E+07	0.00E+00	9.88E+06	0.00E+00	0.00E+00	9.48E+06	1.31E+07	4.26E+07	1.15E+07	6.35E+06	1.87E+06	1.08E+07	1.12E+07	5.29E+06	1.40E+07	1.13E-01
P62873-2;P62873;B1AKQ8;F6X3N5;F6UT28	1.19E+08	8.43E+07	5.96E+07	7.40E+07	1.25E+08	1.03E+08	6.44E+07	6.55E+07	5.74E+07	1.67E+07	5.72E+07	4.65E+07	1.00E+08	4.67E+07	8.28E+07	1.02E+08	7.53E+07	8.69E+07	6.36E+07	2.92E+07	2.58E+07	2.93E+07	1.14E-01
A0A0A0MR02;P45880-2;P45880;P45880-1;Q5JSD2;Q5JSD1	2.39E+07	3.09E+07	2.72E+07	0.00E+00	0.00E+00	0.00E+00	2.67E+07	2.87E+07	1.74E+07	3.14E+07	2.16E+07	1.95E+07	2.39E+07	3.06E+07	3.35E+07	3.47E+07	2.19E+07	1.72E+07	2.66E+07	1.19E+07	1.43E+07	6.76E+06	1.15E-01
P49736;H0Y8E6	6.52E+06	1.12E+07	8.22E+06	0.00E+00	9.94E+06	0.00E+00	7.51E+06	0.00E+00	0.00E+00	0.00E+00	0.00E+00	2.93E+06	7.65E+06	0.00E+00	0.00E+00	6.38E+06	3.77E+06	5.42E+06	2.12E+06	4.24E+06	4.70E+06	3.20E+06	1.23E-01
P00491;G3V5M2	5.29E+07	1.12E+08	1.12E+08	1.32E+07	1.21E+08	2.99E+07	1.50E+08	1.63E+08	0.00E+00	9.72E+07	6.78E+07	3.61E+06	9.48E+07	0.00E+00	3.45E+07	1.16E+08	7.29E+07	9.42E+07	5.17E+07	5.48E+07	5.55E+07	4.81E+07	1.24E-01
Q14204	6.47E+06	4.89E+07	1.62E+07	1.96E+07	6.09E+07	1.11E+07	8.93E+07	6.04E+07	7.59E+06	3.03E+07	4.43E+07	0.00E+00	1.38E+07	8.79E+06	0.00E+00	4.71E+07	2.90E+07	3.91E+07	1.90E+07	2.64E+07	3.00E+07	1.91E+07	1.32E-01
Q15149-7;Q15149-8;Q15149-9;Q15149-5;Q15149-4;Q15149-6;Q15149-3;Q15149-2;Q15149	5.02E+08	2.75E+08	2.05E+08	2.27E+08	1.51E+08	0.00E+00	2.94E+08	2.31E+08	4.80E+08	2.27E+08	3.27E+08	6.08E+08	3.36E+08	3.14E+08	2.74E+08	2.03E+08	2.91E+08	2.36E+08	3.46E+08	1.45E+08	1.41E+08	1.35E+08	1.32E-01
P27824;P27824-2;P27824-3;H0Y9Q7	7.36E+08	2.63E+08	5.15E+08	1.74E+09	1.46E+09	1.17E+09	6.93E+08	9.23E+08	2.49E+09	1.49E+09	3.02E+09	7.70E+08	4.04E+08	1.86E+09	8.71E+08	1.20E+09	1.23E+09	9.37E+08	1.51E+09	7.60E+08	4.93E+08	8.97E+08	1.34E-01
P13645	1.82E+08	9.55E+07	1.25E+08	1.33E+08	1.55E+08	2.13E+08	9.27E+07	1.20E+08	3.79E+08	3.24E+08	0.00E+00	3.02E+08	2.54E+08	3.75E+08	2.28E+06	1.88E+08	1.84E+08	1.39E+08	2.28E+08	1.18E+08	4.17E+07	1.53E+08	1.36E-01
Q15084-3;Q15084;Q15084-4;Q15084-5;Q15084-2	6.77E+06	4.04E+06	8.02E+06	0.00E+00	0.00E+00	1.11E+07	0.00E+00	7.02E+06	0.00E+00	0.00E+00	0.00E+00	4.86E+06	4.20E+06	0.00E+00	5.71E+06	0.00E+00	3.23E+06	4.62E+06	1.85E+06	3.70E+06	4.28E+06	2.58E+06	1.39E-01
P07339;H7C469;F8WD96;C9JH19;F8W787	0.00E+00	0.00E+00	0.00E+00	8.53E+06	8.13E+06	5.72E+06	7.81E+06	1.29E+07	0.00E+00	8.04E+06	0.00E+00	0.00E+00	0.00E+00	8.31E+06	0.00E+00	0.00E+00	3.71E+06	5.38E+06	2.04E+06	4.56E+06	4.88E+06	3.79E+06	1.48E-01
P35998;P35998-2	0.00E+00	0.00E+00	0.00E+00	0.00E+00	0.00E+00	0.00E+00	0.00E+00	0.00E+00	3.11E+06	0.00E+00	3.08E+06	0.00E+00	0.00E+00	0.00E+00	0.00E+00	0.00E+00	3.87E+05	0.00E+00	7.74E+05	1.06E+06	0.00E+00	1.43E+06	1.49E-01

H0Y4X3;G3XAC6;Q14498-3;Q14498-2;Q14498;Q5QP22;Q5QP21;Q5QP23	0.00E+00	0.00E+00	0.00E+00	1.53E+07	0.00E+00	1.55E+07	0.00E+00	0.00E+00	0.00E+00	0.00E+00	0.00E+00	0.00E+00	0.00E+00	0.00E+00	0.00E+00	0.00E+00	1.93E+06	3.86E+06	0.00E+00	5.27E+06	7.14E+06	0.00E+00	1.49E-01
Q15293;Q15293-2	0.00E+00	0.00E+00	0.00E+00	0.00E+00	0.00E+00	0.00E+00	3.95E+06	4.01E+06	0.00E+00	0.00E+00	0.00E+00	0.00E+00	0.00E+00	0.00E+00	0.00E+00	0.00E+00	4.98E+05	9.95E+05	0.00E+00	1.36E+06	1.84E+06	0.00E+00	1.49E-01
P36405	0.00E+00	0.00E+00	0.00E+00	0.00E+00	0.00E+00	0.00E+00	0.00E+00	0.00E+00	0.00E+00	0.00E+00	0.00E+00	5.93E+06	0.00E+00	6.05E+06	0.00E+00	0.00E+00	7.49E+05	0.00E+00	1.50E+06	2.05E+06	0.00E+00	2.77E+06	1.49E-01
Q8IX12-2;Q8IX12;F5H1H2;F5H2E6	0.00E+00	0.00E+00	0.00E+00	0.00E+00	0.00E+00	0.00E+00	0.00E+00	0.00E+00	0.00E+00	0.00E+00	0.00E+00	1.84E+06	1.89E+06	0.00E+00	0.00E+00	0.00E+00	2.33E+05	0.00E+00	4.66E+05	6.37E+05	0.00E+00	8.64E+05	1.49E-01
P49321;P49321-3;P49321-4	0.00E+00	0.00E+00	0.00E+00	0.00E+00	0.00E+00	0.00E+00	0.00E+00	0.00E+00	4.72E+06	4.58E+06	0.00E+00	0.00E+00	0.00E+00	0.00E+00	0.00E+00	0.00E+00	5.81E+05	0.00E+00	1.16E+06	1.59E+06	0.00E+00	2.15E+06	1.49E-01
P42167;P42167-2;G5E972;H0YJH7;P42167-3;P42166	0.00E+00	0.00E+00	0.00E+00	0.00E+00	0.00E+00	0.00E+00	0.00E+00	0.00E+00	0.00E+00	0.00E+00	0.00E+00	0.00E+00	7.59E+06	0.00E+00	7.31E+06	0.00E+00	9.32E+05	0.00E+00	1.86E+06	2.55E+06	0.00E+00	3.45E+06	1.49E-01
O96008;K7EJ57;O96008-2	8.71E+07	1.79E+08	1.28E+08	8.81E+07	1.97E+08	2.91E+07	9.24E+07	8.92E+07	6.63E+07	1.15E+08	1.11E+08	0.00E+00	9.70E+07	7.26E+07	9.03E+07	5.17E+07	9.34E+07	1.11E+08	7.55E+07	4.90E+07	5.48E+07	3.76E+07	1.49E-01
P00338;P00338-4;P00338-3;F5GXY2;P00338-5;P00338-2;F5GYU2;F5H5J4;F5H6W8;F5GXH2	0.00E+00	0.00E+00	0.00E+00	0.00E+00	0.00E+00	0.00E+00	8.91E+06	9.92E+06	0.00E+00	0.00E+00	0.00E+00	0.00E+00	0.00E+00	0.00E+00	0.00E+00	0.00E+00	1.18E+06	2.35E+06	0.00E+00	3.22E+06	4.37E+06	0.00E+00	1.50E-01
K7ES61;Q9BYD3;Q9BYD3-2;X6RAY8	0.00E+00	0.00E+00	0.00E+00	5.73E+06	0.00E+00	5.12E+06	0.00E+00	0.00E+00	0.00E+00	0.00E+00	0.00E+00	0.00E+00	0.00E+00	0.00E+00	0.00E+00	0.00E+00	6.78E+05	1.36E+06	0.00E+00	1.86E+06	2.52E+06	0.00E+00	1.50E-01
Q7L0Y3;C9JVB6	0.00E+00	0.00E+00	0.00E+00	4.21E+06	3.72E+06	0.00E+00	0.00E+00	0.00E+00	0.00E+00	0.00E+00	0.00E+00	0.00E+00	0.00E+00	0.00E+00	0.00E+00	0.00E+00	4.95E+05	9.91E+05	0.00E+00	1.36E+06	1.84E+06	0.00E+00	1.50E-01
Q9BZE1;S4R369	0.00E+00	0.00E+00	0.00E+00	0.00E+00	0.00E+00	0.00E+00	2.64E+06	3.07E+06	0.00E+00	0.00E+00	0.00E+00	0.00E+00	0.00E+00	0.00E+00	0.00E+00	0.00E+00	3.57E+05	7.13E+05	0.00E+00	9.78E+05	1.33E+06	0.00E+00	1.50E-01
J3KS22;Q7Z4W1;J3KRZ4;J3QS36	0.00E+00	0.00E+00	0.00E+00	0.00E+00	0.00E+00	0.00E+00	8.76E+06	7.27E+06	0.00E+00	0.00E+00	0.00E+00	0.00E+00	0.00E+00	0.00E+00	0.00E+00	0.00E+00	1.00E+06	2.00E+06	0.00E+00	2.75E+06	3.73E+06	0.00E+00	1.51E-01
X1WI28;P27635;F8W7C6;A0A087WV22	0.00E+00	0.00E+00	0.00E+00	0.00E+00	0.00E+00	0.00E+00	0.00E+00	0.00E+00	0.00E+00	0.00E+00	0.00E+00	0.00E+00	0.00E+00	0.00E+00	9.51E+06	7.81E+06	1.08E+06	0.00E+00	2.16E+06	2.97E+06	0.00E+00	4.03E+06	1.51E-01
Q9BVK6	0.00E+00	0.00E+00	0.00E+00	0.00E+00	0.00E+00	0.00E+00	0.00E+00	0.00E+00	2.45E+07	1.96E+07	0.00E+00	0.00E+00	0.00E+00	0.00E+00	0.00E+00	0.00E+00	2.75E+06	0.00E+00	5.51E+06	7.58E+06	0.00E+00	1.03E+07	1.52E-01
H7BZJ3	0.00E+00	6.45E+06	0.00E+00	0.00E+00	5.09E+06	0.00E+00	0.00E+00	0.00E+00	0.00E+00	0.00E+00	0.00E+00	0.00E+00	0.00E+00	0.00E+00	0.00E+00	0.00E+00	7.21E+05	1.44E+06	0.00E+00	1.99E+06	2.70E+06	0.00E+00	1.52E-01
Q6P2E9-2;Q6P2E9;I3L2F4	1.04E+07	2.33E+07	2.17E+07	5.11E+06	1.09E+07	2.73E+07	0.00E+00	2.10E+07	1.64E+07	0.00E+00	9.17E+06	7.21E+06	6.46E+06	0.00E+00	7.43E+06	2.11E+07	1.17E+07	1.50E+07	8.48E+06	8.96E+06	9.73E+06	7.31E+06	1.54E-01
REV__P11230	3.97E+07	3.97E+07	3.21E+07	9.57E+06	9.98E+06	4.51E+07	2.12E+07	2.49E+07	2.67E+07	2.01E+07	2.29E+07	0.00E+00	2.63E+07	2.95E+07	0.00E+00	1.99E+07	2.30E+07	2.78E+07	1.82E+07	1.32E+07	1.36E+07	1.17E+07	1.54E-01
P30153;B3KQV6	0.00E+00	0.00E+00	0.00E+00	0.00E+00	0.00E+00	0.00E+00	0.00E+00	0.00E+00	0.00E+00	0.00E+00	1.00E+07	0.00E+00	7.56E+06	0.00E+00	0.00E+00	0.00E+00	1.10E+06	0.00E+00	2.20E+06	3.03E+06	0.00E+00	4.12E+06	1.54E-01
F8VZ49;P09651-3;F8W6I7;P09651-2;P09651;Q32P51;F8VTQ5	0.00E+00	0.00E+00	0.00E+00	0.00E+00	0.00E+00	0.00E+00	0.00E+00	0.00E+00	1.05E+07	7.91E+06	0.00E+00	0.00E+00	0.00E+00	0.00E+00	0.00E+00	0.00E+00	1.15E+06	0.00E+00	2.30E+06	3.17E+06	0.00E+00	4.31E+06	1.54E-01
Q96N66-3;Q96N66;H7C2M4;C9J4E9;A9C4B8;Q96N66-2	0.00E+00	0.00E+00	0.00E+00	0.00E+00	5.21E+06	0.00E+00	0.00E+00	6.94E+06	0.00E+00	0.00E+00	0.00E+00	0.00E+00	0.00E+00	0.00E+00	0.00E+00	0.00E+00	7.60E+05	1.52E+06	0.00E+00	2.10E+06	2.85E+06	0.00E+00	1.54E-01
P36957-2;P36957;Q86SW4	0.00E+00	0.00E+00	0.00E+00	0.00E+00	0.00E+00	0.00E+00	2.93E+06	3.95E+06	0.00E+00	0.00E+00	0.00E+00	0.00E+00	0.00E+00	0.00E+00	0.00E+00	0.00E+00	4.30E+05	8.60E+05	0.00E+00	1.19E+06	1.62E+06	0.00E+00	1.54E-01
Q9HC38-2;Q9HC38;F6TLX2;I3L1I0;Q9HC38-3;I3L3Q4	2.34E+07	0.00E+00	1.73E+07	0.00E+00	0.00E+00	0.00E+00	0.00E+00	0.00E+00	0.00E+00	0.00E+00	0.00E+00	0.00E+00	0.00E+00	0.00E+00	0.00E+00	0.00E+00	2.54E+06	5.08E+06	0.00E+00	7.04E+06	9.56E+06	0.00E+00	1.55E-01
Q9NX40;D6RG39;D6RIT9;D6RDK6;Q9NX40-3;Q9NX40-2;Q9NX40-4;D6RBN5	3.30E+07	3.97E+07	3.67E+07	2.79E+07	3.67E+07	3.74E+07	0.00E+00	3.25E+07	2.30E+07	2.30E+07	4.00E+07	0.00E+00	0.00E+00	2.82E+07	3.15E+07	1.67E+07	2.54E+07	3.05E+07	2.03E+07	1.41E+07	1.29E+07	1.43E+07	1.56E-01
O60568;H7C2V1	0.00E+00	0.00E+00	0.00E+00	0.00E+00	0.00E+00	6.27E+06	4.31E+06	0.00E+00	0.00E+00	0.00E+00	0.00E+00	0.00E+00	0.00E+00	0.00E+00	0.00E+00	0.00E+00	6.61E+05	1.32E+06	0.00E+00	1.84E+06	2.50E+06	0.00E+00	1.57E-01
B4DUR8;P49368;P49368-2	0.00E+00	0.00E+00	0.00E+00	0.00E+00	0.00E+00	0.00E+00	2.33E+06	3.40E+06	0.00E+00	0.00E+00	0.00E+00	0.00E+00	0.00E+00	0.00E+00	0.00E+00	0.00E+00	3.58E+05	7.16E+05	0.00E+00	9.98E+05	1.36E+06	0.00E+00	1.58E-01

P11234;P11234-3;P11234-2;C9J6B1;C9JQB3	0.00E+00	0.00E+00	0.00E+00	0.00E+00	0.00E+00	5.04E+06	0.00E+00	7.40E+06	0.00E+00	0.00E+00	0.00E+00	0.00E+00	0.00E+00	0.00E+00	0.00E+00	0.00E+00	7.78E+05	1.56E+06	0.00E+00	2.17E+06	2.95E+06	0.00E+00	1.58E-01
P22061;P22061-2;A0A0A0MRJ6;H7BY58;F6S8N6	0.00E+00	7.34E+06	0.00E+00	0.00E+00	0.00E+00	0.00E+00	0.00E+00	1.08E+07	0.00E+00	0.00E+00	0.00E+00	0.00E+00	0.00E+00	0.00E+00	0.00E+00	0.00E+00	1.13E+06	2.27E+06	0.00E+00	3.16E+06	4.30E+06	0.00E+00	1.58E-01
Q8NBJ5;M0QYH0	0.00E+00	0.00E+00	0.00E+00	0.00E+00	0.00E+00	0.00E+00	0.00E+00	0.00E+00	0.00E+00	3.55E+06	0.00E+00	0.00E+00	0.00E+00	0.00E+00	0.00E+00	2.41E+06	3.72E+05	0.00E+00	7.44E+05	1.04E+06	0.00E+00	1.41E+06	1.58E-01
Q00688	0.00E+00	0.00E+00	0.00E+00	0.00E+00	0.00E+00	0.00E+00	0.00E+00	0.00E+00	0.00E+00	0.00E+00	0.00E+00	0.00E+00	0.00E+00	9.43E+05	0.00E+00	1.41E+06	1.47E+05	0.00E+00	2.94E+05	4.10E+05	0.00E+00	5.57E+05	1.59E-01
P54727;Q5W0S4;Q5W0S5;P54727-2	8.13E+06	6.82E+06	9.32E+06	1.62E+07	6.52E+06	9.11E+06	5.24E+06	1.26E+07	1.01E+07	4.44E+06	0.00E+00	0.00E+00	8.46E+06	1.38E+07	0.00E+00	9.68E+06	7.52E+06	9.24E+06	5.81E+06	4.79E+06	3.59E+06	5.44E+06	1.59E-01
Q9UQ80-2;Q9UQ80;F8VR77	0.00E+00	0.00E+00	0.00E+00	0.00E+00	0.00E+00	9.20E+06	6.12E+06	0.00E+00	0.00E+00	0.00E+00	0.00E+00	0.00E+00	0.00E+00	0.00E+00	0.00E+00	0.00E+00	9.57E+05	1.91E+06	0.00E+00	2.68E+06	3.64E+06	0.00E+00	1.59E-01
O43615;M0QXU7	6.72E+06	0.00E+00	4.41E+06	0.00E+00	0.00E+00	0.00E+00	0.00E+00	0.00E+00	0.00E+00	0.00E+00	0.00E+00	0.00E+00	0.00E+00	0.00E+00	0.00E+00	0.00E+00	6.96E+05	1.39E+06	0.00E+00	1.95E+06	2.65E+06	0.00E+00	1.60E-01
P05198;H0YJS4;G3V4T5	0.00E+00	0.00E+00	0.00E+00	0.00E+00	0.00E+00	0.00E+00	8.67E+06	1.32E+07	0.00E+00	0.00E+00	0.00E+00	0.00E+00	0.00E+00	0.00E+00	0.00E+00	0.00E+00	1.37E+06	2.74E+06	0.00E+00	3.83E+06	5.21E+06	0.00E+00	1.60E-01
I3L1P8;Q02978;Q02978-2	0.00E+00	0.00E+00	0.00E+00	0.00E+00	0.00E+00	7.14E+06	1.10E+07	0.00E+00	0.00E+00	0.00E+00	0.00E+00	0.00E+00	0.00E+00	0.00E+00	0.00E+00	0.00E+00	1.14E+06	2.27E+06	0.00E+00	3.18E+06	4.33E+06	0.00E+00	1.60E-01
P10586-2;P10586;H0Y6Z7;H0Y7Z9	0.00E+00	0.00E+00	0.00E+00	0.00E+00	0.00E+00	0.00E+00	5.95E+06	9.22E+06	0.00E+00	0.00E+00	0.00E+00	0.00E+00	0.00E+00	0.00E+00	0.00E+00	0.00E+00	9.48E+05	1.90E+06	0.00E+00	2.66E+06	3.62E+06	0.00E+00	1.60E-01
J3KRB3;A0A087WWH0;J3QS96;A0A087WXM6;J3QQT2;J3KRX5;P18621;P18621-3;A0A0A6YYL6;A0A087WY81;P18621-2;J3QLC8;A0A0A0MRF8	0.00E+00	0.00E+00	0.00E+00	0.00E+00	0.00E+00	0.00E+00	0.00E+00	0.00E+00	1.77E+06	0.00E+00	0.00E+00	0.00E+00	0.00E+00	2.80E+06	0.00E+00	0.00E+00	2.86E+05	0.00E+00	5.72E+05	8.03E+05	0.00E+00	1.09E+06	1.61E-01
P00568;Q5T9B7	0.00E+00	0.00E+00	0.00E+00	0.00E+00	0.00E+00	2.90E+06	0.00E+00	1.83E+06	0.00E+00	0.00E+00	0.00E+00	0.00E+00	0.00E+00	0.00E+00	0.00E+00	0.00E+00	2.96E+05	5.92E+05	0.00E+00	8.31E+05	1.13E+06	0.00E+00	1.61E-01
Q9NXF1-2;Q9NXF1	0.00E+00	0.00E+00	0.00E+00	0.00E+00	0.00E+00	0.00E+00	0.00E+00	0.00E+00	9.54E+06	0.00E+00	0.00E+00	0.00E+00	0.00E+00	1.53E+07	0.00E+00	0.00E+00	1.55E+06	0.00E+00	3.11E+06	4.37E+06	0.00E+00	5.95E+06	1.62E-01
F8WEX5;E9PG02;E9PBT8;C9JL30;Q8NBJ7;J3KQJ1;Q8NBJ7-3;C9J660;F8WA42;F8WEV7;F8WES7;Q8NBJ7-2;Q8NBJ7-5;J3QT17;H7C3B2;A8MXB9;Q8NBJ7-4	0.00E+00	0.00E+00	0.00E+00	0.00E+00	0.00E+00	0.00E+00	0.00E+00	0.00E+00	0.00E+00	0.00E+00	0.00E+00	0.00E+00	0.00E+00	0.00E+00	3.36E+06	5.42E+06	5.48E+05	0.00E+00	1.10E+06	1.55E+06	0.00E+00	2.10E+06	1.62E-01
Q15717;Q15717-2;M0QZR9	0.00E+00	0.00E+00	0.00E+00	0.00E+00	1.92E+07	0.00E+00	0.00E+00	1.19E+07	0.00E+00	0.00E+00	0.00E+00	0.00E+00	0.00E+00	0.00E+00	0.00E+00	0.00E+00	1.94E+06	3.89E+06	0.00E+00	5.48E+06	7.46E+06	0.00E+00	1.63E-01
Q96HS1-2;Q96HS1;F5GXG4	2.43E+08	3.10E+08	2.89E+08	2.05E+08	1.87E+08	4.38E+07	2.90E+08	4.11E+08	1.95E+08	1.68E+08	1.91E+08	1.10E+08	3.01E+08	1.16E+08	2.18E+08	1.67E+08	2.15E+08	2.47E+08	1.83E+08	9.07E+07	1.08E+08	6.06E+07	1.65E-01
O60664-4;O60664-3;O60664;O60664-2;K7ERZ3	2.51E+08	3.31E+08	2.93E+08	3.02E+08	3.90E+08	3.68E+08	3.12E+08	3.01E+08	2.93E+08	3.43E+08	2.56E+08	2.97E+08	3.58E+08	2.40E+08	2.72E+08	2.20E+08	3.02E+08	3.18E+08	2.85E+08	4.78E+07	4.41E+07	4.80E+07	1.65E-01
Q00610-2;Q00610;A0A087WVQ6	0.00E+00	0.00E+00	0.00E+00	0.00E+00	1.18E+07	6.89E+06	0.00E+00	0.00E+00	0.00E+00	0.00E+00	0.00E+00	0.00E+00	0.00E+00	0.00E+00	0.00E+00	0.00E+00	1.17E+06	2.34E+06	0.00E+00	3.31E+06	4.52E+06	0.00E+00	1.66E-01
Q5HY54;Q60FE5;P21333-2;P21333;A0A087WWY3	2.49E+07	0.00E+00	0.00E+00	2.01E+07	1.27E+07	1.83E+07	1.45E+07	1.38E+07	0.00E+00	1.64E+07	2.19E+07	0.00E+00	1.30E+07	0.00E+00	0.00E+00	0.00E+00	9.73E+06	1.30E+07	6.41E+06	9.40E+06	8.96E+06	9.17E+06	1.66E-01
B1ANR0;Q13310-2;Q13310;Q13310-3;H0Y5F5	0.00E+00	0.00E+00	0.00E+00	0.00E+00	0.00E+00	0.00E+00	0.00E+00	0.00E+00	8.74E+05	4.89E+05	0.00E+00	0.00E+00	0.00E+00	0.00E+00	0.00E+00	0.00E+00	8.52E+04	0.00E+00	1.70E+05	2.43E+05	0.00E+00	3.32E+05	1.69E-01
Q6PIU2;Q6PIU2-2;A0A0A0MTJ9;Q6PIU2-3;H7C046	0.00E+00	0.00E+00	0.00E+00	0.00E+00	0.00E+00	1.09E+07	0.00E+00	5.88E+06	0.00E+00	0.00E+00	0.00E+00	0.00E+00	0.00E+00	0.00E+00	0.00E+00	0.00E+00	1.05E+06	2.10E+06	0.00E+00	3.01E+06	4.12E+06	0.00E+00	1.71E-01
I3L4U9;Q8TAT6;Q8TAT6-2	3.96E+06	0.00E+00	7.46E+06	0.00E+00	0.00E+00	0.00E+00	0.00E+00	0.00E+00	0.00E+00	0.00E+00	0.00E+00	0.00E+00	0.00E+00	0.00E+00	0.00E+00	0.00E+00	7.14E+05	1.43E+06	0.00E+00	2.05E+06	2.81E+06	0.00E+00	1.72E-01
Q9C004;Q9C004-2	9.34E+06	4.88E+06	0.00E+00	0.00E+00	0.00E+00	0.00E+00	0.00E+00	0.00E+00	0.00E+00	0.00E+00	0.00E+00	0.00E+00	0.00E+00	0.00E+00	0.00E+00	0.00E+00	8.89E+05	1.78E+06	0.00E+00	2.56E+06	3.50E+06	0.00E+00	1.73E-01
Q9UIQ6-3;Q9UIQ6-2;Q9UIQ6	0.00E+00	0.00E+00	0.00E+00	0.00E+00	0.00E+00	0.00E+00	0.00E+00	0.00E+00	9.68E+05	0.00E+00	0.00E+00	5.05E+05	0.00E+00	0.00E+00	0.00E+00	0.00E+00	9.21E+04	0.00E+00	1.84E+05	2.65E+05	0.00E+00	3.63E+05	1.73E-01

Q32P28-4;Q32P28;Q32P28-3;E2QRI1;Q32P28-2	0.00E+00	0.00E+00	0.00E+00	0.00E+00	0.00E+00	0.00E+00	0.00E+00	0.00E+00	0.00E+00	0.00E+00	0.00E+00	9.32E+06	0.00E+00	0.00E+00	0.00E+00	4.84E+06	8.85E+05	0.00E+00	1.77E+06	2.55E+06	0.00E+00	3.49E+06	1.73E-01
P06737-2;E9PK47;P06737	0.00E+00	0.00E+00	0.00E+00	0.00E+00	0.00E+00	8.25E+06	0.00E+00	9.11E+06	0.00E+00	0.00E+00	0.00E+00	1.56E+07	9.91E+06	0.00E+00	1.15E+07	1.22E+07	4.16E+06	2.17E+06	6.16E+06	5.76E+06	4.03E+06	6.77E+06	1.74E-01
A0A096LNH5;H7C2G3;P30042-2;A0A096LPI6;H7C1F6;P30042;A0A096LNJ1;A0A096LP73;A0A096LP12;F2Z2Q0;H7BYH1	0.00E+00	6.01E+06	0.00E+00	0.00E+00	3.05E+06	0.00E+00	0.00E+00	0.00E+00	0.00E+00	0.00E+00	0.00E+00	0.00E+00	0.00E+00	0.00E+00	0.00E+00	0.00E+00	5.66E+05	1.13E+06	0.00E+00	1.64E+06	2.24E+06	0.00E+00	1.75E-01
P25786;P25786-2;F5GX11	0.00E+00	3.75E+07	0.00E+00	3.93E+07	1.99E+07	0.00E+00	4.09E+07	4.25E+07	6.31E+07	1.48E+07	1.41E+07	8.89E+07	1.44E+07	5.90E+07	2.50E+07	3.85E+07	3.11E+07	2.25E+07	3.97E+07	2.51E+07	1.99E+07	2.79E+07	1.78E-01
P54577	0.00E+00	8.60E+06	0.00E+00	0.00E+00	4.11E+06	0.00E+00	0.00E+00	0.00E+00	0.00E+00	0.00E+00	0.00E+00	0.00E+00	0.00E+00	0.00E+00	0.00E+00	0.00E+00	7.94E+05	1.59E+06	0.00E+00	2.32E+06	3.18E+06	0.00E+00	1.79E-01
K7EJE8;K7EKE6;P36776-2;P36776;P36776-3	3.94E+07	6.40E+07	5.09E+07	2.43E+07	1.99E+07	2.16E+07	6.00E+07	3.27E+07	3.07E+07	3.42E+07	4.52E+07	1.96E+07	4.04E+07	8.04E+06	3.25E+07	1.60E+07	3.37E+07	3.91E+07	2.83E+07	1.58E+07	1.75E+07	1.27E+07	1.80E-01
E7EWC2;F5H7S7;Q13576;E9PDT6;D6R939;Q13576-3;Q13576-2	0.00E+00	0.00E+00	0.00E+00	0.00E+00	0.00E+00	0.00E+00	2.76E+06	5.86E+06	0.00E+00	0.00E+00	0.00E+00	0.00E+00	0.00E+00	0.00E+00	0.00E+00	0.00E+00	5.39E+05	1.08E+06	0.00E+00	1.58E+06	2.16E+06	0.00E+00	1.80E-01
Q02878	7.67E+07	4.57E+07	6.47E+07	9.72E+07	5.47E+07	1.26E+08	0.00E+00	1.10E+08	4.82E+07	6.95E+07	3.75E+07	2.61E+07	2.64E+07	6.80E+07	6.19E+07	6.22E+07	6.09E+07	7.19E+07	5.00E+07	3.21E+07	4.01E+07	1.81E+07	1.81E-01
P08559-3;P08559;P08559-2;P08559-4;Q5JPU0;Q5JPT9;Q5JPU1	0.00E+00	3.57E+07	3.26E+07	0.00E+00	0.00E+00	1.92E+07	3.94E+07	3.97E+07	0.00E+00	0.00E+00	0.00E+00	1.34E+07	2.30E+07	0.00E+00	2.17E+07	2.26E+07	1.55E+07	2.08E+07	1.01E+07	1.57E+07	1.84E+07	1.12E+07	1.81E-01
P40939	0.00E+00	0.00E+00	0.00E+00	0.00E+00	0.00E+00	5.77E+06	0.00E+00	1.24E+07	0.00E+00	0.00E+00	0.00E+00	0.00E+00	0.00E+00	0.00E+00	0.00E+00	0.00E+00	1.13E+06	2.26E+06	0.00E+00	3.32E+06	4.55E+06	0.00E+00	1.81E-01
P62081;B5MCP9	0.00E+00	7.43E+05	1.60E+06	0.00E+00	0.00E+00	0.00E+00	0.00E+00	0.00E+00	0.00E+00	0.00E+00	0.00E+00	0.00E+00	0.00E+00	0.00E+00	0.00E+00	0.00E+00	1.46E+05	2.92E+05	0.00E+00	4.29E+05	5.88E+05	0.00E+00	1.81E-01
G3V3R6;P17931	4.13E+06	3.62E+06	0.00E+00	0.00E+00	0.00E+00	0.00E+00	0.00E+00	5.44E+06	0.00E+00	0.00E+00	0.00E+00	0.00E+00	0.00E+00	0.00E+00	0.00E+00	3.02E+06	1.01E+06	1.65E+06	3.77E+05	1.87E+06	2.33E+06	1.07E+06	1.82E-01
Q96HY6;A0A0A0MRX2;Q96HY6-2	5.79E+06	7.73E+06	0.00E+00	0.00E+00	5.95E+06	0.00E+00	8.35E+06	1.25E+07	0.00E+00	4.75E+06	0.00E+00	0.00E+00	0.00E+00	7.92E+06	0.00E+00	5.24E+06	3.64E+06	5.04E+06	2.24E+06	4.13E+06	4.65E+06	3.22E+06	1.83E-01
Q5TA02;P78417-3;P78417;Q5TA01;P78417-2	0.00E+00	0.00E+00	0.00E+00	5.41E+06	0.00E+00	5.55E+06	4.34E+06	4.57E+06	0.00E+00	0.00E+00	0.00E+00	0.00E+00	0.00E+00	3.65E+06	0.00E+00	3.66E+06	1.70E+06	2.49E+06	9.13E+05	2.32E+06	2.69E+06	1.69E+06	1.83E-01
P22234;E9PBS1;P22234-2;D6RF62	0.00E+00	0.00E+00	0.00E+00	0.00E+00	0.00E+00	0.00E+00	0.00E+00	0.00E+00	0.00E+00	0.00E+00	0.00E+00	0.00E+00	9.25E+06	0.00E+00	0.00E+00	4.06E+06	8.32E+05	0.00E+00	1.66E+06	2.46E+06	0.00E+00	3.38E+06	1.85E-01
P62333;A0A087X2I1;H0YJC0	0.00E+00	2.10E+07	0.00E+00	1.35E+07	0.00E+00	0.00E+00	3.23E+07	3.83E+07	5.29E+07	0.00E+00	0.00E+00	2.86E+07	1.70E+07	6.77E+07	2.22E+07	2.86E+07	2.01E+07	1.31E+07	2.71E+07	2.07E+07	1.58E+07	2.36E+07	1.86E-01
P47985;P0C7P4	0.00E+00	0.00E+00	0.00E+00	6.46E+06	0.00E+00	0.00E+00	0.00E+00	0.00E+00	8.71E+06	0.00E+00	0.00E+00	1.19E+07	0.00E+00	0.00E+00	0.00E+00	7.40E+06	2.15E+06	8.08E+05	3.50E+06	3.99E+06	2.28E+06	4.98E+06	1.87E-01
Q9NSE4	4.55E+07	4.08E+07	5.31E+07	1.16E+08	9.21E+07	7.45E+07	9.43E+07	7.88E+07	5.35E+07	7.19E+07	4.14E+07	4.15E+07	9.52E+07	2.07E+07	6.75E+07	6.62E+07	6.58E+07	7.44E+07	5.72E+07	2.55E+07	2.64E+07	2.30E+07	1.87E-01
Q15738;C9JDR0	0.00E+00	0.00E+00	0.00E+00	0.00E+00	2.56E+06	0.00E+00	2.65E+06	5.40E+06	2.15E+06	0.00E+00	0.00E+00	0.00E+00	0.00E+00	0.00E+00	0.00E+00	0.00E+00	7.97E+05	1.33E+06	2.68E+05	1.57E+06	2.02E+06	7.59E+05	1.88E-01
P05388-2;P05388-2;F8VWS0;F8VU65;F8VPE8;G3V210;F8VW21;Q8NHW5;F8VQY6;F8VRK7	0.00E+00	0.00E+00	0.00E+00	0.00E+00	0.00E+00	9.07E+06	1.81E+07	1.29E+07	0.00E+00	0.00E+00	0.00E+00	0.00E+00	0.00E+00	0.00E+00	0.00E+00	9.17E+06	3.08E+06	5.02E+06	1.15E+06	5.84E+06	7.34E+06	3.24E+06	1.94E-01
P63010-3;P63010;P63010-2;A0A087X253;A0A087WU93;A0A087WZQ6;A0A087WYD1	2.61E+07	1.67E+07	0.00E+00	2.52E+07	2.41E+07	2.34E+07	1.23E+07	1.70E+07	4.24E+07	2.90E+07	0.00E+00	0.00E+00	0.00E+00	0.00E+00	0.00E+00	0.00E+00	1.35E+07	1.81E+07	8.92E+06	1.39E+07	8.82E+06	1.69E+07	1.95E-01
P07741;P07741-2;H3BQF1;H3BQZ9;H3BQB1;H3BSW3	0.00E+00	1.27E+07	1.32E+07	0.00E+00	0.00E+00	0.00E+00	8.08E+06	0.00E+00	1.39E+07	1.06E+07	7.63E+06	1.23E+07	1.00E+07	0.00E+00	0.00E+00	1.04E+07	6.17E+06	4.25E+06	8.10E+06	5.85E+06	6.05E+06	5.31E+06	1.97E-01
P34897-3;P34897;P34897-2;G3V2Y4;H0YIZ0;G3V5L0	2.87E+07	0.00E+00	2.70E+07	0.00E+00	1.73E+07	2.44E+07	1.79E+07	0.00E+00	0.00E+00	2.17E+07	0.00E+00	9.47E+07	4.22E+07	0.00E+00	6.12E+07	3.21E+07	2.30E+07	1.44E+07	3.15E+07	2.63E+07	1.26E+07	3.40E+07	2.03E-01

P27816-6;P27816;E7EVA0;P27816-2;P27816-5	3.05E+07	3.78E+07	4.34E+07	2.37E+07	3.04E+07	2.78E+07	3.28E+07	4.00E+07	1.63E+07	4.47E+07	0.00E+00	2.33E+07	3.47E+07	3.84E+07	0.00E+00	3.81E+07	2.89E+07	3.33E+07	2.45E+07	1.36E+07	6.62E+06	1.76E+07	2.05E-01
D6R9P3;D6RD18;Q99729-3;D6RBZ0;A0A087WZV1;Q99729-2;Q99729-4;Q99729	9.19E+08	1.46E+09	1.47E+09	1.71E+09	1.97E+09	2.15E+09	2.04E+09	1.18E+09	1.52E+09	1.08E+09	1.43E+09	1.23E+09	1.07E+09	1.97E+09	1.32E+08	1.82E+09	1.45E+09	1.61E+09	1.28E+09	5.17E+08	4.34E+08	5.68E+08	2.09E-01
P26641;P26641-2	3.54E+07	0.00E+00	0.00E+00	2.12E+07	2.62E+07	4.52E+07	2.94E+07	2.36E+07	4.34E+07	4.06E+07	0.00E+00	5.84E+07	3.39E+07	5.48E+07	0.00E+00	5.59E+07	2.92E+07	2.26E+07	3.59E+07	2.06E+07	1.58E+07	2.37E+07	2.10E-01
P60709	1.41E+07	0.00E+00	0.00E+00	1.68E+07	1.06E+07	0.00E+00	0.00E+00	0.00E+00	1.04E+07	1.07E+07	1.38E+07	1.35E+07	1.45E+07	1.39E+07	0.00E+00	0.00E+00	7.40E+06	5.18E+06	9.61E+06	6.92E+06	7.34E+06	6.12E+06	2.11E-01
Q8NBS9-2;Q86UY0;Q8NBS9	1.39E+07	0.00E+00	0.00E+00	7.31E+06	1.44E+07	1.24E+07	1.40E+07	1.79E+07	7.28E+06	0.00E+00	1.10E+07	0.00E+00	9.23E+06	0.00E+00	0.00E+00	1.72E+07	7.79E+06	9.99E+06	5.59E+06	6.87E+06	6.82E+06	6.61E+06	2.12E-01
Q07065	1.34E+09	5.92E+08	9.98E+08	3.55E+09	3.81E+09	2.41E+09	1.82E+09	1.84E+09	4.86E+09	3.56E+09	5.52E+09	1.96E+09	8.71E+08	3.57E+09	1.26E+09	2.26E+09	2.51E+09	2.05E+09	2.98E+09	1.47E+09	1.15E+09	1.68E+09	2.13E-01
Q6P2Q9	0.00E+00	0.00E+00	0.00E+00	0.00E+00	0.00E+00	0.00E+00	1.08E+07	0.00E+00	0.00E+00	1.25E+07	0.00E+00	1.80E+07	0.00E+00	0.00E+00	0.00E+00	1.12E+07	3.28E+06	1.35E+06	5.20E+06	6.05E+06	3.82E+06	7.44E+06	2.13E-01
P04264	0.00E+00	0.00E+00	0.00E+00	0.00E+00	0.00E+00	1.98E+07	6.05E+06	0.00E+00	0.00E+00	0.00E+00	0.00E+00	0.00E+00	0.00E+00	0.00E+00	0.00E+00	0.00E+00	1.62E+06	3.23E+06	0.00E+00	5.08E+06	7.03E+06	0.00E+00	2.14E-01
P05534;P30447;Q5SRN7;A7MAP4;P30455;P30443;P13746;P04439;Q5SRN5;P13746-2;P01893	0.00E+00	0.00E+00	0.00E+00	0.00E+00	3.78E+06	0.00E+00	5.47E+06	6.91E+06	0.00E+00	4.18E+06	0.00E+00	0.00E+00	0.00E+00	0.00E+00	0.00E+00	0.00E+00	1.27E+06	2.02E+06	5.23E+05	2.36E+06	2.91E+06	1.48E+06	2.16E-01
Q9BPW8	7.73E+06	6.87E+06	0.00E+00	0.00E+00	0.00E+00	9.38E+06	0.00E+00	0.00E+00	1.12E+07	0.00E+00	0.00E+00	1.30E+07	7.89E+06	0.00E+00	9.53E+06	7.25E+06	4.55E+06	3.00E+06	6.11E+06	4.92E+06	4.19E+06	5.36E+06	2.17E-01
P17174;P17174-2	1.87E+07	1.89E+07	1.93E+07	0.00E+00	0.00E+00	1.37E+07	2.00E+07	1.06E+07	0.00E+00	0.00E+00	0.00E+00	0.00E+00	2.45E+07	0.00E+00	1.43E+07	1.52E+07	9.70E+06	1.27E+07	6.75E+06	9.35E+06	8.46E+06	9.79E+06	2.18E-01
P53007;B4DP62	1.10E+07	4.17E+07	2.02E+07	8.75E+06	2.77E+07	1.63E+07	1.50E+07	1.05E+07	9.19E+06	5.72E+07	1.57E+07	3.27E+07	2.50E+07	0.00E+00	4.90E+07	4.76E+07	2.42E+07	1.89E+07	2.95E+07	1.69E+07	1.11E+07	2.06E+07	2.19E-01
P62753;A2A3R5	2.87E+06	3.14E+06	4.43E+06	0.00E+00	0.00E+00	0.00E+00	2.44E+06	4.75E+06	0.00E+00	0.00E+00	0.00E+00	0.00E+00	0.00E+00	0.00E+00	0.00E+00	6.57E+06	1.51E+06	2.20E+06	8.21E+05	2.20E+06	1.98E+06	2.32E+06	2.21E-01
P18669	0.00E+00	4.40E+06	0.00E+00	0.00E+00	0.00E+00	0.00E+00	3.38E+06	0.00E+00	7.80E+06	0.00E+00	3.07E+06	5.51E+06	0.00E+00	4.29E+06	0.00E+00	0.00E+00	1.78E+06	9.73E+05	2.58E+06	2.57E+06	1.82E+06	3.06E+06	2.22E-01
Q92499;Q92499-2;Q92499-3;F1T0B3;A0A087X2G1	0.00E+00	1.27E+07	0.00E+00	0.00E+00	0.00E+00	0.00E+00	9.36E+06	7.85E+06	0.00E+00	0.00E+00	0.00E+00	0.00E+00	8.18E+06	0.00E+00	0.00E+00	0.00E+00	2.38E+06	3.74E+06	1.02E+06	4.37E+06	5.33E+06	2.89E+06	2.26E-01
Q8NI27	2.46E+07	0.00E+00	2.55E+07	3.15E+07	2.29E+07	2.78E+07	0.00E+00	1.78E+07	3.20E+07	2.91E+07	3.79E+07	1.87E+07	1.72E+07	2.51E+07	1.78E+07	2.33E+07	2.20E+07	1.88E+07	2.51E+07	1.03E+07	1.22E+07	7.43E+06	2.28E-01
Q9NR31;Q9NR31-2;Q5SQT8	1.21E+07	6.01E+06	7.28E+06	0.00E+00	0.00E+00	1.07E+07	0.00E+00	0.00E+00	8.03E+06	0.00E+00	7.37E+06	4.55E+06	4.33E+06	1.84E+07	8.52E+06	1.19E+07	6.20E+06	4.52E+06	7.89E+06	5.46E+06	5.18E+06	5.53E+06	2.29E-01
Q12931-2;Q12931;I3L0K7	1.20E+08	6.42E+07	5.28E+07	1.63E+08	7.39E+07	1.05E+08	1.16E+08	1.19E+08	6.58E+07	1.67E+08	1.41E+08	2.55E+08	1.38E+08	0.00E+00	2.53E+08	1.27E+08	1.23E+08	1.02E+08	1.43E+08	6.73E+07	3.63E+07	8.61E+07	2.29E-01
O94826	3.24E+07	0.00E+00	0.00E+00	3.59E+07	0.00E+00	1.53E+07	0.00E+00	2.43E+07	0.00E+00	0.00E+00	0.00E+00	1.60E+07	0.00E+00	2.61E+07	0.00E+00	0.00E+00	9.37E+06	1.35E+07	5.26E+06	1.34E+07	1.56E+07	1.01E+07	2.32E-01
P00505;P00505-2	8.86E+06	0.00E+00	7.87E+06	1.07E+07	0.00E+00	0.00E+00	0.00E+00	0.00E+00	7.94E+06	0.00E+00	0.00E+00	0.00E+00	0.00E+00	0.00E+00	0.00E+00	0.00E+00	2.21E+06	3.43E+06	9.92E+05	4.00E+06	4.80E+06	2.81E+06	2.35E-01
P55084-2;P55084;F5GZQ3;B5MD38	0.00E+00	1.87E+07	1.41E+07	1.74E+07	2.77E+07	0.00E+00	4.94E+07	3.55E+07	1.38E+07	1.38E+07	1.60E+07	0.00E+00	1.67E+07	1.87E+07	0.00E+00	1.87E+07	1.63E+07	2.03E+07	1.22E+07	1.34E+07	1.69E+07	7.76E+06	2.36E-01
P43155-3;P43155-2;P43155	0.00E+00	9.49E+06	0.00E+00	0.00E+00	9.52E+06	9.58E+06	0.00E+00	1.31E+07	0.00E+00	0.00E+00	0.00E+00	0.00E+00	5.21E+06	6.57E+06	0.00E+00	7.05E+06	3.78E+06	5.22E+06	2.35E+06	4.73E+06	5.70E+06	3.29E+06	2.39E-01
P40925;P40925-3;B9A041;C9JF79;P40925-2;B8ZZ51	8.35E+07	9.60E+07	7.08E+07	7.72E+07	1.32E+08	8.78E+07	8.68E+07	9.56E+07	4.09E+07	1.47E+08	0.00E+00	2.25E+07	1.13E+08	1.24E+08	0.00E+00	7.31E+07	7.81E+07	9.12E+07	6.49E+07	4.36E+07	1.85E+07	5.77E+07	2.40E-01
P16152;P16152-2;E9PQ63;A8MTM1	0.00E+00	1.82E+07	1.60E+07	1.34E+07	1.58E+07	0.00E+00	1.70E+07	1.09E+07	0.00E+00	0.00E+00	1.28E+07	0.00E+00	1.53E+07	0.00E+00	1.38E+07	1.36E+07	9.17E+06	1.14E+07	6.92E+06	7.52E+06	7.39E+06	7.43E+06	2.46E-01
P60842;J3KT12;P60842-2;J3KTB5;J3QS69;J3QL43;J3QR64;J3KSZ0	4.41E+07	3.77E+07	4.60E+07	7.30E+07	5.24E+07	5.80E+07	2.53E+07	3.80E+07	6.75E+07	5.57E+07	0.00E+00	5.27E+07	3.06E+07	0.00E+00	4.61E+07	2.30E+07	4.06E+07	4.68E+07	3.44E+07	2.10E+07	1.45E+07	2.54E+07	2.52E-01
P26640	1.91E+07	1.82E+07	0.00E+00	0.00E+00	1.31E+07	1.80E+07	0.00E+00	1.52E+07	0.00E+00	0.00E+00	0.00E+00	1.42E+07	1.33E+07	1.01E+07	0.00E+00	9.01E+06	8.14E+06	1.04E+07	5.82E+06	7.85E+06	8.85E+06	6.43E+06	2.52E-01
P00367;P00367-3;P00367-2;P49448	7.85E+07	6.79E+07	6.40E+07	1.73E+07	7.17E+07	6.31E+07	3.59E+07	5.63E+07	7.61E+06	5.37E+06	1.21E+07	4.49E+07	8.53E+07	1.99E+07	5.43E+07	9.05E+07	4.84E+07	5.68E+07	4.00E+07	2.86E+07	2.04E+07	3.44E+07	2.53E-01
P24534	0.00E+00	0.00E+00	0.00E+00	0.00E+00	0.00E+00	0.00E+00	5.67E+06	0.00E+00	0.00E+00	0.00E+00	0.00E+00	0.00E+00	0.00E+00	0.00E+00	1.55E+07	1.45E+07	2.23E+06	7.09E+05	3.75E+06	5.19E+06	2.01E+06	6.95E+06	2.54E-01

P63244;J3KPE3;H0YAF8;D6RAC2;D6RHH4;H0Y8W2;D6R9Z1;H0YAM7;D6R9L0;D6REE5;D6RFX4	0.00E+00	1.81E+07	1.69E+07	2.71E+07	4.45E+07	0.00E+00	2.36E+07	0.00E+00	1.93E+07	3.37E+07	2.95E+07	1.85E+07	3.11E+07	0.00E+00	3.70E+07	2.77E+07	2.04E+07	1.63E+07	2.46E+07	1.42E+07	1.59E+07	1.19E+07	2.54E-01
O15382-2;M0QZP4;B3KSI3;O15382;M0QZ10;M0QXF9	0.00E+00	0.00E+00	0.00E+00	7.60E+05	0.00E+00	8.98E+05	0.00E+00	0.00E+00	6.91E+05	6.56E+05	6.91E+05	5.47E+05	0.00E+00	0.00E+00	0.00E+00	8.53E+05	3.19E+05	2.07E+05	4.30E+05	3.81E+05	3.85E+05	3.66E+05	2.55E-01
P50991;P50991-2	0.00E+00	0.00E+00	0.00E+00	0.00E+00	1.29E+07	0.00E+00	8.39E+06	1.34E+07	1.52E+07	1.34E+07	2.85E+07	0.00E+00	0.00E+00	0.00E+00	0.00E+00	2.05E+07	7.02E+06	4.33E+06	9.70E+06	9.21E+06	6.16E+06	1.13E+07	2.57E-01
Q53GS9-2;A0A087X1B2;Q53GS9-3;B9A018;Q53GS9	1.38E+08	1.35E+08	9.95E+07	0.00E+00	0.00E+00	1.19E+08	1.28E+08	7.65E+07	0.00E+00	0.00E+00	0.00E+00	6.15E+07	1.70E+08	0.00E+00	9.47E+07	8.53E+07	6.93E+07	8.71E+07	5.15E+07	6.11E+07	5.74E+07	6.31E+07	2.58E-01
Q15393	8.30E+07	9.49E+07	9.18E+07	8.74E+07	1.09E+08	0.00E+00	5.98E+07	6.47E+07	3.20E+07	6.46E+07	6.95E+07	3.59E+07	8.91E+07	3.67E+07	7.23E+07	5.87E+07	6.55E+07	7.38E+07	5.73E+07	2.83E+07	3.37E+07	2.06E+07	2.59E-01
P46781;B5MCT8;C9JM19	1.76E+07	3.93E+07	3.69E+07	0.00E+00	2.89E+07	1.91E+07	1.87E+07	1.96E+07	0.00E+00	0.00E+00	0.00E+00	4.06E+07	2.89E+07	5.46E+06	2.19E+07	1.72E+07	1.84E+07	2.25E+07	1.43E+07	1.42E+07	1.25E+07	1.54E+07	2.60E-01
Q08211	2.02E+07	0.00E+00	0.00E+00	2.27E+07	2.32E+07	1.92E+07	0.00E+00	1.34E+07	2.36E+07	1.75E+07	1.93E+07	1.71E+07	1.49E+07	1.68E+07	1.14E+07	1.53E+07	1.47E+07	1.23E+07	1.70E+07	8.03E+06	1.06E+07	3.55E+06	2.61E-01
P62258;P62258-2	0.00E+00	0.00E+00	0.00E+00	0.00E+00	0.00E+00	0.00E+00	0.00E+00	0.00E+00	0.00E+00	0.00E+00	0.00E+00	0.00E+00	2.96E+06	0.00E+00	1.87E+07	0.00E+00	1.36E+06	0.00E+00	2.71E+06	4.69E+06	0.00E+00	6.56E+06	2.62E-01
O95831-3;O95831;O95831-6;O95831-4	0.00E+00	0.00E+00	0.00E+00	0.00E+00	0.00E+00	8.90E+06	5.74E+07	0.00E+00	0.00E+00	0.00E+00	0.00E+00	0.00E+00	0.00E+00	0.00E+00	0.00E+00	0.00E+00	4.14E+06	8.29E+06	0.00E+00	1.44E+07	2.01E+07	0.00E+00	2.63E-01
Q9BTT0-3;Q9BTT0;E9PPH5;Q5TB19;E9PLC4	5.77E+06	1.29E+07	7.94E+06	0.00E+00	7.48E+06	7.64E+06	0.00E+00	7.62E+06	0.00E+00	4.73E+06	3.90E+06	0.00E+00	0.00E+00	6.67E+06	8.42E+06	7.43E+06	5.03E+06	6.17E+06	3.89E+06	3.99E+06	4.32E+06	3.52E+06	2.68E-01
A6NCN2	0.00E+00	0.00E+00	1.13E+07	0.00E+00	0.00E+00	0.00E+00	0.00E+00	0.00E+00	1.03E+07	1.18E+07	0.00E+00	0.00E+00	0.00E+00	1.31E+07	0.00E+00	0.00E+00	2.91E+06	1.42E+06	4.40E+06	5.22E+06	4.00E+06	6.11E+06	2.68E-01
Q15046;Q15046-2	2.61E+07	3.93E+07	2.29E+07	3.69E+07	4.84E+07	4.34E+07	0.00E+00	2.28E+07	2.44E+07	4.04E+07	2.84E+07	2.30E+07	0.00E+00	2.18E+07	1.63E+07	2.34E+07	2.61E+07	3.00E+07	2.22E+07	1.37E+07	1.54E+07	1.14E+07	2.72E-01
Q9P2E9-3;Q9P2E9-2;Q9P2E9;A0A0A0MRV0;A0A087WVV2;F8W7S5	2.20E+07	0.00E+00	0.00E+00	3.51E+07	0.00E+00	0.00E+00	0.00E+00	0.00E+00	3.65E+07	2.16E+07	0.00E+00	2.09E+07	0.00E+00	2.25E+07	0.00E+00	1.75E+07	1.10E+07	7.13E+06	1.49E+07	1.37E+07	1.37E+07	1.35E+07	2.73E-01
P52272-2;P52272;A0A087X0X3	5.12E+06	0.00E+00	0.00E+00	0.00E+00	0.00E+00	0.00E+00	0.00E+00	0.00E+00	0.00E+00	0.00E+00	0.00E+00	5.79E+06	5.30E+06	0.00E+00	4.53E+06	0.00E+00	1.30E+06	6.39E+05	1.95E+06	2.33E+06	1.81E+06	2.72E+06	2.74E-01
Q9HDC9;Q9HDC9-2;H0Y512	8.70E+07	5.70E+07	1.36E+08	5.56E+07	1.10E+08	4.54E+07	0.00E+00	7.83E+07	3.69E+07	6.63E+07	8.54E+07	4.33E+07	4.57E+07	3.30E+07	5.09E+07	6.24E+07	6.20E+07	7.11E+07	5.30E+07	3.22E+07	4.16E+07	1.74E+07	2.75E-01
O43399;O43399-5;A0A087WYR3;O43399-7;O43399-6;O43399-2;O43399-4;O43399-3;A0A087WZ51	0.00E+00	0.00E+00	0.00E+00	0.00E+00	0.00E+00	0.00E+00	5.45E+06	5.06E+06	0.00E+00	0.00E+00	0.00E+00	0.00E+00	3.99E+06	6.64E+06	5.14E+06	8.29E+06	2.16E+06	1.31E+06	3.01E+06	3.01E+06	2.44E+06	3.44E+06	2.75E-01
P15311;E7EQR4	2.27E+07	2.40E+07	2.15E+07	0.00E+00	1.70E+07	1.95E+07	0.00E+00	4.95E+07	0.00E+00	0.00E+00	0.00E+00	1.51E+07	2.31E+07	1.89E+07	1.91E+07	1.89E+07	1.56E+07	1.93E+07	1.19E+07	1.32E+07	1.56E+07	1.01E+07	2.80E-01
Q9BXP5-5;Q9BXP5-4;Q9BXP5-2;Q9BXP5-3;Q9BXP5;H7C3A1	0.00E+00	0.00E+00	0.00E+00	0.00E+00	3.93E+06	0.00E+00	0.00E+00	9.04E+06	0.00E+00	7.43E+06	0.00E+00	0.00E+00	0.00E+00	3.99E+06	1.45E+07	6.67E+06	2.84E+06	1.62E+06	4.07E+06	4.41E+06	3.30E+06	5.24E+06	2.82E-01
A0A087WWU8;P06753-5;P06753-2;Q5HYB6;P06753-4;P06753-3;P06753-6;Q5VU61;D6R904;J3KN67;P06753	7.62E+07	1.10E+08	9.49E+07	8.78E+07	1.08E+08	8.71E+07	1.54E+08	1.26E+08	4.11E+07	1.04E+08	9.05E+07	1.93E+07	1.03E+08	5.32E+07	1.30E+08	1.44E+08	9.54E+07	1.05E+08	8.56E+07	3.59E+07	2.49E+07	4.38E+07	2.86E-01
Q9BXW7-2;Q9BXW7	0.00E+00	1.34E+07	1.62E+07	0.00E+00	0.00E+00	7.40E+06	1.70E+07	1.93E+07	0.00E+00	6.32E+06	0.00E+00	8.23E+06	9.92E+06	0.00E+00	7.37E+06	1.14E+07	7.29E+06	9.17E+06	5.41E+06	6.84E+06	8.35E+06	4.74E+06	2.87E-01
P49327	0.00E+00	0.00E+00	0.00E+00	0.00E+00	0.00E+00	0.00E+00	1.07E+07	1.19E+07	1.06E+07	1.20E+07	0.00E+00	1.65E+07	0.00E+00	0.00E+00	0.00E+00	9.73E+06	4.46E+06	2.82E+06	6.10E+06	6.11E+06	5.24E+06	6.81E+06	2.99E-01
P32119;A6NIW5	0.00E+00	0.00E+00	7.04E+06	0.00E+00	0.00E+00	5.09E+06	0.00E+00	0.00E+00	0.00E+00	0.00E+00	0.00E+00	1.43E+07	0.00E+00	6.89E+06	0.00E+00	1.08E+07	2.76E+06	1.52E+06	4.00E+06	4.64E+06	2.86E+06	5.86E+06	3.00E-01
Q5JRX3;Q5JRX3-2;Q5JRX3-3	0.00E+00	1.44E+07	0.00E+00	0.00E+00	5.90E+06	0.00E+00	0.00E+00	0.00E+00	0.00E+00	1.40E+07	6.39E+06	0.00E+00	8.65E+06	0.00E+00	5.92E+06	7.22E+06	3.91E+06	2.54E+06	5.27E+06	5.15E+06	5.23E+06	5.02E+06	3.05E-01

P08574	0.00E+00	0.00E+00	0.00E+00	0.00E+00	0.00E+00	6.46E+06	0.00E+00	0.00E+00	0.00E+00	0.00E+00	0.00E+00	0.00E+00	0.00E+00	1.60E+07	0.00E+00	1.08E+07	2.08E+06	8.08E+05	3.35E+06	4.80E+06	2.28E+06	6.36E+06	3.05E-01
P22102	0.00E+00	1.67E+07	0.00E+00	0.00E+00	0.00E+00	1.41E+07	5.74E+08	0.00E+00	0.00E+00	0.00E+00	0.00E+00	0.00E+00	0.00E+00	0.00E+00	0.00E+00	0.00E+00	3.78E+07	7.56E+07	0.00E+00	1.43E+08	2.01E+08	0.00E+00	3.07E-01
Q16629-3;Q16629-2;Q16629-4;C9JAB2;Q16629	1.33E+07	0.00E+00	0.00E+00	2.09E+07	1.67E+07	2.41E+07	1.90E+07	2.07E+07	1.54E+07	1.81E+07	0.00E+00	1.66E+07	0.00E+00	1.28E+07	0.00E+00	1.47E+07	1.20E+07	1.43E+07	9.69E+06	8.83E+06	9.39E+06	8.17E+06	3.10E-01
Q15323;O76009	0.00E+00	1.76E+07	0.00E+00	0.00E+00	0.00E+00	0.00E+00	4.34E+08	0.00E+00	0.00E+00	0.00E+00	0.00E+00	0.00E+00	0.00E+00	0.00E+00	0.00E+00	0.00E+00	2.82E+07	5.65E+07	0.00E+00	1.08E+08	1.53E+08	0.00E+00	3.13E-01
P42765;K7EME0	0.00E+00	0.00E+00	0.00E+00	0.00E+00	0.00E+00	0.00E+00	0.00E+00	4.48E+06	0.00E+00	0.00E+00	0.00E+00	0.00E+00	7.65E+06	0.00E+00	0.00E+00	9.64E+06	1.36E+06	5.60E+05	2.16E+06	3.07E+06	1.58E+06	4.04E+06	3.14E-01
P78386;F5GYI5	0.00E+00	0.00E+00	3.48E+06	0.00E+00	0.00E+00	0.00E+00	0.00E+00	2.03E+06	1.35E+06	0.00E+00	0.00E+00	0.00E+00	0.00E+00	0.00E+00	0.00E+00	0.00E+00	4.29E+05	6.89E+05	1.68E+05	1.00E+06	1.33E+06	4.76E+05	3.16E-01
B1ALA9;P60891-2;P60891;P21108;P11908;P11908-2;B7ZB02;H7C540	2.43E+07	4.18E+07	0.00E+00	0.00E+00	3.78E+07	0.00E+00	4.69E+07	2.66E+07	2.41E+07	3.06E+07	2.46E+07	2.83E+07	4.23E+07	2.00E+07	3.40E+07	3.52E+07	2.60E+07	2.22E+07	2.99E+07	1.49E+07	1.98E+07	7.19E+06	3.18E-01
P50995-2;P50995	0.00E+00	0.00E+00	0.00E+00	0.00E+00	0.00E+00	0.00E+00	4.90E+06	3.39E+06	0.00E+00	0.00E+00	0.00E+00	0.00E+00	2.15E+06	0.00E+00	0.00E+00	0.00E+00	6.52E+05	1.04E+06	2.69E+05	1.49E+06	1.96E+06	7.60E+05	3.19E-01
Q13200;Q13200-3;Q13200-2	0.00E+00	1.88E+07	1.50E+07	1.02E+07	1.65E+07	9.49E+06	0.00E+00	1.22E+07	0.00E+00	0.00E+00	0.00E+00	7.65E+06	1.08E+07	0.00E+00	1.84E+07	1.51E+07	8.39E+06	1.03E+07	6.49E+06	7.36E+06	7.07E+06	7.60E+06	3.20E-01
Q16795	8.26E+06	1.74E+07	1.69E+07	1.96E+07	3.11E+07	1.32E+07	1.12E+07	2.02E+07	1.42E+07	2.62E+07	1.13E+07	8.22E+06	6.98E+06	0.00E+00	1.74E+07	2.20E+07	1.53E+07	1.72E+07	1.33E+07	7.78E+06	6.96E+06	8.51E+06	3.28E-01
P26038	9.12E+06	1.67E+07	1.04E+07	0.00E+00	1.00E+07	0.00E+00	9.70E+06	1.46E+07	6.63E+06	1.36E+07	9.55E+06	0.00E+00	2.17E+07	1.04E+07	2.03E+07	1.49E+07	1.05E+07	8.82E+06	1.21E+07	6.61E+06	6.04E+06	7.13E+06	3.32E-01
P40227;P40227-2	0.00E+00	0.00E+00	0.00E+00	0.00E+00	4.17E+06	0.00E+00	0.00E+00	0.00E+00	0.00E+00	0.00E+00	0.00E+00	0.00E+00	0.00E+00	0.00E+00	0.00E+00	0.00E+00	2.60E+05	5.21E+05	0.00E+00	1.04E+06	1.47E+06	0.00E+00	3.34E-01
O75694-2;O75694;E9PF10	0.00E+00	0.00E+00	0.00E+00	0.00E+00	0.00E+00	0.00E+00	0.00E+00	0.00E+00	0.00E+00	0.00E+00	0.00E+00	0.00E+00	0.00E+00	0.00E+00	2.71E+06	0.00E+00	1.70E+05	0.00E+00	3.39E+05	6.79E+05	0.00E+00	9.60E+05	3.34E-01
P06493;A0A024QZP7;A0A087WY43;E5RIU6;A0A087WZZ9;P06493-2	0.00E+00	0.00E+00	0.00E+00	1.41E+06	0.00E+00	0.00E+00	0.00E+00	0.00E+00	0.00E+00	0.00E+00	0.00E+00	0.00E+00	0.00E+00	0.00E+00	0.00E+00	0.00E+00	8.79E+04	1.76E+05	0.00E+00	3.52E+05	4.97E+05	0.00E+00	3.34E-01
P35237;A0A024QZX5;A0A087X1N8	3.84E+06	0.00E+00	0.00E+00	0.00E+00	0.00E+00	0.00E+00	0.00E+00	0.00E+00	0.00E+00	0.00E+00	0.00E+00	0.00E+00	0.00E+00	0.00E+00	0.00E+00	0.00E+00	2.40E+05	4.80E+05	0.00E+00	9.59E+05	1.36E+06	0.00E+00	3.34E-01
Q9H4M9;A0A024R571;C9JDQ8;C9J2Z4;C9JC03;Q9NZN3	0.00E+00	0.00E+00	0.00E+00	2.61E+08	0.00E+00	0.00E+00	0.00E+00	0.00E+00	0.00E+00	0.00E+00	0.00E+00	0.00E+00	0.00E+00	0.00E+00	0.00E+00	0.00E+00	1.63E+07	3.26E+07	0.00E+00	6.52E+07	9.22E+07	0.00E+00	3.34E-01
A0A075B6F6;Q8TCT9-5;Q8TCT9;Q8TCT9-4;Q8TCT9-2	0.00E+00	0.00E+00	0.00E+00	0.00E+00	0.00E+00	0.00E+00	0.00E+00	0.00E+00	0.00E+00	8.28E+05	0.00E+00	0.00E+00	0.00E+00	0.00E+00	0.00E+00	0.00E+00	5.17E+04	0.00E+00	1.03E+05	2.07E+05	0.00E+00	2.93E+05	3.34E-01
P11532-3;P11532-2;P11532-4;A0A087WV90;E9PDN5;A0A087WTU7;P11532;A0A075B6G3	0.00E+00	0.00E+00	0.00E+00	0.00E+00	1.33E+07	0.00E+00	0.00E+00	0.00E+00	0.00E+00	0.00E+00	0.00E+00	0.00E+00	0.00E+00	0.00E+00	0.00E+00	0.00E+00	8.29E+05	1.66E+06	0.00E+00	3.32E+06	4.69E+06	0.00E+00	3.34E-01
E9PKG6;V9HW75;P80303-2;P80303;A0A087WSV8;H0YD18;H0YEG8;Q2L696	0.00E+00	0.00E+00	0.00E+00	0.00E+00	0.00E+00	0.00E+00	0.00E+00	0.00E+00	0.00E+00	0.00E+00	0.00E+00	0.00E+00	0.00E+00	0.00E+00	0.00E+00	2.75E+05	1.72E+04	0.00E+00	3.44E+04	6.88E+04	0.00E+00	9.73E+04	3.34E-01
E9PIR7;F8W809;Q16881-5;Q16881-2;A0A087WSW9;Q16881-4;E9PNQ6;Q16881-3;Q16881-6;Q16881;A0A087WSY9;E2QRB9;Q16881-7	0.00E+00	0.00E+00	0.00E+00	0.00E+00	0.00E+00	0.00E+00	4.52E+06	0.00E+00	0.00E+00	0.00E+00	0.00E+00	0.00E+00	0.00E+00	0.00E+00	0.00E+00	0.00E+00	2.83E+05	5.65E+05	0.00E+00	1.13E+06	1.60E+06	0.00E+00	3.34E-01
A0A087WT34;A0A087X1H1;A0A0A0MTS6;K7EIE8;O95983-2;O95983	0.00E+00	0.00E+00	0.00E+00	0.00E+00	0.00E+00	0.00E+00	0.00E+00	0.00E+00	0.00E+00	0.00E+00	0.00E+00	1.40E+06	0.00E+00	0.00E+00	0.00E+00	0.00E+00	8.74E+04	0.00E+00	1.75E+05	3.50E+05	0.00E+00	4.94E+05	3.34E-01
A0A087WUE9;Q92797	0.00E+00	1.59E+07	0.00E+00	0.00E+00	0.00E+00	0.00E+00	0.00E+00	0.00E+00	0.00E+00	0.00E+00	0.00E+00	0.00E+00	0.00E+00	0.00E+00	0.00E+00	0.00E+00	9.93E+05	1.99E+06	0.00E+00	3.97E+06	5.62E+06	0.00E+00	3.34E-01

O14979-3;O14979-2;A0A087WUK2;O14979	0.00E+00	2.95E+06	0.00E+00	0.00E+00	0.00E+00	0.00E+00	0.00E+00	0.00E+00	0.00E+00	0.00E+00	0.00E+00	0.00E+00	0.00E+00	0.00E+00	0.00E+00	0.00E+00	1.84E+05	3.68E+05	0.00E+00	7.36E+05	1.04E+06	0.00E+00	3.34E-01
O60841;A0A087WUT6	0.00E+00	0.00E+00	0.00E+00	0.00E+00	0.00E+00	0.00E+00	0.00E+00	0.00E+00	0.00E+00	0.00E+00	0.00E+00	0.00E+00	0.00E+00	3.04E+06	0.00E+00	0.00E+00	1.90E+05	0.00E+00	3.80E+05	7.60E+05	0.00E+00	1.07E+06	3.34E-01
A0A087WV66;P46013;P46013-2	0.00E+00	0.00E+00	0.00E+00	0.00E+00	0.00E+00	0.00E+00	0.00E+00	0.00E+00	0.00E+00	0.00E+00	0.00E+00	0.00E+00	0.00E+00	1.24E+06	0.00E+00	0.00E+00	7.72E+04	0.00E+00	1.54E+05	3.09E+05	0.00E+00	4.37E+05	3.34E-01
A0A087WVC1;A0A087WVB0;Q9BQ39	0.00E+00	0.00E+00	0.00E+00	0.00E+00	0.00E+00	0.00E+00	4.66E+06	0.00E+00	0.00E+00	0.00E+00	0.00E+00	0.00E+00	0.00E+00	0.00E+00	0.00E+00	0.00E+00	2.91E+05	5.83E+05	0.00E+00	1.17E+06	1.65E+06	0.00E+00	3.34E-01
Q5JPE7-3;A0A087WW46;Q5JPE7-2;Q15155;P69849;Q5JPE7;J3KN36;A0A087X117	0.00E+00	0.00E+00	0.00E+00	0.00E+00	0.00E+00	0.00E+00	7.70E+05	0.00E+00	0.00E+00	0.00E+00	0.00E+00	0.00E+00	0.00E+00	0.00E+00	0.00E+00	0.00E+00	4.82E+04	9.63E+04	0.00E+00	1.93E+05	2.72E+05	0.00E+00	3.34E-01
Q99460-2;Q99460;A0A087WW66;H7C378	0.00E+00	0.00E+00	0.00E+00	0.00E+00	0.00E+00	0.00E+00	0.00E+00	1.45E+06	0.00E+00	0.00E+00	0.00E+00	0.00E+00	0.00E+00	0.00E+00	0.00E+00	0.00E+00	9.07E+04	1.81E+05	0.00E+00	3.63E+05	5.13E+05	0.00E+00	3.34E-01
A0A087WX58;K7EPS6;Q7Z4V5-2;Q7Z4V5	0.00E+00	0.00E+00	0.00E+00	0.00E+00	0.00E+00	0.00E+00	0.00E+00	0.00E+00	0.00E+00	0.00E+00	0.00E+00	0.00E+00	0.00E+00	0.00E+00	0.00E+00	1.20E+07	7.50E+05	0.00E+00	1.50E+06	3.00E+06	0.00E+00	4.24E+06	3.34E-01
O95299;A0A087WXC5;E7ESZ7;O95299-2;Q8N1B9;C9J6X0	0.00E+00	0.00E+00	0.00E+00	0.00E+00	0.00E+00	0.00E+00	0.00E+00	0.00E+00	0.00E+00	7.47E+06	0.00E+00	0.00E+00	0.00E+00	0.00E+00	0.00E+00	0.00E+00	4.67E+05	0.00E+00	9.34E+05	1.87E+06	0.00E+00	2.64E+06	3.34E-01
P18564-2;A0A087WXP3;E9PEE8;P18564	0.00E+00	0.00E+00	0.00E+00	0.00E+00	0.00E+00	0.00E+00	0.00E+00	5.18E+05	0.00E+00	0.00E+00	0.00E+00	0.00E+00	0.00E+00	0.00E+00	0.00E+00	0.00E+00	3.23E+04	6.47E+04	0.00E+00	1.29E+05	1.83E+05	0.00E+00	3.34E-01
A0A087WXS7;O43681	0.00E+00	0.00E+00	0.00E+00	0.00E+00	0.00E+00	0.00E+00	0.00E+00	0.00E+00	0.00E+00	0.00E+00	3.97E+05	0.00E+00	0.00E+00	0.00E+00	0.00E+00	0.00E+00	2.48E+04	0.00E+00	4.97E+04	9.93E+04	0.00E+00	1.40E+05	3.34E-01
A0A087WY55;Q9NP79	0.00E+00	0.00E+00	0.00E+00	0.00E+00	0.00E+00	0.00E+00	1.06E+06	0.00E+00	0.00E+00	0.00E+00	0.00E+00	0.00E+00	0.00E+00	0.00E+00	0.00E+00	0.00E+00	6.61E+04	1.32E+05	0.00E+00	2.64E+05	3.74E+05	0.00E+00	3.34E-01
Q14980-4;Q14980-3;A0A087WY61;Q14980-2;Q14980;H0YFY6	0.00E+00	0.00E+00	0.00E+00	0.00E+00	0.00E+00	0.00E+00	1.86E+06	0.00E+00	0.00E+00	0.00E+00	0.00E+00	0.00E+00	0.00E+00	0.00E+00	0.00E+00	0.00E+00	1.16E+05	2.33E+05	0.00E+00	4.66E+05	6.59E+05	0.00E+00	3.34E-01
Q6ICJ4;A0A087WY67;P0CG30;P0CG29	0.00E+00	0.00E+00	0.00E+00	0.00E+00	0.00E+00	0.00E+00	6.34E+05	0.00E+00	0.00E+00	0.00E+00	0.00E+00	0.00E+00	0.00E+00	0.00E+00	0.00E+00	0.00E+00	3.96E+04	7.92E+04	0.00E+00	1.58E+05	2.24E+05	0.00E+00	3.34E-01
H0Y5G9;G3V1R5;A0A087WYK8;O43847;O43847-2;B1AKJ5	0.00E+00	0.00E+00	0.00E+00	8.85E+06	0.00E+00	0.00E+00	0.00E+00	0.00E+00	0.00E+00	0.00E+00	0.00E+00	0.00E+00	0.00E+00	0.00E+00	0.00E+00	0.00E+00	5.53E+05	1.11E+06	0.00E+00	2.21E+06	3.13E+06	0.00E+00	3.34E-01
Q7Z478;A0A087WYN9	0.00E+00	0.00E+00	0.00E+00	0.00E+00	0.00E+00	0.00E+00	0.00E+00	0.00E+00	0.00E+00	0.00E+00	0.00E+00	0.00E+00	0.00E+00	0.00E+00	5.22E+06	0.00E+00	3.26E+05	0.00E+00	6.53E+05	1.31E+06	0.00E+00	1.85E+06	3.34E-01
A0A087WYW6;Q96A33-2;Q96A33	0.00E+00	0.00E+00	0.00E+00	0.00E+00	0.00E+00	0.00E+00	0.00E+00	0.00E+00	0.00E+00	0.00E+00	0.00E+00	0.00E+00	0.00E+00	0.00E+00	0.00E+00	1.33E+06	8.30E+04	0.00E+00	1.66E+05	3.32E+05	0.00E+00	4.70E+05	3.34E-01
E5RJT0;A0A087WZK9;O15372;B3KS98	0.00E+00	0.00E+00	0.00E+00	0.00E+00	0.00E+00	0.00E+00	0.00E+00	0.00E+00	0.00E+00	0.00E+00	0.00E+00	0.00E+00	1.85E+06	0.00E+00	0.00E+00	0.00E+00	1.16E+05	0.00E+00	2.31E+05	4.63E+05	0.00E+00	6.54E+05	3.34E-01
A0A087X2E5;O43837-2;O43837;A0A087WZN1;O43837-3	0.00E+00	0.00E+00	0.00E+00	4.72E+06	0.00E+00	0.00E+00	0.00E+00	0.00E+00	0.00E+00	0.00E+00	0.00E+00	0.00E+00	0.00E+00	0.00E+00	0.00E+00	0.00E+00	2.95E+05	5.90E+05	0.00E+00	1.18E+06	1.67E+06	0.00E+00	3.34E-01
M0R061;O14579-3;O14579-2;A0A087X0I4;O14579;M0QXB4	0.00E+00	0.00E+00	0.00E+00	0.00E+00	0.00E+00	0.00E+00	0.00E+00	0.00E+00	0.00E+00	0.00E+00	0.00E+00	0.00E+00	0.00E+00	1.86E+06	0.00E+00	0.00E+00	1.16E+05	0.00E+00	2.32E+05	4.65E+05	0.00E+00	6.57E+05	3.34E-01
A0A087X0K1;Q9Y376	0.00E+00	0.00E+00	0.00E+00	0.00E+00	0.00E+00	0.00E+00	0.00E+00	0.00E+00	0.00E+00	3.96E+06	0.00E+00	0.00E+00	0.00E+00	0.00E+00	0.00E+00	0.00E+00	2.47E+05	0.00E+00	4.95E+05	9.90E+05	0.00E+00	1.40E+06	3.34E-01
A0A087X176;Q6ZRP7;H0Y430	0.00E+00	0.00E+00	0.00E+00	0.00E+00	0.00E+00	0.00E+00	0.00E+00	1.14E+06	0.00E+00	0.00E+00	0.00E+00	0.00E+00	0.00E+00	0.00E+00	0.00E+00	0.00E+00	7.12E+04	1.42E+05	0.00E+00	2.85E+05	4.03E+05	0.00E+00	3.34E-01

H7C3I1;F6VDH7;Q3KNR6;Q8IZP2;A0A087X1H6;P50502;Q8NFI4	0.00E+00	0.00E+00	0.00E+00	0.00E+00	0.00E+00	0.00E+00	0.00E+00	0.00E+00	0.00E+00	0.00E+00	0.00E+00	0.00E+00	1.94E+05	0.00E+00	0.00E+00	0.00E+00	1.21E+04	0.00E+00	2.42E+04	4.85E+04	0.00E+00	6.85E+04	3.34E-01
A0A087X1K9;E5RGR0;O75608-2;O75608;E5RJ48	0.00E+00	0.00E+00	0.00E+00	0.00E+00	0.00E+00	0.00E+00	0.00E+00	0.00E+00	0.00E+00	0.00E+00	0.00E+00	0.00E+00	0.00E+00	0.00E+00	0.00E+00	4.37E+06	2.73E+05	0.00E+00	5.46E+05	1.09E+06	0.00E+00	1.55E+06	3.34E-01
J3KQV6;J3QT67;J3QT66;J3QT69;J3QT50;J3QT43;J3QT95;A0A087X1P5;Q9H9Q2;Q9H9Q2-3;J3KQ41	0.00E+00	0.00E+00	0.00E+00	0.00E+00	0.00E+00	0.00E+00	0.00E+00	0.00E+00	0.00E+00	0.00E+00	0.00E+00	0.00E+00	3.97E+05	0.00E+00	0.00E+00	0.00E+00	2.48E+04	0.00E+00	4.96E+04	9.91E+04	0.00E+00	1.40E+05	3.34E-01
A0A087X1S2;P67809;H0Y449;C9J5V9	0.00E+00	0.00E+00	0.00E+00	0.00E+00	5.28E+06	0.00E+00	0.00E+00	0.00E+00	0.00E+00	0.00E+00	0.00E+00	0.00E+00	0.00E+00	0.00E+00	0.00E+00	0.00E+00	3.30E+05	6.60E+05	0.00E+00	1.32E+06	1.87E+06	0.00E+00	3.34E-01
A0A087X271;B4DDF4;Q99439;B4DUT8;K7ES69;A0A087X1X5;H3BVI6;Q99439-2	0.00E+00	0.00E+00	0.00E+00	0.00E+00	2.83E+05	0.00E+00	0.00E+00	0.00E+00	0.00E+00	0.00E+00	0.00E+00	0.00E+00	0.00E+00	0.00E+00	0.00E+00	0.00E+00	1.77E+04	3.54E+04	0.00E+00	7.08E+04	1.00E+05	0.00E+00	3.34E-01
Q14525;A0A087X2I6	0.00E+00	0.00E+00	0.00E+00	0.00E+00	1.17E+06	0.00E+00	0.00E+00	0.00E+00	0.00E+00	0.00E+00	0.00E+00	0.00E+00	0.00E+00	0.00E+00	0.00E+00	0.00E+00	7.32E+04	1.46E+05	0.00E+00	2.93E+05	4.14E+05	0.00E+00	3.34E-01
A0A096LNY5;P30566-2;P30566;A0A0A6YY92;A0A096LP92;B4DEP1;A0A096LPA2;A0A096LP72;A0A096LNY4;A0A096LNY6	0.00E+00	0.00E+00	0.00E+00	0.00E+00	0.00E+00	0.00E+00	0.00E+00	0.00E+00	0.00E+00	0.00E+00	1.49E+08	0.00E+00	0.00E+00	0.00E+00	0.00E+00	0.00E+00	9.32E+06	0.00E+00	1.86E+07	3.73E+07	0.00E+00	5.27E+07	3.34E-01
U3KQC1;K7EIR0;A0A0A0MQU0;Q9BV38	0.00E+00	0.00E+00	0.00E+00	0.00E+00	0.00E+00	0.00E+00	0.00E+00	0.00E+00	0.00E+00	0.00E+00	0.00E+00	0.00E+00	0.00E+00	0.00E+00	0.00E+00	7.61E+05	4.76E+04	0.00E+00	9.52E+04	1.90E+05	0.00E+00	2.69E+05	3.34E-01
H0YCI6;A0A0A0MQX4;Q96KG9-5;E9PPN3;Q96KG9-3;E9PS17;E9PK59;Q96KG9-4;Q96KG9-6;Q96KG9-2;Q96KG9	0.00E+00	0.00E+00	0.00E+00	0.00E+00	0.00E+00	0.00E+00	0.00E+00	0.00E+00	0.00E+00	0.00E+00	0.00E+00	0.00E+00	0.00E+00	0.00E+00	0.00E+00	5.67E+05	3.54E+04	0.00E+00	7.08E+04	1.42E+05	0.00E+00	2.00E+05	3.34E-01
H0YDK8;A0A0A0MR59;E9PR38;Q8TB72-2;Q8TB72-4;Q8TB72-3;Q8TB72;H0YEH2;Q14671-4;Q14671-2;Q14671;Q14671-3;Q5T1Z4;Q5T1Z8	0.00E+00	2.02E+06	0.00E+00	0.00E+00	0.00E+00	0.00E+00	0.00E+00	0.00E+00	0.00E+00	0.00E+00	0.00E+00	0.00E+00	0.00E+00	0.00E+00	0.00E+00	0.00E+00	1.26E+05	2.52E+05	0.00E+00	5.05E+05	7.14E+05	0.00E+00	3.34E-01
H3BRM6;B9ZVX7;P09488-2;Q03013-2;Q5T8R1;Q03013;P46439;P09488;A0A0A0MR85	0.00E+00	0.00E+00	0.00E+00	9.01E+06	0.00E+00	0.00E+00	0.00E+00	0.00E+00	0.00E+00	0.00E+00	0.00E+00	0.00E+00	0.00E+00	0.00E+00	0.00E+00	0.00E+00	5.63E+05	1.13E+06	0.00E+00	2.25E+06	3.19E+06	0.00E+00	3.34E-01
M0R3F1;Q9BUJ2-3;Q9BUJ2-4;A0A0A0MRA5;B7Z4B8;Q9BUJ2-2;Q9BUJ2;M0QYZ0	0.00E+00	0.00E+00	0.00E+00	0.00E+00	0.00E+00	0.00E+00	0.00E+00	0.00E+00	0.00E+00	0.00E+00	0.00E+00	0.00E+00	0.00E+00	0.00E+00	0.00E+00	4.21E+06	2.63E+05	0.00E+00	5.26E+05	1.05E+06	0.00E+00	1.49E+06	3.34E-01
G3V5X4;A0A0A0MRE3;Q8WXH0;Q8WXH0-2	0.00E+00	0.00E+00	0.00E+00	0.00E+00	0.00E+00	0.00E+00	0.00E+00	0.00E+00	0.00E+00	3.48E+05	0.00E+00	0.00E+00	0.00E+00	0.00E+00	0.00E+00	0.00E+00	2.18E+04	0.00E+00	4.35E+04	8.71E+04	0.00E+00	1.23E+05	3.34E-01
A0A0A0MRH6;Q5JRA6-2;Q5JRA6	0.00E+00	0.00E+00	0.00E+00	0.00E+00	0.00E+00	0.00E+00	0.00E+00	0.00E+00	0.00E+00	0.00E+00	0.00E+00	0.00E+00	0.00E+00	0.00E+00	0.00E+00	4.82E+06	3.01E+05	0.00E+00	6.02E+05	1.20E+06	0.00E+00	1.70E+06	3.34E-01
Q13505-3;Q13505;A0A0A0MRK6	0.00E+00	0.00E+00	0.00E+00	0.00E+00	0.00E+00	0.00E+00	0.00E+00	0.00E+00	0.00E+00	0.00E+00	0.00E+00	0.00E+00	7.84E+05	0.00E+00	0.00E+00	0.00E+00	4.90E+04	0.00E+00	9.80E+04	1.96E+05	0.00E+00	2.77E+05	3.34E-01

A0A0A0MS41;Q9BWM7;S4R3N9	0.00E+00	0.00E+00	5.92E+06	0.00E+00	0.00E+00	0.00E+00	0.00E+00	0.00E+00	0.00E+00	0.00E+00	0.00E+00	0.00E+00	0.00E+00	0.00E+00	0.00E+00	0.00E+00	3.70E+05	7.40E+05	0.00E+00	1.48E+06	2.09E+06	0.00E+00	3.34E-01
P13797-3;F8W8D8;P13797-2;A0A0A0MSQ0;P13797	0.00E+00	0.00E+00	0.00E+00	0.00E+00	0.00E+00	0.00E+00	0.00E+00	0.00E+00	0.00E+00	0.00E+00	0.00E+00	0.00E+00	0.00E+00	1.48E+06	0.00E+00	0.00E+00	9.25E+04	0.00E+00	1.85E+05	3.70E+05	0.00E+00	5.23E+05	3.34E-01
A0A0A0MT32;Q5T073;P38571-2;P38571	0.00E+00	0.00E+00	0.00E+00	0.00E+00	0.00E+00	0.00E+00	0.00E+00	0.00E+00	0.00E+00	0.00E+00	0.00E+00	0.00E+00	0.00E+00	0.00E+00	0.00E+00	6.14E+05	3.83E+04	0.00E+00	7.67E+04	1.53E+05	0.00E+00	2.17E+05	3.34E-01
A0A0A0MTI6;Q9NYP7;Q9NYP7-2;Q9NYP7-3	0.00E+00	0.00E+00	0.00E+00	0.00E+00	0.00E+00	0.00E+00	0.00E+00	0.00E+00	0.00E+00	0.00E+00	0.00E+00	0.00E+00	0.00E+00	0.00E+00	1.30E+05	0.00E+00	8.13E+03	0.00E+00	1.63E+04	3.25E+04	0.00E+00	4.60E+04	3.34E-01
A6NJA2;P54578-2;P54578-3;A0A0A6YYH3;P54578	0.00E+00	0.00E+00	0.00E+00	0.00E+00	0.00E+00	0.00E+00	0.00E+00	0.00E+00	5.50E+05	0.00E+00	0.00E+00	0.00E+00	0.00E+00	0.00E+00	0.00E+00	0.00E+00	3.43E+04	0.00E+00	6.87E+04	1.37E+05	0.00E+00	1.94E+05	3.34E-01
I3L276;I3L4P8;A0A0A6YYS0;I3L463;I3L159;I3L1F5;P30519;P30519-2;A0A087WT44	0.00E+00	0.00E+00	2.01E+06	0.00E+00	0.00E+00	0.00E+00	0.00E+00	0.00E+00	0.00E+00	0.00E+00	0.00E+00	0.00E+00	0.00E+00	0.00E+00	0.00E+00	0.00E+00	1.26E+05	2.52E+05	0.00E+00	5.04E+05	7.12E+05	0.00E+00	3.34E-01
H7BXI1;A0FGR8-2;A0FGR8;A0A087WXU3;A0FGR8-6	0.00E+00	0.00E+00	0.00E+00	0.00E+00	0.00E+00	0.00E+00	0.00E+00	0.00E+00	0.00E+00	0.00E+00	5.16E+05	0.00E+00	0.00E+00	0.00E+00	0.00E+00	0.00E+00	3.22E+04	0.00E+00	6.45E+04	1.29E+05	0.00E+00	1.82E+05	3.34E-01
A6NE76;Q13620-3;Q13620-1;K4DI93;Q13620;A0A087WWN2;Q13619-2;A0A0A0MR50;Q13619	0.00E+00	0.00E+00	0.00E+00	0.00E+00	0.00E+00	0.00E+00	0.00E+00	0.00E+00	0.00E+00	0.00E+00	0.00E+00	0.00E+00	0.00E+00	0.00E+00	0.00E+00	9.87E+05	6.17E+04	0.00E+00	1.23E+05	2.47E+05	0.00E+00	3.49E+05	3.34E-01
A6NHR9-2;A6NHR9	0.00E+00	2.10E+06	0.00E+00	0.00E+00	0.00E+00	0.00E+00	0.00E+00	0.00E+00	0.00E+00	0.00E+00	0.00E+00	0.00E+00	0.00E+00	0.00E+00	0.00E+00	0.00E+00	1.31E+05	2.63E+05	0.00E+00	5.25E+05	7.43E+05	0.00E+00	3.34E-01
A6NKB8;Q9H4A4	0.00E+00	0.00E+00	0.00E+00	0.00E+00	0.00E+00	0.00E+00	0.00E+00	0.00E+00	0.00E+00	0.00E+00	0.00E+00	0.00E+00	0.00E+00	0.00E+00	4.37E+06	0.00E+00	2.73E+05	0.00E+00	5.46E+05	1.09E+06	0.00E+00	1.54E+06	3.34E-01
F6WIT2;Q15257-3;Q15257-2;A6PVN5;Q15257;B7ZBQ0;A6PVN7;Q68CR8;A6PVN8;C9IZ76;A6PVN6;Q5T949;Q5T948;A6PVN9;Q15257-4	0.00E+00	0.00E+00	0.00E+00	0.00E+00	0.00E+00	0.00E+00	0.00E+00	0.00E+00	0.00E+00	0.00E+00	0.00E+00	0.00E+00	8.73E+05	0.00E+00	0.00E+00	0.00E+00	5.45E+04	0.00E+00	1.09E+05	2.18E+05	0.00E+00	3.09E+05	3.34E-01
F5H793;C9J7H8;A8MW50;P07195	0.00E+00	0.00E+00	0.00E+00	0.00E+00	0.00E+00	0.00E+00	0.00E+00	6.30E+06	0.00E+00	0.00E+00	0.00E+00	0.00E+00	0.00E+00	0.00E+00	0.00E+00	0.00E+00	3.94E+05	7.88E+05	0.00E+00	1.58E+06	2.23E+06	0.00E+00	3.34E-01
F8W808;A8MWP7;P41227-2;P41227	0.00E+00	0.00E+00	0.00E+00	0.00E+00	0.00E+00	0.00E+00	0.00E+00	0.00E+00	0.00E+00	0.00E+00	0.00E+00	0.00E+00	3.19E+05	0.00E+00	0.00E+00	0.00E+00	1.99E+04	0.00E+00	3.98E+04	7.97E+04	0.00E+00	1.13E+05	3.34E-01
A8MXV4	0.00E+00	0.00E+00	0.00E+00	0.00E+00	6.19E+06	0.00E+00	0.00E+00	0.00E+00	0.00E+00	0.00E+00	0.00E+00	0.00E+00	0.00E+00	0.00E+00	0.00E+00	0.00E+00	3.87E+05	7.74E+05	0.00E+00	1.55E+06	2.19E+06	0.00E+00	3.34E-01
J3QR71;J3QRI9;J3QKW7;A8MZF9;P55039	0.00E+00	0.00E+00	0.00E+00	0.00E+00	0.00E+00	1.56E+06	0.00E+00	0.00E+00	0.00E+00	0.00E+00	0.00E+00	0.00E+00	0.00E+00	0.00E+00	0.00E+00	0.00E+00	9.75E+04	1.95E+05	0.00E+00	3.90E+05	5.52E+05	0.00E+00	3.34E-01
Q9Y262-2;Q9Y262;B0QY89;H7C3A0;C9JHP4;C9K0Q7;B0QY90	0.00E+00	0.00E+00	0.00E+00	0.00E+00	0.00E+00	3.14E+06	0.00E+00	0.00E+00	0.00E+00	0.00E+00	0.00E+00	0.00E+00	0.00E+00	0.00E+00	0.00E+00	0.00E+00	1.96E+05	3.92E+05	0.00E+00	7.84E+05	1.11E+06	0.00E+00	3.34E-01
B0QYA5;O15371-2;O15371-3;O15371;B0QYA4;B0QYA8;B0QYA6;B0QYA7	0.00E+00	0.00E+00	0.00E+00	0.00E+00	0.00E+00	0.00E+00	0.00E+00	0.00E+00	0.00E+00	0.00E+00	0.00E+00	0.00E+00	2.24E+05	0.00E+00	0.00E+00	0.00E+00	1.40E+04	0.00E+00	2.80E+04	5.61E+04	0.00E+00	7.93E+04	3.34E-01
H7BY36;Q01844-2;Q01844-6;B0QYK0;C9JGE3;Q01844-3;Q01844;Q01844-5	0.00E+00	0.00E+00	0.00E+00	0.00E+00	0.00E+00	0.00E+00	0.00E+00	0.00E+00	0.00E+00	0.00E+00	0.00E+00	0.00E+00	0.00E+00	0.00E+00	0.00E+00	1.10E+06	6.89E+04	0.00E+00	1.38E+05	2.76E+05	0.00E+00	3.90E+05	3.34E-01
B1AHA8;P09601	0.00E+00	0.00E+00	0.00E+00	0.00E+00	0.00E+00	1.11E+07	0.00E+00	0.00E+00	0.00E+00	0.00E+00	0.00E+00	0.00E+00	0.00E+00	0.00E+00	0.00E+00	0.00E+00	6.95E+05	1.39E+06	0.00E+00	2.78E+06	3.93E+06	0.00E+00	3.34E-01
B1AJY5;B1AJY7;O75832;B1AJY6;O75832-2	0.00E+00	0.00E+00	0.00E+00	0.00E+00	0.00E+00	0.00E+00	0.00E+00	0.00E+00	0.00E+00	0.00E+00	0.00E+00	0.00E+00	0.00E+00	0.00E+00	0.00E+00	3.65E+06	2.28E+05	0.00E+00	4.57E+05	9.14E+05	0.00E+00	1.29E+06	3.34E-01
B1AKD8;Q5TZA2-2;Q5TZA2	0.00E+00	0.00E+00	0.00E+00	0.00E+00	1.60E+06	0.00E+00	0.00E+00	0.00E+00	0.00E+00	0.00E+00	0.00E+00	0.00E+00	0.00E+00	0.00E+00	0.00E+00	0.00E+00	1.00E+05	2.00E+05	0.00E+00	4.01E+05	5.67E+05	0.00E+00	3.34E-01

B1APM4;P35610-3;P35610-2;P35610	0.00E+00	0.00E+00	0.00E+00	0.00E+00	0.00E+00	0.00E+00	0.00E+00	0.00E+00	0.00E+00	0.00E+00	0.00E+00	1.61E+06	0.00E+00	0.00E+00	0.00E+00	0.00E+00	1.01E+05	0.00E+00	2.02E+05	4.04E+05	0.00E+00	5.71E+05	3.34E-01
B3KWE1;P12081-3;P12081-2;P12081-4;P12081;D6RJE6;E7ETE2;B4E1C5;B4DDD8;P49590-2;P49590	0.00E+00	0.00E+00	0.00E+00	2.01E+06	0.00E+00	0.00E+00	0.00E+00	0.00E+00	0.00E+00	0.00E+00	0.00E+00	0.00E+00	0.00E+00	0.00E+00	0.00E+00	0.00E+00	1.26E+05	2.51E+05	0.00E+00	5.02E+05	7.10E+05	0.00E+00	3.34E-01
B4DIP2;Q96RT1-7;Q96RT1-6;Q96RT1-4;Q96RT1-5;Q96RT1-3;Q96RT1-9;Q96RT1-2;Q96RT1;Q96RT1-8	0.00E+00	0.00E+00	0.00E+00	0.00E+00	0.00E+00	0.00E+00	1.02E+06	0.00E+00	0.00E+00	0.00E+00	0.00E+00	0.00E+00	0.00E+00	0.00E+00	0.00E+00	0.00E+00	6.37E+04	1.27E+05	0.00E+00	2.55E+05	3.60E+05	0.00E+00	3.34E-01
B4DJ81;P28331-3;P28331-4;P28331-5;P28331;P28331-2	0.00E+00	0.00E+00	0.00E+00	0.00E+00	0.00E+00	0.00E+00	4.91E+07	0.00E+00	0.00E+00	0.00E+00	0.00E+00	0.00E+00	0.00E+00	0.00E+00	0.00E+00	0.00E+00	3.07E+06	6.14E+06	0.00E+00	1.23E+07	1.74E+07	0.00E+00	3.34E-01
Q9UBX3;Q9UBX3-2;B4DLN1;I3L1E8;F6RGN5	0.00E+00	0.00E+00	0.00E+00	0.00E+00	0.00E+00	0.00E+00	0.00E+00	0.00E+00	0.00E+00	0.00E+00	0.00E+00	0.00E+00	0.00E+00	0.00E+00	0.00E+00	2.34E+06	1.46E+05	0.00E+00	2.92E+05	5.85E+05	0.00E+00	8.27E+05	3.34E-01
B8ZZF7;O00338;O00338-2;B4DLP0	0.00E+00	0.00E+00	0.00E+00	0.00E+00	0.00E+00	6.47E+06	0.00E+00	0.00E+00	0.00E+00	0.00E+00	0.00E+00	0.00E+00	0.00E+00	0.00E+00	0.00E+00	0.00E+00	4.04E+05	8.09E+05	0.00E+00	1.62E+06	2.29E+06	0.00E+00	3.34E-01
P61619;B4DR61;P61619-3	0.00E+00	0.00E+00	0.00E+00	0.00E+00	0.00E+00	1.43E+06	0.00E+00	0.00E+00	0.00E+00	0.00E+00	0.00E+00	0.00E+00	0.00E+00	0.00E+00	0.00E+00	0.00E+00	8.92E+04	1.78E+05	0.00E+00	3.57E+05	5.05E+05	0.00E+00	3.34E-01
P51114-2;B4DXZ6;P51114;E7EU85;E9PFF5;P51114-3	0.00E+00	0.00E+00	0.00E+00	0.00E+00	0.00E+00	0.00E+00	3.24E+06	0.00E+00	0.00E+00	0.00E+00	0.00E+00	0.00E+00	0.00E+00	0.00E+00	0.00E+00	0.00E+00	2.02E+05	4.05E+05	0.00E+00	8.10E+05	1.15E+06	0.00E+00	3.34E-01
B4DZG7;P40616-2;P40616;F8VYN9	0.00E+00	0.00E+00	0.00E+00	5.23E+06	0.00E+00	0.00E+00	0.00E+00	0.00E+00	0.00E+00	0.00E+00	0.00E+00	0.00E+00	0.00E+00	0.00E+00	0.00E+00	0.00E+00	3.27E+05	6.54E+05	0.00E+00	1.31E+06	1.85E+06	0.00E+00	3.34E-01
H7C3M2;B5MCW2;H7C422;G5E9G0;P39023	0.00E+00	0.00E+00	0.00E+00	0.00E+00	2.83E+06	0.00E+00	0.00E+00	0.00E+00	0.00E+00	0.00E+00	0.00E+00	0.00E+00	0.00E+00	0.00E+00	0.00E+00	0.00E+00	1.77E+05	3.54E+05	0.00E+00	7.07E+05	1.00E+06	0.00E+00	3.34E-01
P62495-2;B7Z7P8;P62495	0.00E+00	0.00E+00	0.00E+00	0.00E+00	0.00E+00	0.00E+00	0.00E+00	0.00E+00	0.00E+00	0.00E+00	0.00E+00	3.81E+05	0.00E+00	0.00E+00	0.00E+00	0.00E+00	2.38E+04	0.00E+00	4.76E+04	9.51E+04	0.00E+00	1.35E+05	3.34E-01
B7Z9I1;P11310;P11310-2;Q5T4U5	0.00E+00	0.00E+00	0.00E+00	1.45E+06	0.00E+00	0.00E+00	0.00E+00	0.00E+00	0.00E+00	0.00E+00	0.00E+00	0.00E+00	0.00E+00	0.00E+00	0.00E+00	0.00E+00	9.05E+04	1.81E+05	0.00E+00	3.62E+05	5.12E+05	0.00E+00	3.34E-01
E9PKN8;B7ZAG4;Q9Y2W6-3;Q5SZR4;Q9Y2W6;F6TB26	0.00E+00	0.00E+00	0.00E+00	0.00E+00	0.00E+00	0.00E+00	2.14E+07	0.00E+00	0.00E+00	0.00E+00	0.00E+00	0.00E+00	0.00E+00	0.00E+00	0.00E+00	0.00E+00	1.34E+06	2.67E+06	0.00E+00	5.34E+06	7.56E+06	0.00E+00	3.34E-01
B8ZZC5;B8ZZA8;O94925-3	0.00E+00	0.00E+00	0.00E+00	0.00E+00	0.00E+00	0.00E+00	0.00E+00	0.00E+00	0.00E+00	0.00E+00	0.00E+00	9.89E+05	0.00E+00	0.00E+00	0.00E+00	0.00E+00	6.18E+04	0.00E+00	1.24E+05	2.47E+05	0.00E+00	3.50E+05	3.34E-01
C4AM86;Q92764	0.00E+00	0.00E+00	0.00E+00	0.00E+00	3.85E+06	0.00E+00	0.00E+00	0.00E+00	0.00E+00	0.00E+00	0.00E+00	0.00E+00	0.00E+00	0.00E+00	0.00E+00	0.00E+00	2.41E+05	4.81E+05	0.00E+00	9.63E+05	1.36E+06	0.00E+00	3.34E-01
C9IY40;Q5T653	0.00E+00	0.00E+00	0.00E+00	0.00E+00	0.00E+00	0.00E+00	4.34E+05	0.00E+00	0.00E+00	0.00E+00	0.00E+00	0.00E+00	0.00E+00	0.00E+00	0.00E+00	0.00E+00	2.71E+04	5.42E+04	0.00E+00	1.08E+05	1.53E+05	0.00E+00	3.34E-01
C9IZ41;H0Y2Y8;Q15942	0.00E+00	0.00E+00	0.00E+00	0.00E+00	0.00E+00	0.00E+00	3.46E+06	0.00E+00	0.00E+00	0.00E+00	0.00E+00	0.00E+00	0.00E+00	0.00E+00	0.00E+00	0.00E+00	2.16E+05	4.32E+05	0.00E+00	8.64E+05	1.22E+06	0.00E+00	3.34E-01
C9JRU6;C9J0B2;C9JUW4;C9J8R4;C9JVE2;Q96GG9	0.00E+00	0.00E+00	0.00E+00	0.00E+00	0.00E+00	0.00E+00	9.47E+05	0.00E+00	0.00E+00	0.00E+00	0.00E+00	0.00E+00	0.00E+00	0.00E+00	0.00E+00	0.00E+00	5.92E+04	1.18E+05	0.00E+00	2.37E+05	3.35E+05	0.00E+00	3.34E-01
Q71UI9-5;Q71UI9-2;C9J0D1;Q99878;Q96KK5;Q71UI9;P0C0S5;Q9BTM1;Q16777;Q93077;Q8IUE6;Q7L7L0;Q6FI13;P20671;P0C0S8;P04908;Q96QV6;P16104;Q9BTM1-2	0.00E+00	0.00E+00	0.00E+00	0.00E+00	0.00E+00	0.00E+00	0.00E+00	0.00E+00	0.00E+00	0.00E+00	7.79E+06	0.00E+00	0.00E+00	0.00E+00	0.00E+00	0.00E+00	4.87E+05	0.00E+00	9.74E+05	1.95E+06	0.00E+00	2.75E+06	3.34E-01
C9J1E7;Q10567-4;Q10567-3;Q10567-2;Q10567	0.00E+00	0.00E+00	0.00E+00	0.00E+00	0.00E+00	0.00E+00	0.00E+00	0.00E+00	0.00E+00	0.00E+00	0.00E+00	0.00E+00	0.00E+00	0.00E+00	0.00E+00	9.20E+06	5.75E+05	0.00E+00	1.15E+06	2.30E+06	0.00E+00	3.25E+06	3.34E-01

C9JJV6;C9JZL8;C9J5M0;C9JC07;Q96S97	0.00E+00	0.00E+00	0.00E+00	0.00E+00	0.00E+00	0.00E+00	0.00E+00	0.00E+00	0.00E+00	0.00E+00	0.00E+00	0.00E+00	0.00E+00	0.00E+00	5.84E+05	0.00E+00	3.65E+04	0.00E+00	7.29E+04	1.46E+05	0.00E+00	2.06E+05	3.34E-01
C9J719;C9JJ78;Q15125	0.00E+00	0.00E+00	0.00E+00	0.00E+00	0.00E+00	0.00E+00	0.00E+00	0.00E+00	0.00E+00	0.00E+00	0.00E+00	0.00E+00	0.00E+00	0.00E+00	3.02E+05	0.00E+00	1.89E+04	0.00E+00	3.77E+04	7.54E+04	0.00E+00	1.07E+05	3.34E-01
E9PFH4;Q9Y5L0-5;Q9Y5L0-3;Q9Y5L0;Q9Y5L0-1;C9J7E5	0.00E+00	0.00E+00	0.00E+00	0.00E+00	0.00E+00	0.00E+00	0.00E+00	0.00E+00	0.00E+00	0.00E+00	0.00E+00	0.00E+00	0.00E+00	0.00E+00	0.00E+00	4.43E+05	2.77E+04	0.00E+00	5.54E+04	1.11E+05	0.00E+00	1.57E+05	3.34E-01
C9JQM9;C9J7S3;H7C278;C9JLC1;P14868-2;P14868	0.00E+00	0.00E+00	0.00E+00	0.00E+00	0.00E+00	0.00E+00	0.00E+00	0.00E+00	0.00E+00	3.00E+06	0.00E+00	0.00E+00	0.00E+00	0.00E+00	0.00E+00	0.00E+00	1.88E+05	0.00E+00	3.75E+05	7.51E+05	0.00E+00	1.06E+06	3.34E-01
C9J8Q1;F8W9P7;Q9UMS0-2;H7C537;Q9UMS0-3;Q9UMS0	0.00E+00	0.00E+00	0.00E+00	0.00E+00	0.00E+00	0.00E+00	0.00E+00	0.00E+00	0.00E+00	0.00E+00	0.00E+00	0.00E+00	0.00E+00	0.00E+00	0.00E+00	1.60E+06	9.98E+04	0.00E+00	2.00E+05	3.99E+05	0.00E+00	5.64E+05	3.34E-01
C9J9W2;Q14847-3;Q14847;Q14847-2	0.00E+00	0.00E+00	0.00E+00	0.00E+00	0.00E+00	0.00E+00	0.00E+00	0.00E+00	0.00E+00	0.00E+00	0.00E+00	3.63E+05	0.00E+00	0.00E+00	0.00E+00	0.00E+00	2.27E+04	0.00E+00	4.53E+04	9.06E+04	0.00E+00	1.28E+05	3.34E-01
C9JNK6;C9JAZ1;O75431-2;O75431	0.00E+00	0.00E+00	0.00E+00	0.00E+00	0.00E+00	0.00E+00	0.00E+00	0.00E+00	0.00E+00	0.00E+00	0.00E+00	0.00E+00	0.00E+00	5.75E+05	0.00E+00	0.00E+00	3.59E+04	0.00E+00	7.18E+04	1.44E+05	0.00E+00	2.03E+05	3.34E-01
H7C131;C9JDE9;P09110	0.00E+00	0.00E+00	0.00E+00	0.00E+00	0.00E+00	0.00E+00	0.00E+00	0.00E+00	1.54E+06	0.00E+00	0.00E+00	0.00E+00	0.00E+00	0.00E+00	0.00E+00	0.00E+00	9.60E+04	0.00E+00	1.92E+05	3.84E+05	0.00E+00	5.43E+05	3.34E-01
C9JG87;Q9NYK5;Q9NYK5-2	0.00E+00	0.00E+00	0.00E+00	0.00E+00	0.00E+00	0.00E+00	0.00E+00	0.00E+00	0.00E+00	0.00E+00	0.00E+00	0.00E+00	1.03E+06	0.00E+00	0.00E+00	0.00E+00	6.41E+04	0.00E+00	1.28E+05	2.56E+05	0.00E+00	3.62E+05	3.34E-01
C9JGT6;C9JM55;C9K055;F8W8W4;Q9UHG3-2;Q9UHG3	0.00E+00	0.00E+00	0.00E+00	0.00E+00	0.00E+00	0.00E+00	0.00E+00	0.00E+00	0.00E+00	0.00E+00	0.00E+00	0.00E+00	0.00E+00	0.00E+00	0.00E+00	5.06E+06	3.16E+05	0.00E+00	6.33E+05	1.27E+06	0.00E+00	1.79E+06	3.34E-01
C9JHW1;E7ESB6;Q6Y7W6-4;Q6Y7W6-5;Q6Y7W6;Q6Y7W6-3;I1E4Y6	0.00E+00	0.00E+00	0.00E+00	0.00E+00	0.00E+00	0.00E+00	0.00E+00	0.00E+00	0.00E+00	0.00E+00	0.00E+00	0.00E+00	0.00E+00	0.00E+00	1.55E+06	0.00E+00	9.69E+04	0.00E+00	1.94E+05	3.88E+05	0.00E+00	5.48E+05	3.34E-01
C9JIS1;C9JXA5;P62879;C9JZN1;E7EP32;Q9HAV0	0.00E+00	0.00E+00	0.00E+00	0.00E+00	0.00E+00	0.00E+00	0.00E+00	0.00E+00	4.18E+06	0.00E+00	0.00E+00	0.00E+00	0.00E+00	0.00E+00	0.00E+00	0.00E+00	2.62E+05	0.00E+00	5.23E+05	1.05E+06	0.00E+00	1.48E+06	3.34E-01
C9JTY3;C9JUE0;C9JJP5;Q92734-4;Q92734-3;Q05BK6;Q92734-2;Q92734	0.00E+00	0.00E+00	0.00E+00	0.00E+00	0.00E+00	0.00E+00	0.00E+00	0.00E+00	1.34E+05	0.00E+00	0.00E+00	0.00E+00	0.00E+00	0.00E+00	0.00E+00	0.00E+00	8.36E+03	0.00E+00	1.67E+04	3.34E+04	0.00E+00	4.73E+04	3.34E-01
C9JKI3;Q03135-2;E9PCT5;Q03135	0.00E+00	0.00E+00	0.00E+00	0.00E+00	0.00E+00	0.00E+00	0.00E+00	0.00E+00	0.00E+00	0.00E+00	0.00E+00	0.00E+00	0.00E+00	1.18E+06	0.00E+00	0.00E+00	7.39E+04	0.00E+00	1.48E+05	2.96E+05	0.00E+00	4.18E+05	3.34E-01
C9JLF4;Q9NVH0-2;Q9NVH0	0.00E+00	0.00E+00	0.00E+00	0.00E+00	0.00E+00	5.56E+06	0.00E+00	0.00E+00	0.00E+00	0.00E+00	0.00E+00	0.00E+00	0.00E+00	0.00E+00	0.00E+00	0.00E+00	3.47E+05	6.95E+05	0.00E+00	1.39E+06	1.96E+06	0.00E+00	3.34E-01
J3QKR0;C9JLV5;Q9UNS2;J3QL22;J3QS85;J3KTQ1;K7ES36;H7C3P9;Q9UNS2-2	0.00E+00	0.00E+00	0.00E+00	0.00E+00	0.00E+00	1.15E+06	0.00E+00	0.00E+00	0.00E+00	0.00E+00	0.00E+00	0.00E+00	0.00E+00	0.00E+00	0.00E+00	0.00E+00	7.21E+04	1.44E+05	0.00E+00	2.88E+05	4.08E+05	0.00E+00	3.34E-01
C9JXB8;C9JNW5;P83731	0.00E+00	0.00E+00	0.00E+00	0.00E+00	0.00E+00	0.00E+00	0.00E+00	1.14E+07	0.00E+00	0.00E+00	0.00E+00	0.00E+00	0.00E+00	0.00E+00	0.00E+00	0.00E+00	7.11E+05	1.42E+06	0.00E+00	2.85E+06	4.02E+06	0.00E+00	3.34E-01
C9JU35;Q9NPL8	0.00E+00	0.00E+00	0.00E+00	5.26E+06	0.00E+00	0.00E+00	0.00E+00	0.00E+00	0.00E+00	0.00E+00	0.00E+00	0.00E+00	0.00E+00	0.00E+00	0.00E+00	0.00E+00	3.29E+05	6.58E+05	0.00E+00	1.32E+06	1.86E+06	0.00E+00	3.34E-01
C9JY09;E7ET39;Q8NDZ4-2;Q8NDZ4	0.00E+00	0.00E+00	0.00E+00	0.00E+00	0.00E+00	0.00E+00	0.00E+00	0.00E+00	0.00E+00	0.00E+00	0.00E+00	2.77E+06	0.00E+00	0.00E+00	0.00E+00	0.00E+00	1.73E+05	0.00E+00	3.47E+05	6.93E+05	0.00E+00	9.80E+05	3.34E-01
C9JYS8;Q15233-2;Q15233;H7C367	0.00E+00	0.00E+00	0.00E+00	0.00E+00	0.00E+00	0.00E+00	1.02E+06	0.00E+00	0.00E+00	0.00E+00	0.00E+00	0.00E+00	0.00E+00	0.00E+00	0.00E+00	0.00E+00	6.36E+04	1.27E+05	0.00E+00	2.55E+05	3.60E+05	0.00E+00	3.34E-01
H7C2W1;C9K0G7;E9PCG9;Q02338	0.00E+00	0.00E+00	0.00E+00	0.00E+00	0.00E+00	0.00E+00	0.00E+00	0.00E+00	0.00E+00	0.00E+00	0.00E+00	2.32E+06	0.00E+00	0.00E+00	0.00E+00	0.00E+00	1.45E+05	0.00E+00	2.90E+05	5.79E+05	0.00E+00	8.19E+05	3.34E-01
P78344-2;H0Y3P2;P78344;D3DQV9	0.00E+00	0.00E+00	0.00E+00	0.00E+00	0.00E+00	0.00E+00	0.00E+00	0.00E+00	0.00E+00	0.00E+00	0.00E+00	0.00E+00	0.00E+00	0.00E+00	2.56E+06	0.00E+00	1.60E+05	0.00E+00	3.20E+05	6.40E+05	0.00E+00	9.05E+05	3.34E-01

H7C1W2;E9PF84;G5E9Q7;D3DWV9;E7EQB8;P51553-2;P51553	0.00E+00	0.00E+00	0.00E+00	0.00E+00	2.82E+06	0.00E+00	0.00E+00	0.00E+00	0.00E+00	0.00E+00	0.00E+00	0.00E+00	0.00E+00	0.00E+00	0.00E+00	0.00E+00	1.76E+05	3.52E+05	0.00E+00	7.04E+05	9.96E+05	0.00E+00	3.34E-01
Q9BWF3-3;E9PLB0;E9PM61;D6R9K7;U3KQD5;J3QRR5;Q9BWF3-4;Q9BWF3-2;E9PB51;Q9BQ04;Q9BWF3	0.00E+00	0.00E+00	0.00E+00	0.00E+00	0.00E+00	0.00E+00	0.00E+00	0.00E+00	0.00E+00	0.00E+00	0.00E+00	0.00E+00	0.00E+00	0.00E+00	0.00E+00	7.04E+05	4.40E+04	0.00E+00	8.80E+04	1.76E+05	0.00E+00	2.49E+05	3.34E-01
P46926-2;D6RAY7;D6R9P4;P46926;D6RFF8;D6R917;D6RB13	0.00E+00	0.00E+00	0.00E+00	0.00E+00	0.00E+00	0.00E+00	0.00E+00	0.00E+00	0.00E+00	0.00E+00	0.00E+00	0.00E+00	2.55E+05	0.00E+00	0.00E+00	0.00E+00	1.60E+04	0.00E+00	3.19E+04	6.38E+04	0.00E+00	9.03E+04	3.34E-01
D6RIZ4;D6RA47;Q14728;D6RE79	0.00E+00	0.00E+00	0.00E+00	0.00E+00	0.00E+00	0.00E+00	0.00E+00	0.00E+00	0.00E+00	0.00E+00	0.00E+00	0.00E+00	0.00E+00	1.10E+06	0.00E+00	0.00E+00	6.88E+04	0.00E+00	1.38E+05	2.75E+05	0.00E+00	3.89E+05	3.34E-01
Q9BT78-2;Q9BT78;D6RAX7;D6RFN0;D6RD63	0.00E+00	0.00E+00	0.00E+00	0.00E+00	0.00E+00	0.00E+00	0.00E+00	3.88E+06	0.00E+00	0.00E+00	0.00E+00	0.00E+00	0.00E+00	0.00E+00	0.00E+00	0.00E+00	2.43E+05	4.85E+05	0.00E+00	9.71E+05	1.37E+06	0.00E+00	3.34E-01
D6RDG0;Q14108-2;Q14108	0.00E+00	0.00E+00	0.00E+00	0.00E+00	0.00E+00	0.00E+00	0.00E+00	0.00E+00	0.00E+00	0.00E+00	0.00E+00	0.00E+00	0.00E+00	0.00E+00	0.00E+00	2.12E+06	1.33E+05	0.00E+00	2.66E+05	5.31E+05	0.00E+00	7.51E+05	3.34E-01
D6RGI3;Q9NVA2;D6RER5;D6RDP1;D6R9Y6;Q14141-3;D6RDU5;Q14141-2;Q14141-4;B1AMS2;Q14141;Q9NVA2-2	0.00E+00	0.00E+00	0.00E+00	4.21E+06	0.00E+00	0.00E+00	0.00E+00	0.00E+00	0.00E+00	0.00E+00	0.00E+00	0.00E+00	0.00E+00	0.00E+00	0.00E+00	0.00E+00	2.63E+05	5.26E+05	0.00E+00	1.05E+06	1.49E+06	0.00E+00	3.34E-01
H0YAF5;H0Y8W8;O94979-7;H7BXG7;O94979-6;O94979-3;O94979-10;O94979-4;O94979-9;O94979-2;O94979;O94979-8;D6REX3	0.00E+00	0.00E+00	0.00E+00	0.00E+00	0.00E+00	0.00E+00	0.00E+00	0.00E+00	0.00E+00	0.00E+00	0.00E+00	5.19E+06	0.00E+00	0.00E+00	0.00E+00	0.00E+00	3.24E+05	0.00E+00	6.49E+05	1.30E+06	0.00E+00	1.83E+06	3.34E-01
D6RH20;G5EA06;Q92552;Q92552-2;D6RJC7	3.30E+07	0.00E+00	0.00E+00	0.00E+00	0.00E+00	0.00E+00	0.00E+00	0.00E+00	0.00E+00	0.00E+00	0.00E+00	0.00E+00	0.00E+00	0.00E+00	0.00E+00	0.00E+00	2.06E+06	4.13E+06	0.00E+00	8.25E+06	1.17E+07	0.00E+00	3.34E-01
D6RHJ3	0.00E+00	0.00E+00	0.00E+00	0.00E+00	0.00E+00	2.85E+06	0.00E+00	0.00E+00	0.00E+00	0.00E+00	0.00E+00	0.00E+00	0.00E+00	0.00E+00	0.00E+00	0.00E+00	1.78E+05	3.57E+05	0.00E+00	7.13E+05	1.01E+06	0.00E+00	3.34E-01
O60313;E5KLK1;E5KLJ6;E5KLJ9;O60313-2;E5KLJ5	0.00E+00	0.00E+00	0.00E+00	0.00E+00	0.00E+00	0.00E+00	0.00E+00	0.00E+00	0.00E+00	0.00E+00	0.00E+00	0.00E+00	6.25E+05	0.00E+00	0.00E+00	0.00E+00	3.90E+04	0.00E+00	7.81E+04	1.56E+05	0.00E+00	2.21E+05	3.34E-01
E5RGA2;P60228;H0YAW4;E5RHS5	0.00E+00	0.00E+00	0.00E+00	0.00E+00	0.00E+00	0.00E+00	6.60E+06	0.00E+00	0.00E+00	0.00E+00	0.00E+00	0.00E+00	0.00E+00	0.00E+00	0.00E+00	0.00E+00	4.13E+05	8.25E+05	0.00E+00	1.65E+06	2.33E+06	0.00E+00	3.34E-01
E5RGJ2;Q15006	0.00E+00	0.00E+00	0.00E+00	0.00E+00	0.00E+00	0.00E+00	0.00E+00	0.00E+00	0.00E+00	0.00E+00	0.00E+00	0.00E+00	0.00E+00	0.00E+00	0.00E+00	2.27E+06	1.42E+05	0.00E+00	2.84E+05	5.69E+05	0.00E+00	8.04E+05	3.34E-01
E5RH50;Q6PKG0-3;Q6PKG0;H0YC33;H0YC73;H0YBW1	0.00E+00	0.00E+00	0.00E+00	0.00E+00	0.00E+00	0.00E+00	4.18E+06	0.00E+00	0.00E+00	0.00E+00	0.00E+00	0.00E+00	0.00E+00	0.00E+00	0.00E+00	0.00E+00	2.61E+05	5.23E+05	0.00E+00	1.05E+06	1.48E+06	0.00E+00	3.34E-01
E5RHF4;Q9P015;E5RIZ4	0.00E+00	0.00E+00	0.00E+00	0.00E+00	0.00E+00	1.04E+06	0.00E+00	0.00E+00	0.00E+00	0.00E+00	0.00E+00	0.00E+00	0.00E+00	0.00E+00	0.00E+00	0.00E+00	6.51E+04	1.30E+05	0.00E+00	2.61E+05	3.69E+05	0.00E+00	3.34E-01

E5RIK2;Q9UQ16-5;E5RHK8;Q9UQ16-2;Q9UQ16-3;P50570-3;P50570-2;Q9UQ16;P50570-5;P50570-4;P50570;Q9UQ16-4	0.00E+00	0.00E+00	0.00E+00	0.00E+00	0.00E+00	0.00E+00	0.00E+00	0.00E+00	0.00E+00	0.00E+00	0.00E+00	0.00E+00	0.00E+00	0.00E+00	0.00E+00	3.41E+06	2.13E+05	0.00E+00	4.26E+05	8.52E+05	0.00E+00	1.20E+06	3.34E-01
E5RHW4;O94905;E5RJ09	0.00E+00	0.00E+00	0.00E+00	0.00E+00	0.00E+00	0.00E+00	0.00E+00	9.46E+05	0.00E+00	0.00E+00	0.00E+00	0.00E+00	0.00E+00	0.00E+00	0.00E+00	0.00E+00	5.91E+04	1.18E+05	0.00E+00	2.36E+05	3.34E+05	0.00E+00	3.34E-01
E5RK64;O95292-2;O95292	0.00E+00	0.00E+00	0.00E+00	0.00E+00	0.00E+00	0.00E+00	0.00E+00	0.00E+00	0.00E+00	0.00E+00	0.00E+00	0.00E+00	5.18E+05	0.00E+00	0.00E+00	0.00E+00	3.24E+04	0.00E+00	6.48E+04	1.30E+05	0.00E+00	1.83E+05	3.34E-01
E7EM64;Q7L5N1	0.00E+00	0.00E+00	0.00E+00	0.00E+00	0.00E+00	0.00E+00	0.00E+00	0.00E+00	0.00E+00	7.58E+05	0.00E+00	0.00E+00	0.00E+00	0.00E+00	0.00E+00	0.00E+00	4.74E+04	0.00E+00	9.48E+04	1.90E+05	0.00E+00	2.68E+05	3.34E-01
H0YBB4;H0YAV7;E7EMW7;O95071-2;O95071	0.00E+00	0.00E+00	2.76E+06	0.00E+00	0.00E+00	0.00E+00	0.00E+00	0.00E+00	0.00E+00	0.00E+00	0.00E+00	0.00E+00	0.00E+00	0.00E+00	0.00E+00	0.00E+00	1.72E+05	3.44E+05	0.00E+00	6.89E+05	9.74E+05	0.00E+00	3.34E-01
K7EJL1;E7ENJ6;Q9BXS5;Q9BXS5-2;K7EPJ8;A0A087WZX7	0.00E+00	0.00E+00	0.00E+00	0.00E+00	0.00E+00	0.00E+00	0.00E+00	0.00E+00	0.00E+00	0.00E+00	0.00E+00	0.00E+00	0.00E+00	2.96E+06	0.00E+00	0.00E+00	1.85E+05	0.00E+00	3.71E+05	7.41E+05	0.00E+00	1.05E+06	3.34E-01
E7ENN3;Q8NF91-4;Q8NF91;A0A087WYJ5;F5GYQ7;F5GXQ8;Q8NF91-5;Q8NF91-6;Q8NF91-8;Q8NF91-2;Q8NF91-7	0.00E+00	0.00E+00	0.00E+00	0.00E+00	0.00E+00	0.00E+00	0.00E+00	0.00E+00	0.00E+00	0.00E+00	0.00E+00	0.00E+00	8.86E+05	0.00E+00	0.00E+00	0.00E+00	5.54E+04	0.00E+00	1.11E+05	2.21E+05	0.00E+00	3.13E+05	3.34E-01
G3V1Q4;E7ES33;Q16181-2;Q16181;E7EPK1;H0Y3Y4	0.00E+00	0.00E+00	0.00E+00	0.00E+00	7.03E+04	0.00E+00	0.00E+00	0.00E+00	0.00E+00	0.00E+00	0.00E+00	0.00E+00	0.00E+00	0.00E+00	0.00E+00	0.00E+00	4.39E+03	8.78E+03	0.00E+00	1.76E+04	2.48E+04	0.00E+00	3.34E-01
P19404;E7EPT4	0.00E+00	0.00E+00	0.00E+00	0.00E+00	0.00E+00	0.00E+00	3.80E+06	0.00E+00	0.00E+00	0.00E+00	0.00E+00	0.00E+00	0.00E+00	0.00E+00	0.00E+00	0.00E+00	2.38E+05	4.75E+05	0.00E+00	9.51E+05	1.34E+06	0.00E+00	3.34E-01
E7EWE1;E7EQ61;Q9GZZ9	9.75E+06	0.00E+00	0.00E+00	0.00E+00	0.00E+00	0.00E+00	0.00E+00	0.00E+00	0.00E+00	0.00E+00	0.00E+00	0.00E+00	0.00E+00	0.00E+00	0.00E+00	0.00E+00	6.10E+05	1.22E+06	0.00E+00	2.44E+06	3.45E+06	0.00E+00	3.34E-01
E7EQ72;Q15363;F5GX39	0.00E+00	0.00E+00	0.00E+00	0.00E+00	0.00E+00	4.46E+06	0.00E+00	0.00E+00	0.00E+00	0.00E+00	0.00E+00	0.00E+00	0.00E+00	0.00E+00	0.00E+00	0.00E+00	2.79E+05	5.58E+05	0.00E+00	1.12E+06	1.58E+06	0.00E+00	3.34E-01
P02788-2;E7EQB2;E7ER44;P02788	0.00E+00	0.00E+00	0.00E+00	0.00E+00	0.00E+00	0.00E+00	0.00E+00	3.07E+06	0.00E+00	0.00E+00	0.00E+00	0.00E+00	0.00E+00	0.00E+00	0.00E+00	0.00E+00	1.92E+05	3.84E+05	0.00E+00	7.68E+05	1.09E+06	0.00E+00	3.34E-01
E7EQG2;Q14240;Q14240-2	0.00E+00	3.93E+06	0.00E+00	0.00E+00	0.00E+00	0.00E+00	0.00E+00	0.00E+00	0.00E+00	0.00E+00	0.00E+00	0.00E+00	0.00E+00	0.00E+00	0.00E+00	0.00E+00	2.46E+05	4.92E+05	0.00E+00	9.83E+05	1.39E+06	0.00E+00	3.34E-01
E9PES6;E7ES08;E7EQU1;O15347	0.00E+00	0.00E+00	0.00E+00	0.00E+00	0.00E+00	0.00E+00	0.00E+00	0.00E+00	0.00E+00	0.00E+00	0.00E+00	0.00E+00	0.00E+00	5.00E+06	0.00E+00	0.00E+00	3.13E+05	0.00E+00	6.25E+05	1.25E+06	0.00E+00	1.77E+06	3.34E-01
E7ERW8;H9KV28;O60610-2;E9PHQ0;O60610-3;E9PEZ3;O60610;E7EMV0	0.00E+00	0.00E+00	0.00E+00	0.00E+00	0.00E+00	0.00E+00	0.00E+00	7.02E+05	0.00E+00	0.00E+00	0.00E+00	0.00E+00	0.00E+00	0.00E+00	0.00E+00	0.00E+00	4.39E+04	8.78E+04	0.00E+00	1.76E+05	2.48E+05	0.00E+00	3.34E-01
Q13428-2;Q13428-8;J3KQ96;Q13428-6;Q13428-7;Q13428;E7ETY2;Q13428-3;Q13428-4	0.00E+00	0.00E+00	0.00E+00	0.00E+00	0.00E+00	0.00E+00	0.00E+00	0.00E+00	0.00E+00	0.00E+00	0.00E+00	0.00E+00	1.48E+05	0.00E+00	0.00E+00	0.00E+00	9.22E+03	0.00E+00	1.84E+04	3.69E+04	0.00E+00	5.21E+04	3.34E-01
Q75MG1;E7ETZ4;Q9Y6E2;E9PFE3;C9JF98;F8WDX8;E9PFD4;Q9Y6E2-2;E7EMS9;B5MCE7;B5MCH7	0.00E+00	0.00E+00	0.00E+00	0.00E+00	2.94E+05	0.00E+00	0.00E+00	0.00E+00	0.00E+00	0.00E+00	0.00E+00	0.00E+00	0.00E+00	0.00E+00	0.00E+00	0.00E+00	1.84E+04	3.67E+04	0.00E+00	7.34E+04	1.04E+05	0.00E+00	3.34E-01
E7EV59;P21283	0.00E+00	0.00E+00	0.00E+00	0.00E+00	0.00E+00	0.00E+00	0.00E+00	0.00E+00	0.00E+00	0.00E+00	0.00E+00	0.00E+00	6.21E+05	0.00E+00	0.00E+00	0.00E+00	3.88E+04	0.00E+00	7.76E+04	1.55E+05	0.00E+00	2.19E+05	3.34E-01
E7EVJ5;Q96F07-2;Q96F07;A0A087WVE1;A0A087WTQ3;A0A087WWZ1	0.00E+00	0.00E+00	0.00E+00	0.00E+00	2.32E+06	0.00E+00	0.00E+00	0.00E+00	0.00E+00	0.00E+00	0.00E+00	0.00E+00	0.00E+00	0.00E+00	0.00E+00	0.00E+00	1.45E+05	2.90E+05	0.00E+00	5.80E+05	8.20E+05	0.00E+00	3.34E-01
E9PH64;E7EWZ0;Q9Y6M9;E9PF49	0.00E+00	0.00E+00	0.00E+00	0.00E+00	0.00E+00	0.00E+00	0.00E+00	0.00E+00	0.00E+00	0.00E+00	0.00E+00	6.78E+06	0.00E+00	0.00E+00	0.00E+00	0.00E+00	4.24E+05	0.00E+00	8.48E+05	1.70E+06	0.00E+00	2.40E+06	3.34E-01

Q14203-5;Q14203-2;Q14203-3;Q14203-4;E7EX90;Q14203-6;Q14203;Q6AWB1	0.00E+00	0.00E+00	0.00E+00	0.00E+00	0.00E+00	0.00E+00	0.00E+00	0.00E+00	0.00E+00	0.00E+00	0.00E+00	0.00E+00	0.00E+00	0.00E+00	2.20E+06	0.00E+00	1.38E+05	0.00E+00	2.76E+05	5.51E+05	0.00E+00	7.79E+05	3.34E-01
Q86V81;E9PB61	0.00E+00	0.00E+00	0.00E+00	0.00E+00	0.00E+00	0.00E+00	0.00E+00	0.00E+00	0.00E+00	2.19E+06	0.00E+00	0.00E+00	0.00E+00	0.00E+00	0.00E+00	0.00E+00	1.37E+05	0.00E+00	2.74E+05	5.47E+05	0.00E+00	7.74E+05	3.34E-01
E9PB90;P52789	0.00E+00	0.00E+00	0.00E+00	0.00E+00	0.00E+00	0.00E+00	0.00E+00	0.00E+00	2.95E+06	0.00E+00	0.00E+00	0.00E+00	0.00E+00	0.00E+00	0.00E+00	0.00E+00	1.84E+05	0.00E+00	3.68E+05	7.37E+05	0.00E+00	1.04E+06	3.34E-01
E9PG39;E9PC15;Q53H12	0.00E+00	0.00E+00	0.00E+00	0.00E+00	0.00E+00	0.00E+00	0.00E+00	0.00E+00	0.00E+00	0.00E+00	0.00E+00	0.00E+00	0.00E+00	0.00E+00	0.00E+00	1.91E+05	1.19E+04	0.00E+00	2.38E+04	4.76E+04	0.00E+00	6.74E+04	3.34E-01
E9PCB6;Q9BYT8;H0YAF7;H0YAK4	0.00E+00	0.00E+00	0.00E+00	0.00E+00	0.00E+00	0.00E+00	0.00E+00	0.00E+00	0.00E+00	7.51E+06	0.00E+00	0.00E+00	0.00E+00	0.00E+00	0.00E+00	0.00E+00	4.69E+05	0.00E+00	9.39E+05	1.88E+06	0.00E+00	2.66E+06	3.34E-01
H7C1Z2;E9PCP1;F8WD49;P48751-2;J3KNG9;P04920-2;P48751;P04920-3;P04920;P48751-3	0.00E+00	0.00E+00	0.00E+00	0.00E+00	7.69E+06	0.00E+00	0.00E+00	0.00E+00	0.00E+00	0.00E+00	0.00E+00	0.00E+00	0.00E+00	0.00E+00	0.00E+00	0.00E+00	4.80E+05	9.61E+05	0.00E+00	1.92E+06	2.72E+06	0.00E+00	3.34E-01
E9PCY7;P31943;G8JLB6;E5RGV0;D6RIU0;D6RBM0;E5RGH4;E7EQJ0;D6RAM1;D6R9T0;D6RFM3;D6RIT2;E7EN40;D6RDU3;D6RJ04;D6RIH9;H0YB39	0.00E+00	0.00E+00	0.00E+00	0.00E+00	0.00E+00	0.00E+00	0.00E+00	0.00E+00	0.00E+00	0.00E+00	6.08E+06	0.00E+00	0.00E+00	0.00E+00	0.00E+00	0.00E+00	3.80E+05	0.00E+00	7.60E+05	1.52E+06	0.00E+00	2.15E+06	3.34E-01
Q9NTJ3-2;E9PD53;Q9NTJ3	0.00E+00	0.00E+00	0.00E+00	0.00E+00	0.00E+00	0.00E+00	0.00E+00	0.00E+00	0.00E+00	0.00E+00	0.00E+00	0.00E+00	0.00E+00	4.40E+06	0.00E+00	0.00E+00	2.75E+05	0.00E+00	5.51E+05	1.10E+06	0.00E+00	1.56E+06	3.34E-01
Q96AE4;Q96AE4-2;E9PEB5	0.00E+00	0.00E+00	0.00E+00	0.00E+00	0.00E+00	0.00E+00	0.00E+00	2.20E+06	0.00E+00	0.00E+00	0.00E+00	0.00E+00	0.00E+00	0.00E+00	0.00E+00	0.00E+00	1.38E+05	2.75E+05	0.00E+00	5.51E+05	7.79E+05	0.00E+00	3.34E-01
H0YDD4;E9PEJ4;P10515	0.00E+00	0.00E+00	0.00E+00	0.00E+00	0.00E+00	0.00E+00	0.00E+00	0.00E+00	0.00E+00	1.16E+06	0.00E+00	0.00E+00	0.00E+00	0.00E+00	0.00E+00	0.00E+00	7.23E+04	0.00E+00	1.45E+05	2.89E+05	0.00E+00	4.09E+05	3.34E-01
Q96CM8-4;E9PF16;Q96CM8-3;Q96CM8;Q96CM8-2	0.00E+00	0.00E+00	0.00E+00	0.00E+00	0.00E+00	0.00E+00	0.00E+00	0.00E+00	2.74E+06	0.00E+00	0.00E+00	0.00E+00	0.00E+00	0.00E+00	0.00E+00	0.00E+00	1.72E+05	0.00E+00	3.43E+05	6.86E+05	0.00E+00	9.70E+05	3.34E-01
E9PF19;Q9Y4P3	0.00E+00	0.00E+00	0.00E+00	0.00E+00	0.00E+00	0.00E+00	0.00E+00	0.00E+00	0.00E+00	0.00E+00	0.00E+00	0.00E+00	2.25E+06	0.00E+00	0.00E+00	0.00E+00	1.40E+05	0.00E+00	2.81E+05	5.62E+05	0.00E+00	7.95E+05	3.34E-01
Q504U8;E9PFD7;P00533;C9JYS6;P00533-2;P00533-4;P00533-3	0.00E+00	0.00E+00	0.00E+00	0.00E+00	0.00E+00	0.00E+00	0.00E+00	0.00E+00	0.00E+00	0.00E+00	0.00E+00	0.00E+00	0.00E+00	2.07E+06	0.00E+00	0.00E+00	1.29E+05	0.00E+00	2.59E+05	5.18E+05	0.00E+00	7.32E+05	3.34E-01
Q9Y2Q3-4;E9PFN5;Q9Y2Q3;C9JNT3;Q9Y2Q3-3;Q9Y2Q3-2	0.00E+00	0.00E+00	0.00E+00	0.00E+00	0.00E+00	0.00E+00	0.00E+00	0.00E+00	0.00E+00	0.00E+00	0.00E+00	0.00E+00	0.00E+00	0.00E+00	2.39E+06	0.00E+00	1.50E+05	0.00E+00	2.99E+05	5.98E+05	0.00E+00	8.46E+05	3.34E-01
F5GYT8;E9PG35;E9PHF7;Q96RQ3	0.00E+00	0.00E+00	0.00E+00	0.00E+00	0.00E+00	0.00E+00	0.00E+00	0.00E+00	0.00E+00	0.00E+00	0.00E+00	0.00E+00	3.25E+06	0.00E+00	0.00E+00	0.00E+00	2.03E+05	0.00E+00	4.07E+05	8.14E+05	0.00E+00	1.15E+06	3.34E-01
P78559;P78559-2;E9PGC8;P46821	0.00E+00	0.00E+00	0.00E+00	0.00E+00	0.00E+00	0.00E+00	0.00E+00	0.00E+00	0.00E+00	1.46E+06	0.00E+00	0.00E+00	0.00E+00	0.00E+00	0.00E+00	0.00E+00	9.12E+04	0.00E+00	1.82E+05	3.65E+05	0.00E+00	5.16E+05	3.34E-01
I6L9B1;O43491-2;E9PII3;O43491-3;E9PK52;O43491-4;E9PHY5;O43491	0.00E+00	0.00E+00	0.00E+00	0.00E+00	0.00E+00	0.00E+00	0.00E+00	0.00E+00	0.00E+00	0.00E+00	0.00E+00	0.00E+00	2.13E+06	0.00E+00	0.00E+00	0.00E+00	1.33E+05	0.00E+00	2.66E+05	5.33E+05	0.00E+00	7.53E+05	3.34E-01
E9PI70;E9PIP0;E9PRL3;E9PPH0;E9PPA0;Q9BWS9-3;Q9BWS9;Q9BWS9-2	0.00E+00	0.00E+00	0.00E+00	0.00E+00	0.00E+00	0.00E+00	3.49E+06	0.00E+00	0.00E+00	0.00E+00	0.00E+00	0.00E+00	0.00E+00	0.00E+00	0.00E+00	0.00E+00	2.18E+05	4.37E+05	0.00E+00	8.74E+05	1.24E+06	0.00E+00	3.34E-01
K4DIA8;K4DIA2;K4DIB0;K4DIB2;E9PQ36;K4DIB8;E9PI74;K4DIB3;E9PS95;E9PJH7;Q9H1K4;Q9H936	0.00E+00	0.00E+00	0.00E+00	0.00E+00	0.00E+00	0.00E+00	0.00E+00	0.00E+00	0.00E+00	0.00E+00	0.00E+00	0.00E+00	3.35E+04	0.00E+00	0.00E+00	0.00E+00	2.10E+03	0.00E+00	4.19E+03	8.38E+03	0.00E+00	1.19E+04	3.34E-01

E9PIZ4;E9PSD5;E9PPQ5;Q9UHD1;Q9UHD1-2	0.00E+00	0.00E+00	0.00E+00	0.00E+00	0.00E+00	0.00E+00	0.00E+00	0.00E+00	0.00E+00	0.00E+00	0.00E+00	0.00E+00	0.00E+00	0.00E+00	0.00E+00	5.28E+04	3.30E+03	0.00E+00	6.60E+03	1.32E+04	0.00E+00	1.87E+04	3.34E-01
E9PK09;E9PQN2;E9PKI6;Q9NYF8-3;E9PK91;Q9NYF8-2;Q9NYF8	0.00E+00	0.00E+00	0.00E+00	0.00E+00	0.00E+00	0.00E+00	0.00E+00	0.00E+00	0.00E+00	0.00E+00	0.00E+00	0.00E+00	9.42E+05	0.00E+00	0.00E+00	0.00E+00	5.89E+04	0.00E+00	1.18E+05	2.35E+05	0.00E+00	3.33E+05	3.34E-01
E9PP36;E9PKZ0;E9PKU4;P62917	0.00E+00	0.00E+00	0.00E+00	0.00E+00	0.00E+00	0.00E+00	2.43E+06	0.00E+00	0.00E+00	0.00E+00	0.00E+00	0.00E+00	0.00E+00	0.00E+00	0.00E+00	0.00E+00	1.52E+05	3.04E+05	0.00E+00	6.08E+05	8.60E+05	0.00E+00	3.34E-01
E9PLA9;Q14444-2;Q14444;E9PP31;G3V153;A0A087X082	0.00E+00	0.00E+00	0.00E+00	0.00E+00	0.00E+00	9.07E+05	0.00E+00	0.00E+00	0.00E+00	0.00E+00	0.00E+00	0.00E+00	0.00E+00	0.00E+00	0.00E+00	0.00E+00	5.67E+04	1.13E+05	0.00E+00	2.27E+05	3.21E+05	0.00E+00	3.34E-01
E9PLT0;O75534-2;O75534;O75534-3;O75534-4	0.00E+00	0.00E+00	0.00E+00	0.00E+00	6.88E+05	0.00E+00	0.00E+00	0.00E+00	0.00E+00	0.00E+00	0.00E+00	0.00E+00	0.00E+00	0.00E+00	0.00E+00	0.00E+00	4.30E+04	8.60E+04	0.00E+00	1.72E+05	2.43E+05	0.00E+00	3.34E-01
E9PQP1;E9PMX3;P49821-2;G3V0I5;P49821;E9PPS5;E9PJL9;H0YE81;E9PLC6;E9PPR0;B4DE93	0.00E+00	0.00E+00	0.00E+00	0.00E+00	0.00E+00	0.00E+00	0.00E+00	0.00E+00	0.00E+00	0.00E+00	0.00E+00	6.03E+05	0.00E+00	0.00E+00	0.00E+00	0.00E+00	3.77E+04	0.00E+00	7.54E+04	1.51E+05	0.00E+00	2.13E+05	3.34E-01
F2Z2E2;Q86VI3	0.00E+00	0.00E+00	0.00E+00	0.00E+00	0.00E+00	0.00E+00	0.00E+00	0.00E+00	0.00E+00	0.00E+00	0.00E+00	0.00E+00	0.00E+00	0.00E+00	0.00E+00	2.57E+05	1.61E+04	0.00E+00	3.22E+04	6.43E+04	0.00E+00	9.10E+04	3.34E-01
F2Z2K0;Q9UNZ2;Q9UNZ2-5;R4GNE6;Q9UNZ2-6;Q9UNZ2-4	0.00E+00	0.00E+00	0.00E+00	0.00E+00	0.00E+00	0.00E+00	0.00E+00	0.00E+00	0.00E+00	1.57E+06	0.00E+00	0.00E+00	0.00E+00	0.00E+00	0.00E+00	0.00E+00	9.84E+04	0.00E+00	1.97E+05	3.94E+05	0.00E+00	5.57E+05	3.34E-01
O00764-3;F2Z2Y4;O00764;O00764-2	0.00E+00	0.00E+00	0.00E+00	0.00E+00	0.00E+00	0.00E+00	2.46E+05	0.00E+00	0.00E+00	0.00E+00	0.00E+00	0.00E+00	0.00E+00	0.00E+00	0.00E+00	0.00E+00	1.54E+04	3.07E+04	0.00E+00	6.15E+04	8.69E+04	0.00E+00	3.34E-01
F2Z3J2;Q16401-2;Q16401	0.00E+00	0.00E+00	0.00E+00	0.00E+00	0.00E+00	0.00E+00	0.00E+00	0.00E+00	0.00E+00	0.00E+00	0.00E+00	0.00E+00	0.00E+00	0.00E+00	0.00E+00	1.96E+05	1.22E+04	0.00E+00	2.45E+04	4.89E+04	0.00E+00	6.92E+04	3.34E-01
F5GWX2;Q9NRV9;H0YG71	0.00E+00	0.00E+00	0.00E+00	0.00E+00	0.00E+00	0.00E+00	0.00E+00	0.00E+00	0.00E+00	0.00E+00	0.00E+00	0.00E+00	0.00E+00	0.00E+00	3.01E+06	0.00E+00	1.88E+05	0.00E+00	3.76E+05	7.53E+05	0.00E+00	1.06E+06	3.34E-01
F5GYN4;Q96FW1;J3KR44;F5H6Q1;F5GYJ8;F5H3F0;Q96FW1-2	0.00E+00	0.00E+00	0.00E+00	0.00E+00	0.00E+00	1.89E+06	0.00E+00	0.00E+00	0.00E+00	0.00E+00	0.00E+00	0.00E+00	0.00E+00	0.00E+00	0.00E+00	0.00E+00	1.18E+05	2.37E+05	0.00E+00	4.74E+05	6.70E+05	0.00E+00	3.34E-01
J3QL14;R4GN72;P61421;F5GYQ1	0.00E+00	0.00E+00	0.00E+00	2.22E+06	0.00E+00	0.00E+00	0.00E+00	0.00E+00	0.00E+00	0.00E+00	0.00E+00	0.00E+00	0.00E+00	0.00E+00	0.00E+00	0.00E+00	1.39E+05	2.77E+05	0.00E+00	5.55E+05	7.84E+05	0.00E+00	3.34E-01
G3V5X8;G3V4Q2;G3V4V1;G3V1W4;G3V3G5;G3V2R6;F5H365;Q15436	0.00E+00	0.00E+00	0.00E+00	0.00E+00	0.00E+00	0.00E+00	0.00E+00	0.00E+00	0.00E+00	0.00E+00	0.00E+00	0.00E+00	0.00E+00	0.00E+00	0.00E+00	8.09E+05	5.06E+04	0.00E+00	1.01E+05	2.02E+05	0.00E+00	2.86E+05	3.34E-01
F5H4C6;H7C3P4;P15586-2;P15586;F6S8M0;H0YFA9	0.00E+00	0.00E+00	0.00E+00	0.00E+00	0.00E+00	0.00E+00	0.00E+00	0.00E+00	1.30E+06	0.00E+00	0.00E+00	0.00E+00	0.00E+00	0.00E+00	0.00E+00	0.00E+00	8.11E+04	0.00E+00	1.62E+05	3.24E+05	0.00E+00	4.59E+05	3.34E-01
Q12849-5;H0Y8R1;F5H5I6;H0YAK1;Q12849	0.00E+00	0.00E+00	0.00E+00	0.00E+00	0.00E+00	0.00E+00	0.00E+00	0.00E+00	0.00E+00	0.00E+00	0.00E+00	0.00E+00	1.63E+06	0.00E+00	0.00E+00	0.00E+00	1.02E+05	0.00E+00	2.04E+05	4.07E+05	0.00E+00	5.76E+05	3.34E-01
O00159-2;F5H6E2;O00159-3;O00159;I3L4D4;I3L501;I3L3Y6;I3L204	0.00E+00	0.00E+00	0.00E+00	0.00E+00	0.00E+00	0.00E+00	0.00E+00	0.00E+00	0.00E+00	0.00E+00	0.00E+00	0.00E+00	0.00E+00	0.00E+00	0.00E+00	1.73E+06	1.08E+05	0.00E+00	2.16E+05	4.32E+05	0.00E+00	6.11E+05	3.34E-01
G3V1U5;F5H6U7;Q9Y3E0	0.00E+00	0.00E+00	1.12E+06	0.00E+00	0.00E+00	0.00E+00	0.00E+00	0.00E+00	0.00E+00	0.00E+00	0.00E+00	0.00E+00	0.00E+00	0.00E+00	0.00E+00	0.00E+00	7.01E+04	1.40E+05	0.00E+00	2.80E+05	3.97E+05	0.00E+00	3.34E-01
F6S6P2;X6REW1;P46379-4;P46379-5;P46379-2;P46379;P46379-3	0.00E+00	0.00E+00	0.00E+00	0.00E+00	0.00E+00	0.00E+00	4.31E+06	0.00E+00	0.00E+00	0.00E+00	0.00E+00	0.00E+00	0.00E+00	0.00E+00	0.00E+00	0.00E+00	2.69E+05	5.38E+05	0.00E+00	1.08E+06	1.52E+06	0.00E+00	3.34E-01
F8VX13;F8VNX2;F8VZQ1;P08237-2;P08237;P08237-3	0.00E+00	0.00E+00	0.00E+00	0.00E+00	0.00E+00	2.37E+06	0.00E+00	0.00E+00	0.00E+00	0.00E+00	0.00E+00	0.00E+00	0.00E+00	0.00E+00	0.00E+00	0.00E+00	1.48E+05	2.96E+05	0.00E+00	5.93E+05	8.38E+05	0.00E+00	3.34E-01

F8VPD4;P27708	0.00E+00	0.00E+00	0.00E+00	0.00E+00	0.00E+00	0.00E+00	0.00E+00	0.00E+00	0.00E+00	3.56E+06	0.00E+00	0.00E+00	0.00E+00	0.00E+00	0.00E+00	0.00E+00	2.23E+05	0.00E+00	4.45E+05	8.91E+05	0.00E+00	1.26E+06	3.34E-01
H0YHL1;F8VX93;F8VRV7;F8VW18;F8W1I6;Q13561;Q13561-3;Q13561-2	0.00E+00	0.00E+00	0.00E+00	0.00E+00	0.00E+00	0.00E+00	0.00E+00	0.00E+00	2.39E+06	0.00E+00	0.00E+00	0.00E+00	0.00E+00	0.00E+00	0.00E+00	0.00E+00	1.49E+05	0.00E+00	2.98E+05	5.96E+05	0.00E+00	8.43E+05	3.34E-01
F8VV32;P61626	0.00E+00	0.00E+00	0.00E+00	0.00E+00	0.00E+00	3.51E+06	0.00E+00	0.00E+00	0.00E+00	0.00E+00	0.00E+00	0.00E+00	0.00E+00	0.00E+00	0.00E+00	0.00E+00	2.19E+05	4.39E+05	0.00E+00	8.77E+05	1.24E+06	0.00E+00	3.34E-01
P82979;F8VZQ9;H0YHG0	0.00E+00	0.00E+00	0.00E+00	0.00E+00	0.00E+00	0.00E+00	0.00E+00	0.00E+00	0.00E+00	0.00E+00	0.00E+00	0.00E+00	0.00E+00	0.00E+00	1.65E+06	0.00E+00	1.03E+05	0.00E+00	2.07E+05	4.13E+05	0.00E+00	5.85E+05	3.34E-01
F8W950;Q13155;A8MU58	0.00E+00	0.00E+00	0.00E+00	0.00E+00	0.00E+00	0.00E+00	0.00E+00	0.00E+00	0.00E+00	0.00E+00	0.00E+00	0.00E+00	0.00E+00	3.80E+05	0.00E+00	0.00E+00	2.38E+04	0.00E+00	4.75E+04	9.50E+04	0.00E+00	1.34E+05	3.34E-01
Q13277-2;F8W9Y0;Q13277-3;Q13277	0.00E+00	0.00E+00	0.00E+00	0.00E+00	0.00E+00	0.00E+00	0.00E+00	0.00E+00	0.00E+00	0.00E+00	0.00E+00	0.00E+00	0.00E+00	2.16E+06	0.00E+00	0.00E+00	1.35E+05	0.00E+00	2.69E+05	5.39E+05	0.00E+00	7.62E+05	3.34E-01
Q16630-3;F8WJN3;Q16630;Q16630-2	0.00E+00	0.00E+00	0.00E+00	0.00E+00	0.00E+00	1.49E+06	0.00E+00	0.00E+00	0.00E+00	0.00E+00	0.00E+00	0.00E+00	0.00E+00	0.00E+00	0.00E+00	0.00E+00	9.32E+04	1.86E+05	0.00E+00	3.73E+05	5.27E+05	0.00E+00	3.34E-01
O75439;G3V0E4	0.00E+00	0.00E+00	0.00E+00	0.00E+00	0.00E+00	0.00E+00	0.00E+00	0.00E+00	3.94E+06	0.00E+00	0.00E+00	0.00E+00	0.00E+00	0.00E+00	0.00E+00	0.00E+00	2.46E+05	0.00E+00	4.92E+05	9.85E+05	0.00E+00	1.39E+06	3.34E-01
G3V126;Q9UI12-2;Q9UI12	0.00E+00	0.00E+00	0.00E+00	0.00E+00	0.00E+00	0.00E+00	0.00E+00	0.00E+00	8.81E+05	0.00E+00	0.00E+00	0.00E+00	0.00E+00	0.00E+00	0.00E+00	0.00E+00	5.51E+04	0.00E+00	1.10E+05	2.20E+05	0.00E+00	3.12E+05	3.34E-01
Q9BZZ5-3;Q9BZZ5-2;G3V1C3;Q9BZZ5-6;Q9BZZ5;Q9BZZ5-1;Q9BZZ5-5	0.00E+00	0.00E+00	0.00E+00	0.00E+00	0.00E+00	0.00E+00	0.00E+00	0.00E+00	0.00E+00	0.00E+00	0.00E+00	4.33E+05	0.00E+00	0.00E+00	0.00E+00	0.00E+00	2.71E+04	0.00E+00	5.42E+04	1.08E+05	0.00E+00	1.53E+05	3.34E-01
G3V325;P56134-4;C9JJT5;P56134-3;P56134-2;P56134;C9JU26;O75127	0.00E+00	0.00E+00	0.00E+00	0.00E+00	0.00E+00	0.00E+00	0.00E+00	0.00E+00	0.00E+00	0.00E+00	0.00E+00	0.00E+00	0.00E+00	0.00E+00	4.13E+05	0.00E+00	2.58E+04	0.00E+00	5.16E+04	1.03E+05	0.00E+00	1.46E+05	3.34E-01
G3V3B3;Q9UBV2-2;Q9UBV2	0.00E+00	0.00E+00	0.00E+00	0.00E+00	0.00E+00	0.00E+00	0.00E+00	0.00E+00	0.00E+00	0.00E+00	0.00E+00	0.00E+00	0.00E+00	0.00E+00	0.00E+00	2.43E+06	1.52E+05	0.00E+00	3.03E+05	6.07E+05	0.00E+00	8.58E+05	3.34E-01
G3V529;Q9GZR7	0.00E+00	1.20E+06	0.00E+00	0.00E+00	0.00E+00	0.00E+00	0.00E+00	0.00E+00	0.00E+00	0.00E+00	0.00E+00	0.00E+00	0.00E+00	0.00E+00	0.00E+00	0.00E+00	7.51E+04	1.50E+05	0.00E+00	3.01E+05	4.25E+05	0.00E+00	3.34E-01
M0QXI4;Q5HY98;G3XAE0	0.00E+00	0.00E+00	0.00E+00	2.88E+06	0.00E+00	0.00E+00	0.00E+00	0.00E+00	0.00E+00	0.00E+00	0.00E+00	0.00E+00	0.00E+00	0.00E+00	0.00E+00	0.00E+00	1.80E+05	3.60E+05	0.00E+00	7.20E+05	1.02E+06	0.00E+00	3.34E-01
P35221-3;G3XAM7;P35221;P35221-2	0.00E+00	0.00E+00	0.00E+00	0.00E+00	0.00E+00	0.00E+00	0.00E+00	0.00E+00	0.00E+00	0.00E+00	0.00E+00	0.00E+00	0.00E+00	0.00E+00	0.00E+00	5.41E+06	3.38E+05	0.00E+00	6.76E+05	1.35E+06	0.00E+00	1.91E+06	3.34E-01
Q9BQ52-2;Q9BQ52-4;G5E9D5;Q9BQ52;J3QRS2;V9GYU5;J3QL08;J3QLK4;V9GZ72;V9GYS7;H7C2I4;E7ES68	0.00E+00	0.00E+00	1.75E+06	0.00E+00	0.00E+00	0.00E+00	0.00E+00	0.00E+00	0.00E+00	0.00E+00	0.00E+00	0.00E+00	0.00E+00	0.00E+00	0.00E+00	0.00E+00	1.09E+05	2.19E+05	0.00E+00	4.38E+05	6.19E+05	0.00E+00	3.34E-01
G5E9W7;G5E9V5;P82650;H7C5L9	0.00E+00	3.94E+05	0.00E+00	0.00E+00	0.00E+00	0.00E+00	0.00E+00	0.00E+00	0.00E+00	0.00E+00	0.00E+00	0.00E+00	0.00E+00	0.00E+00	0.00E+00	0.00E+00	2.46E+04	4.93E+04	0.00E+00	9.85E+04	1.39E+05	0.00E+00	3.34E-01
O00429-4;O00429-5;O00429-3;G8JLD5;O00429-2;O00429;O00429-8;O00429-6;O00429-7	0.00E+00	0.00E+00	0.00E+00	0.00E+00	1.21E+07	0.00E+00	0.00E+00	0.00E+00	0.00E+00	0.00E+00	0.00E+00	0.00E+00	0.00E+00	0.00E+00	0.00E+00	0.00E+00	7.58E+05	1.52E+06	0.00E+00	3.03E+06	4.29E+06	0.00E+00	3.34E-01
H0Y390;Q9UPN3-4;Q9UPN3-3;Q9UPN3-2;Q9UPN3;H3BPE1;H3BQK9;H0Y314;Q9UPN3-5	0.00E+00	0.00E+00	0.00E+00	0.00E+00	0.00E+00	0.00E+00	2.78E+05	0.00E+00	0.00E+00	0.00E+00	0.00E+00	0.00E+00	0.00E+00	0.00E+00	0.00E+00	0.00E+00	1.74E+04	3.48E+04	0.00E+00	6.96E+04	9.84E+04	0.00E+00	3.34E-01
H0Y4R1;P12268	0.00E+00	0.00E+00	0.00E+00	0.00E+00	0.00E+00	0.00E+00	0.00E+00	0.00E+00	0.00E+00	0.00E+00	0.00E+00	0.00E+00	0.00E+00	7.81E+06	0.00E+00	0.00E+00	4.88E+05	0.00E+00	9.76E+05	1.95E+06	0.00E+00	2.76E+06	3.34E-01
Q5T446;H0Y5R6;P06132	0.00E+00	0.00E+00	0.00E+00	0.00E+00	0.00E+00	0.00E+00	0.00E+00	4.56E+05	0.00E+00	0.00E+00	0.00E+00	0.00E+00	0.00E+00	0.00E+00	0.00E+00	0.00E+00	2.85E+04	5.70E+04	0.00E+00	1.14E+05	1.61E+05	0.00E+00	3.34E-01
H3BR27;P38159-3;H0Y6E7;H3BT71;Q96E39;P38159;H3BNC1;P38159-2	0.00E+00	0.00E+00	0.00E+00	0.00E+00	0.00E+00	0.00E+00	0.00E+00	0.00E+00	0.00E+00	0.00E+00	0.00E+00	0.00E+00	0.00E+00	0.00E+00	0.00E+00	6.29E+05	3.93E+04	0.00E+00	7.86E+04	1.57E+05	0.00E+00	2.22E+05	3.34E-01

H0Y750;O75787-2;O75787;H7C240;H7C3E1	0.00E+00	0.00E+00	0.00E+00	0.00E+00	0.00E+00	0.00E+00	0.00E+00	0.00E+00	0.00E+00	0.00E+00	0.00E+00	0.00E+00	0.00E+00	0.00E+00	7.86E+05	0.00E+00	4.91E+04	0.00E+00	9.82E+04	1.96E+05	0.00E+00	2.78E+05	3.34E-01
H0YC27;H0YC67;H0YBD9;Q96DB5-2;Q96DB5	0.00E+00	0.00E+00	0.00E+00	0.00E+00	0.00E+00	0.00E+00	1.72E+06	0.00E+00	0.00E+00	0.00E+00	0.00E+00	0.00E+00	0.00E+00	0.00E+00	0.00E+00	0.00E+00	1.07E+05	2.14E+05	0.00E+00	4.29E+05	6.07E+05	0.00E+00	3.34E-01
H0YC04;P21281	0.00E+00	0.00E+00	0.00E+00	0.00E+00	0.00E+00	0.00E+00	0.00E+00	9.42E+05	0.00E+00	0.00E+00	0.00E+00	0.00E+00	0.00E+00	0.00E+00	0.00E+00	0.00E+00	5.89E+04	1.18E+05	0.00E+00	2.36E+05	3.33E+05	0.00E+00	3.34E-01
H0YFA4;P52943;P52943-2;H0YHD8	0.00E+00	0.00E+00	0.00E+00	0.00E+00	0.00E+00	0.00E+00	0.00E+00	0.00E+00	0.00E+00	0.00E+00	0.00E+00	0.00E+00	0.00E+00	0.00E+00	0.00E+00	9.45E+05	5.91E+04	0.00E+00	1.18E+05	2.36E+05	0.00E+00	3.34E+05	3.34E-01
H0YGJ7;H0YLP3;O75822-2;O75822-3;O75822	0.00E+00	0.00E+00	0.00E+00	0.00E+00	0.00E+00	0.00E+00	0.00E+00	0.00E+00	0.00E+00	6.32E+05	0.00E+00	0.00E+00	0.00E+00	0.00E+00	0.00E+00	0.00E+00	3.95E+04	0.00E+00	7.90E+04	1.58E+05	0.00E+00	2.23E+05	3.34E-01
H7C0I5;H0YGN5;Q9Y697-2;Q9Y697-3;Q9Y697	0.00E+00	0.00E+00	0.00E+00	0.00E+00	0.00E+00	0.00E+00	0.00E+00	0.00E+00	0.00E+00	0.00E+00	0.00E+00	0.00E+00	0.00E+00	0.00E+00	0.00E+00	1.46E+06	9.10E+04	0.00E+00	1.82E+05	3.64E+05	0.00E+00	5.15E+05	3.34E-01
H0YHS6;Q9Y2Z4	0.00E+00	0.00E+00	0.00E+00	0.00E+00	0.00E+00	0.00E+00	0.00E+00	0.00E+00	0.00E+00	0.00E+00	0.00E+00	0.00E+00	1.70E+06	0.00E+00	0.00E+00	0.00E+00	1.06E+05	0.00E+00	2.12E+05	4.24E+05	0.00E+00	6.00E+05	3.34E-01
H0YIB4;S4R3G0;Q13242	0.00E+00	0.00E+00	0.00E+00	0.00E+00	0.00E+00	0.00E+00	0.00E+00	1.17E+06	0.00E+00	0.00E+00	0.00E+00	0.00E+00	0.00E+00	0.00E+00	0.00E+00	0.00E+00	7.31E+04	1.46E+05	0.00E+00	2.92E+05	4.14E+05	0.00E+00	3.34E-01
H0YN26;P39687	0.00E+00	0.00E+00	0.00E+00	0.00E+00	0.00E+00	0.00E+00	0.00E+00	0.00E+00	0.00E+00	0.00E+00	0.00E+00	0.00E+00	0.00E+00	0.00E+00	0.00E+00	1.07E+06	6.68E+04	0.00E+00	1.34E+05	2.67E+05	0.00E+00	3.78E+05	3.34E-01
H3BLU7;O43488	0.00E+00	0.00E+00	0.00E+00	0.00E+00	0.00E+00	0.00E+00	0.00E+00	0.00E+00	0.00E+00	0.00E+00	0.00E+00	0.00E+00	0.00E+00	0.00E+00	0.00E+00	2.61E+06	1.63E+05	0.00E+00	3.26E+05	6.52E+05	0.00E+00	9.22E+05	3.34E-01
Q92841-1;Q92841-3;Q92841-2;Q92841;H3BLZ8	0.00E+00	0.00E+00	0.00E+00	0.00E+00	0.00E+00	3.37E+06	0.00E+00	0.00E+00	0.00E+00	0.00E+00	0.00E+00	0.00E+00	0.00E+00	0.00E+00	0.00E+00	0.00E+00	2.10E+05	4.21E+05	0.00E+00	8.41E+05	1.19E+06	0.00E+00	3.34E-01
H3BV04;H3BN93;H3BUB6;H3BPL9;O15127	0.00E+00	0.00E+00	0.00E+00	0.00E+00	0.00E+00	0.00E+00	0.00E+00	0.00E+00	0.00E+00	0.00E+00	0.00E+00	0.00E+00	0.00E+00	0.00E+00	0.00E+00	5.76E+05	3.60E+04	0.00E+00	7.20E+04	1.44E+05	0.00E+00	2.04E+05	3.34E-01
P80404;H3BRN4;H3BNQ7;H3BPW8	0.00E+00	0.00E+00	0.00E+00	0.00E+00	0.00E+00	0.00E+00	0.00E+00	7.82E+05	0.00E+00	0.00E+00	0.00E+00	0.00E+00	0.00E+00	0.00E+00	0.00E+00	0.00E+00	4.89E+04	9.77E+04	0.00E+00	1.95E+05	2.76E+05	0.00E+00	3.34E-01
H3BPW3;O75208-2;H3BVA5;H3BPC6;H3BRC0;H3BSJ5;H3BNT2;O75208	0.00E+00	0.00E+00	0.00E+00	0.00E+00	0.00E+00	0.00E+00	0.00E+00	0.00E+00	0.00E+00	0.00E+00	0.00E+00	0.00E+00	0.00E+00	0.00E+00	0.00E+00	7.47E+05	4.67E+04	0.00E+00	9.33E+04	1.87E+05	0.00E+00	2.64E+05	3.34E-01
H3BQI7;H3BT52;Q3SXM5-2;Q3SXM5	0.00E+00	0.00E+00	0.00E+00	0.00E+00	0.00E+00	0.00E+00	0.00E+00	0.00E+00	0.00E+00	0.00E+00	0.00E+00	0.00E+00	2.58E+06	0.00E+00	0.00E+00	0.00E+00	1.62E+05	0.00E+00	3.23E+05	6.46E+05	0.00E+00	9.14E+05	3.34E-01
H3BV15;H3BRD9;Q8N1F7-2;Q8N1F7;H3BVG0	9.71E+05	0.00E+00	0.00E+00	0.00E+00	0.00E+00	0.00E+00	0.00E+00	0.00E+00	0.00E+00	0.00E+00	0.00E+00	0.00E+00	0.00E+00	0.00E+00	0.00E+00	0.00E+00	6.07E+04	1.21E+05	0.00E+00	2.43E+05	3.43E+05	0.00E+00	3.34E-01
Q00059-2;H7BYN3;Q00059	0.00E+00	0.00E+00	0.00E+00	0.00E+00	0.00E+00	0.00E+00	0.00E+00	0.00E+00	0.00E+00	0.00E+00	0.00E+00	0.00E+00	1.46E+06	0.00E+00	0.00E+00	0.00E+00	9.11E+04	0.00E+00	1.82E+05	3.64E+05	0.00E+00	5.15E+05	3.34E-01
H7BZ81;Q6NUM9-2;Q6NUM9;H7BZ16;H7C3J0	0.00E+00	0.00E+00	0.00E+00	0.00E+00	1.01E+06	0.00E+00	0.00E+00	0.00E+00	0.00E+00	0.00E+00	0.00E+00	0.00E+00	0.00E+00	0.00E+00	0.00E+00	0.00E+00	6.28E+04	1.26E+05	0.00E+00	2.51E+05	3.55E+05	0.00E+00	3.34E-01
H7C0X8;P40121-2;P40121	0.00E+00	0.00E+00	3.53E+05	0.00E+00	0.00E+00	0.00E+00	0.00E+00	0.00E+00	0.00E+00	0.00E+00	0.00E+00	0.00E+00	0.00E+00	0.00E+00	0.00E+00	0.00E+00	2.20E+04	4.41E+04	0.00E+00	8.82E+04	1.25E+05	0.00E+00	3.34E-01
H7C4F6;O00629	0.00E+00	0.00E+00	0.00E+00	0.00E+00	0.00E+00	1.45E+06	0.00E+00	0.00E+00	0.00E+00	0.00E+00	0.00E+00	0.00E+00	0.00E+00	0.00E+00	0.00E+00	0.00E+00	9.06E+04	1.81E+05	0.00E+00	3.62E+05	5.12E+05	0.00E+00	3.34E-01
M0R2V8;H9KV91;Q96B36	2.36E+06	0.00E+00	0.00E+00	0.00E+00	0.00E+00	0.00E+00	0.00E+00	0.00E+00	0.00E+00	0.00E+00	0.00E+00	0.00E+00	0.00E+00	0.00E+00	0.00E+00	0.00E+00	1.47E+05	2.95E+05	0.00E+00	5.90E+05	8.34E+05	0.00E+00	3.34E-01
P46459-2;I3L0N3;P46459;I3L2G1;K7EQD6	0.00E+00	0.00E+00	0.00E+00	0.00E+00	0.00E+00	0.00E+00	0.00E+00	0.00E+00	0.00E+00	0.00E+00	0.00E+00	0.00E+00	1.07E+05	0.00E+00	0.00E+00	0.00E+00	6.69E+03	0.00E+00	1.34E+04	2.67E+04	0.00E+00	3.78E+04	3.34E-01
I3L0P9;Q8N137-5;Q8N137;Q8N137-3;Q8N137-2	0.00E+00	0.00E+00	0.00E+00	0.00E+00	0.00E+00	0.00E+00	0.00E+00	0.00E+00	0.00E+00	7.70E+05	0.00E+00	0.00E+00	0.00E+00	0.00E+00	0.00E+00	0.00E+00	4.81E+04	0.00E+00	9.62E+04	1.92E+05	0.00E+00	2.72E+05	3.34E-01
P46108-2;I3L297;P46108	0.00E+00	0.00E+00	0.00E+00	0.00E+00	0.00E+00	0.00E+00	0.00E+00	9.78E+05	0.00E+00	0.00E+00	0.00E+00	0.00E+00	0.00E+00	0.00E+00	0.00E+00	0.00E+00	6.11E+04	1.22E+05	0.00E+00	2.44E+05	3.46E+05	0.00E+00	3.34E-01
I3L3E9;P39748-2;P39748	0.00E+00	0.00E+00	0.00E+00	0.00E+00	0.00E+00	0.00E+00	0.00E+00	0.00E+00	0.00E+00	0.00E+00	0.00E+00	0.00E+00	0.00E+00	0.00E+00	0.00E+00	1.40E+05	8.76E+03	0.00E+00	1.75E+04	3.50E+04	0.00E+00	4.95E+04	3.34E-01
Q5VYK3;J3KN16	0.00E+00	0.00E+00	0.00E+00	0.00E+00	0.00E+00	0.00E+00	0.00E+00	0.00E+00	0.00E+00	0.00E+00	0.00E+00	0.00E+00	0.00E+00	0.00E+00	0.00E+00	1.07E+07	6.66E+05	0.00E+00	1.33E+06	2.66E+06	0.00E+00	3.77E+06	3.34E-01
J3QL05;J3KP15;Q01130-2;Q01130;Q9BRL6-2;Q9BRL6	0.00E+00	0.00E+00	0.00E+00	0.00E+00	0.00E+00	0.00E+00	0.00E+00	0.00E+00	8.08E+05	0.00E+00	0.00E+00	0.00E+00	0.00E+00	0.00E+00	0.00E+00	0.00E+00	5.05E+04	0.00E+00	1.01E+05	2.02E+05	0.00E+00	2.86E+05	3.34E-01
J3QL56;O75880	0.00E+00	0.00E+00	0.00E+00	0.00E+00	0.00E+00	0.00E+00	0.00E+00	0.00E+00	0.00E+00	0.00E+00	0.00E+00	0.00E+00	0.00E+00	4.47E+06	0.00E+00	0.00E+00	2.79E+05	0.00E+00	5.59E+05	1.12E+06	0.00E+00	1.58E+06	3.34E-01

J3QLI9;P62314	0.00E+00	0.00E+00	0.00E+00	0.00E+00	0.00E+00	0.00E+00	0.00E+00	0.00E+00	0.00E+00	0.00E+00	0.00E+00	0.00E+00	0.00E+00	0.00E+00	0.00E+00	5.42E+06	3.39E+05	0.00E+00	6.77E+05	1.35E+06	0.00E+00	1.91E+06	3.34E-01
J3QRD1;P51648;P51648-2;J3QS00;I3L1M4	0.00E+00	0.00E+00	0.00E+00	0.00E+00	0.00E+00	0.00E+00	2.71E+07	0.00E+00	0.00E+00	0.00E+00	0.00E+00	0.00E+00	0.00E+00	0.00E+00	0.00E+00	0.00E+00	1.69E+06	3.39E+06	0.00E+00	6.78E+06	9.59E+06	0.00E+00	3.34E-01
J3QT28;O43684-2;O43684;J3QSX4	0.00E+00	0.00E+00	0.00E+00	0.00E+00	0.00E+00	0.00E+00	0.00E+00	0.00E+00	0.00E+00	0.00E+00	0.00E+00	0.00E+00	6.89E+05	0.00E+00	0.00E+00	0.00E+00	4.31E+04	0.00E+00	8.61E+04	1.72E+05	0.00E+00	2.44E+05	3.34E-01
K7EM02;K7EIJ8;Q8IYT4-2;Q8IYT4	0.00E+00	0.00E+00	0.00E+00	0.00E+00	0.00E+00	0.00E+00	0.00E+00	0.00E+00	0.00E+00	0.00E+00	0.00E+00	0.00E+00	1.19E+06	0.00E+00	0.00E+00	0.00E+00	7.42E+04	0.00E+00	1.48E+05	2.97E+05	0.00E+00	4.20E+05	3.34E-01
K7EIK4;K7EL32;P52888-2;K7EL02;K7EP46;P52888	0.00E+00	0.00E+00	0.00E+00	0.00E+00	0.00E+00	0.00E+00	0.00E+00	0.00E+00	0.00E+00	0.00E+00	0.00E+00	4.58E+05	0.00E+00	0.00E+00	0.00E+00	0.00E+00	2.87E+04	0.00E+00	5.73E+04	1.15E+05	0.00E+00	1.62E+05	3.34E-01
K7EIU0;K7EQA9;Q16543	0.00E+00	0.00E+00	0.00E+00	0.00E+00	0.00E+00	0.00E+00	0.00E+00	0.00E+00	0.00E+00	1.31E+06	0.00E+00	0.00E+00	0.00E+00	0.00E+00	0.00E+00	0.00E+00	8.17E+04	0.00E+00	1.63E+05	3.27E+05	0.00E+00	4.62E+05	3.34E-01
K7EJX5;K7ELX4;P22830;P22830-2	0.00E+00	0.00E+00	0.00E+00	0.00E+00	2.40E+06	0.00E+00	0.00E+00	0.00E+00	0.00E+00	0.00E+00	0.00E+00	0.00E+00	0.00E+00	0.00E+00	0.00E+00	0.00E+00	1.50E+05	3.00E+05	0.00E+00	5.99E+05	8.48E+05	0.00E+00	3.34E-01
K7EK06;K7ER16;Q9Y285-2;Q9Y285	0.00E+00	0.00E+00	0.00E+00	0.00E+00	0.00E+00	0.00E+00	0.00E+00	0.00E+00	0.00E+00	0.00E+00	0.00E+00	0.00E+00	0.00E+00	0.00E+00	8.70E+06	0.00E+00	5.44E+05	0.00E+00	1.09E+06	2.17E+06	0.00E+00	3.08E+06	3.34E-01
K7EP07;K7EK42;Q99426-2;Q99426	0.00E+00	0.00E+00	0.00E+00	0.00E+00	0.00E+00	0.00E+00	0.00E+00	0.00E+00	0.00E+00	1.87E+06	0.00E+00	0.00E+00	0.00E+00	0.00E+00	0.00E+00	0.00E+00	1.17E+05	0.00E+00	2.34E+05	4.68E+05	0.00E+00	6.62E+05	3.34E-01
K7EM09;K7EPR0;K7ELQ9;Q6UW68	0.00E+00	0.00E+00	0.00E+00	0.00E+00	0.00E+00	0.00E+00	0.00E+00	0.00E+00	0.00E+00	0.00E+00	0.00E+00	0.00E+00	0.00E+00	0.00E+00	0.00E+00	4.18E+06	2.61E+05	0.00E+00	5.22E+05	1.04E+06	0.00E+00	1.48E+06	3.34E-01
K7EQ66;K7ELZ9;K7ENM2;K7EMW4;Q969V3-2;Q969V3	0.00E+00	0.00E+00	0.00E+00	0.00E+00	0.00E+00	0.00E+00	0.00E+00	0.00E+00	0.00E+00	0.00E+00	0.00E+00	0.00E+00	0.00E+00	0.00E+00	0.00E+00	8.99E+05	5.62E+04	0.00E+00	1.12E+05	2.25E+05	0.00E+00	3.18E+05	3.34E-01
K7ES31;K7EMQ9;K7ERF1;Q9UBQ5-2;Q9UBQ5	0.00E+00	0.00E+00	0.00E+00	0.00E+00	0.00E+00	0.00E+00	0.00E+00	0.00E+00	0.00E+00	2.66E+06	0.00E+00	0.00E+00	0.00E+00	0.00E+00	0.00E+00	0.00E+00	1.66E+05	0.00E+00	3.33E+05	6.66E+05	0.00E+00	9.42E+05	3.34E-01
K7EN20;Q9NZL4;Q9NZL4-3	0.00E+00	0.00E+00	0.00E+00	1.21E+07	0.00E+00	0.00E+00	0.00E+00	0.00E+00	0.00E+00	0.00E+00	0.00E+00	0.00E+00	0.00E+00	0.00E+00	0.00E+00	0.00E+00	7.54E+05	1.51E+06	0.00E+00	3.01E+06	4.26E+06	0.00E+00	3.34E-01
K7ENA8;O75821;K7EP16	0.00E+00	0.00E+00	0.00E+00	0.00E+00	0.00E+00	0.00E+00	0.00E+00	0.00E+00	0.00E+00	0.00E+00	0.00E+00	0.00E+00	1.50E+06	0.00E+00	0.00E+00	0.00E+00	9.36E+04	0.00E+00	1.87E+05	3.75E+05	0.00E+00	5.30E+05	3.34E-01
K7ENG2;P26368-2;P26368	0.00E+00	0.00E+00	0.00E+00	0.00E+00	5.48E+06	0.00E+00	0.00E+00	0.00E+00	0.00E+00	0.00E+00	0.00E+00	0.00E+00	0.00E+00	0.00E+00	0.00E+00	0.00E+00	3.43E+05	6.85E+05	0.00E+00	1.37E+06	1.94E+06	0.00E+00	3.34E-01
K7ENW7;P26358-3;P26358;P26358-2	0.00E+00	0.00E+00	0.00E+00	0.00E+00	0.00E+00	0.00E+00	0.00E+00	0.00E+00	0.00E+00	0.00E+00	0.00E+00	1.87E+06	0.00E+00	0.00E+00	0.00E+00	0.00E+00	1.17E+05	0.00E+00	2.34E+05	4.67E+05	0.00E+00	6.61E+05	3.34E-01
K7EPL2;Q9UBT2;U3KQ55;K7ES38	0.00E+00	0.00E+00	0.00E+00	0.00E+00	0.00E+00	2.72E+07	0.00E+00	0.00E+00	0.00E+00	0.00E+00	0.00E+00	0.00E+00	0.00E+00	0.00E+00	0.00E+00	0.00E+00	1.70E+06	3.40E+06	0.00E+00	6.81E+06	9.62E+06	0.00E+00	3.34E-01
K7EQG1;Q9NXS2-3;Q9NXS2	0.00E+00	0.00E+00	0.00E+00	0.00E+00	0.00E+00	0.00E+00	0.00E+00	0.00E+00	0.00E+00	0.00E+00	4.75E+06	0.00E+00	0.00E+00	0.00E+00	0.00E+00	0.00E+00	2.97E+05	0.00E+00	5.93E+05	1.19E+06	0.00E+00	1.68E+06	3.34E-01
K7ERE3;P13646-3;P13646;P13646-2;K7EMD9	0.00E+00	0.00E+00	0.00E+00	0.00E+00	0.00E+00	0.00E+00	0.00E+00	6.41E+06	0.00E+00	0.00E+00	0.00E+00	0.00E+00	0.00E+00	0.00E+00	0.00E+00	0.00E+00	4.01E+05	8.01E+05	0.00E+00	1.60E+06	2.27E+06	0.00E+00	3.34E-01
K7ERX7;K7EQH4	0.00E+00	0.00E+00	0.00E+00	0.00E+00	0.00E+00	0.00E+00	0.00E+00	0.00E+00	0.00E+00	0.00E+00	0.00E+00	0.00E+00	0.00E+00	0.00E+00	0.00E+00	1.63E+06	1.02E+05	0.00E+00	2.04E+05	4.08E+05	0.00E+00	5.76E+05	3.34E-01
K7ES67;Q96CS2-2;Q96CS2	0.00E+00	0.00E+00	0.00E+00	0.00E+00	0.00E+00	0.00E+00	0.00E+00	0.00E+00	0.00E+00	0.00E+00	0.00E+00	0.00E+00	2.39E+05	0.00E+00	0.00E+00	0.00E+00	1.49E+04	0.00E+00	2.99E+04	5.98E+04	0.00E+00	8.45E+04	3.34E-01
M0R2U2;M0R1H0;M0R2B0;M0R0P1;M0R299;M0QXL5;M0R2Q4;P22087	0.00E+00	0.00E+00	0.00E+00	0.00E+00	0.00E+00	0.00E+00	0.00E+00	0.00E+00	0.00E+00	0.00E+00	0.00E+00	0.00E+00	2.96E+05	0.00E+00	0.00E+00	0.00E+00	1.85E+04	0.00E+00	3.70E+04	7.39E+04	0.00E+00	1.05E+05	3.34E-01
M0QXN5;P37198	0.00E+00	0.00E+00	0.00E+00	0.00E+00	0.00E+00	0.00E+00	0.00E+00	0.00E+00	0.00E+00	0.00E+00	0.00E+00	0.00E+00	2.75E+06	0.00E+00	0.00E+00	0.00E+00	1.72E+05	0.00E+00	3.44E+05	6.87E+05	0.00E+00	9.72E+05	3.34E-01
M0QY97;Q9UPT8	0.00E+00	0.00E+00	0.00E+00	0.00E+00	0.00E+00	0.00E+00	0.00E+00	0.00E+00	0.00E+00	0.00E+00	0.00E+00	0.00E+00	0.00E+00	0.00E+00	0.00E+00	1.93E+06	1.21E+05	0.00E+00	2.41E+05	4.83E+05	0.00E+00	6.83E+05	3.34E-01
M0R0P7;M0R3D6;M0R1A7;M0R117;Q02543	0.00E+00	0.00E+00	0.00E+00	0.00E+00	0.00E+00	0.00E+00	0.00E+00	0.00E+00	0.00E+00	0.00E+00	0.00E+00	0.00E+00	0.00E+00	0.00E+00	9.97E+05	0.00E+00	6.23E+04	0.00E+00	1.25E+05	2.49E+05	0.00E+00	3.52E+05	3.34E-01
M0R0Y2;P54920;M0R2M1	0.00E+00	0.00E+00	0.00E+00	0.00E+00	0.00E+00	0.00E+00	0.00E+00	0.00E+00	0.00E+00	0.00E+00	0.00E+00	3.12E+06	0.00E+00	0.00E+00	0.00E+00	0.00E+00	1.95E+05	0.00E+00	3.90E+05	7.81E+05	0.00E+00	1.10E+06	3.34E-01
M0R192;P30043	0.00E+00	0.00E+00	0.00E+00	0.00E+00	0.00E+00	0.00E+00	0.00E+00	0.00E+00	0.00E+00	0.00E+00	0.00E+00	2.24E+05	0.00E+00	0.00E+00	0.00E+00	0.00E+00	1.40E+04	0.00E+00	2.80E+04	5.60E+04	0.00E+00	7.92E+04	3.34E-01
M0R208;Q16740	0.00E+00	0.00E+00	0.00E+00	0.00E+00	0.00E+00	0.00E+00	0.00E+00	0.00E+00	0.00E+00	0.00E+00	0.00E+00	0.00E+00	0.00E+00	0.00E+00	0.00E+00	1.08E+06	6.76E+04	0.00E+00	1.35E+05	2.70E+05	0.00E+00	3.82E+05	3.34E-01

Q15642-5;M0R2H7;Q15642-4;Q15642-2;W4VSQ9;Q15642-3;Q15642	0.00E+00	0.00E+00	0.00E+00	0.00E+00	0.00E+00	0.00E+00	0.00E+00	0.00E+00	0.00E+00	0.00E+00	0.00E+00	0.00E+00	0.00E+00	0.00E+00	0.00E+00	1.37E+06	8.55E+04	0.00E+00	1.71E+05	3.42E+05	0.00E+00	4.84E+05	3.34E-01
M0R389;Q15102;M0QXS6;M0R323;M0QZT2	0.00E+00	0.00E+00	0.00E+00	0.00E+00	0.00E+00	0.00E+00	0.00E+00	0.00E+00	0.00E+00	0.00E+00	0.00E+00	0.00E+00	0.00E+00	1.73E+06	0.00E+00	0.00E+00	1.08E+05	0.00E+00	2.16E+05	4.32E+05	0.00E+00	6.11E+05	3.34E-01
P67870;Q5SRQ3;Q5SRQ6;N0E472	0.00E+00	0.00E+00	0.00E+00	0.00E+00	0.00E+00	0.00E+00	0.00E+00	0.00E+00	0.00E+00	6.32E+06	0.00E+00	0.00E+00	0.00E+00	0.00E+00	0.00E+00	0.00E+00	3.95E+05	0.00E+00	7.89E+05	1.58E+06	0.00E+00	2.23E+06	3.34E-01
O00186	0.00E+00	0.00E+00	0.00E+00	0.00E+00	0.00E+00	0.00E+00	2.77E+05	0.00E+00	0.00E+00	0.00E+00	0.00E+00	0.00E+00	0.00E+00	0.00E+00	0.00E+00	0.00E+00	1.73E+04	3.46E+04	0.00E+00	6.92E+04	9.79E+04	0.00E+00	3.34E-01
O00303	0.00E+00	0.00E+00	0.00E+00	0.00E+00	0.00E+00	0.00E+00	0.00E+00	0.00E+00	0.00E+00	0.00E+00	0.00E+00	0.00E+00	0.00E+00	1.84E+06	0.00E+00	0.00E+00	1.15E+05	0.00E+00	2.29E+05	4.59E+05	0.00E+00	6.49E+05	3.34E-01
O00411	0.00E+00	0.00E+00	0.00E+00	0.00E+00	0.00E+00	0.00E+00	8.19E+06	0.00E+00	0.00E+00	0.00E+00	0.00E+00	0.00E+00	0.00E+00	0.00E+00	0.00E+00	0.00E+00	5.12E+05	1.02E+06	0.00E+00	2.05E+06	2.90E+06	0.00E+00	3.34E-01
O00425	0.00E+00	0.00E+00	0.00E+00	0.00E+00	3.28E+06	0.00E+00	0.00E+00	0.00E+00	0.00E+00	0.00E+00	0.00E+00	0.00E+00	0.00E+00	0.00E+00	0.00E+00	0.00E+00	2.05E+05	4.11E+05	0.00E+00	8.21E+05	1.16E+06	0.00E+00	3.34E-01
O00487	0.00E+00	0.00E+00	0.00E+00	0.00E+00	0.00E+00	2.98E+06	0.00E+00	0.00E+00	0.00E+00	0.00E+00	0.00E+00	0.00E+00	0.00E+00	0.00E+00	0.00E+00	0.00E+00	1.86E+05	3.73E+05	0.00E+00	7.45E+05	1.05E+06	0.00E+00	3.34E-01
O00560-2;O00560;B4DHN5;E9PBU7;O00560-3;G5EA09	0.00E+00	3.59E+06	0.00E+00	0.00E+00	0.00E+00	0.00E+00	0.00E+00	0.00E+00	0.00E+00	0.00E+00	0.00E+00	0.00E+00	0.00E+00	0.00E+00	0.00E+00	0.00E+00	2.25E+05	4.49E+05	0.00E+00	8.98E+05	1.27E+06	0.00E+00	3.34E-01
O00767	0.00E+00	0.00E+00	0.00E+00	0.00E+00	0.00E+00	0.00E+00	0.00E+00	0.00E+00	0.00E+00	0.00E+00	0.00E+00	0.00E+00	0.00E+00	0.00E+00	0.00E+00	1.70E+06	1.07E+05	0.00E+00	2.13E+05	4.26E+05	0.00E+00	6.03E+05	3.34E-01
O14617-4;O14617;O14617-5;O14617-3;O14617-2	0.00E+00	0.00E+00	0.00E+00	0.00E+00	0.00E+00	0.00E+00	0.00E+00	0.00E+00	0.00E+00	0.00E+00	0.00E+00	5.68E+05	0.00E+00	0.00E+00	0.00E+00	0.00E+00	3.55E+04	0.00E+00	7.10E+04	1.42E+05	0.00E+00	2.01E+05	3.34E-01
O14656	0.00E+00	0.00E+00	0.00E+00	0.00E+00	0.00E+00	0.00E+00	0.00E+00	0.00E+00	0.00E+00	0.00E+00	0.00E+00	0.00E+00	0.00E+00	2.64E+06	0.00E+00	0.00E+00	1.65E+05	0.00E+00	3.30E+05	6.59E+05	0.00E+00	9.33E+05	3.34E-01
O14745;J3QRP6	0.00E+00	0.00E+00	0.00E+00	0.00E+00	0.00E+00	0.00E+00	4.99E+06	0.00E+00	0.00E+00	0.00E+00	0.00E+00	0.00E+00	0.00E+00	0.00E+00	0.00E+00	0.00E+00	3.12E+05	6.24E+05	0.00E+00	1.25E+06	1.76E+06	0.00E+00	3.34E-01
O14773-2;O14773	0.00E+00	0.00E+00	0.00E+00	0.00E+00	0.00E+00	0.00E+00	0.00E+00	0.00E+00	0.00E+00	0.00E+00	0.00E+00	3.23E+06	0.00E+00	0.00E+00	0.00E+00	0.00E+00	2.02E+05	0.00E+00	4.03E+05	8.07E+05	0.00E+00	1.14E+06	3.34E-01
O14925;Q5SRD1;B4DDK6	0.00E+00	0.00E+00	0.00E+00	0.00E+00	0.00E+00	0.00E+00	0.00E+00	0.00E+00	0.00E+00	3.28E+06	0.00E+00	0.00E+00	0.00E+00	0.00E+00	0.00E+00	0.00E+00	2.05E+05	0.00E+00	4.10E+05	8.20E+05	0.00E+00	1.16E+06	3.34E-01
O15042-2;O15042;U3KPT1;C9JDJ7;C9J5L1;E7EW00;H0Y8D9;C9JB80;O15042-3;E7ET15	0.00E+00	0.00E+00	0.00E+00	0.00E+00	0.00E+00	0.00E+00	2.19E+06	0.00E+00	0.00E+00	0.00E+00	0.00E+00	0.00E+00	0.00E+00	0.00E+00	0.00E+00	0.00E+00	1.37E+05	2.74E+05	0.00E+00	5.48E+05	7.75E+05	0.00E+00	3.34E-01
O15050	0.00E+00	0.00E+00	0.00E+00	0.00E+00	0.00E+00	0.00E+00	0.00E+00	0.00E+00	0.00E+00	0.00E+00	0.00E+00	1.24E+06	0.00E+00	0.00E+00	0.00E+00	0.00E+00	7.74E+04	0.00E+00	1.55E+05	3.10E+05	0.00E+00	4.38E+05	3.34E-01
O15067;J3KTQ5	0.00E+00	0.00E+00	0.00E+00	0.00E+00	0.00E+00	0.00E+00	0.00E+00	3.44E+06	0.00E+00	0.00E+00	0.00E+00	0.00E+00	0.00E+00	0.00E+00	0.00E+00	0.00E+00	2.15E+05	4.30E+05	0.00E+00	8.61E+05	1.22E+06	0.00E+00	3.34E-01
O15118	0.00E+00	0.00E+00	0.00E+00	0.00E+00	0.00E+00	0.00E+00	0.00E+00	3.51E+06	0.00E+00	0.00E+00	0.00E+00	0.00E+00	0.00E+00	0.00E+00	0.00E+00	0.00E+00	2.20E+05	4.39E+05	0.00E+00	8.78E+05	1.24E+06	0.00E+00	3.34E-01
O15143;C9JTT6;C9JBJ7;C9K057;C9J4Z7;F8VXW2;C9JEY1;C9JQM8;C9JFG9;C9J6C8	0.00E+00	0.00E+00	0.00E+00	0.00E+00	9.66E+05	0.00E+00	0.00E+00	0.00E+00	0.00E+00	0.00E+00	0.00E+00	0.00E+00	0.00E+00	0.00E+00	0.00E+00	0.00E+00	6.04E+04	1.21E+05	0.00E+00	2.42E+05	3.42E+05	0.00E+00	3.34E-01
O15144	0.00E+00	0.00E+00	4.73E+05	0.00E+00	0.00E+00	0.00E+00	0.00E+00	0.00E+00	0.00E+00	0.00E+00	0.00E+00	0.00E+00	0.00E+00	0.00E+00	0.00E+00	0.00E+00	2.96E+04	5.92E+04	0.00E+00	1.18E+05	1.67E+05	0.00E+00	3.34E-01
O15355	0.00E+00	0.00E+00	0.00E+00	0.00E+00	0.00E+00	0.00E+00	0.00E+00	4.44E+06	0.00E+00	0.00E+00	0.00E+00	0.00E+00	0.00E+00	0.00E+00	0.00E+00	0.00E+00	2.77E+05	5.54E+05	0.00E+00	1.11E+06	1.57E+06	0.00E+00	3.34E-01
O15460-2;O15460	0.00E+00	0.00E+00	0.00E+00	0.00E+00	0.00E+00	5.38E+06	0.00E+00	0.00E+00	0.00E+00	0.00E+00	0.00E+00	0.00E+00	0.00E+00	0.00E+00	0.00E+00	0.00E+00	3.36E+05	6.72E+05	0.00E+00	1.34E+06	1.90E+06	0.00E+00	3.34E-01
Q5SZU1;O43175	0.00E+00	0.00E+00	0.00E+00	0.00E+00	0.00E+00	0.00E+00	1.22E+06	0.00E+00	0.00E+00	0.00E+00	0.00E+00	0.00E+00	0.00E+00	0.00E+00	0.00E+00	0.00E+00	7.66E+04	1.53E+05	0.00E+00	3.06E+05	4.33E+05	0.00E+00	3.34E-01
O43242;O43242-2	0.00E+00	0.00E+00	0.00E+00	0.00E+00	0.00E+00	0.00E+00	0.00E+00	0.00E+00	0.00E+00	0.00E+00	0.00E+00	0.00E+00	1.52E+06	0.00E+00	0.00E+00	0.00E+00	9.52E+04	0.00E+00	1.90E+05	3.81E+05	0.00E+00	5.39E+05	3.34E-01
O43395	0.00E+00	0.00E+00	0.00E+00	5.99E+06	0.00E+00	0.00E+00	0.00E+00	0.00E+00	0.00E+00	0.00E+00	0.00E+00	0.00E+00	0.00E+00	0.00E+00	0.00E+00	0.00E+00	3.74E+05	7.48E+05	0.00E+00	1.50E+06	2.12E+06	0.00E+00	3.34E-01
O43396;K7ER96;K7EML9	0.00E+00	0.00E+00	0.00E+00	0.00E+00	0.00E+00	0.00E+00	0.00E+00	0.00E+00	0.00E+00	0.00E+00	0.00E+00	0.00E+00	0.00E+00	0.00E+00	0.00E+00	1.55E+07	9.70E+05	0.00E+00	1.94E+06	3.88E+06	0.00E+00	5.49E+06	3.34E-01
O43464-3;O43464;O43464-2;O43464-4	0.00E+00	0.00E+00	0.00E+00	0.00E+00	0.00E+00	0.00E+00	0.00E+00	3.37E+06	0.00E+00	0.00E+00	0.00E+00	0.00E+00	0.00E+00	0.00E+00	0.00E+00	0.00E+00	2.11E+05	4.22E+05	0.00E+00	8.43E+05	1.19E+06	0.00E+00	3.34E-01
O43707;O43707-2;O43707-3;F5GXS2	0.00E+00	0.00E+00	0.00E+00	0.00E+00	0.00E+00	0.00E+00	0.00E+00	0.00E+00	0.00E+00	0.00E+00	0.00E+00	0.00E+00	0.00E+00	0.00E+00	1.30E+07	0.00E+00	8.10E+05	0.00E+00	1.62E+06	3.24E+06	0.00E+00	4.58E+06	3.34E-01
O43719	0.00E+00	1.85E+06	0.00E+00	0.00E+00	0.00E+00	0.00E+00	0.00E+00	0.00E+00	0.00E+00	0.00E+00	0.00E+00	0.00E+00	0.00E+00	0.00E+00	0.00E+00	0.00E+00	1.16E+05	2.31E+05	0.00E+00	4.63E+05	6.55E+05	0.00E+00	3.34E-01

O43760;O43760-2;K7ELD9;K7ENG9;K7EJ35	0.00E+00	0.00E+00	0.00E+00	0.00E+00	0.00E+00	0.00E+00	2.02E+07	0.00E+00	0.00E+00	0.00E+00	0.00E+00	0.00E+00	0.00E+00	0.00E+00	0.00E+00	0.00E+00	1.26E+06	2.53E+06	0.00E+00	5.06E+06	7.15E+06	0.00E+00	3.34E-01
O43776	0.00E+00	0.00E+00	0.00E+00	0.00E+00	0.00E+00	0.00E+00	0.00E+00	0.00E+00	0.00E+00	0.00E+00	0.00E+00	2.00E+05	0.00E+00	0.00E+00	0.00E+00	0.00E+00	1.25E+04	0.00E+00	2.50E+04	5.00E+04	0.00E+00	7.07E+04	3.34E-01
O43790	0.00E+00	0.00E+00	0.00E+00	0.00E+00	0.00E+00	0.00E+00	0.00E+00	0.00E+00	0.00E+00	0.00E+00	0.00E+00	0.00E+00	0.00E+00	1.12E+07	0.00E+00	0.00E+00	7.01E+05	0.00E+00	1.40E+06	2.80E+06	0.00E+00	3.96E+06	3.34E-01
O43823	0.00E+00	0.00E+00	0.00E+00	0.00E+00	0.00E+00	0.00E+00	0.00E+00	0.00E+00	0.00E+00	0.00E+00	0.00E+00	0.00E+00	0.00E+00	0.00E+00	0.00E+00	3.16E+05	1.97E+04	0.00E+00	3.95E+04	7.89E+04	0.00E+00	1.12E+05	3.34E-01
O43852-13;O43852-14;O43852-11;O43852-10;O43852-5;O43852-6;O43852;O43852-3	0.00E+00	0.00E+00	0.00E+00	0.00E+00	0.00E+00	7.92E+05	0.00E+00	0.00E+00	0.00E+00	0.00E+00	0.00E+00	0.00E+00	0.00E+00	0.00E+00	0.00E+00	0.00E+00	4.95E+04	9.90E+04	0.00E+00	1.98E+05	2.80E+05	0.00E+00	3.34E-01
O60264	0.00E+00	0.00E+00	0.00E+00	0.00E+00	0.00E+00	0.00E+00	0.00E+00	0.00E+00	0.00E+00	0.00E+00	0.00E+00	0.00E+00	0.00E+00	0.00E+00	0.00E+00	3.15E+06	1.97E+05	0.00E+00	3.94E+05	7.88E+05	0.00E+00	1.11E+06	3.34E-01
O60268-2;O60268-3;O60268	0.00E+00	0.00E+00	0.00E+00	0.00E+00	0.00E+00	0.00E+00	0.00E+00	0.00E+00	0.00E+00	0.00E+00	0.00E+00	0.00E+00	0.00E+00	0.00E+00	0.00E+00	8.08E+05	5.05E+04	0.00E+00	1.01E+05	2.02E+05	0.00E+00	2.86E+05	3.34E-01
O60306	0.00E+00	0.00E+00	0.00E+00	0.00E+00	0.00E+00	0.00E+00	0.00E+00	0.00E+00	0.00E+00	0.00E+00	0.00E+00	0.00E+00	0.00E+00	0.00E+00	0.00E+00	1.71E+06	1.07E+05	0.00E+00	2.14E+05	4.28E+05	0.00E+00	6.06E+05	3.34E-01
O60502;O60502-3;O60502-4	0.00E+00	0.00E+00	0.00E+00	0.00E+00	9.27E+06	0.00E+00	0.00E+00	0.00E+00	0.00E+00	0.00E+00	0.00E+00	0.00E+00	0.00E+00	0.00E+00	0.00E+00	0.00E+00	5.79E+05	1.16E+06	0.00E+00	2.32E+06	3.28E+06	0.00E+00	3.34E-01
O60884	0.00E+00	0.00E+00	0.00E+00	0.00E+00	0.00E+00	0.00E+00	0.00E+00	0.00E+00	0.00E+00	0.00E+00	5.44E+05	0.00E+00	0.00E+00	0.00E+00	0.00E+00	0.00E+00	3.40E+04	0.00E+00	6.80E+04	1.36E+05	0.00E+00	1.92E+05	3.34E-01
O75083;D6RD66;O75083-3	0.00E+00	0.00E+00	0.00E+00	4.29E+06	0.00E+00	0.00E+00	0.00E+00	0.00E+00	0.00E+00	0.00E+00	0.00E+00	0.00E+00	0.00E+00	0.00E+00	0.00E+00	0.00E+00	2.68E+05	5.37E+05	0.00E+00	1.07E+06	1.52E+06	0.00E+00	3.34E-01
O75165	0.00E+00	0.00E+00	0.00E+00	0.00E+00	0.00E+00	0.00E+00	0.00E+00	0.00E+00	0.00E+00	0.00E+00	0.00E+00	0.00E+00	0.00E+00	0.00E+00	0.00E+00	3.23E+06	2.02E+05	0.00E+00	4.04E+05	8.07E+05	0.00E+00	1.14E+06	3.34E-01
O75306-2;O75306	0.00E+00	0.00E+00	0.00E+00	0.00E+00	6.45E+06	0.00E+00	0.00E+00	0.00E+00	0.00E+00	0.00E+00	0.00E+00	0.00E+00	0.00E+00	0.00E+00	0.00E+00	0.00E+00	4.03E+05	8.06E+05	0.00E+00	1.61E+06	2.28E+06	0.00E+00	3.34E-01
S4R3Q6;O75436-2;O75436	0.00E+00	0.00E+00	0.00E+00	0.00E+00	0.00E+00	3.27E+06	0.00E+00	0.00E+00	0.00E+00	0.00E+00	0.00E+00	0.00E+00	0.00E+00	0.00E+00	0.00E+00	0.00E+00	2.05E+05	4.09E+05	0.00E+00	8.19E+05	1.16E+06	0.00E+00	3.34E-01
O75475	0.00E+00	0.00E+00	0.00E+00	0.00E+00	0.00E+00	0.00E+00	0.00E+00	0.00E+00	0.00E+00	0.00E+00	0.00E+00	0.00E+00	2.01E+05	0.00E+00	0.00E+00	0.00E+00	1.26E+04	0.00E+00	2.52E+04	5.03E+04	0.00E+00	7.12E+04	3.34E-01
O75477;B0QZ43	0.00E+00	0.00E+00	0.00E+00	0.00E+00	0.00E+00	0.00E+00	0.00E+00	0.00E+00	0.00E+00	0.00E+00	0.00E+00	0.00E+00	0.00E+00	6.57E+05	0.00E+00	0.00E+00	4.11E+04	0.00E+00	8.22E+04	1.64E+05	0.00E+00	2.32E+05	3.34E-01
O75844	0.00E+00	0.00E+00	0.00E+00	0.00E+00	0.00E+00	0.00E+00	0.00E+00	0.00E+00	0.00E+00	0.00E+00	0.00E+00	0.00E+00	0.00E+00	0.00E+00	0.00E+00	7.34E+05	4.59E+04	0.00E+00	9.18E+04	1.84E+05	0.00E+00	2.60E+05	3.34E-01
O75874	0.00E+00	0.00E+00	0.00E+00	0.00E+00	0.00E+00	0.00E+00	1.00E+08	0.00E+00	0.00E+00	0.00E+00	0.00E+00	0.00E+00	0.00E+00	0.00E+00	0.00E+00	0.00E+00	6.25E+06	1.25E+07	0.00E+00	2.50E+07	3.53E+07	0.00E+00	3.34E-01
O75891-2;O75891;O75891-3	0.00E+00	0.00E+00	0.00E+00	0.00E+00	0.00E+00	0.00E+00	0.00E+00	0.00E+00	0.00E+00	0.00E+00	0.00E+00	0.00E+00	6.37E+05	0.00E+00	0.00E+00	0.00E+00	3.98E+04	0.00E+00	7.96E+04	1.59E+05	0.00E+00	2.25E+05	3.34E-01
O76013-2;O76013;O76015	0.00E+00	0.00E+00	0.00E+00	0.00E+00	0.00E+00	1.88E+06	0.00E+00	0.00E+00	0.00E+00	0.00E+00	0.00E+00	0.00E+00	0.00E+00	0.00E+00	0.00E+00	0.00E+00	1.17E+05	2.34E+05	0.00E+00	4.69E+05	6.63E+05	0.00E+00	3.34E-01
O76094-2;O76094;R4GNC1;D6RDY6	3.69E+06	0.00E+00	0.00E+00	0.00E+00	0.00E+00	0.00E+00	0.00E+00	0.00E+00	0.00E+00	0.00E+00	0.00E+00	0.00E+00	0.00E+00	0.00E+00	0.00E+00	0.00E+00	2.31E+05	4.62E+05	0.00E+00	9.23E+05	1.31E+06	0.00E+00	3.34E-01
O94776	0.00E+00	0.00E+00	0.00E+00	0.00E+00	0.00E+00	0.00E+00	1.55E+07	0.00E+00	0.00E+00	0.00E+00	0.00E+00	0.00E+00	0.00E+00	0.00E+00	0.00E+00	0.00E+00	9.66E+05	1.93E+06	0.00E+00	3.86E+06	5.46E+06	0.00E+00	3.34E-01
O94874-3;O94874-2;O94874	0.00E+00	0.00E+00	0.00E+00	0.00E+00	0.00E+00	0.00E+00	7.89E+05	0.00E+00	0.00E+00	0.00E+00	0.00E+00	0.00E+00	0.00E+00	0.00E+00	0.00E+00	0.00E+00	4.93E+04	9.86E+04	0.00E+00	1.97E+05	2.79E+05	0.00E+00	3.34E-01
O94973-3;O94973;O94973-2;O95782-2;O95782	0.00E+00	0.00E+00	0.00E+00	0.00E+00	0.00E+00	0.00E+00	0.00E+00	0.00E+00	0.00E+00	0.00E+00	0.00E+00	0.00E+00	0.00E+00	0.00E+00	0.00E+00	2.48E+06	1.55E+05	0.00E+00	3.10E+05	6.20E+05	0.00E+00	8.77E+05	3.34E-01
O95147	0.00E+00	0.00E+00	0.00E+00	0.00E+00	0.00E+00	1.76E+06	0.00E+00	0.00E+00	0.00E+00	0.00E+00	0.00E+00	0.00E+00	0.00E+00	0.00E+00	0.00E+00	0.00E+00	1.10E+05	2.20E+05	0.00E+00	4.40E+05	6.23E+05	0.00E+00	3.34E-01
O95347-2;O95347	0.00E+00	0.00E+00	0.00E+00	0.00E+00	0.00E+00	0.00E+00	0.00E+00	0.00E+00	0.00E+00	0.00E+00	0.00E+00	0.00E+00	0.00E+00	0.00E+00	0.00E+00	2.79E+05	1.74E+04	0.00E+00	3.49E+04	6.97E+04	0.00E+00	9.86E+04	3.34E-01
O95573	0.00E+00	0.00E+00	0.00E+00	0.00E+00	0.00E+00	0.00E+00	1.41E+06	0.00E+00	0.00E+00	0.00E+00	0.00E+00	0.00E+00	0.00E+00	0.00E+00	0.00E+00	0.00E+00	8.84E+04	1.77E+05	0.00E+00	3.54E+05	5.00E+05	0.00E+00	3.34E-01
O95671-3;O95671-2;O95671	0.00E+00	0.00E+00	0.00E+00	0.00E+00	0.00E+00	0.00E+00	0.00E+00	0.00E+00	0.00E+00	0.00E+00	0.00E+00	0.00E+00	3.32E+05	0.00E+00	0.00E+00	0.00E+00	2.08E+04	0.00E+00	4.15E+04	8.31E+04	0.00E+00	1.17E+05	3.34E-01
O95678	0.00E+00	0.00E+00	0.00E+00	0.00E+00	0.00E+00	0.00E+00	0.00E+00	0.00E+00	0.00E+00	0.00E+00	0.00E+00	0.00E+00	0.00E+00	3.01E+06	0.00E+00	0.00E+00	1.88E+05	0.00E+00	3.77E+05	7.53E+05	0.00E+00	1.07E+06	3.34E-01
O95716	0.00E+00	0.00E+00	0.00E+00	0.00E+00	0.00E+00	1.80E+06	0.00E+00	0.00E+00	0.00E+00	0.00E+00	0.00E+00	0.00E+00	0.00E+00	0.00E+00	0.00E+00	0.00E+00	1.12E+05	2.25E+05	0.00E+00	4.50E+05	6.36E+05	0.00E+00	3.34E-01
O96019-2;O96019	0.00E+00	0.00E+00	0.00E+00	0.00E+00	0.00E+00	0.00E+00	0.00E+00	0.00E+00	0.00E+00	0.00E+00	0.00E+00	0.00E+00	0.00E+00	0.00E+00	5.33E+06	0.00E+00	3.33E+05	0.00E+00	6.66E+05	1.33E+06	0.00E+00	1.88E+06	3.34E-01
P00403	0.00E+00	0.00E+00	0.00E+00	0.00E+00	0.00E+00	5.94E+06	0.00E+00	0.00E+00	0.00E+00	0.00E+00	0.00E+00	0.00E+00	0.00E+00	0.00E+00	0.00E+00	0.00E+00	3.71E+05	7.42E+05	0.00E+00	1.48E+06	2.10E+06	0.00E+00	3.34E-01
P00846	0.00E+00	0.00E+00	0.00E+00	0.00E+00	0.00E+00	0.00E+00	0.00E+00	0.00E+00	0.00E+00	0.00E+00	0.00E+00	0.00E+00	0.00E+00	0.00E+00	0.00E+00	3.59E+06	2.24E+05	0.00E+00	4.48E+05	8.96E+05	0.00E+00	1.27E+06	3.34E-01
Q5H9A7;P01033	0.00E+00	0.00E+00	4.08E+05	0.00E+00	0.00E+00	0.00E+00	0.00E+00	0.00E+00	0.00E+00	0.00E+00	0.00E+00	0.00E+00	0.00E+00	0.00E+00	0.00E+00	0.00E+00	2.55E+04	5.10E+04	0.00E+00	1.02E+05	1.44E+05	0.00E+00	3.34E-01
P02545-2;Q3BDU5;Q5TCI8	0.00E+00	0.00E+00	0.00E+00	0.00E+00	0.00E+00	1.55E+07	0.00E+00	0.00E+00	0.00E+00	0.00E+00	0.00E+00	0.00E+00	0.00E+00	0.00E+00	0.00E+00	0.00E+00	9.71E+05	1.94E+06	0.00E+00	3.88E+06	5.49E+06	0.00E+00	3.34E-01

P04040	0.00E+00	0.00E+00	0.00E+00	0.00E+00	0.00E+00	0.00E+00	0.00E+00	0.00E+00	0.00E+00	0.00E+00	0.00E+00	0.00E+00	1.32E+06	0.00E+00	0.00E+00	0.00E+00	8.23E+04	0.00E+00	1.65E+05	3.29E+05	0.00E+00	4.66E+05	3.34E-01
P04350	0.00E+00	0.00E+00	0.00E+00	0.00E+00	0.00E+00	0.00E+00	0.00E+00	0.00E+00	0.00E+00	0.00E+00	0.00E+00	0.00E+00	0.00E+00	0.00E+00	2.02E+06	0.00E+00	1.26E+05	0.00E+00	2.53E+05	5.06E+05	0.00E+00	7.15E+05	3.34E-01
P04818-2;P04818;P04818-3	0.00E+00	0.00E+00	0.00E+00	0.00E+00	0.00E+00	4.14E+05	0.00E+00	0.00E+00	0.00E+00	0.00E+00	0.00E+00	0.00E+00	0.00E+00	0.00E+00	0.00E+00	0.00E+00	2.59E+04	5.18E+04	0.00E+00	1.04E+05	1.46E+05	0.00E+00	3.34E-01
P04899;P04899-4;P04899-6;P04899-3;P04899-5;P04899-2;P63096	0.00E+00	0.00E+00	0.00E+00	0.00E+00	0.00E+00	0.00E+00	0.00E+00	0.00E+00	0.00E+00	0.00E+00	0.00E+00	9.89E+05	0.00E+00	0.00E+00	0.00E+00	0.00E+00	6.18E+04	0.00E+00	1.24E+05	2.47E+05	0.00E+00	3.50E+05	3.34E-01
S4R3S4;P05091-2;P05091	0.00E+00	0.00E+00	0.00E+00	0.00E+00	0.00E+00	0.00E+00	0.00E+00	0.00E+00	0.00E+00	0.00E+00	0.00E+00	0.00E+00	0.00E+00	1.67E+06	0.00E+00	0.00E+00	1.04E+05	0.00E+00	2.08E+05	4.17E+05	0.00E+00	5.89E+05	3.34E-01
P06702	0.00E+00	0.00E+00	0.00E+00	0.00E+00	0.00E+00	0.00E+00	0.00E+00	0.00E+00	0.00E+00	0.00E+00	0.00E+00	6.67E+06	0.00E+00	0.00E+00	0.00E+00	0.00E+00	4.17E+05	0.00E+00	8.34E+05	1.67E+06	0.00E+00	2.36E+06	3.34E-01
P06756-3;P06756-2;P06756	0.00E+00	0.00E+00	0.00E+00	0.00E+00	0.00E+00	0.00E+00	0.00E+00	0.00E+00	0.00E+00	0.00E+00	0.00E+00	0.00E+00	6.23E+05	0.00E+00	0.00E+00	0.00E+00	3.89E+04	0.00E+00	7.78E+04	1.56E+05	0.00E+00	2.20E+05	3.34E-01
P07858	0.00E+00	0.00E+00	7.78E+05	0.00E+00	0.00E+00	0.00E+00	0.00E+00	0.00E+00	0.00E+00	0.00E+00	0.00E+00	0.00E+00	0.00E+00	0.00E+00	0.00E+00	0.00E+00	4.86E+04	9.72E+04	0.00E+00	1.94E+05	2.75E+05	0.00E+00	3.34E-01
Q5TCU3;P07951-2;P07951-3;P07951;Q5TCU8	0.00E+00	0.00E+00	0.00E+00	0.00E+00	0.00E+00	0.00E+00	0.00E+00	0.00E+00	0.00E+00	0.00E+00	0.00E+00	4.91E+05	0.00E+00	0.00E+00	0.00E+00	0.00E+00	3.07E+04	0.00E+00	6.14E+04	1.23E+05	0.00E+00	1.74E+05	3.34E-01
P08243-3;P08243-2;P08243	0.00E+00	0.00E+00	0.00E+00	0.00E+00	0.00E+00	0.00E+00	0.00E+00	0.00E+00	0.00E+00	0.00E+00	0.00E+00	0.00E+00	0.00E+00	0.00E+00	0.00E+00	1.81E+06	1.13E+05	0.00E+00	2.27E+05	4.54E+05	0.00E+00	6.41E+05	3.34E-01
P08473	0.00E+00	0.00E+00	0.00E+00	0.00E+00	0.00E+00	8.50E+07	0.00E+00	0.00E+00	0.00E+00	0.00E+00	0.00E+00	0.00E+00	0.00E+00	0.00E+00	0.00E+00	0.00E+00	5.31E+06	1.06E+07	0.00E+00	2.13E+07	3.01E+07	0.00E+00	3.34E-01
P08754	0.00E+00	0.00E+00	0.00E+00	0.00E+00	0.00E+00	0.00E+00	0.00E+00	0.00E+00	0.00E+00	0.00E+00	0.00E+00	0.00E+00	0.00E+00	0.00E+00	0.00E+00	2.90E+06	1.81E+05	0.00E+00	3.63E+05	7.25E+05	0.00E+00	1.03E+06	3.34E-01
P09104-2;P09104;F5H0C8	0.00E+00	0.00E+00	0.00E+00	0.00E+00	0.00E+00	0.00E+00	0.00E+00	0.00E+00	0.00E+00	0.00E+00	0.00E+00	0.00E+00	0.00E+00	0.00E+00	5.30E+06	0.00E+00	3.32E+05	0.00E+00	6.63E+05	1.33E+06	0.00E+00	1.88E+06	3.34E-01
Q5T7C4;Q5T7C6;P09429	0.00E+00	0.00E+00	0.00E+00	0.00E+00	0.00E+00	0.00E+00	0.00E+00	0.00E+00	0.00E+00	0.00E+00	0.00E+00	0.00E+00	3.37E+07	0.00E+00	0.00E+00	0.00E+00	2.11E+06	0.00E+00	4.21E+06	8.42E+06	0.00E+00	1.19E+07	3.34E-01
P09471-2;P09471;H3BQG8;H3BTM2;H3BN82	0.00E+00	0.00E+00	0.00E+00	0.00E+00	0.00E+00	0.00E+00	8.28E+06	0.00E+00	0.00E+00	0.00E+00	0.00E+00	0.00E+00	0.00E+00	0.00E+00	0.00E+00	0.00E+00	5.18E+05	1.04E+06	0.00E+00	2.07E+06	2.93E+06	0.00E+00	3.34E-01
P09525;Q6P452;P09525-2	0.00E+00	0.00E+00	0.00E+00	0.00E+00	0.00E+00	0.00E+00	0.00E+00	0.00E+00	1.63E+06	0.00E+00	0.00E+00	0.00E+00	0.00E+00	0.00E+00	0.00E+00	0.00E+00	1.02E+05	0.00E+00	2.04E+05	4.09E+05	0.00E+00	5.78E+05	3.34E-01
P09972;K7EKH5;C9J8F3;J3KSV6;J3QKP5;A8MVZ9	0.00E+00	0.00E+00	2.69E+06	0.00E+00	0.00E+00	0.00E+00	0.00E+00	0.00E+00	0.00E+00	0.00E+00	0.00E+00	0.00E+00	0.00E+00	0.00E+00	0.00E+00	0.00E+00	1.68E+05	3.36E+05	0.00E+00	6.71E+05	9.50E+05	0.00E+00	3.34E-01
P10155-2;P10155-3;P10155-5;P10155-4;P10155	0.00E+00	0.00E+00	0.00E+00	0.00E+00	0.00E+00	0.00E+00	0.00E+00	0.00E+00	0.00E+00	0.00E+00	0.00E+00	0.00E+00	9.25E+05	0.00E+00	0.00E+00	0.00E+00	5.78E+04	0.00E+00	1.16E+05	2.31E+05	0.00E+00	3.27E+05	3.34E-01
P10253	0.00E+00	0.00E+00	0.00E+00	0.00E+00	0.00E+00	4.06E+07	0.00E+00	0.00E+00	0.00E+00	0.00E+00	0.00E+00	0.00E+00	0.00E+00	0.00E+00	0.00E+00	0.00E+00	2.54E+06	5.08E+06	0.00E+00	1.02E+07	1.44E+07	0.00E+00	3.34E-01
P10301	0.00E+00	0.00E+00	0.00E+00	0.00E+00	0.00E+00	0.00E+00	0.00E+00	0.00E+00	0.00E+00	0.00E+00	0.00E+00	0.00E+00	2.86E+06	0.00E+00	0.00E+00	0.00E+00	1.79E+05	0.00E+00	3.58E+05	7.16E+05	0.00E+00	1.01E+06	3.34E-01
P10523	0.00E+00	0.00E+00	0.00E+00	0.00E+00	0.00E+00	0.00E+00	0.00E+00	0.00E+00	0.00E+00	0.00E+00	0.00E+00	0.00E+00	0.00E+00	0.00E+00	0.00E+00	1.97E+06	1.23E+05	0.00E+00	2.46E+05	4.93E+05	0.00E+00	6.97E+05	3.34E-01
P11172-2;P11172-3;P11172	0.00E+00	0.00E+00	0.00E+00	0.00E+00	0.00E+00	0.00E+00	8.51E+06	0.00E+00	0.00E+00	0.00E+00	0.00E+00	0.00E+00	0.00E+00	0.00E+00	0.00E+00	0.00E+00	5.32E+05	1.06E+06	0.00E+00	2.13E+06	3.01E+06	0.00E+00	3.34E-01
P11233;H7C3P7	0.00E+00	0.00E+00	0.00E+00	0.00E+00	6.77E+06	0.00E+00	0.00E+00	0.00E+00	0.00E+00	0.00E+00	0.00E+00	0.00E+00	0.00E+00	0.00E+00	0.00E+00	0.00E+00	4.23E+05	8.46E+05	0.00E+00	1.69E+06	2.39E+06	0.00E+00	3.34E-01
P11279	0.00E+00	0.00E+00	0.00E+00	0.00E+00	0.00E+00	0.00E+00	0.00E+00	0.00E+00	0.00E+00	0.00E+00	0.00E+00	0.00E+00	0.00E+00	0.00E+00	6.66E+07	0.00E+00	4.16E+06	0.00E+00	8.33E+06	1.67E+07	0.00E+00	2.36E+07	3.34E-01
P11413;P11413-2;E7EM57;E7EUI8;P11413-3;E9PD92	0.00E+00	0.00E+00	0.00E+00	0.00E+00	0.00E+00	0.00E+00	0.00E+00	0.00E+00	0.00E+00	0.00E+00	0.00E+00	0.00E+00	0.00E+00	0.00E+00	3.42E+06	0.00E+00	2.13E+05	0.00E+00	4.27E+05	8.54E+05	0.00E+00	1.21E+06	3.34E-01
P11488;C9JCV8;P19087;A8MTJ3	0.00E+00	0.00E+00	0.00E+00	0.00E+00	0.00E+00	4.23E+06	0.00E+00	0.00E+00	0.00E+00	0.00E+00	0.00E+00	0.00E+00	0.00E+00	0.00E+00	0.00E+00	0.00E+00	2.64E+05	5.29E+05	0.00E+00	1.06E+06	1.50E+06	0.00E+00	3.34E-01
P11498;E9PRE7;P11498-2	0.00E+00	0.00E+00	0.00E+00	0.00E+00	0.00E+00	0.00E+00	0.00E+00	0.00E+00	0.00E+00	0.00E+00	0.00E+00	0.00E+00	0.00E+00	6.98E+07	0.00E+00	0.00E+00	4.36E+06	0.00E+00	8.72E+06	1.74E+07	0.00E+00	2.47E+07	3.34E-01
P11717	0.00E+00	0.00E+00	0.00E+00	0.00E+00	0.00E+00	0.00E+00	1.55E+07	0.00E+00	0.00E+00	0.00E+00	0.00E+00	0.00E+00	0.00E+00	0.00E+00	0.00E+00	0.00E+00	9.68E+05	1.94E+06	0.00E+00	3.87E+06	5.48E+06	0.00E+00	3.34E-01
P12277;H0YJG0	0.00E+00	0.00E+00	0.00E+00	0.00E+00	0.00E+00	0.00E+00	0.00E+00	0.00E+00	0.00E+00	0.00E+00	0.00E+00	0.00E+00	1.03E+07	0.00E+00	0.00E+00	0.00E+00	6.45E+05	0.00E+00	1.29E+06	2.58E+06	0.00E+00	3.65E+06	3.34E-01
P13473-2;P13473;P13473-3	0.00E+00	0.00E+00	0.00E+00	0.00E+00	0.00E+00	0.00E+00	0.00E+00	0.00E+00	0.00E+00	3.56E+05	0.00E+00	0.00E+00	0.00E+00	0.00E+00	0.00E+00	0.00E+00	2.22E+04	0.00E+00	4.45E+04	8.90E+04	0.00E+00	1.26E+05	3.34E-01
P13693;Q5W0H4;J3KPG2;E9PJF7	0.00E+00	0.00E+00	0.00E+00	0.00E+00	0.00E+00	2.22E+06	0.00E+00	0.00E+00	0.00E+00	0.00E+00	0.00E+00	0.00E+00	0.00E+00	0.00E+00	0.00E+00	0.00E+00	1.39E+05	2.77E+05	0.00E+00	5.54E+05	7.84E+05	0.00E+00	3.34E-01
P13861-2;P13861;C9J830;H7C1L0	0.00E+00	0.00E+00	0.00E+00	0.00E+00	0.00E+00	0.00E+00	0.00E+00	0.00E+00	0.00E+00	0.00E+00	0.00E+00	0.00E+00	0.00E+00	0.00E+00	0.00E+00	6.62E+05	4.14E+04	0.00E+00	8.28E+04	1.66E+05	0.00E+00	2.34E+05	3.34E-01
P14136-3;P14136;P14136-2;K7ELP4;K7EJU1;K7EKH9	0.00E+00	0.00E+00	0.00E+00	0.00E+00	0.00E+00	0.00E+00	0.00E+00	0.00E+00	0.00E+00	0.00E+00	0.00E+00	0.00E+00	0.00E+00	0.00E+00	0.00E+00	2.46E+05	1.54E+04	0.00E+00	3.08E+04	6.15E+04	0.00E+00	8.70E+04	3.34E-01

P15121;E9PCX2	0.00E+00	0.00E+00	0.00E+00	0.00E+00	0.00E+00	0.00E+00	0.00E+00	0.00E+00	0.00E+00	0.00E+00	0.00E+00	0.00E+00	0.00E+00	0.00E+00	0.00E+00	1.17E+06	7.32E+04	0.00E+00	1.46E+05	2.93E+05	0.00E+00	4.14E+05	3.34E-01
P17066;P48741	0.00E+00	0.00E+00	0.00E+00	0.00E+00	0.00E+00	2.55E+07	0.00E+00	0.00E+00	0.00E+00	0.00E+00	0.00E+00	0.00E+00	0.00E+00	0.00E+00	0.00E+00	0.00E+00	1.59E+06	3.18E+06	0.00E+00	6.36E+06	9.00E+06	0.00E+00	3.34E-01
P17655-2;P17655	0.00E+00	0.00E+00	0.00E+00	0.00E+00	0.00E+00	0.00E+00	0.00E+00	0.00E+00	0.00E+00	0.00E+00	0.00E+00	3.59E+05	0.00E+00	0.00E+00	0.00E+00	0.00E+00	2.24E+04	0.00E+00	4.49E+04	8.98E+04	0.00E+00	1.27E+05	3.34E-01
P19013	0.00E+00	0.00E+00	0.00E+00	0.00E+00	0.00E+00	0.00E+00	1.91E+05	0.00E+00	0.00E+00	0.00E+00	0.00E+00	0.00E+00	0.00E+00	0.00E+00	0.00E+00	0.00E+00	1.19E+04	2.39E+04	0.00E+00	4.77E+04	6.75E+04	0.00E+00	3.34E-01
P20042	0.00E+00	0.00E+00	0.00E+00	0.00E+00	0.00E+00	0.00E+00	2.19E+06	0.00E+00	0.00E+00	0.00E+00	0.00E+00	0.00E+00	0.00E+00	0.00E+00	0.00E+00	0.00E+00	1.37E+05	2.74E+05	0.00E+00	5.49E+05	7.76E+05	0.00E+00	3.34E-01
P20339-2;P20339	0.00E+00	0.00E+00	0.00E+00	0.00E+00	0.00E+00	0.00E+00	0.00E+00	0.00E+00	0.00E+00	0.00E+00	0.00E+00	2.60E+05	0.00E+00	0.00E+00	0.00E+00	0.00E+00	1.62E+04	0.00E+00	3.25E+04	6.49E+04	0.00E+00	9.18E+04	3.34E-01
P21266;A0A0A0MTN3	0.00E+00	0.00E+00	0.00E+00	0.00E+00	0.00E+00	0.00E+00	0.00E+00	0.00E+00	0.00E+00	0.00E+00	0.00E+00	0.00E+00	0.00E+00	1.49E+06	0.00E+00	0.00E+00	9.28E+04	0.00E+00	1.86E+05	3.71E+05	0.00E+00	5.25E+05	3.34E-01
P21953;P21953-2	0.00E+00	0.00E+00	0.00E+00	0.00E+00	0.00E+00	0.00E+00	3.16E+06	0.00E+00	0.00E+00	0.00E+00	0.00E+00	0.00E+00	0.00E+00	0.00E+00	0.00E+00	0.00E+00	1.98E+05	3.95E+05	0.00E+00	7.91E+05	1.12E+06	0.00E+00	3.34E-01
P22033	0.00E+00	0.00E+00	0.00E+00	0.00E+00	0.00E+00	0.00E+00	0.00E+00	0.00E+00	0.00E+00	0.00E+00	0.00E+00	0.00E+00	0.00E+00	0.00E+00	8.03E+06	0.00E+00	5.02E+05	0.00E+00	1.00E+06	2.01E+06	0.00E+00	2.84E+06	3.34E-01
P28070	0.00E+00	0.00E+00	0.00E+00	0.00E+00	0.00E+00	0.00E+00	0.00E+00	0.00E+00	0.00E+00	0.00E+00	0.00E+00	0.00E+00	0.00E+00	0.00E+00	0.00E+00	3.67E+05	2.29E+04	0.00E+00	4.58E+04	9.17E+04	0.00E+00	1.30E+05	3.34E-01
P28072;A0A087X2I4	0.00E+00	0.00E+00	0.00E+00	0.00E+00	0.00E+00	0.00E+00	0.00E+00	0.00E+00	0.00E+00	0.00E+00	4.15E+06	0.00E+00	0.00E+00	0.00E+00	0.00E+00	0.00E+00	2.60E+05	0.00E+00	5.19E+05	1.04E+06	0.00E+00	1.47E+06	3.34E-01
P28288-2;P28288	0.00E+00	0.00E+00	0.00E+00	0.00E+00	0.00E+00	0.00E+00	0.00E+00	1.85E+05	0.00E+00	0.00E+00	0.00E+00	0.00E+00	0.00E+00	0.00E+00	0.00E+00	0.00E+00	1.15E+04	2.31E+04	0.00E+00	4.61E+04	6.52E+04	0.00E+00	3.34E-01
P29317	0.00E+00	0.00E+00	0.00E+00	0.00E+00	0.00E+00	0.00E+00	0.00E+00	0.00E+00	0.00E+00	0.00E+00	2.98E+05	0.00E+00	0.00E+00	0.00E+00	0.00E+00	0.00E+00	1.86E+04	0.00E+00	3.73E+04	7.46E+04	0.00E+00	1.05E+05	3.34E-01
P29992;K7EL62;A0A087W VZ3	0.00E+00	0.00E+00	0.00E+00	0.00E+00	0.00E+00	0.00E+00	0.00E+00	0.00E+00	0.00E+00	0.00E+00	0.00E+00	0.00E+00	0.00E+00	0.00E+00	6.66E+05	0.00E+00	4.16E+04	0.00E+00	8.32E+04	1.66E+05	0.00E+00	2.35E+05	3.34E-01
P30038-2;P30038-3;P30038	0.00E+00	0.00E+00	0.00E+00	0.00E+00	0.00E+00	0.00E+00	0.00E+00	0.00E+00	0.00E+00	0.00E+00	0.00E+00	0.00E+00	0.00E+00	0.00E+00	0.00E+00	2.84E+06	1.78E+05	0.00E+00	3.55E+05	7.10E+05	0.00E+00	1.00E+06	3.34E-01
P30419-2;P30419;K7EN82	0.00E+00	0.00E+00	0.00E+00	0.00E+00	0.00E+00	0.00E+00	0.00E+00	0.00E+00	0.00E+00	0.00E+00	0.00E+00	0.00E+00	2.76E+06	0.00E+00	0.00E+00	0.00E+00	1.73E+05	0.00E+00	3.45E+05	6.90E+05	0.00E+00	9.76E+05	3.34E-01
Q29940;P30493;P30492;P30480;P30479;P30466;P30462;P30460;P18463;P30510;P30504;P30685;P30498;P30495;P30491;P30490;P30484;P30464;P18465;P18464;P10319;Q29865	0.00E+00	0.00E+00	0.00E+00	0.00E+00	2.76E+06	0.00E+00	0.00E+00	0.00E+00	0.00E+00	0.00E+00	0.00E+00	0.00E+00	0.00E+00	0.00E+00	0.00E+00	0.00E+00	1.73E+05	3.45E+05	0.00E+00	6.90E+05	9.76E+05	0.00E+00	3.34E-01
P30520	0.00E+00	0.00E+00	0.00E+00	0.00E+00	0.00E+00	0.00E+00	0.00E+00	0.00E+00	0.00E+00	0.00E+00	0.00E+00	0.00E+00	1.31E+06	0.00E+00	0.00E+00	0.00E+00	8.16E+04	0.00E+00	1.63E+05	3.26E+05	0.00E+00	4.61E+05	3.34E-01
P30740-2;P30740	0.00E+00	0.00E+00	0.00E+00	0.00E+00	8.55E+05	0.00E+00	0.00E+00	0.00E+00	0.00E+00	0.00E+00	0.00E+00	0.00E+00	0.00E+00	0.00E+00	0.00E+00	0.00E+00	5.34E+04	1.07E+05	0.00E+00	2.14E+05	3.02E+05	0.00E+00	3.34E-01
P30837	0.00E+00	0.00E+00	0.00E+00	0.00E+00	0.00E+00	0.00E+00	3.11E+05	0.00E+00	0.00E+00	0.00E+00	0.00E+00	0.00E+00	0.00E+00	0.00E+00	0.00E+00	0.00E+00	1.94E+04	3.89E+04	0.00E+00	7.78E+04	1.10E+05	0.00E+00	3.34E-01
Q5VSP4;P31025	0.00E+00	0.00E+00	0.00E+00	0.00E+00	0.00E+00	2.72E+06	0.00E+00	0.00E+00	0.00E+00	0.00E+00	0.00E+00	0.00E+00	0.00E+00	0.00E+00	0.00E+00	0.00E+00	1.70E+05	3.39E+05	0.00E+00	6.79E+05	9.60E+05	0.00E+00	3.34E-01
P31153-2;P31153	0.00E+00	0.00E+00	0.00E+00	0.00E+00	0.00E+00	6.18E+06	0.00E+00	0.00E+00	0.00E+00	0.00E+00	0.00E+00	0.00E+00	0.00E+00	0.00E+00	0.00E+00	0.00E+00	3.86E+05	7.72E+05	0.00E+00	1.54E+06	2.18E+06	0.00E+00	3.34E-01
P31937	0.00E+00	0.00E+00	0.00E+00	0.00E+00	0.00E+00	0.00E+00	0.00E+00	0.00E+00	0.00E+00	0.00E+00	0.00E+00	0.00E+00	9.56E+05	0.00E+00	0.00E+00	0.00E+00	5.98E+04	0.00E+00	1.20E+05	2.39E+05	0.00E+00	3.38E+05	3.34E-01
P31939-2;P31939;H7C1S2	0.00E+00	0.00E+00	0.00E+00	0.00E+00	0.00E+00	0.00E+00	0.00E+00	0.00E+00	0.00E+00	0.00E+00	0.00E+00	0.00E+00	0.00E+00	0.00E+00	0.00E+00	3.11E+05	1.95E+04	0.00E+00	3.89E+04	7.78E+04	0.00E+00	1.10E+05	3.34E-01
P31946-2;P31946	0.00E+00	0.00E+00	3.12E+06	0.00E+00	0.00E+00	0.00E+00	0.00E+00	0.00E+00	0.00E+00	0.00E+00	0.00E+00	0.00E+00	0.00E+00	0.00E+00	0.00E+00	0.00E+00	1.95E+05	3.90E+05	0.00E+00	7.80E+05	1.10E+06	0.00E+00	3.34E-01
P31947-2;P31947	0.00E+00	0.00E+00	1.03E+06	0.00E+00	0.00E+00	0.00E+00	0.00E+00	0.00E+00	0.00E+00	0.00E+00	0.00E+00	0.00E+00	0.00E+00	0.00E+00	0.00E+00	0.00E+00	6.44E+04	1.29E+05	0.00E+00	2.58E+05	3.64E+05	0.00E+00	3.34E-01
P34931	0.00E+00	0.00E+00	0.00E+00	0.00E+00	0.00E+00	0.00E+00	3.24E+05	0.00E+00	0.00E+00	0.00E+00	0.00E+00	0.00E+00	0.00E+00	0.00E+00	0.00E+00	0.00E+00	2.02E+04	4.04E+04	0.00E+00	8.09E+04	1.14E+05	0.00E+00	3.34E-01
P35241;P35241-5;E9PNV3;E9PQ82	1.19E+06	0.00E+00	0.00E+00	0.00E+00	0.00E+00	0.00E+00	0.00E+00	0.00E+00	0.00E+00	0.00E+00	0.00E+00	0.00E+00	0.00E+00	0.00E+00	0.00E+00	0.00E+00	7.46E+04	1.49E+05	0.00E+00	2.98E+05	4.22E+05	0.00E+00	3.34E-01
P35250-2;P35250;H7C5S7;H7C5P1;H7C5Q7;F8WC37;H7C5A0;H7C5G4;A0A087WVY3	0.00E+00	0.00E+00	0.00E+00	0.00E+00	0.00E+00	0.00E+00	0.00E+00	0.00E+00	0.00E+00	0.00E+00	0.00E+00	0.00E+00	0.00E+00	0.00E+00	0.00E+00	1.41E+06	8.84E+04	0.00E+00	1.77E+05	3.53E+05	0.00E+00	5.00E+05	3.34E-01
P35270	0.00E+00	0.00E+00	0.00E+00	0.00E+00	0.00E+00	0.00E+00	0.00E+00	0.00E+00	0.00E+00	0.00E+00	0.00E+00	0.00E+00	0.00E+00	0.00E+00	5.32E+06	0.00E+00	3.33E+05	0.00E+00	6.65E+05	1.33E+06	0.00E+00	1.88E+06	3.34E-01
P36551;H0YA22	0.00E+00	0.00E+00	0.00E+00	0.00E+00	0.00E+00	0.00E+00	0.00E+00	0.00E+00	0.00E+00	4.17E+06	0.00E+00	0.00E+00	0.00E+00	0.00E+00	0.00E+00	0.00E+00	2.61E+05	0.00E+00	5.22E+05	1.04E+06	0.00E+00	1.48E+06	3.34E-01
P38606-2;P38606	0.00E+00	0.00E+00	0.00E+00	0.00E+00	0.00E+00	0.00E+00	0.00E+00	0.00E+00	0.00E+00	0.00E+00	0.00E+00	0.00E+00	2.08E+05	0.00E+00	0.00E+00	0.00E+00	1.30E+04	0.00E+00	2.60E+04	5.21E+04	0.00E+00	7.36E+04	3.34E-01
P38919	0.00E+00	0.00E+00	0.00E+00	0.00E+00	0.00E+00	0.00E+00	0.00E+00	0.00E+00	0.00E+00	2.10E+06	0.00E+00	0.00E+00	0.00E+00	0.00E+00	0.00E+00	0.00E+00	1.32E+05	0.00E+00	2.63E+05	5.26E+05	0.00E+00	7.44E+05	3.34E-01
P40222	0.00E+00	0.00E+00	1.67E+06	0.00E+00	0.00E+00	0.00E+00	0.00E+00	0.00E+00	0.00E+00	0.00E+00	0.00E+00	0.00E+00	0.00E+00	0.00E+00	0.00E+00	0.00E+00	1.04E+05	2.09E+05	0.00E+00	4.17E+05	5.90E+05	0.00E+00	3.34E-01

P41091;H7BZU1;Q2VIR3-2;F8W810;Q2VIR3	0.00E+00	0.00E+00	0.00E+00	0.00E+00	0.00E+00	0.00E+00	0.00E+00	0.00E+00	0.00E+00	0.00E+00	0.00E+00	0.00E+00	0.00E+00	0.00E+00	0.00E+00	2.73E+05	1.70E+04	0.00E+00	3.41E+04	6.82E+04	0.00E+00	9.64E+04	3.34E-01
P42224;E7ENM1;E7EPD2;D2KFR9;P42224-2;J3KPM9	0.00E+00	0.00E+00	0.00E+00	0.00E+00	4.49E+06	0.00E+00	0.00E+00	0.00E+00	0.00E+00	0.00E+00	0.00E+00	0.00E+00	0.00E+00	0.00E+00	0.00E+00	0.00E+00	2.80E+05	5.61E+05	0.00E+00	1.12E+06	1.59E+06	0.00E+00	3.34E-01
P48449-2;P48449-3;P48449	0.00E+00	0.00E+00	0.00E+00	0.00E+00	0.00E+00	0.00E+00	0.00E+00	0.00E+00	0.00E+00	0.00E+00	0.00E+00	0.00E+00	0.00E+00	0.00E+00	0.00E+00	3.42E+05	2.14E+04	0.00E+00	4.28E+04	8.56E+04	0.00E+00	1.21E+05	3.34E-01
P48637-2;P48637	0.00E+00	0.00E+00	0.00E+00	0.00E+00	0.00E+00	0.00E+00	0.00E+00	0.00E+00	0.00E+00	0.00E+00	0.00E+00	0.00E+00	0.00E+00	2.84E+06	0.00E+00	0.00E+00	1.77E+05	0.00E+00	3.55E+05	7.10E+05	0.00E+00	1.00E+06	3.34E-01
P48960-2;P48960-3;P48960	0.00E+00	0.00E+00	0.00E+00	0.00E+00	0.00E+00	0.00E+00	0.00E+00	0.00E+00	0.00E+00	0.00E+00	0.00E+00	0.00E+00	0.00E+00	0.00E+00	0.00E+00	9.41E+06	5.88E+05	0.00E+00	1.18E+06	2.35E+06	0.00E+00	3.33E+06	3.34E-01
P49792	0.00E+00	0.00E+00	0.00E+00	0.00E+00	0.00E+00	0.00E+00	2.39E+06	0.00E+00	0.00E+00	0.00E+00	0.00E+00	0.00E+00	0.00E+00	0.00E+00	0.00E+00	0.00E+00	1.50E+05	2.99E+05	0.00E+00	5.98E+05	8.46E+05	0.00E+00	3.34E-01
P50402;Q5HY57	0.00E+00	0.00E+00	0.00E+00	0.00E+00	0.00E+00	0.00E+00	0.00E+00	3.79E+06	0.00E+00	0.00E+00	0.00E+00	0.00E+00	0.00E+00	0.00E+00	0.00E+00	0.00E+00	2.37E+05	4.74E+05	0.00E+00	9.48E+05	1.34E+06	0.00E+00	3.34E-01
P50453	0.00E+00	0.00E+00	0.00E+00	0.00E+00	0.00E+00	0.00E+00	0.00E+00	0.00E+00	0.00E+00	0.00E+00	0.00E+00	0.00E+00	0.00E+00	0.00E+00	0.00E+00	2.22E+06	1.39E+05	0.00E+00	2.78E+05	5.56E+05	0.00E+00	7.87E+05	3.34E-01
P51151;Q9NP90	0.00E+00	0.00E+00	0.00E+00	0.00E+00	0.00E+00	0.00E+00	0.00E+00	0.00E+00	0.00E+00	2.08E+06	0.00E+00	0.00E+00	0.00E+00	0.00E+00	0.00E+00	0.00E+00	1.30E+05	0.00E+00	2.61E+05	5.21E+05	0.00E+00	7.37E+05	3.34E-01
P51153	0.00E+00	0.00E+00	0.00E+00	0.00E+00	0.00E+00	0.00E+00	0.00E+00	0.00E+00	0.00E+00	0.00E+00	0.00E+00	0.00E+00	0.00E+00	0.00E+00	3.90E+06	0.00E+00	2.44E+05	0.00E+00	4.87E+05	9.75E+05	0.00E+00	1.38E+06	3.34E-01
P51659;E7ER27;E7EWE5;P51659-3;P51659-2	0.00E+00	0.00E+00	0.00E+00	0.00E+00	0.00E+00	0.00E+00	1.00E+06	0.00E+00	0.00E+00	0.00E+00	0.00E+00	0.00E+00	0.00E+00	0.00E+00	0.00E+00	0.00E+00	6.25E+04	1.25E+05	0.00E+00	2.50E+05	3.53E+05	0.00E+00	3.34E-01
P52292	0.00E+00	0.00E+00	0.00E+00	0.00E+00	0.00E+00	0.00E+00	0.00E+00	0.00E+00	0.00E+00	0.00E+00	0.00E+00	0.00E+00	2.02E+05	0.00E+00	0.00E+00	0.00E+00	1.26E+04	0.00E+00	2.52E+04	5.05E+04	0.00E+00	7.14E+04	3.34E-01
P52701-4;P52701-3;P52701;A0A087WYT6;A0A087WWJ1;P52701-2	0.00E+00	0.00E+00	8.48E+05	0.00E+00	0.00E+00	0.00E+00	0.00E+00	0.00E+00	0.00E+00	0.00E+00	0.00E+00	0.00E+00	0.00E+00	0.00E+00	0.00E+00	0.00E+00	5.30E+04	1.06E+05	0.00E+00	2.12E+05	3.00E+05	0.00E+00	3.34E-01
P52732	0.00E+00	0.00E+00	0.00E+00	0.00E+00	0.00E+00	0.00E+00	0.00E+00	0.00E+00	0.00E+00	0.00E+00	0.00E+00	0.00E+00	0.00E+00	0.00E+00	0.00E+00	1.47E+06	9.22E+04	0.00E+00	1.84E+05	3.69E+05	0.00E+00	5.21E+05	3.34E-01
P52788;P52788-2;H7C2R7	0.00E+00	0.00E+00	0.00E+00	0.00E+00	0.00E+00	0.00E+00	0.00E+00	0.00E+00	0.00E+00	0.00E+00	0.00E+00	8.08E+05	0.00E+00	0.00E+00	0.00E+00	0.00E+00	5.05E+04	0.00E+00	1.01E+05	2.02E+05	0.00E+00	2.86E+05	3.34E-01
P53701	0.00E+00	0.00E+00	0.00E+00	0.00E+00	0.00E+00	0.00E+00	0.00E+00	0.00E+00	0.00E+00	0.00E+00	0.00E+00	0.00E+00	0.00E+00	0.00E+00	6.96E+05	0.00E+00	4.35E+04	0.00E+00	8.70E+04	1.74E+05	0.00E+00	2.46E+05	3.34E-01
P55957;P55957-2	0.00E+00	0.00E+00	0.00E+00	0.00E+00	0.00E+00	0.00E+00	0.00E+00	0.00E+00	0.00E+00	0.00E+00	0.00E+00	0.00E+00	0.00E+00	0.00E+00	0.00E+00	9.17E+05	5.73E+04	0.00E+00	1.15E+05	2.29E+05	0.00E+00	3.24E+05	3.34E-01
P56192;H0YHL6;H0YI94;H0YI27;H0YIP0;H0YHV5;P56192-2	0.00E+00	0.00E+00	0.00E+00	0.00E+00	0.00E+00	0.00E+00	2.19E+06	0.00E+00	0.00E+00	0.00E+00	0.00E+00	0.00E+00	0.00E+00	0.00E+00	0.00E+00	0.00E+00	1.37E+05	2.74E+05	0.00E+00	5.48E+05	7.75E+05	0.00E+00	3.34E-01
P56545;Q5SQP8;P56545-2	0.00E+00	0.00E+00	0.00E+00	0.00E+00	0.00E+00	0.00E+00	0.00E+00	0.00E+00	0.00E+00	0.00E+00	0.00E+00	0.00E+00	2.73E+06	0.00E+00	0.00E+00	0.00E+00	1.71E+05	0.00E+00	3.41E+05	6.83E+05	0.00E+00	9.65E+05	3.34E-01
P60953;Q5JYX0;P60953-1	0.00E+00	0.00E+00	0.00E+00	0.00E+00	0.00E+00	0.00E+00	0.00E+00	0.00E+00	0.00E+00	0.00E+00	0.00E+00	0.00E+00	4.42E+06	0.00E+00	0.00E+00	0.00E+00	2.76E+05	0.00E+00	5.52E+05	1.10E+06	0.00E+00	1.56E+06	3.34E-01
P61009	0.00E+00	0.00E+00	0.00E+00	0.00E+00	0.00E+00	0.00E+00	0.00E+00	0.00E+00	0.00E+00	0.00E+00	0.00E+00	0.00E+00	0.00E+00	5.33E+06	0.00E+00	0.00E+00	3.33E+05	0.00E+00	6.66E+05	1.33E+06	0.00E+00	1.88E+06	3.34E-01
P61026	0.00E+00	0.00E+00	0.00E+00	0.00E+00	0.00E+00	0.00E+00	0.00E+00	0.00E+00	0.00E+00	3.46E+06	0.00E+00	0.00E+00	0.00E+00	0.00E+00	0.00E+00	0.00E+00	2.16E+05	0.00E+00	4.32E+05	8.65E+05	0.00E+00	1.22E+06	3.34E-01
P61158;B4DXW1	0.00E+00	0.00E+00	0.00E+00	0.00E+00	0.00E+00	0.00E+00	0.00E+00	0.00E+00	0.00E+00	0.00E+00	0.00E+00	0.00E+00	0.00E+00	0.00E+00	2.50E+05	0.00E+00	1.56E+04	0.00E+00	3.13E+04	6.26E+04	0.00E+00	8.85E+04	3.34E-01
R4GMT0;P61163;P42025	0.00E+00	0.00E+00	0.00E+00	0.00E+00	0.00E+00	0.00E+00	0.00E+00	0.00E+00	0.00E+00	0.00E+00	0.00E+00	0.00E+00	0.00E+00	0.00E+00	1.34E+07	0.00E+00	8.39E+05	0.00E+00	1.68E+06	3.35E+06	0.00E+00	4.74E+06	3.34E-01
P61266-2;P61266;H3BT82	0.00E+00	0.00E+00	0.00E+00	0.00E+00	0.00E+00	0.00E+00	0.00E+00	1.24E+06	0.00E+00	0.00E+00	0.00E+00	0.00E+00	0.00E+00	0.00E+00	0.00E+00	0.00E+00	7.77E+04	1.55E+05	0.00E+00	3.11E+05	4.40E+05	0.00E+00	3.34E-01
P61764;P61764-2	0.00E+00	0.00E+00	0.00E+00	0.00E+00	0.00E+00	0.00E+00	0.00E+00	0.00E+00	0.00E+00	0.00E+00	0.00E+00	0.00E+00	0.00E+00	0.00E+00	5.71E+05	0.00E+00	3.57E+04	0.00E+00	7.14E+04	1.43E+05	0.00E+00	2.02E+05	3.34E-01
P61964	0.00E+00	0.00E+00	0.00E+00	0.00E+00	2.77E+07	0.00E+00	0.00E+00	0.00E+00	0.00E+00	0.00E+00	0.00E+00	0.00E+00	0.00E+00	0.00E+00	0.00E+00	0.00E+00	1.73E+06	3.47E+06	0.00E+00	6.93E+06	9.80E+06	0.00E+00	3.34E-01
P67775;P62714;H0YBN9;H0YC23;P67775-2	0.00E+00	0.00E+00	0.00E+00	0.00E+00	0.00E+00	0.00E+00	5.53E+06	0.00E+00	0.00E+00	0.00E+00	0.00E+00	0.00E+00	0.00E+00	0.00E+00	0.00E+00	0.00E+00	3.45E+05	6.91E+05	0.00E+00	1.38E+06	1.95E+06	0.00E+00	3.34E-01
P62805	0.00E+00	0.00E+00	0.00E+00	0.00E+00	0.00E+00	0.00E+00	0.00E+00	0.00E+00	0.00E+00	0.00E+00	0.00E+00	0.00E+00	2.51E+06	0.00E+00	0.00E+00	0.00E+00	1.57E+05	0.00E+00	3.14E+05	6.29E+05	0.00E+00	8.89E+05	3.34E-01
P62820;P62820-2;E7END7	0.00E+00	0.00E+00	0.00E+00	0.00E+00	0.00E+00	0.00E+00	0.00E+00	0.00E+00	0.00E+00	0.00E+00	0.00E+00	0.00E+00	0.00E+00	0.00E+00	0.00E+00	4.01E+05	2.51E+04	0.00E+00	5.01E+04	1.00E+05	0.00E+00	1.42E+05	3.34E-01
P62993-2;P62993	0.00E+00	0.00E+00	0.00E+00	0.00E+00	0.00E+00	0.00E+00	0.00E+00	0.00E+00	0.00E+00	0.00E+00	0.00E+00	0.00E+00	0.00E+00	1.80E+07	0.00E+00	0.00E+00	1.13E+06	0.00E+00	2.25E+06	4.51E+06	0.00E+00	6.37E+06	3.34E-01
P63092-3;P63092-2;P63092;P63092-4;Q5JWF2-2;Q5JWF2;A0A0A0MR13;P38405;P38405-2	0.00E+00	0.00E+00	0.00E+00	0.00E+00	0.00E+00	0.00E+00	0.00E+00	0.00E+00	0.00E+00	0.00E+00	0.00E+00	0.00E+00	0.00E+00	2.08E+08	0.00E+00	0.00E+00	1.30E+07	0.00E+00	2.60E+07	5.21E+07	0.00E+00	7.36E+07	3.34E-01
P63261;I3L3I0;I3L1U9;I3L4N8	0.00E+00	0.00E+00	0.00E+00	0.00E+00	0.00E+00	0.00E+00	0.00E+00	0.00E+00	0.00E+00	0.00E+00	0.00E+00	0.00E+00	0.00E+00	0.00E+00	0.00E+00	2.25E+07	1.41E+06	0.00E+00	2.81E+06	5.62E+06	0.00E+00	7.95E+06	3.34E-01

P68133;P68032;P63267;P62736;Q5T8M8;Q5T8M7;A6NL76;F8WB63;B8ZZJ2;C9JFL5;F6UVQ4;F6QUT6;P63267-2	0.00E+00	0.00E+00	0.00E+00	0.00E+00	0.00E+00	7.14E+06	0.00E+00	0.00E+00	0.00E+00	0.00E+00	0.00E+00	0.00E+00	0.00E+00	0.00E+00	0.00E+00	0.00E+00	4.46E+05	8.92E+05	0.00E+00	1.78E+06	2.52E+06	0.00E+00	3.34E-01
P68363;P68363-2	0.00E+00	0.00E+00	0.00E+00	0.00E+00	0.00E+00	0.00E+00	0.00E+00	0.00E+00	0.00E+00	0.00E+00	0.00E+00	0.00E+00	0.00E+00	0.00E+00	0.00E+00	1.37E+06	8.55E+04	0.00E+00	1.71E+05	3.42E+05	0.00E+00	4.84E+05	3.34E-01
P68366-2;P68366	0.00E+00	0.00E+00	0.00E+00	0.00E+00	0.00E+00	0.00E+00	7.59E+07	0.00E+00	0.00E+00	0.00E+00	0.00E+00	0.00E+00	0.00E+00	0.00E+00	0.00E+00	0.00E+00	4.75E+06	9.49E+06	0.00E+00	1.90E+07	2.69E+07	0.00E+00	3.34E-01
P68402;P68402-3;J3KNE3;P68402-4	0.00E+00	0.00E+00	0.00E+00	0.00E+00	0.00E+00	0.00E+00	0.00E+00	0.00E+00	0.00E+00	0.00E+00	3.06E+07	0.00E+00	0.00E+00	0.00E+00	0.00E+00	0.00E+00	1.91E+06	0.00E+00	3.83E+06	7.66E+06	0.00E+00	1.08E+07	3.34E-01
P78385	0.00E+00	0.00E+00	0.00E+00	0.00E+00	0.00E+00	0.00E+00	0.00E+00	0.00E+00	0.00E+00	0.00E+00	0.00E+00	0.00E+00	0.00E+00	0.00E+00	5.89E+05	0.00E+00	3.68E+04	0.00E+00	7.36E+04	1.47E+05	0.00E+00	2.08E+05	3.34E-01
P81605;P81605-2	0.00E+00	0.00E+00	0.00E+00	0.00E+00	0.00E+00	0.00E+00	0.00E+00	0.00E+00	0.00E+00	0.00E+00	0.00E+00	0.00E+00	5.42E+05	0.00E+00	0.00E+00	0.00E+00	3.39E+04	0.00E+00	6.77E+04	1.35E+05	0.00E+00	1.92E+05	3.34E-01
P82673	0.00E+00	0.00E+00	0.00E+00	0.00E+00	0.00E+00	0.00E+00	0.00E+00	0.00E+00	0.00E+00	0.00E+00	0.00E+00	0.00E+00	6.76E+05	0.00E+00	0.00E+00	0.00E+00	4.23E+04	0.00E+00	8.45E+04	1.69E+05	0.00E+00	2.39E+05	3.34E-01
P82914	0.00E+00	0.00E+00	6.19E+06	0.00E+00	0.00E+00	0.00E+00	0.00E+00	0.00E+00	0.00E+00	0.00E+00	0.00E+00	0.00E+00	0.00E+00	0.00E+00	0.00E+00	0.00E+00	3.87E+05	7.74E+05	0.00E+00	1.55E+06	2.19E+06	0.00E+00	3.34E-01
P82933	0.00E+00	0.00E+00	0.00E+00	0.00E+00	0.00E+00	0.00E+00	0.00E+00	0.00E+00	0.00E+00	0.00E+00	0.00E+00	0.00E+00	0.00E+00	0.00E+00	0.00E+00	9.88E+05	6.18E+04	0.00E+00	1.24E+05	2.47E+05	0.00E+00	3.49E+05	3.34E-01
Q01813-2;Q01813;Q5VSR5;V9GYV7	0.00E+00	0.00E+00	0.00E+00	0.00E+00	0.00E+00	0.00E+00	0.00E+00	0.00E+00	0.00E+00	0.00E+00	0.00E+00	0.00E+00	0.00E+00	0.00E+00	0.00E+00	2.02E+06	1.26E+05	0.00E+00	2.53E+05	5.05E+05	0.00E+00	7.15E+05	3.34E-01
Q01970-2;Q01970	0.00E+00	0.00E+00	0.00E+00	0.00E+00	0.00E+00	0.00E+00	0.00E+00	0.00E+00	0.00E+00	0.00E+00	0.00E+00	0.00E+00	0.00E+00	0.00E+00	0.00E+00	4.54E+06	2.83E+05	0.00E+00	5.67E+05	1.13E+06	0.00E+00	1.60E+06	3.34E-01
Q02790;F5H1U3	0.00E+00	0.00E+00	0.00E+00	0.00E+00	0.00E+00	4.74E+06	0.00E+00	0.00E+00	0.00E+00	0.00E+00	0.00E+00	0.00E+00	0.00E+00	0.00E+00	0.00E+00	0.00E+00	2.96E+05	5.92E+05	0.00E+00	1.18E+06	1.68E+06	0.00E+00	3.34E-01
Q02809;Q02809-2	0.00E+00	0.00E+00	0.00E+00	0.00E+00	0.00E+00	0.00E+00	0.00E+00	0.00E+00	0.00E+00	0.00E+00	0.00E+00	2.59E+06	0.00E+00	0.00E+00	0.00E+00	0.00E+00	1.62E+05	0.00E+00	3.24E+05	6.48E+05	0.00E+00	9.17E+05	3.34E-01
Q04446;E9PGM4	0.00E+00	0.00E+00	0.00E+00	0.00E+00	0.00E+00	0.00E+00	0.00E+00	0.00E+00	0.00E+00	0.00E+00	0.00E+00	0.00E+00	0.00E+00	0.00E+00	0.00E+00	2.15E+06	1.35E+05	0.00E+00	2.69E+05	5.39E+05	0.00E+00	7.62E+05	3.34E-01
Q04917;A2IDB2	0.00E+00	0.00E+00	0.00E+00	0.00E+00	0.00E+00	0.00E+00	0.00E+00	0.00E+00	0.00E+00	0.00E+00	0.00E+00	0.00E+00	0.00E+00	2.70E+06	0.00E+00	0.00E+00	1.69E+05	0.00E+00	3.38E+05	6.75E+05	0.00E+00	9.55E+05	3.34E-01
Q08379-2;Q08379;A0A087WYC0;B7ZC06;Q9NVV4-2	0.00E+00	0.00E+00	0.00E+00	0.00E+00	0.00E+00	0.00E+00	0.00E+00	0.00E+00	0.00E+00	0.00E+00	0.00E+00	0.00E+00	2.70E+06	0.00E+00	0.00E+00	0.00E+00	1.69E+05	0.00E+00	3.38E+05	6.76E+05	0.00E+00	9.56E+05	3.34E-01
Q08554-2;Q08554	6.04E+06	0.00E+00	0.00E+00	0.00E+00	0.00E+00	0.00E+00	0.00E+00	0.00E+00	0.00E+00	0.00E+00	0.00E+00	0.00E+00	0.00E+00	0.00E+00	0.00E+00	0.00E+00	3.77E+05	7.55E+05	0.00E+00	1.51E+06	2.13E+06	0.00E+00	3.34E-01
Q08J23-3;Q08J23-2;Q08J23	0.00E+00	0.00E+00	0.00E+00	0.00E+00	0.00E+00	0.00E+00	0.00E+00	0.00E+00	0.00E+00	0.00E+00	0.00E+00	0.00E+00	0.00E+00	0.00E+00	0.00E+00	1.20E+06	7.51E+04	0.00E+00	1.50E+05	3.00E+05	0.00E+00	4.25E+05	3.34E-01
Q09161	0.00E+00	0.00E+00	0.00E+00	0.00E+00	0.00E+00	0.00E+00	0.00E+00	0.00E+00	0.00E+00	0.00E+00	0.00E+00	0.00E+00	0.00E+00	0.00E+00	0.00E+00	1.02E+06	6.36E+04	0.00E+00	1.27E+05	2.54E+05	0.00E+00	3.60E+05	3.34E-01
Q10713	0.00E+00	0.00E+00	0.00E+00	0.00E+00	0.00E+00	0.00E+00	0.00E+00	0.00E+00	0.00E+00	0.00E+00	0.00E+00	0.00E+00	0.00E+00	2.61E+06	0.00E+00	0.00E+00	1.63E+05	0.00E+00	3.27E+05	6.54E+05	0.00E+00	9.24E+05	3.34E-01
Q12792;F8VRG3;Q12792-4;F8VS81;Q12792-3	0.00E+00	0.00E+00	0.00E+00	0.00E+00	0.00E+00	0.00E+00	0.00E+00	0.00E+00	0.00E+00	2.44E+06	0.00E+00	0.00E+00	0.00E+00	0.00E+00	0.00E+00	0.00E+00	1.52E+05	0.00E+00	3.05E+05	6.10E+05	0.00E+00	8.62E+05	3.34E-01
Q12846-2;Q12846	0.00E+00	0.00E+00	0.00E+00	0.00E+00	0.00E+00	0.00E+00	1.92E+06	0.00E+00	0.00E+00	0.00E+00	0.00E+00	0.00E+00	0.00E+00	0.00E+00	0.00E+00	0.00E+00	1.20E+05	2.40E+05	0.00E+00	4.79E+05	6.77E+05	0.00E+00	3.34E-01
Q12874	0.00E+00	0.00E+00	0.00E+00	0.00E+00	1.20E+07	0.00E+00	0.00E+00	0.00E+00	0.00E+00	0.00E+00	0.00E+00	0.00E+00	0.00E+00	0.00E+00	0.00E+00	0.00E+00	7.51E+05	1.50E+06	0.00E+00	3.00E+06	4.25E+06	0.00E+00	3.34E-01
Q12904;Q12904-2	0.00E+00	0.00E+00	0.00E+00	0.00E+00	0.00E+00	0.00E+00	0.00E+00	0.00E+00	0.00E+00	0.00E+00	0.00E+00	0.00E+00	0.00E+00	7.77E+06	0.00E+00	0.00E+00	4.86E+05	0.00E+00	9.71E+05	1.94E+06	0.00E+00	2.75E+06	3.34E-01
Q13045-2;Q13045;Q13045-3	0.00E+00	0.00E+00	0.00E+00	0.00E+00	0.00E+00	0.00E+00	0.00E+00	0.00E+00	0.00E+00	0.00E+00	0.00E+00	0.00E+00	0.00E+00	0.00E+00	3.23E+06	0.00E+00	2.02E+05	0.00E+00	4.04E+05	8.07E+05	0.00E+00	1.14E+06	3.34E-01
Q13148;A0A087WX29;B1AKP7;G3V162;A0A087X260;A0A087WYY0;A0A087WXQ5;K7EJM5;A0A087WV68;K7EN94;Q13148-4	0.00E+00	0.00E+00	0.00E+00	0.00E+00	0.00E+00	0.00E+00	0.00E+00	0.00E+00	0.00E+00	0.00E+00	0.00E+00	0.00E+00	0.00E+00	0.00E+00	1.07E+07	0.00E+00	6.67E+05	0.00E+00	1.33E+06	2.67E+06	0.00E+00	3.78E+06	3.34E-01
Q13217;X6R9L0	0.00E+00	0.00E+00	0.00E+00	0.00E+00	0.00E+00	0.00E+00	2.18E+06	0.00E+00	0.00E+00	0.00E+00	0.00E+00	0.00E+00	0.00E+00	0.00E+00	0.00E+00	0.00E+00	1.36E+05	2.73E+05	0.00E+00	5.46E+05	7.72E+05	0.00E+00	3.34E-01
Q13243-3;Q13243	0.00E+00	0.00E+00	0.00E+00	0.00E+00	0.00E+00	0.00E+00	0.00E+00	0.00E+00	0.00E+00	0.00E+00	0.00E+00	0.00E+00	0.00E+00	0.00E+00	0.00E+00	2.57E+06	1.61E+05	0.00E+00	3.22E+05	6.43E+05	0.00E+00	9.10E+05	3.34E-01
Q13247-3;Q13247	0.00E+00	0.00E+00	0.00E+00	0.00E+00	0.00E+00	0.00E+00	0.00E+00	0.00E+00	0.00E+00	0.00E+00	0.00E+00	0.00E+00	3.43E+06	0.00E+00	0.00E+00	0.00E+00	2.15E+05	0.00E+00	4.29E+05	8.58E+05	0.00E+00	1.21E+06	3.34E-01
Q13257-2;Q13257	0.00E+00	0.00E+00	0.00E+00	0.00E+00	0.00E+00	0.00E+00	2.14E+06	0.00E+00	0.00E+00	0.00E+00	0.00E+00	0.00E+00	0.00E+00	0.00E+00	0.00E+00	0.00E+00	1.34E+05	2.67E+05	0.00E+00	5.35E+05	7.56E+05	0.00E+00	3.34E-01
Q13283	0.00E+00	0.00E+00	0.00E+00	0.00E+00	0.00E+00	0.00E+00	0.00E+00	0.00E+00	0.00E+00	0.00E+00	0.00E+00	0.00E+00	0.00E+00	0.00E+00	0.00E+00	1.03E+06	6.41E+04	0.00E+00	1.28E+05	2.56E+05	0.00E+00	3.62E+05	3.34E-01
Q13347;Q5TFK1	0.00E+00	0.00E+00	0.00E+00	0.00E+00	0.00E+00	1.06E+06	0.00E+00	0.00E+00	0.00E+00	0.00E+00	0.00E+00	0.00E+00	0.00E+00	0.00E+00	0.00E+00	0.00E+00	6.63E+04	1.33E+05	0.00E+00	2.65E+05	3.75E+05	0.00E+00	3.34E-01
Q13442	0.00E+00	0.00E+00	0.00E+00	0.00E+00	0.00E+00	0.00E+00	0.00E+00	0.00E+00	0.00E+00	0.00E+00	0.00E+00	0.00E+00	0.00E+00	0.00E+00	0.00E+00	5.66E+06	3.54E+05	0.00E+00	7.07E+05	1.41E+06	0.00E+00	2.00E+06	3.34E-01

Q13492-4;Q13492-3;Q13492-2;Q13492-5;Q13492	0.00E+00	0.00E+00	3.01E+06	0.00E+00	0.00E+00	0.00E+00	0.00E+00	0.00E+00	0.00E+00	0.00E+00	0.00E+00	0.00E+00	0.00E+00	0.00E+00	0.00E+00	0.00E+00	1.88E+05	3.76E+05	0.00E+00	7.53E+05	1.06E+06	0.00E+00	3.34E-01
Q13509;Q13509-2	0.00E+00	0.00E+00	0.00E+00	0.00E+00	0.00E+00	0.00E+00	0.00E+00	0.00E+00	0.00E+00	0.00E+00	6.71E+05	0.00E+00	0.00E+00	0.00E+00	0.00E+00	0.00E+00	4.20E+04	0.00E+00	8.39E+04	1.68E+05	0.00E+00	2.37E+05	3.34E-01
Q5TEE2;Q92769-3;Q13547;Q92769	0.00E+00	0.00E+00	0.00E+00	0.00E+00	0.00E+00	0.00E+00	0.00E+00	0.00E+00	0.00E+00	0.00E+00	0.00E+00	0.00E+00	0.00E+00	0.00E+00	0.00E+00	9.47E+05	5.92E+04	0.00E+00	1.18E+05	2.37E+05	0.00E+00	3.35E+05	3.34E-01
Q13616	0.00E+00	0.00E+00	0.00E+00	0.00E+00	0.00E+00	7.13E+07	0.00E+00	0.00E+00	0.00E+00	0.00E+00	0.00E+00	0.00E+00	0.00E+00	0.00E+00	0.00E+00	0.00E+00	4.46E+06	8.92E+06	0.00E+00	1.78E+07	2.52E+07	0.00E+00	3.34E-01
Q13637	0.00E+00	0.00E+00	0.00E+00	0.00E+00	0.00E+00	0.00E+00	1.10E+06	0.00E+00	0.00E+00	0.00E+00	0.00E+00	0.00E+00	0.00E+00	0.00E+00	0.00E+00	0.00E+00	6.85E+04	1.37E+05	0.00E+00	2.74E+05	3.87E+05	0.00E+00	3.34E-01
Q13885;Q9BVA1	0.00E+00	0.00E+00	0.00E+00	0.00E+00	0.00E+00	0.00E+00	0.00E+00	0.00E+00	0.00E+00	0.00E+00	0.00E+00	0.00E+00	0.00E+00	0.00E+00	0.00E+00	3.61E+06	2.26E+05	0.00E+00	4.52E+05	9.03E+05	0.00E+00	1.28E+06	3.34E-01
Q13907;Q13907-2;C9JD53;C9JKM8	0.00E+00	0.00E+00	0.00E+00	0.00E+00	0.00E+00	0.00E+00	0.00E+00	0.00E+00	2.59E+06	0.00E+00	0.00E+00	0.00E+00	0.00E+00	0.00E+00	0.00E+00	0.00E+00	1.62E+05	0.00E+00	3.24E+05	6.47E+05	0.00E+00	9.16E+05	3.34E-01
Q14151;Q15424-2;Q15424;Q15424-4;Q15424-3	0.00E+00	0.00E+00	0.00E+00	0.00E+00	1.60E+06	0.00E+00	0.00E+00	0.00E+00	0.00E+00	0.00E+00	0.00E+00	0.00E+00	0.00E+00	0.00E+00	0.00E+00	0.00E+00	9.98E+04	2.00E+05	0.00E+00	3.99E+05	5.64E+05	0.00E+00	3.34E-01
Q14160-2;Q14160;Q14160-3;H0YCG0	0.00E+00	0.00E+00	0.00E+00	0.00E+00	0.00E+00	0.00E+00	0.00E+00	0.00E+00	0.00E+00	0.00E+00	7.21E+06	0.00E+00	0.00E+00	0.00E+00	0.00E+00	0.00E+00	4.51E+05	0.00E+00	9.02E+05	1.80E+06	0.00E+00	2.55E+06	3.34E-01
Q14554;H7C4F9;Q14554-2	0.00E+00	0.00E+00	0.00E+00	0.00E+00	0.00E+00	0.00E+00	0.00E+00	0.00E+00	0.00E+00	0.00E+00	0.00E+00	3.38E+06	0.00E+00	0.00E+00	0.00E+00	0.00E+00	2.11E+05	0.00E+00	4.22E+05	8.44E+05	0.00E+00	1.19E+06	3.34E-01
Q14739;C9JXK0	0.00E+00	0.00E+00	0.00E+00	0.00E+00	0.00E+00	0.00E+00	0.00E+00	0.00E+00	0.00E+00	1.72E+07	0.00E+00	0.00E+00	0.00E+00	0.00E+00	0.00E+00	0.00E+00	1.08E+06	0.00E+00	2.16E+06	4.31E+06	0.00E+00	6.10E+06	3.34E-01
Q14789-4;Q14789-3;Q14789;Q14789-2;H0Y867;E7EU81	0.00E+00	0.00E+00	0.00E+00	1.49E+06	0.00E+00	0.00E+00	0.00E+00	0.00E+00	0.00E+00	0.00E+00	0.00E+00	0.00E+00	0.00E+00	0.00E+00	0.00E+00	0.00E+00	9.29E+04	1.86E+05	0.00E+00	3.71E+05	5.25E+05	0.00E+00	3.34E-01
Q14997-2;Q14997;Q14997-3	0.00E+00	0.00E+00	0.00E+00	0.00E+00	0.00E+00	0.00E+00	0.00E+00	0.00E+00	0.00E+00	0.00E+00	0.00E+00	1.99E+06	0.00E+00	0.00E+00	0.00E+00	0.00E+00	1.24E+05	0.00E+00	2.49E+05	4.98E+05	0.00E+00	7.04E+05	3.34E-01
Q15008;Q15008-4;C9J7B7;Q15008-3;Q15008-2	0.00E+00	0.00E+00	0.00E+00	0.00E+00	0.00E+00	0.00E+00	0.00E+00	0.00E+00	0.00E+00	0.00E+00	0.00E+00	0.00E+00	0.00E+00	0.00E+00	1.28E+06	0.00E+00	8.00E+04	0.00E+00	1.60E+05	3.20E+05	0.00E+00	4.53E+05	3.34E-01
Q15020-4;Q15020;F8VV04	0.00E+00	0.00E+00	0.00E+00	0.00E+00	0.00E+00	0.00E+00	0.00E+00	0.00E+00	0.00E+00	0.00E+00	0.00E+00	0.00E+00	4.95E+06	0.00E+00	0.00E+00	0.00E+00	3.10E+05	0.00E+00	6.19E+05	1.24E+06	0.00E+00	1.75E+06	3.34E-01
Q15021	0.00E+00	0.00E+00	0.00E+00	0.00E+00	0.00E+00	0.00E+00	0.00E+00	0.00E+00	0.00E+00	0.00E+00	0.00E+00	0.00E+00	2.65E+05	0.00E+00	0.00E+00	0.00E+00	1.66E+04	0.00E+00	3.31E+04	6.63E+04	0.00E+00	9.38E+04	3.34E-01
Q15056-2;Q15056	0.00E+00	0.00E+00	0.00E+00	0.00E+00	0.00E+00	0.00E+00	0.00E+00	0.00E+00	7.28E+06	0.00E+00	0.00E+00	0.00E+00	0.00E+00	0.00E+00	0.00E+00	0.00E+00	4.55E+05	0.00E+00	9.11E+05	1.82E+06	0.00E+00	2.58E+06	3.34E-01
Q15404-2;Q15404	0.00E+00	0.00E+00	0.00E+00	0.00E+00	0.00E+00	0.00E+00	0.00E+00	0.00E+00	0.00E+00	0.00E+00	0.00E+00	0.00E+00	7.31E+06	0.00E+00	0.00E+00	0.00E+00	4.57E+05	0.00E+00	9.14E+05	1.83E+06	0.00E+00	2.58E+06	3.34E-01
Q15459;Q15459-2	0.00E+00	0.00E+00	0.00E+00	0.00E+00	0.00E+00	0.00E+00	0.00E+00	0.00E+00	0.00E+00	0.00E+00	0.00E+00	0.00E+00	5.85E+05	0.00E+00	0.00E+00	0.00E+00	3.66E+04	0.00E+00	7.32E+04	1.46E+05	0.00E+00	2.07E+05	3.34E-01
Q15691	0.00E+00	0.00E+00	0.00E+00	0.00E+00	0.00E+00	0.00E+00	0.00E+00	0.00E+00	0.00E+00	4.49E+06	0.00E+00	0.00E+00	0.00E+00	0.00E+00	0.00E+00	0.00E+00	2.81E+05	0.00E+00	5.61E+05	1.12E+06	0.00E+00	1.59E+06	3.34E-01
Q15785	0.00E+00	0.00E+00	0.00E+00	0.00E+00	0.00E+00	0.00E+00	0.00E+00	0.00E+00	0.00E+00	0.00E+00	0.00E+00	0.00E+00	0.00E+00	0.00E+00	2.12E+07	0.00E+00	1.32E+06	0.00E+00	2.65E+06	5.29E+06	0.00E+00	7.48E+06	3.34E-01
Q16531;F5GY55	0.00E+00	0.00E+00	0.00E+00	0.00E+00	0.00E+00	0.00E+00	0.00E+00	0.00E+00	0.00E+00	0.00E+00	8.15E+05	0.00E+00	0.00E+00	0.00E+00	0.00E+00	0.00E+00	5.10E+04	0.00E+00	1.02E+05	2.04E+05	0.00E+00	2.88E+05	3.34E-01
Q16555-2;Q16555	0.00E+00	0.00E+00	0.00E+00	0.00E+00	2.46E+06	0.00E+00	0.00E+00	0.00E+00	0.00E+00	0.00E+00	0.00E+00	0.00E+00	0.00E+00	0.00E+00	0.00E+00	0.00E+00	1.54E+05	3.08E+05	0.00E+00	6.16E+05	8.71E+05	0.00E+00	3.34E-01
Q16643;Q16643-3;D6RFI1;Q16643-2	4.56E+06	0.00E+00	0.00E+00	0.00E+00	0.00E+00	0.00E+00	0.00E+00	0.00E+00	0.00E+00	0.00E+00	0.00E+00	0.00E+00	0.00E+00	0.00E+00	0.00E+00	0.00E+00	2.85E+05	5.70E+05	0.00E+00	1.14E+06	1.61E+06	0.00E+00	3.34E-01
Q16650	0.00E+00	0.00E+00	0.00E+00	0.00E+00	0.00E+00	0.00E+00	0.00E+00	0.00E+00	0.00E+00	0.00E+00	0.00E+00	0.00E+00	2.23E+06	0.00E+00	0.00E+00	0.00E+00	1.39E+05	0.00E+00	2.78E+05	5.57E+05	0.00E+00	7.87E+05	3.34E-01
Q16706	0.00E+00	0.00E+00	0.00E+00	0.00E+00	0.00E+00	0.00E+00	0.00E+00	0.00E+00	0.00E+00	0.00E+00	0.00E+00	0.00E+00	0.00E+00	1.27E+06	0.00E+00	0.00E+00	7.91E+04	0.00E+00	1.58E+05	3.17E+05	0.00E+00	4.48E+05	3.34E-01
Q27J81-2;Q27J81;A0A0A0MQU1	0.00E+00	0.00E+00	0.00E+00	0.00E+00	0.00E+00	0.00E+00	0.00E+00	0.00E+00	0.00E+00	0.00E+00	0.00E+00	0.00E+00	0.00E+00	0.00E+00	2.30E+07	0.00E+00	1.44E+06	0.00E+00	2.88E+06	5.75E+06	0.00E+00	8.14E+06	3.34E-01
Q29RF7	0.00E+00	0.00E+00	0.00E+00	0.00E+00	0.00E+00	0.00E+00	0.00E+00	0.00E+00	0.00E+00	0.00E+00	0.00E+00	0.00E+00	0.00E+00	2.67E+07	0.00E+00	0.00E+00	1.67E+06	0.00E+00	3.34E+06	6.69E+06	0.00E+00	9.46E+06	3.34E-01
Q3ZCM7;A0A075B736;Q5SQY0	0.00E+00	0.00E+00	0.00E+00	0.00E+00	0.00E+00	0.00E+00	1.57E+06	0.00E+00	0.00E+00	0.00E+00	0.00E+00	0.00E+00	0.00E+00	0.00E+00	0.00E+00	0.00E+00	9.79E+04	1.96E+05	0.00E+00	3.92E+05	5.54E+05	0.00E+00	3.34E-01
Q499Z4-2	0.00E+00	0.00E+00	0.00E+00	0.00E+00	0.00E+00	0.00E+00	0.00E+00	0.00E+00	0.00E+00	0.00E+00	0.00E+00	0.00E+00	0.00E+00	8.30E+08	0.00E+00	0.00E+00	5.19E+07	0.00E+00	1.04E+08	2.07E+08	0.00E+00	2.93E+08	3.34E-01
Q8IUC0;Q52LG2	1.68E+07	0.00E+00	0.00E+00	0.00E+00	0.00E+00	0.00E+00	0.00E+00	0.00E+00	0.00E+00	0.00E+00	0.00E+00	0.00E+00	0.00E+00	0.00E+00	0.00E+00	0.00E+00	1.05E+06	2.10E+06	0.00E+00	4.21E+06	5.95E+06	0.00E+00	3.34E-01

Q562R1	0.00E+00	0.00E+00	0.00E+00	0.00E+00	0.00E+00	0.00E+00	5.18E+07	0.00E+00	0.00E+00	0.00E+00	0.00E+00	0.00E+00	0.00E+00	0.00E+00	0.00E+00	0.00E+00	3.24E+06	6.48E+06	0.00E+00	1.30E+07	1.83E+07	0.00E+00	3.34E-01
Q58FF6	0.00E+00	0.00E+00	0.00E+00	0.00E+00	0.00E+00	0.00E+00	0.00E+00	0.00E+00	0.00E+00	0.00E+00	0.00E+00	0.00E+00	0.00E+00	0.00E+00	5.55E+05	0.00E+00	3.47E+04	0.00E+00	6.94E+04	1.39E+05	0.00E+00	1.96E+05	3.34E-01
Q58FF8	4.82E+06	0.00E+00	0.00E+00	0.00E+00	0.00E+00	0.00E+00	0.00E+00	0.00E+00	0.00E+00	0.00E+00	0.00E+00	0.00E+00	0.00E+00	0.00E+00	0.00E+00	0.00E+00	3.01E+05	6.02E+05	0.00E+00	1.20E+06	1.70E+06	0.00E+00	3.34E-01
Q5C9Z4	0.00E+00	0.00E+00	0.00E+00	0.00E+00	0.00E+00	0.00E+00	0.00E+00	0.00E+00	0.00E+00	0.00E+00	0.00E+00	0.00E+00	0.00E+00	0.00E+00	0.00E+00	4.55E+05	2.84E+04	0.00E+00	5.69E+04	1.14E+05	0.00E+00	1.61E+05	3.34E-01
Q5JTH9-2;Q5JTH9-3;Q5JTH9	0.00E+00	0.00E+00	0.00E+00	0.00E+00	0.00E+00	0.00E+00	0.00E+00	0.00E+00	0.00E+00	0.00E+00	0.00E+00	0.00E+00	0.00E+00	0.00E+00	2.60E+06	0.00E+00	1.62E+05	0.00E+00	3.25E+05	6.49E+05	0.00E+00	9.18E+05	3.34E-01
Q5JVF3-3;Q5JVF3-2;Q5JVF3;Q5JVF3-4	0.00E+00	0.00E+00	0.00E+00	0.00E+00	0.00E+00	0.00E+00	0.00E+00	0.00E+00	0.00E+00	0.00E+00	0.00E+00	0.00E+00	2.34E+05	0.00E+00	0.00E+00	0.00E+00	1.46E+04	0.00E+00	2.92E+04	5.84E+04	0.00E+00	8.26E+04	3.34E-01
Q5QPM2;Q5QPM0;Q5QPM1;Q5QPL9;Q9UKM9-2;Q9UKM9	0.00E+00	0.00E+00	0.00E+00	0.00E+00	0.00E+00	0.00E+00	0.00E+00	0.00E+00	0.00E+00	0.00E+00	0.00E+00	0.00E+00	0.00E+00	9.85E+05	0.00E+00	0.00E+00	6.16E+04	0.00E+00	1.23E+05	2.46E+05	0.00E+00	3.48E+05	3.34E-01
Q5SRE5-2;Q5SRE5;H7C4K7	0.00E+00	0.00E+00	0.00E+00	0.00E+00	0.00E+00	0.00E+00	0.00E+00	0.00E+00	0.00E+00	0.00E+00	0.00E+00	0.00E+00	1.80E+05	0.00E+00	0.00E+00	0.00E+00	1.12E+04	0.00E+00	2.25E+04	4.49E+04	0.00E+00	6.36E+04	3.34E-01
X6RGJ2;Q5SSJ5;B0QZK4;Q5SWC8;Q5SSJ5-3;Q5SSJ5-2	0.00E+00	0.00E+00	0.00E+00	0.00E+00	0.00E+00	0.00E+00	0.00E+00	0.00E+00	0.00E+00	0.00E+00	4.34E+05	0.00E+00	0.00E+00	0.00E+00	0.00E+00	0.00E+00	2.71E+04	0.00E+00	5.42E+04	1.08E+05	0.00E+00	1.53E+05	3.34E-01
Q5T5E9;U3KQ50;Q5T5F2;Q9BW92-2;U3KQG0;Q9BW92	0.00E+00	0.00E+00	0.00E+00	0.00E+00	0.00E+00	6.20E+06	0.00E+00	0.00E+00	0.00E+00	0.00E+00	0.00E+00	0.00E+00	0.00E+00	0.00E+00	0.00E+00	0.00E+00	3.87E+05	7.75E+05	0.00E+00	1.55E+06	2.19E+06	0.00E+00	3.34E-01
Q5T9A4;Q5T9A4-3	0.00E+00	0.00E+00	0.00E+00	0.00E+00	0.00E+00	0.00E+00	0.00E+00	0.00E+00	0.00E+00	0.00E+00	0.00E+00	0.00E+00	0.00E+00	0.00E+00	2.62E+06	0.00E+00	1.63E+05	0.00E+00	3.27E+05	6.54E+05	0.00E+00	9.25E+05	3.34E-01
Q5TB53;Q9HD45	0.00E+00	0.00E+00	0.00E+00	0.00E+00	0.00E+00	0.00E+00	0.00E+00	0.00E+00	0.00E+00	0.00E+00	0.00E+00	0.00E+00	0.00E+00	6.96E+06	0.00E+00	0.00E+00	4.35E+05	0.00E+00	8.70E+05	1.74E+06	0.00E+00	2.46E+06	3.34E-01
Q5TBR0;Q5TBR1;Q9NR45	0.00E+00	0.00E+00	0.00E+00	0.00E+00	0.00E+00	0.00E+00	0.00E+00	0.00E+00	0.00E+00	0.00E+00	0.00E+00	0.00E+00	0.00E+00	1.73E+06	0.00E+00	0.00E+00	1.08E+05	0.00E+00	2.17E+05	4.33E+05	0.00E+00	6.12E+05	3.34E-01
Q6NUK1-2;Q6NUK1	0.00E+00	0.00E+00	0.00E+00	0.00E+00	0.00E+00	0.00E+00	0.00E+00	1.40E+06	0.00E+00	0.00E+00	0.00E+00	0.00E+00	0.00E+00	0.00E+00	0.00E+00	0.00E+00	8.77E+04	1.75E+05	0.00E+00	3.51E+05	4.96E+05	0.00E+00	3.34E-01
Q6NUQ4-2;Q6NUQ4;H7BZI0;H7C0H8;H7C085;H7C008	1.36E+05	0.00E+00	0.00E+00	0.00E+00	0.00E+00	0.00E+00	0.00E+00	0.00E+00	0.00E+00	0.00E+00	0.00E+00	0.00E+00	0.00E+00	0.00E+00	0.00E+00	0.00E+00	8.49E+03	1.70E+04	0.00E+00	3.40E+04	4.80E+04	0.00E+00	3.34E-01
Q6NVY1;A0A087WXI1;Q6NVY1-2	0.00E+00	0.00E+00	0.00E+00	0.00E+00	0.00E+00	0.00E+00	1.15E+06	0.00E+00	0.00E+00	0.00E+00	0.00E+00	0.00E+00	0.00E+00	0.00E+00	0.00E+00	0.00E+00	7.20E+04	1.44E+05	0.00E+00	2.88E+05	4.08E+05	0.00E+00	3.34E-01
Q6P4A8	0.00E+00	0.00E+00	0.00E+00	0.00E+00	0.00E+00	0.00E+00	0.00E+00	0.00E+00	0.00E+00	0.00E+00	1.86E+07	0.00E+00	0.00E+00	0.00E+00	0.00E+00	0.00E+00	1.16E+06	0.00E+00	2.33E+06	4.65E+06	0.00E+00	6.58E+06	3.34E-01
Q6P587;Q6P587-2;Q6P587-3	0.00E+00	0.00E+00	0.00E+00	0.00E+00	0.00E+00	0.00E+00	0.00E+00	0.00E+00	0.00E+00	0.00E+00	0.00E+00	0.00E+00	0.00E+00	1.34E+05	0.00E+00	0.00E+00	8.36E+03	0.00E+00	1.67E+04	3.34E+04	0.00E+00	4.73E+04	3.34E-01
Q6S8J3;P0CG38	0.00E+00	0.00E+00	0.00E+00	0.00E+00	0.00E+00	0.00E+00	0.00E+00	0.00E+00	0.00E+00	1.25E+05	0.00E+00	0.00E+00	0.00E+00	0.00E+00	0.00E+00	0.00E+00	7.83E+03	0.00E+00	1.57E+04	3.13E+04	0.00E+00	4.43E+04	3.34E-01
Q6YN16-2;Q6YN16	0.00E+00	0.00E+00	0.00E+00	0.00E+00	0.00E+00	3.39E+07	0.00E+00	0.00E+00	0.00E+00	0.00E+00	0.00E+00	0.00E+00	0.00E+00	0.00E+00	0.00E+00	0.00E+00	2.12E+06	4.24E+06	0.00E+00	8.47E+06	1.20E+07	0.00E+00	3.34E-01
Q70UQ0-2;Q70UQ0	0.00E+00	0.00E+00	0.00E+00	0.00E+00	0.00E+00	0.00E+00	0.00E+00	0.00E+00	0.00E+00	0.00E+00	0.00E+00	0.00E+00	0.00E+00	0.00E+00	0.00E+00	7.88E+05	4.93E+04	0.00E+00	9.85E+04	1.97E+05	0.00E+00	2.79E+05	3.34E-01
Q71U36-2;Q71U36;Q13748;Q13748-2;F8VQQ4;Q6PEY2	0.00E+00	2.45E+06	0.00E+00	0.00E+00	0.00E+00	0.00E+00	0.00E+00	0.00E+00	0.00E+00	0.00E+00	0.00E+00	0.00E+00	0.00E+00	0.00E+00	0.00E+00	0.00E+00	1.53E+05	3.07E+05	0.00E+00	6.14E+05	8.68E+05	0.00E+00	3.34E-01
Q7KZ85-3;Q7KZ85	0.00E+00	4.47E+06	0.00E+00	0.00E+00	0.00E+00	0.00E+00	0.00E+00	0.00E+00	0.00E+00	0.00E+00	0.00E+00	0.00E+00	0.00E+00	0.00E+00	0.00E+00	0.00E+00	2.79E+05	5.58E+05	0.00E+00	1.12E+06	1.58E+06	0.00E+00	3.34E-01
Q7L2H7;J3KNJ2;Q7L2H7-2;E9PRY0;H0YCQ8	0.00E+00	0.00E+00	0.00E+00	0.00E+00	0.00E+00	0.00E+00	0.00E+00	0.00E+00	0.00E+00	1.55E+05	0.00E+00	0.00E+00	0.00E+00	0.00E+00	0.00E+00	0.00E+00	9.71E+03	0.00E+00	1.94E+04	3.88E+04	0.00E+00	5.49E+04	3.34E-01
Q7L576;Q7L576-3;Q7L576-2	0.00E+00	0.00E+00	0.00E+00	0.00E+00	0.00E+00	0.00E+00	0.00E+00	0.00E+00	0.00E+00	0.00E+00	0.00E+00	0.00E+00	0.00E+00	0.00E+00	1.24E+07	0.00E+00	7.74E+05	0.00E+00	1.55E+06	3.09E+06	0.00E+00	4.38E+06	3.34E-01
Q7L8L6	0.00E+00	0.00E+00	0.00E+00	4.49E+07	0.00E+00	0.00E+00	0.00E+00	0.00E+00	0.00E+00	0.00E+00	0.00E+00	0.00E+00	0.00E+00	0.00E+00	0.00E+00	0.00E+00	2.81E+06	5.61E+06	0.00E+00	1.12E+07	1.59E+07	0.00E+00	3.34E-01
Q7Z406;Q7Z406-6;Q7Z406-2;M0QY43;Q7Z406-5;Q7Z406-4	0.00E+00	0.00E+00	0.00E+00	0.00E+00	0.00E+00	0.00E+00	0.00E+00	0.00E+00	0.00E+00	0.00E+00	7.91E+05	0.00E+00	0.00E+00	0.00E+00	0.00E+00	0.00E+00	4.94E+04	0.00E+00	9.89E+04	1.98E+05	0.00E+00	2.80E+05	3.34E-01
Q7Z5P9-2;Q7Z5P9	0.00E+00	0.00E+00	0.00E+00	0.00E+00	0.00E+00	0.00E+00	0.00E+00	0.00E+00	0.00E+00	0.00E+00	0.00E+00	0.00E+00	0.00E+00	0.00E+00	0.00E+00	1.74E+06	1.09E+05	0.00E+00	2.18E+05	4.36E+05	0.00E+00	6.17E+05	3.34E-01
Q7Z794	0.00E+00	0.00E+00	0.00E+00	0.00E+00	0.00E+00	0.00E+00	1.30E+06	0.00E+00	0.00E+00	0.00E+00	0.00E+00	0.00E+00	0.00E+00	0.00E+00	0.00E+00	0.00E+00	8.14E+04	1.63E+05	0.00E+00	3.26E+05	4.61E+05	0.00E+00	3.34E-01
Q7Z7H8;Q7Z7H8-2	0.00E+00	0.00E+00	0.00E+00	0.00E+00	0.00E+00	1.48E+06	0.00E+00	0.00E+00	0.00E+00	0.00E+00	0.00E+00	0.00E+00	0.00E+00	0.00E+00	0.00E+00	0.00E+00	9.23E+04	1.85E+05	0.00E+00	3.69E+05	5.22E+05	0.00E+00	3.34E-01
Q86TC9;Q86TC9-3;A0A087WX60;Q86TC9-2	0.00E+00	0.00E+00	0.00E+00	0.00E+00	0.00E+00	0.00E+00	0.00E+00	0.00E+00	3.16E+06	0.00E+00	0.00E+00	0.00E+00	0.00E+00	0.00E+00	0.00E+00	0.00E+00	1.97E+05	0.00E+00	3.95E+05	7.90E+05	0.00E+00	1.12E+06	3.34E-01

Q86U42-2;Q86U42;H0YJH9;B4DEH8;G3V4T2;Q92843-2	0.00E+00	0.00E+00	0.00E+00	0.00E+00	0.00E+00	0.00E+00	0.00E+00	0.00E+00	0.00E+00	2.31E+05	0.00E+00	0.00E+00	0.00E+00	0.00E+00	0.00E+00	0.00E+00	1.44E+04	0.00E+00	2.89E+04	5.77E+04	0.00E+00	8.16E+04	3.34E-01
Q86W42-3;Q86W42;Q86W42-2	7.72E+06	0.00E+00	0.00E+00	0.00E+00	0.00E+00	0.00E+00	0.00E+00	0.00E+00	0.00E+00	0.00E+00	0.00E+00	0.00E+00	0.00E+00	0.00E+00	0.00E+00	0.00E+00	4.83E+05	9.65E+05	0.00E+00	1.93E+06	2.73E+06	0.00E+00	3.34E-01
Q8IVS2	0.00E+00	0.00E+00	0.00E+00	0.00E+00	0.00E+00	0.00E+00	0.00E+00	0.00E+00	0.00E+00	0.00E+00	0.00E+00	0.00E+00	5.04E+05	0.00E+00	0.00E+00	0.00E+00	3.15E+04	0.00E+00	6.30E+04	1.26E+05	0.00E+00	1.78E+05	3.34E-01
Q8IY17-2;Q8IY17-3;Q8IY17;Q8IY17-4;Q8IY17-5	0.00E+00	0.00E+00	0.00E+00	0.00E+00	0.00E+00	0.00E+00	0.00E+00	0.00E+00	0.00E+00	0.00E+00	0.00E+00	0.00E+00	0.00E+00	0.00E+00	0.00E+00	1.26E+06	7.90E+04	0.00E+00	1.58E+05	3.16E+05	0.00E+00	4.47E+05	3.34E-01
Q8N0U8	0.00E+00	0.00E+00	0.00E+00	0.00E+00	0.00E+00	0.00E+00	0.00E+00	0.00E+00	0.00E+00	0.00E+00	0.00E+00	0.00E+00	0.00E+00	0.00E+00	0.00E+00	3.33E+05	2.08E+04	0.00E+00	4.17E+04	8.34E+04	0.00E+00	1.18E+05	3.34E-01
Q8N766-4;Q8N766-3;Q8N766-2;Q8N766	0.00E+00	0.00E+00	0.00E+00	0.00E+00	0.00E+00	0.00E+00	0.00E+00	0.00E+00	0.00E+00	0.00E+00	0.00E+00	0.00E+00	0.00E+00	0.00E+00	3.48E+05	0.00E+00	2.18E+04	0.00E+00	4.35E+04	8.71E+04	0.00E+00	1.23E+05	3.34E-01
Q8NBX0	0.00E+00	0.00E+00	0.00E+00	0.00E+00	0.00E+00	0.00E+00	0.00E+00	0.00E+00	0.00E+00	0.00E+00	0.00E+00	0.00E+00	0.00E+00	0.00E+00	0.00E+00	5.71E+05	3.57E+04	0.00E+00	7.14E+04	1.43E+05	0.00E+00	2.02E+05	3.34E-01
Q8NF37	0.00E+00	0.00E+00	0.00E+00	0.00E+00	0.00E+00	0.00E+00	0.00E+00	0.00E+00	0.00E+00	0.00E+00	0.00E+00	5.67E+05	0.00E+00	0.00E+00	0.00E+00	0.00E+00	3.54E+04	0.00E+00	7.08E+04	1.42E+05	0.00E+00	2.00E+05	3.34E-01
Q8NFQ8	0.00E+00	0.00E+00	0.00E+00	0.00E+00	0.00E+00	0.00E+00	0.00E+00	0.00E+00	0.00E+00	0.00E+00	0.00E+00	0.00E+00	3.70E+06	0.00E+00	0.00E+00	0.00E+00	2.31E+05	0.00E+00	4.62E+05	9.24E+05	0.00E+00	1.31E+06	3.34E-01
Q8TAE8	0.00E+00	0.00E+00	0.00E+00	0.00E+00	0.00E+00	0.00E+00	0.00E+00	0.00E+00	0.00E+00	0.00E+00	0.00E+00	0.00E+00	0.00E+00	0.00E+00	0.00E+00	4.43E+06	2.77E+05	0.00E+00	5.54E+05	1.11E+06	0.00E+00	1.57E+06	3.34E-01
Q8TC12-2;Q8TC12;H0YIZ8;G3V2G6;H0YJ46	6.30E+06	0.00E+00	0.00E+00	0.00E+00	0.00E+00	0.00E+00	0.00E+00	0.00E+00	0.00E+00	0.00E+00	0.00E+00	0.00E+00	0.00E+00	0.00E+00	0.00E+00	0.00E+00	3.94E+05	7.88E+05	0.00E+00	1.58E+06	2.23E+06	0.00E+00	3.34E-01
Q8TC76	0.00E+00	0.00E+00	0.00E+00	0.00E+00	0.00E+00	0.00E+00	0.00E+00	0.00E+00	0.00E+00	0.00E+00	0.00E+00	0.00E+00	2.08E+06	0.00E+00	0.00E+00	0.00E+00	1.30E+05	0.00E+00	2.59E+05	5.19E+05	0.00E+00	7.34E+05	3.34E-01
Q8TCS8	0.00E+00	0.00E+00	0.00E+00	0.00E+00	1.45E+06	0.00E+00	0.00E+00	0.00E+00	0.00E+00	0.00E+00	0.00E+00	0.00E+00	0.00E+00	0.00E+00	0.00E+00	0.00E+00	9.09E+04	1.82E+05	0.00E+00	3.64E+05	5.14E+05	0.00E+00	3.34E-01
Q8TED1;J3KNB5;E7ETY7	0.00E+00	0.00E+00	0.00E+00	0.00E+00	0.00E+00	0.00E+00	0.00E+00	0.00E+00	1.27E+05	0.00E+00	0.00E+00	0.00E+00	0.00E+00	0.00E+00	0.00E+00	0.00E+00	7.97E+03	0.00E+00	1.59E+04	3.19E+04	0.00E+00	4.51E+04	3.34E-01
Q8TEQ6	0.00E+00	0.00E+00	0.00E+00	0.00E+00	0.00E+00	0.00E+00	0.00E+00	4.09E+06	0.00E+00	0.00E+00	0.00E+00	0.00E+00	0.00E+00	0.00E+00	0.00E+00	0.00E+00	2.56E+05	5.11E+05	0.00E+00	1.02E+06	1.45E+06	0.00E+00	3.34E-01
Q8WVM8;Q8WVM8-2;Q8WVM8-3;J3KNG4	0.00E+00	0.00E+00	0.00E+00	0.00E+00	0.00E+00	0.00E+00	0.00E+00	0.00E+00	0.00E+00	0.00E+00	0.00E+00	0.00E+00	0.00E+00	0.00E+00	0.00E+00	1.22E+06	7.62E+04	0.00E+00	1.52E+05	3.05E+05	0.00E+00	4.31E+05	3.34E-01
Q8WWC4;H7C0V0	0.00E+00	0.00E+00	0.00E+00	0.00E+00	0.00E+00	0.00E+00	0.00E+00	0.00E+00	0.00E+00	0.00E+00	0.00E+00	0.00E+00	0.00E+00	6.34E+05	0.00E+00	0.00E+00	3.97E+04	0.00E+00	7.93E+04	1.59E+05	0.00E+00	2.24E+05	3.34E-01
Q8WWM7-6;Q8WWM7-8;Q8WWM7-5;Q8WWM7-4;Q8WWM7-9;Q8WWM7-2;Q8WWM7;Q8WWM7-3	0.00E+00	0.00E+00	0.00E+00	0.00E+00	0.00E+00	0.00E+00	0.00E+00	0.00E+00	0.00E+00	0.00E+00	0.00E+00	0.00E+00	0.00E+00	4.39E+05	0.00E+00	0.00E+00	2.74E+04	0.00E+00	5.48E+04	1.10E+05	0.00E+00	1.55E+05	3.34E-01
Q8WX92	0.00E+00	0.00E+00	0.00E+00	0.00E+00	0.00E+00	0.00E+00	0.00E+00	0.00E+00	0.00E+00	0.00E+00	8.49E+05	0.00E+00	0.00E+00	0.00E+00	0.00E+00	0.00E+00	5.31E+04	0.00E+00	1.06E+05	2.12E+05	0.00E+00	3.00E+05	3.34E-01
X6RDA4;Q8WXF1-2;Q8WXF1	0.00E+00	0.00E+00	0.00E+00	0.00E+00	2.07E+06	0.00E+00	0.00E+00	0.00E+00	0.00E+00	0.00E+00	0.00E+00	0.00E+00	0.00E+00	0.00E+00	0.00E+00	0.00E+00	1.29E+05	2.59E+05	0.00E+00	5.18E+05	7.33E+05	0.00E+00	3.34E-01
Q92621	0.00E+00	0.00E+00	0.00E+00	0.00E+00	8.97E+06	0.00E+00	0.00E+00	0.00E+00	0.00E+00	0.00E+00	0.00E+00	0.00E+00	0.00E+00	0.00E+00	0.00E+00	0.00E+00	5.61E+05	1.12E+06	0.00E+00	2.24E+06	3.17E+06	0.00E+00	3.34E-01
Q92878-3;Q92878;Q92878-2;E7EN38	0.00E+00	0.00E+00	0.00E+00	0.00E+00	0.00E+00	0.00E+00	0.00E+00	0.00E+00	0.00E+00	0.00E+00	0.00E+00	0.00E+00	0.00E+00	0.00E+00	0.00E+00	4.63E+05	2.89E+04	0.00E+00	5.78E+04	1.16E+05	0.00E+00	1.64E+05	3.34E-01
Q92900-2;Q92900	0.00E+00	0.00E+00	0.00E+00	0.00E+00	0.00E+00	0.00E+00	0.00E+00	0.00E+00	0.00E+00	0.00E+00	0.00E+00	4.05E+06	0.00E+00	0.00E+00	0.00E+00	0.00E+00	2.53E+05	0.00E+00	5.06E+05	1.01E+06	0.00E+00	1.43E+06	3.34E-01
Q969Z0;C9J7P5;H7C4R5;C9IZN7;Q969Z0-2	0.00E+00	0.00E+00	0.00E+00	0.00E+00	0.00E+00	0.00E+00	0.00E+00	0.00E+00	0.00E+00	0.00E+00	0.00E+00	2.57E+06	0.00E+00	0.00E+00	0.00E+00	0.00E+00	1.61E+05	0.00E+00	3.22E+05	6.44E+05	0.00E+00	9.10E+05	3.34E-01
Q96A35	0.00E+00	0.00E+00	0.00E+00	0.00E+00	0.00E+00	0.00E+00	0.00E+00	0.00E+00	0.00E+00	0.00E+00	0.00E+00	0.00E+00	1.12E+05	0.00E+00	0.00E+00	0.00E+00	7.03E+03	0.00E+00	1.41E+04	2.81E+04	0.00E+00	3.98E+04	3.34E-01
Q96AB3-3;Q96AB3;Q96AB3-2;K7ENV7;K7EKW4	0.00E+00	0.00E+00	0.00E+00	0.00E+00	0.00E+00	0.00E+00	0.00E+00	0.00E+00	0.00E+00	0.00E+00	0.00E+00	0.00E+00	0.00E+00	0.00E+00	0.00E+00	3.62E+05	2.26E+04	0.00E+00	4.52E+04	9.04E+04	0.00E+00	1.28E+05	3.34E-01
Q96B26	0.00E+00	0.00E+00	0.00E+00	4.09E+05	0.00E+00	0.00E+00	0.00E+00	0.00E+00	0.00E+00	0.00E+00	0.00E+00	0.00E+00	0.00E+00	0.00E+00	0.00E+00	0.00E+00	2.56E+04	5.11E+04	0.00E+00	1.02E+05	1.45E+05	0.00E+00	3.34E-01
Q96G03	0.00E+00	0.00E+00	0.00E+00	0.00E+00	0.00E+00	0.00E+00	0.00E+00	0.00E+00	0.00E+00	0.00E+00	0.00E+00	0.00E+00	0.00E+00	0.00E+00	4.43E+05	0.00E+00	2.77E+04	0.00E+00	5.54E+04	1.11E+05	0.00E+00	1.57E+05	3.34E-01
Q96GM8-2;Q96GM8	0.00E+00	0.00E+00	0.00E+00	0.00E+00	2.89E+06	0.00E+00	0.00E+00	0.00E+00	0.00E+00	0.00E+00	0.00E+00	0.00E+00	0.00E+00	0.00E+00	0.00E+00	0.00E+00	1.80E+05	3.61E+05	0.00E+00	7.22E+05	1.02E+06	0.00E+00	3.34E-01
Q96HY7;H7C1J3	0.00E+00	0.00E+00	0.00E+00	0.00E+00	0.00E+00	0.00E+00	0.00E+00	2.88E+06	0.00E+00	0.00E+00	0.00E+00	0.00E+00	0.00E+00	0.00E+00	0.00E+00	0.00E+00	1.80E+05	3.60E+05	0.00E+00	7.20E+05	1.02E+06	0.00E+00	3.34E-01
Q96LW1	0.00E+00	0.00E+00	0.00E+00	0.00E+00	0.00E+00	0.00E+00	0.00E+00	0.00E+00	0.00E+00	0.00E+00	0.00E+00	0.00E+00	0.00E+00	0.00E+00	6.52E+05	0.00E+00	4.07E+04	0.00E+00	8.15E+04	1.63E+05	0.00E+00	2.30E+05	3.34E-01
Q96P70	0.00E+00	0.00E+00	0.00E+00	0.00E+00	0.00E+00	0.00E+00	0.00E+00	0.00E+00	0.00E+00	0.00E+00	0.00E+00	0.00E+00	0.00E+00	0.00E+00	0.00E+00	4.09E+06	2.56E+05	0.00E+00	5.11E+05	1.02E+06	0.00E+00	1.45E+06	3.34E-01

Q96PU8-5;Q96PU8-9;Q96PU8-8;Q96PU8-6;Q96PU8-3;Q96PU8	0.00E+00	0.00E+00	0.00E+00	0.00E+00	0.00E+00	0.00E+00	0.00E+00	0.00E+00	0.00E+00	0.00E+00	0.00E+00	0.00E+00	0.00E+00	1.56E+06	0.00E+00	0.00E+00	9.77E+04	0.00E+00	1.95E+05	3.91E+05	0.00E+00	5.53E+05	3.34E-01
Q96QD8;F8VUY8;Q96QD8-2	0.00E+00	0.00E+00	0.00E+00	0.00E+00	4.49E+06	0.00E+00	0.00E+00	0.00E+00	0.00E+00	0.00E+00	0.00E+00	0.00E+00	0.00E+00	0.00E+00	0.00E+00	0.00E+00	2.81E+05	5.61E+05	0.00E+00	1.12E+06	1.59E+06	0.00E+00	3.34E-01
Q96S52-2;Q96S52	0.00E+00	0.00E+00	0.00E+00	0.00E+00	0.00E+00	0.00E+00	0.00E+00	3.46E+06	0.00E+00	0.00E+00	0.00E+00	0.00E+00	0.00E+00	0.00E+00	0.00E+00	0.00E+00	2.16E+05	4.32E+05	0.00E+00	8.64E+05	1.22E+06	0.00E+00	3.34E-01
Q96ST3	0.00E+00	0.00E+00	0.00E+00	0.00E+00	0.00E+00	0.00E+00	0.00E+00	0.00E+00	0.00E+00	0.00E+00	0.00E+00	0.00E+00	0.00E+00	0.00E+00	3.01E+05	0.00E+00	1.88E+04	0.00E+00	3.76E+04	7.53E+04	0.00E+00	1.06E+05	3.34E-01
Q99536;K7EJM4;K7ERT7;Q99536-2;Q99536-3	0.00E+00	0.00E+00	0.00E+00	0.00E+00	0.00E+00	7.24E+05	0.00E+00	0.00E+00	0.00E+00	0.00E+00	0.00E+00	0.00E+00	0.00E+00	0.00E+00	0.00E+00	0.00E+00	4.53E+04	9.05E+04	0.00E+00	1.81E+05	2.56E+05	0.00E+00	3.34E-01
Q99614	1.99E+07	0.00E+00	0.00E+00	0.00E+00	0.00E+00	0.00E+00	0.00E+00	0.00E+00	0.00E+00	0.00E+00	0.00E+00	0.00E+00	0.00E+00	0.00E+00	0.00E+00	0.00E+00	1.24E+06	2.48E+06	0.00E+00	4.97E+06	7.03E+06	0.00E+00	3.34E-01
Q99720-3;Q99720;Q99720-4;Q5T1J1;Q99720-5;Q99720-2	0.00E+00	0.00E+00	0.00E+00	0.00E+00	0.00E+00	0.00E+00	2.15E+06	0.00E+00	0.00E+00	0.00E+00	0.00E+00	0.00E+00	0.00E+00	0.00E+00	0.00E+00	0.00E+00	1.34E+05	2.69E+05	0.00E+00	5.38E+05	7.60E+05	0.00E+00	3.34E-01
Q99805	0.00E+00	0.00E+00	0.00E+00	0.00E+00	0.00E+00	0.00E+00	0.00E+00	0.00E+00	0.00E+00	0.00E+00	0.00E+00	0.00E+00	0.00E+00	1.57E+07	0.00E+00	0.00E+00	9.83E+05	0.00E+00	1.97E+06	3.93E+06	0.00E+00	5.56E+06	3.34E-01
Q9BPX3	0.00E+00	0.00E+00	0.00E+00	0.00E+00	0.00E+00	0.00E+00	0.00E+00	0.00E+00	0.00E+00	0.00E+00	0.00E+00	0.00E+00	0.00E+00	0.00E+00	0.00E+00	2.80E+06	1.75E+05	0.00E+00	3.50E+05	7.00E+05	0.00E+00	9.91E+05	3.34E-01
Q9BS26	0.00E+00	0.00E+00	0.00E+00	0.00E+00	0.00E+00	0.00E+00	3.25E+05	0.00E+00	0.00E+00	0.00E+00	0.00E+00	0.00E+00	0.00E+00	0.00E+00	0.00E+00	0.00E+00	2.03E+04	4.06E+04	0.00E+00	8.12E+04	1.15E+05	0.00E+00	3.34E-01
Q9BSH4	0.00E+00	0.00E+00	0.00E+00	0.00E+00	0.00E+00	0.00E+00	0.00E+00	0.00E+00	0.00E+00	0.00E+00	3.14E+05	0.00E+00	0.00E+00	0.00E+00	0.00E+00	0.00E+00	1.96E+04	0.00E+00	3.93E+04	7.85E+04	0.00E+00	1.11E+05	3.34E-01
Q9BVM2;Q5JQQ4	0.00E+00	0.00E+00	0.00E+00	0.00E+00	0.00E+00	0.00E+00	0.00E+00	0.00E+00	0.00E+00	0.00E+00	0.00E+00	0.00E+00	0.00E+00	2.40E+06	0.00E+00	0.00E+00	1.50E+05	0.00E+00	3.00E+05	6.00E+05	0.00E+00	8.49E+05	3.34E-01
Q9BVP2-2;Q9BVP2	0.00E+00	0.00E+00	0.00E+00	0.00E+00	0.00E+00	2.59E+06	0.00E+00	0.00E+00	0.00E+00	0.00E+00	0.00E+00	0.00E+00	0.00E+00	0.00E+00	0.00E+00	0.00E+00	1.62E+05	3.24E+05	0.00E+00	6.47E+05	9.15E+05	0.00E+00	3.34E-01
Q9BW60-2;Q9BW60	0.00E+00	0.00E+00	0.00E+00	0.00E+00	0.00E+00	0.00E+00	0.00E+00	0.00E+00	0.00E+00	7.45E+05	0.00E+00	0.00E+00	0.00E+00	0.00E+00	0.00E+00	0.00E+00	4.66E+04	0.00E+00	9.31E+04	1.86E+05	0.00E+00	2.63E+05	3.34E-01
Q9BYD6;H0Y8N7	5.70E+06	0.00E+00	0.00E+00	0.00E+00	0.00E+00	0.00E+00	0.00E+00	0.00E+00	0.00E+00	0.00E+00	0.00E+00	0.00E+00	0.00E+00	0.00E+00	0.00E+00	0.00E+00	3.56E+05	7.13E+05	0.00E+00	1.43E+06	2.02E+06	0.00E+00	3.34E-01
Q9BZE4;Q5T3R7;Q9BZE4-2	0.00E+00	0.00E+00	0.00E+00	0.00E+00	0.00E+00	0.00E+00	0.00E+00	0.00E+00	0.00E+00	0.00E+00	0.00E+00	0.00E+00	0.00E+00	0.00E+00	0.00E+00	9.04E+05	5.65E+04	0.00E+00	1.13E+05	2.26E+05	0.00E+00	3.20E+05	3.34E-01
Q9H2U2-2;Q9H2U2-3;Q9H2U2;D6RAD3;D6RGV9	0.00E+00	0.00E+00	0.00E+00	0.00E+00	0.00E+00	2.35E+06	0.00E+00	0.00E+00	0.00E+00	0.00E+00	0.00E+00	0.00E+00	0.00E+00	0.00E+00	0.00E+00	0.00E+00	1.47E+05	2.93E+05	0.00E+00	5.86E+05	8.29E+05	0.00E+00	3.34E-01
Q9H444	0.00E+00	0.00E+00	0.00E+00	0.00E+00	0.00E+00	0.00E+00	0.00E+00	0.00E+00	0.00E+00	0.00E+00	0.00E+00	0.00E+00	0.00E+00	0.00E+00	0.00E+00	3.48E+06	2.18E+05	0.00E+00	4.36E+05	8.71E+05	0.00E+00	1.23E+06	3.34E-01
Q9H488-2;Q9H488	9.31E+06	0.00E+00	0.00E+00	0.00E+00	0.00E+00	0.00E+00	0.00E+00	0.00E+00	0.00E+00	0.00E+00	0.00E+00	0.00E+00	0.00E+00	0.00E+00	0.00E+00	0.00E+00	5.82E+05	1.16E+06	0.00E+00	2.33E+06	3.29E+06	0.00E+00	3.34E-01
Q9H5Q4	0.00E+00	0.00E+00	0.00E+00	0.00E+00	0.00E+00	0.00E+00	0.00E+00	0.00E+00	0.00E+00	0.00E+00	0.00E+00	5.35E+06	0.00E+00	0.00E+00	0.00E+00	0.00E+00	3.34E+05	0.00E+00	6.69E+05	1.34E+06	0.00E+00	1.89E+06	3.34E-01
Q9H5V8;Q9H5V8-2	0.00E+00	4.65E+06	0.00E+00	0.00E+00	0.00E+00	0.00E+00	0.00E+00	0.00E+00	0.00E+00	0.00E+00	0.00E+00	0.00E+00	0.00E+00	0.00E+00	0.00E+00	0.00E+00	2.91E+05	5.82E+05	0.00E+00	1.16E+06	1.65E+06	0.00E+00	3.34E-01
Q9H7Z7;A6NHH0;X6RJ95	0.00E+00	0.00E+00	0.00E+00	0.00E+00	0.00E+00	0.00E+00	0.00E+00	0.00E+00	0.00E+00	0.00E+00	0.00E+00	0.00E+00	0.00E+00	1.35E+06	0.00E+00	0.00E+00	8.42E+04	0.00E+00	1.68E+05	3.37E+05	0.00E+00	4.76E+05	3.34E-01
Q9HAV4	0.00E+00	0.00E+00	0.00E+00	0.00E+00	0.00E+00	0.00E+00	0.00E+00	0.00E+00	3.42E+06	0.00E+00	0.00E+00	0.00E+00	0.00E+00	0.00E+00	0.00E+00	0.00E+00	2.14E+05	0.00E+00	4.27E+05	8.55E+05	0.00E+00	1.21E+06	3.34E-01
Q9HBH5	0.00E+00	0.00E+00	0.00E+00	0.00E+00	0.00E+00	0.00E+00	0.00E+00	0.00E+00	0.00E+00	0.00E+00	0.00E+00	0.00E+00	2.49E+06	0.00E+00	0.00E+00	0.00E+00	1.56E+05	0.00E+00	3.11E+05	6.23E+05	0.00E+00	8.81E+05	3.34E-01
Q9HCC0-2;Q9HCC0;D6RD67	0.00E+00	0.00E+00	7.16E+05	0.00E+00	0.00E+00	0.00E+00	0.00E+00	0.00E+00	0.00E+00	0.00E+00	0.00E+00	0.00E+00	0.00E+00	0.00E+00	0.00E+00	0.00E+00	4.48E+04	8.95E+04	0.00E+00	1.79E+05	2.53E+05	0.00E+00	3.34E-01
Q9NP72	0.00E+00	0.00E+00	0.00E+00	0.00E+00	0.00E+00	0.00E+00	2.26E+08	0.00E+00	0.00E+00	0.00E+00	0.00E+00	0.00E+00	0.00E+00	0.00E+00	0.00E+00	0.00E+00	1.41E+07	2.82E+07	0.00E+00	5.65E+07	7.98E+07	0.00E+00	3.34E-01
Q9NPH2-2;Q9NPH2-3;Q9NPH2	0.00E+00	0.00E+00	0.00E+00	0.00E+00	0.00E+00	0.00E+00	0.00E+00	0.00E+00	0.00E+00	0.00E+00	0.00E+00	0.00E+00	0.00E+00	0.00E+00	0.00E+00	4.27E+05	2.67E+04	0.00E+00	5.33E+04	1.07E+05	0.00E+00	1.51E+05	3.34E-01
Q9NSB4	0.00E+00	0.00E+00	0.00E+00	0.00E+00	0.00E+00	0.00E+00	0.00E+00	5.95E+05	0.00E+00	0.00E+00	0.00E+00	0.00E+00	0.00E+00	0.00E+00	0.00E+00	0.00E+00	3.72E+04	7.44E+04	0.00E+00	1.49E+05	2.10E+05	0.00E+00	3.34E-01
Q9NT62-2;Q9NT62	0.00E+00	0.00E+00	0.00E+00	0.00E+00	0.00E+00	0.00E+00	0.00E+00	0.00E+00	0.00E+00	0.00E+00	0.00E+00	1.31E+05	0.00E+00	0.00E+00	0.00E+00	0.00E+00	8.17E+03	0.00E+00	1.63E+04	3.27E+04	0.00E+00	4.62E+04	3.34E-01
Q9NUQ9;E5RK61;E5RFS4;E5RHU5;E5RGI7;E5RJE1;E5RIR8;E5RJL8;E5RI16;Q9NUQ9-2	0.00E+00	0.00E+00	0.00E+00	0.00E+00	0.00E+00	0.00E+00	0.00E+00	0.00E+00	0.00E+00	0.00E+00	0.00E+00	0.00E+00	0.00E+00	0.00E+00	0.00E+00	7.43E+05	4.64E+04	0.00E+00	9.29E+04	1.86E+05	0.00E+00	2.63E+05	3.34E-01
Q9NUT2-5;Q9NUT2-4;Q9NUT2-3;Q9NUT2-2;Q9NUT2	0.00E+00	0.00E+00	0.00E+00	0.00E+00	0.00E+00	3.92E+05	0.00E+00	0.00E+00	0.00E+00	0.00E+00	0.00E+00	0.00E+00	0.00E+00	0.00E+00	0.00E+00	0.00E+00	2.45E+04	4.90E+04	0.00E+00	9.80E+04	1.39E+05	0.00E+00	3.34E-01
Q9NX47	0.00E+00	0.00E+00	0.00E+00	0.00E+00	0.00E+00	0.00E+00	0.00E+00	0.00E+00	0.00E+00	0.00E+00	1.05E+07	0.00E+00	0.00E+00	0.00E+00	0.00E+00	0.00E+00	6.57E+05	0.00E+00	1.31E+06	2.63E+06	0.00E+00	3.72E+06	3.34E-01
Q9NXG2	0.00E+00	0.00E+00	0.00E+00	0.00E+00	0.00E+00	0.00E+00	0.00E+00	0.00E+00	0.00E+00	0.00E+00	0.00E+00	0.00E+00	0.00E+00	0.00E+00	0.00E+00	7.89E+06	4.93E+05	0.00E+00	9.87E+05	1.97E+06	0.00E+00	2.79E+06	3.34E-01
Q9NZ01;Q9NZ01-2	0.00E+00	0.00E+00	0.00E+00	0.00E+00	0.00E+00	0.00E+00	0.00E+00	0.00E+00	0.00E+00	0.00E+00	0.00E+00	0.00E+00	0.00E+00	0.00E+00	0.00E+00	1.88E+06	1.17E+05	0.00E+00	2.35E+05	4.69E+05	0.00E+00	6.64E+05	3.34E-01

Q9NZL9-4;Q9NZL9-2;Q9NZL9;Q9NZL9-3	0.00E+00	0.00E+00	0.00E+00	0.00E+00	0.00E+00	0.00E+00	0.00E+00	0.00E+00	0.00E+00	0.00E+00	3.24E+06	0.00E+00	0.00E+00	0.00E+00	0.00E+00	0.00E+00	2.02E+05	0.00E+00	4.05E+05	8.10E+05	0.00E+00	1.15E+06	3.34E-01
Q9P032	0.00E+00	0.00E+00	0.00E+00	0.00E+00	0.00E+00	0.00E+00	0.00E+00	0.00E+00	0.00E+00	0.00E+00	0.00E+00	1.59E+05	0.00E+00	0.00E+00	0.00E+00	0.00E+00	9.96E+03	0.00E+00	1.99E+04	3.98E+04	0.00E+00	5.63E+04	3.34E-01
Q9P225-2;Q9P225;I3L520	0.00E+00	0.00E+00	0.00E+00	0.00E+00	0.00E+00	0.00E+00	0.00E+00	0.00E+00	9.00E+06	0.00E+00	0.00E+00	0.00E+00	0.00E+00	0.00E+00	0.00E+00	0.00E+00	5.63E+05	0.00E+00	1.13E+06	2.25E+06	0.00E+00	3.18E+06	3.34E-01
U3KPZ7;Q9P2N5	0.00E+00	0.00E+00	0.00E+00	0.00E+00	0.00E+00	0.00E+00	0.00E+00	7.25E+06	0.00E+00	0.00E+00	0.00E+00	0.00E+00	0.00E+00	0.00E+00	0.00E+00	0.00E+00	4.53E+05	9.06E+05	0.00E+00	1.81E+06	2.56E+06	0.00E+00	3.34E-01
Q9P2R7-2;Q9P2R7	0.00E+00	0.00E+00	0.00E+00	0.00E+00	0.00E+00	0.00E+00	0.00E+00	1.66E+06	0.00E+00	0.00E+00	0.00E+00	0.00E+00	0.00E+00	0.00E+00	0.00E+00	0.00E+00	1.03E+05	2.07E+05	0.00E+00	4.14E+05	5.85E+05	0.00E+00	3.34E-01
Q9UBR2	0.00E+00	0.00E+00	0.00E+00	0.00E+00	0.00E+00	4.87E+05	0.00E+00	0.00E+00	0.00E+00	0.00E+00	0.00E+00	0.00E+00	0.00E+00	0.00E+00	0.00E+00	0.00E+00	3.05E+04	6.09E+04	0.00E+00	1.22E+05	1.72E+05	0.00E+00	3.34E-01
Q9UFN0;Q9BS92	7.33E+06	0.00E+00	0.00E+00	0.00E+00	0.00E+00	0.00E+00	0.00E+00	0.00E+00	0.00E+00	0.00E+00	0.00E+00	0.00E+00	0.00E+00	0.00E+00	0.00E+00	0.00E+00	4.58E+05	9.16E+05	0.00E+00	1.83E+06	2.59E+06	0.00E+00	3.34E-01
Q9UGM6	0.00E+00	0.00E+00	0.00E+00	0.00E+00	0.00E+00	0.00E+00	0.00E+00	0.00E+00	0.00E+00	0.00E+00	0.00E+00	3.81E+05	0.00E+00	0.00E+00	0.00E+00	0.00E+00	2.38E+04	0.00E+00	4.76E+04	9.52E+04	0.00E+00	1.35E+05	3.34E-01
Q9UGP8	0.00E+00	0.00E+00	0.00E+00	0.00E+00	1.20E+06	0.00E+00	0.00E+00	0.00E+00	0.00E+00	0.00E+00	0.00E+00	0.00E+00	0.00E+00	0.00E+00	0.00E+00	0.00E+00	7.50E+04	1.50E+05	0.00E+00	3.00E+05	4.24E+05	0.00E+00	3.34E-01
Q9UH99-3;Q9UH99;Q9UH99-2	0.00E+00	0.00E+00	0.00E+00	0.00E+00	0.00E+00	1.46E+06	0.00E+00	0.00E+00	0.00E+00	0.00E+00	0.00E+00	0.00E+00	0.00E+00	0.00E+00	0.00E+00	0.00E+00	9.11E+04	1.82E+05	0.00E+00	3.65E+05	5.15E+05	0.00E+00	3.34E-01
Q9UHB9-4;Q9UHB9	4.05E+06	0.00E+00	0.00E+00	0.00E+00	0.00E+00	0.00E+00	0.00E+00	0.00E+00	0.00E+00	0.00E+00	0.00E+00	0.00E+00	0.00E+00	0.00E+00	0.00E+00	0.00E+00	2.53E+05	5.06E+05	0.00E+00	1.01E+06	1.43E+06	0.00E+00	3.34E-01
Q9UHD8-3;Q9UHD8-7;Q9UHD8-2;Q9UHD8-5;Q9UHD8;K7ERG1;K7EJ51;K7ER52;K7EIE4;K7EQD7;K7EK18;K7EL40;Q9UHD8-4;Q9UHD8-9;Q9UHD8-8	0.00E+00	0.00E+00	0.00E+00	0.00E+00	0.00E+00	0.00E+00	0.00E+00	0.00E+00	0.00E+00	0.00E+00	0.00E+00	0.00E+00	0.00E+00	0.00E+00	1.29E+06	0.00E+00	8.06E+04	0.00E+00	1.61E+05	3.22E+05	0.00E+00	4.56E+05	3.34E-01
Q9UHQ9;H7C0R7	0.00E+00	0.00E+00	0.00E+00	0.00E+00	0.00E+00	0.00E+00	0.00E+00	0.00E+00	0.00E+00	0.00E+00	0.00E+00	1.10E+06	0.00E+00	0.00E+00	0.00E+00	0.00E+00	6.89E+04	0.00E+00	1.38E+05	2.76E+05	0.00E+00	3.90E+05	3.34E-01
Q9UIJ7-2;Q9UIJ7-3;Q9UIJ7	0.00E+00	0.00E+00	0.00E+00	0.00E+00	0.00E+00	0.00E+00	2.67E+05	0.00E+00	0.00E+00	0.00E+00	0.00E+00	0.00E+00	0.00E+00	0.00E+00	0.00E+00	0.00E+00	1.67E+04	3.33E+04	0.00E+00	6.66E+04	9.42E+04	0.00E+00	3.34E-01
Q9UIW2;Q9HCM2-4;Q9HCM2	0.00E+00	0.00E+00	0.00E+00	0.00E+00	0.00E+00	0.00E+00	0.00E+00	1.03E+06	0.00E+00	0.00E+00	0.00E+00	0.00E+00	0.00E+00	0.00E+00	0.00E+00	0.00E+00	6.41E+04	1.28E+05	0.00E+00	2.56E+05	3.63E+05	0.00E+00	3.34E-01
Q9UL25	0.00E+00	0.00E+00	0.00E+00	0.00E+00	8.47E+06	0.00E+00	0.00E+00	0.00E+00	0.00E+00	0.00E+00	0.00E+00	0.00E+00	0.00E+00	0.00E+00	0.00E+00	0.00E+00	5.29E+05	1.06E+06	0.00E+00	2.12E+06	3.00E+06	0.00E+00	3.34E-01
Q9UMS4	0.00E+00	0.00E+00	0.00E+00	0.00E+00	0.00E+00	0.00E+00	0.00E+00	0.00E+00	0.00E+00	0.00E+00	0.00E+00	0.00E+00	0.00E+00	1.69E+06	0.00E+00	0.00E+00	1.06E+05	0.00E+00	2.11E+05	4.22E+05	0.00E+00	5.97E+05	3.34E-01
Q9UMX0-2;Q9UMX0;H0YDS0;Q9NRR5;Q9UHD9	0.00E+00	0.00E+00	0.00E+00	0.00E+00	0.00E+00	0.00E+00	0.00E+00	0.00E+00	0.00E+00	0.00E+00	8.88E+06	0.00E+00	0.00E+00	0.00E+00	0.00E+00	0.00E+00	5.55E+05	0.00E+00	1.11E+06	2.22E+06	0.00E+00	3.14E+06	3.34E-01
Q9UNQ2	0.00E+00	0.00E+00	0.00E+00	0.00E+00	0.00E+00	0.00E+00	0.00E+00	0.00E+00	0.00E+00	0.00E+00	0.00E+00	0.00E+00	0.00E+00	2.45E+05	0.00E+00	0.00E+00	1.53E+04	0.00E+00	3.06E+04	6.12E+04	0.00E+00	8.66E+04	3.34E-01
Q9UQ35	0.00E+00	0.00E+00	0.00E+00	0.00E+00	2.80E+06	0.00E+00	0.00E+00	0.00E+00	0.00E+00	0.00E+00	0.00E+00	0.00E+00	0.00E+00	0.00E+00	0.00E+00	0.00E+00	1.75E+05	3.50E+05	0.00E+00	6.99E+05	9.89E+05	0.00E+00	3.34E-01
Q9UQE7	0.00E+00	0.00E+00	0.00E+00	0.00E+00	0.00E+00	1.39E+06	0.00E+00	0.00E+00	0.00E+00	0.00E+00	0.00E+00	0.00E+00	0.00E+00	0.00E+00	0.00E+00	0.00E+00	8.69E+04	1.74E+05	0.00E+00	3.48E+05	4.92E+05	0.00E+00	3.34E-01
Q9Y223-5;Q9Y223-4;Q9Y223-3;Q9Y223;Q9Y223-2	0.00E+00	5.29E+05	0.00E+00	0.00E+00	0.00E+00	0.00E+00	0.00E+00	0.00E+00	0.00E+00	0.00E+00	0.00E+00	0.00E+00	0.00E+00	0.00E+00	0.00E+00	0.00E+00	3.31E+04	6.61E+04	0.00E+00	1.32E+05	1.87E+05	0.00E+00	3.34E-01
Q9Y224	0.00E+00	0.00E+00	0.00E+00	0.00E+00	0.00E+00	0.00E+00	0.00E+00	0.00E+00	0.00E+00	0.00E+00	0.00E+00	0.00E+00	0.00E+00	0.00E+00	0.00E+00	4.14E+06	2.59E+05	0.00E+00	5.17E+05	1.03E+06	0.00E+00	1.46E+06	3.34E-01
Q9Y276;C9J1S9;H7C492	0.00E+00	0.00E+00	0.00E+00	0.00E+00	0.00E+00	0.00E+00	0.00E+00	0.00E+00	0.00E+00	0.00E+00	0.00E+00	0.00E+00	0.00E+00	0.00E+00	4.56E+06	0.00E+00	2.85E+05	0.00E+00	5.70E+05	1.14E+06	0.00E+00	1.61E+06	3.34E-01
Q9Y2W1	0.00E+00	0.00E+00	0.00E+00	0.00E+00	0.00E+00	0.00E+00	0.00E+00	0.00E+00	0.00E+00	0.00E+00	0.00E+00	0.00E+00	1.32E+06	0.00E+00	0.00E+00	0.00E+00	8.27E+04	0.00E+00	1.65E+05	3.31E+05	0.00E+00	4.68E+05	3.34E-01
Q9Y2Z0-2;Q9Y2Z0	0.00E+00	0.00E+00	0.00E+00	0.00E+00	0.00E+00	0.00E+00	0.00E+00	0.00E+00	0.00E+00	0.00E+00	0.00E+00	6.58E+06	0.00E+00	0.00E+00	0.00E+00	0.00E+00	4.11E+05	0.00E+00	8.22E+05	1.64E+06	0.00E+00	2.33E+06	3.34E-01
Q9Y3A6	0.00E+00	0.00E+00	0.00E+00	0.00E+00	0.00E+00	0.00E+00	0.00E+00	0.00E+00	0.00E+00	0.00E+00	0.00E+00	0.00E+00	0.00E+00	0.00E+00	0.00E+00	2.85E+06	1.78E+05	0.00E+00	3.56E+05	7.13E+05	0.00E+00	1.01E+06	3.34E-01
Q9Y3B7-2;Q9Y3B7-3;Q9Y3B7;H0YCD5	0.00E+00	0.00E+00	0.00E+00	0.00E+00	0.00E+00	0.00E+00	3.02E+06	0.00E+00	0.00E+00	0.00E+00	0.00E+00	0.00E+00	0.00E+00	0.00E+00	0.00E+00	0.00E+00	1.88E+05	3.77E+05	0.00E+00	7.54E+05	1.07E+06	0.00E+00	3.34E-01
Q9Y3F4;Q9Y3F4-2	0.00E+00	0.00E+00	0.00E+00	0.00E+00	0.00E+00	0.00E+00	0.00E+00	0.00E+00	0.00E+00	0.00E+00	1.25E+07	0.00E+00	0.00E+00	0.00E+00	0.00E+00	0.00E+00	7.80E+05	0.00E+00	1.56E+06	3.12E+06	0.00E+00	4.41E+06	3.34E-01
Q9Y3I0	0.00E+00	0.00E+00	0.00E+00	0.00E+00	0.00E+00	0.00E+00	1.23E+06	0.00E+00	0.00E+00	0.00E+00	0.00E+00	0.00E+00	0.00E+00	0.00E+00	0.00E+00	0.00E+00	7.70E+04	1.54E+05	0.00E+00	3.08E+05	4.35E+05	0.00E+00	3.34E-01
Q9Y5B9	0.00E+00	0.00E+00	0.00E+00	0.00E+00	4.92E+05	0.00E+00	0.00E+00	0.00E+00	0.00E+00	0.00E+00	0.00E+00	0.00E+00	0.00E+00	0.00E+00	0.00E+00	0.00E+00	3.07E+04	6.15E+04	0.00E+00	1.23E+05	1.74E+05	0.00E+00	3.34E-01
Q9Y5S9-2;Q9Y5S9	0.00E+00	0.00E+00	0.00E+00	0.00E+00	0.00E+00	0.00E+00	0.00E+00	0.00E+00	0.00E+00	0.00E+00	0.00E+00	0.00E+00	0.00E+00	0.00E+00	0.00E+00	5.98E+06	3.74E+05	0.00E+00	7.47E+05	1.49E+06	0.00E+00	2.11E+06	3.34E-01
Q9Y613	0.00E+00	0.00E+00	0.00E+00	0.00E+00	0.00E+00	0.00E+00	0.00E+00	0.00E+00	0.00E+00	0.00E+00	1.43E+06	0.00E+00	0.00E+00	0.00E+00	0.00E+00	0.00E+00	8.94E+04	0.00E+00	1.79E+05	3.58E+05	0.00E+00	5.06E+05	3.34E-01
Q9Y617;Q9Y617-2	4.40E+06	0.00E+00	0.00E+00	0.00E+00	0.00E+00	0.00E+00	0.00E+00	0.00E+00	0.00E+00	0.00E+00	0.00E+00	0.00E+00	0.00E+00	0.00E+00	0.00E+00	0.00E+00	2.75E+05	5.50E+05	0.00E+00	1.10E+06	1.56E+06	0.00E+00	3.34E-01

Q9Y678	3.08E+07	4.78E+07	0.00E+00	1.74E+07	1.28E+07	8.43E+07	8.71E+07	5.70E+07	0.00E+00	0.00E+00	1.27E+07	1.24E+07	5.99E+07	0.00E+00	8.10E+07	4.35E+07	3.42E+07	4.21E+07	2.62E+07	3.20E+07	3.26E+07	3.13E+07	3.35E-01
Q9Y6M5	0.00E+00	0.00E+00	0.00E+00	4.85E+06	0.00E+00	0.00E+00	0.00E+00	7.64E+06	7.64E+06	0.00E+00	0.00E+00	5.02E+06	6.05E+06	0.00E+00	0.00E+00	6.71E+06	2.37E+06	1.56E+06	3.18E+06	3.24E+06	2.99E+06	3.47E+06	3.35E-01
P23634-7;P23634-6;P23634-8;P23634;P23634-5;P23634-4;P23634-3;P23634-2;P20020-5;P20020-2;P20020-6;P20020-3;P20020-4;P20020	3.62E+07	2.77E+07	2.36E+07	1.06E+07	0.00E+00	3.13E+07	3.29E+07	2.24E+07	0.00E+00	0.00E+00	0.00E+00	3.24E+07	4.04E+07	0.00E+00	2.13E+07	3.05E+07	1.93E+07	2.31E+07	1.56E+07	1.50E+07	1.22E+07	1.74E+07	3.36E-01
P51148;P51148-2;K7ERI8;K7ERQ8;F8VVK3;K7ENY4	3.28E+07	0.00E+00	4.00E+07	3.55E+07	3.50E+07	3.26E+07	0.00E+00	2.18E+07	2.34E+07	3.23E+07	3.96E+07	3.46E+07	2.74E+07	4.43E+07	2.56E+07	2.10E+07	2.79E+07	2.47E+07	3.10E+07	1.28E+07	1.61E+07	8.16E+06	3.40E-01
P49748-2;P49748;P49748-3	0.00E+00	0.00E+00	0.00E+00	0.00E+00	0.00E+00	2.07E+06	0.00E+00	2.32E+06	0.00E+00	0.00E+00	0.00E+00	0.00E+00	0.00E+00	3.28E+06	6.54E+06	1.82E+06	1.00E+06	5.49E+05	1.45E+06	1.83E+06	1.02E+06	2.39E+06	3.40E-01
P36543-2;P36543;P36543-3;C9J8H1	1.19E+07	0.00E+00	7.33E+06	2.92E+07	2.97E+07	2.34E+07	5.96E+06	4.97E+06	0.00E+00	1.98E+07	1.36E+07	2.12E+07	8.56E+06	0.00E+00	0.00E+00	8.86E+06	1.15E+07	1.41E+07	9.00E+06	1.03E+07	1.17E+07	8.69E+06	3.43E-01
P13995;B9A062;P13995-2	0.00E+00	0.00E+00	0.00E+00	0.00E+00	0.00E+00	0.00E+00	4.27E+06	1.07E+07	4.23E+06	4.51E+06	0.00E+00	0.00E+00	0.00E+00	6.36E+06	6.41E+06	7.24E+06	2.73E+06	1.87E+06	3.59E+06	3.51E+06	3.86E+06	3.14E+06	3.43E-01
P49588;P49588-2	0.00E+00	0.00E+00	2.20E+07	1.45E+07	1.66E+07	1.91E+07	7.40E+06	1.97E+07	1.23E+07	1.48E+07	0.00E+00	9.58E+06	1.19E+07	1.20E+07	0.00E+00	9.85E+06	1.06E+07	1.24E+07	8.80E+06	7.40E+06	8.82E+06	5.66E+06	3.46E-01
P52209-2;P52209;K7EMN2;K7EM49;K7EPF6	2.02E+07	2.82E+07	2.39E+07	7.48E+06	3.15E+07	1.92E+07	1.21E+07	4.08E+07	1.84E+07	0.00E+00	1.51E+07	3.03E+07	3.00E+07	2.10E+07	0.00E+00	2.47E+07	2.02E+07	2.29E+07	1.74E+07	1.13E+07	1.07E+07	1.20E+07	3.48E-01
O00299	0.00E+00	0.00E+00	1.01E+07	0.00E+00	0.00E+00	1.28E+07	1.17E+07	1.23E+07	0.00E+00	0.00E+00	0.00E+00	0.00E+00	1.24E+07	0.00E+00	0.00E+00	1.15E+07	4.43E+06	5.87E+06	2.99E+06	5.93E+06	6.32E+06	5.54E+06	3.48E-01
Q99714;Q99714-2;Q5H928	2.83E+07	4.59E+07	6.54E+07	4.40E+07	4.47E+07	7.07E+07	0.00E+00	5.77E+07	1.02E+07	5.23E+07	3.80E+07	1.04E+07	4.16E+07	6.45E+06	6.00E+07	5.21E+07	3.92E+07	4.46E+07	3.39E+07	2.20E+07	2.25E+07	2.17E+07	3.49E-01
P29401;P29401-2	2.29E+08	3.53E+08	6.00E+08	2.79E+08	5.68E+08	6.49E+08	6.28E+08	5.59E+08	3.74E+08	4.59E+08	4.89E+08	3.29E+08	5.24E+08	5.54E+06	5.73E+08	4.47E+08	4.42E+08	4.83E+08	4.00E+08	1.72E+08	1.68E+08	1.77E+08	3.53E-01
P04406-2;P04406;E7EUT5	2.14E+07	3.50E+07	2.00E+07	1.88E+07	3.60E+07	0.00E+00	3.10E+07	2.24E+07	0.00E+00	2.54E+07	2.94E+07	0.00E+00	3.43E+07	1.49E+07	1.71E+07	1.71E+07	2.02E+07	2.31E+07	1.73E+07	1.20E+07	1.16E+07	1.26E+07	3.53E-01
P41252;J3KR24;A0A0A0MSX9	1.34E+08	2.25E+08	9.18E+07	1.41E+08	1.99E+08	1.19E+08	1.84E+08	1.44E+08	0.00E+00	1.32E+08	1.38E+08	1.72E+08	1.96E+08	3.36E+07	1.93E+08	1.45E+08	1.40E+08	1.55E+08	1.26E+08	5.97E+07	4.44E+07	7.22E+07	3.57E-01
P04075;J3KPS3;P04075-2;H3BQN4;H3BPS8;H3BUH7	0.00E+00	0.00E+00	0.00E+00	0.00E+00	0.00E+00	0.00E+00	2.76E+06	0.00E+00	0.00E+00	0.00E+00	0.00E+00	0.00E+00	0.00E+00	4.44E+06	0.00E+00	4.43E+06	7.27E+05	3.45E+05	1.11E+06	1.60E+06	9.75E+05	2.05E+06	3.58E-01
P13489;E9PMN0;E9PIK5;E9PMJ3;E9PLZ3;E9PIM9;H0YCR7	8.37E+06	5.65E+06	7.22E+06	0.00E+00	0.00E+00	0.00E+00	0.00E+00	0.00E+00	0.00E+00	0.00E+00	0.00E+00	4.42E+06	5.18E+06	0.00E+00	0.00E+00	0.00E+00	1.93E+06	2.66E+06	1.20E+06	3.07E+06	3.74E+06	2.23E+06	3.60E-01
Q06323;Q06323-3;Q06323-2	0.00E+00	0.00E+00	0.00E+00	2.71E+06	0.00E+00	0.00E+00	0.00E+00	0.00E+00	3.60E+06	0.00E+00	0.00E+00	0.00E+00	0.00E+00	5.03E+06	0.00E+00	0.00E+00	7.09E+05	3.39E+05	1.08E+06	1.58E+06	9.59E+05	2.04E+06	3.68E-01
P23786	9.86E+06	0.00E+00	1.39E+07	0.00E+00	1.02E+07	0.00E+00	0.00E+00	1.27E+07	1.24E+07	0.00E+00	0.00E+00	2.30E+07	1.27E+07	1.57E+07	0.00E+00	1.07E+07	7.57E+06	5.82E+06	9.32E+06	7.50E+06	6.35E+06	8.56E+06	3.69E-01
Q92896;Q92896-3;Q92896-2;H3BM42	0.00E+00	8.57E+07	0.00E+00	3.97E+07	3.70E+07	0.00E+00	7.62E+07	7.80E+07	1.49E+07	3.84E+07	3.58E+07	0.00E+00	3.85E+07	0.00E+00	4.42E+07	3.64E+07	3.28E+07	3.96E+07	2.60E+07	2.91E+07	3.71E+07	1.82E+07	3.70E-01
P34932;A0A087WYC1;A0A087WTS8	0.00E+00	0.00E+00	0.00E+00	0.00E+00	0.00E+00	3.08E+06	0.00E+00	0.00E+00	0.00E+00	0.00E+00	0.00E+00	5.84E+06	3.78E+06	0.00E+00	0.00E+00	0.00E+00	7.94E+05	3.85E+05	1.20E+06	1.79E+06	1.09E+06	2.29E+06	3.77E-01
O60762;Q5QPK2;H0Y368;Q5QPJ9	0.00E+00	0.00E+00	0.00E+00	0.00E+00	0.00E+00	2.23E+07	0.00E+00	1.78E+07	0.00E+00	0.00E+00	2.34E+07	1.58E+07	0.00E+00	1.89E+07	0.00E+00	1.79E+07	7.25E+06	5.01E+06	9.49E+06	9.81E+06	9.35E+06	1.04E+07	3.79E-01
F8VVM2;Q00325-2;Q00325;F8VZL5;F8VWR4;F8VWQ0	3.28E+07	2.91E+07	2.91E+07	4.05E+07	0.00E+00	3.85E+07	0.00E+00	3.15E+07	3.66E+07	3.21E+07	6.82E+07	2.64E+07	2.04E+07	1.41E+07	2.09E+07	4.32E+07	2.90E+07	2.52E+07	3.27E+07	1.65E+07	1.61E+07	1.72E+07	3.80E-01
P20700;E9PBF6	4.25E+07	5.68E+07	2.67E+07	4.28E+07	5.85E+07	0.00E+00	4.64E+07	4.67E+07	1.04E+07	2.32E+07	2.86E+07	1.36E+07	6.24E+07	3.01E+07	5.19E+07	3.36E+07	3.59E+07	4.00E+07	3.17E+07	1.82E+07	1.89E+07	1.78E+07	3.80E-01
Q00341;A0A024R4E5;Q00341-2;H0Y394	0.00E+00	0.00E+00	0.00E+00	4.86E+07	0.00E+00	0.00E+00	0.00E+00	0.00E+00	7.21E+07	0.00E+00	7.23E+07	0.00E+00	0.00E+00	0.00E+00	0.00E+00	0.00E+00	1.21E+07	6.07E+06	1.80E+07	2.64E+07	1.72E+07	3.34E+07	3.83E-01

Q5VTQ0-7;Q5VTQ0-5;Q5VTQ0-2;Q5VTQ0-6;Q5VTQ0-3;Q5VTQ0-4;Q5VTQ0	0.00E+00	0.00E+00	0.00E+00	0.00E+00	0.00E+00	0.00E+00	1.12E+07	0.00E+00	0.00E+00	0.00E+00	0.00E+00	1.87E+07	0.00E+00	0.00E+00	0.00E+00	1.47E+07	2.79E+06	1.40E+06	4.17E+06	6.15E+06	3.96E+06	7.80E+06	3.85E-01
P49721;A0A087WVV1	0.00E+00	0.00E+00	0.00E+00	0.00E+00	0.00E+00	0.00E+00	0.00E+00	6.65E+06	0.00E+00	0.00E+00	0.00E+00	0.00E+00	7.26E+06	1.35E+07	0.00E+00	0.00E+00	1.71E+06	8.32E+05	2.59E+06	3.93E+06	2.35E+06	5.07E+06	3.89E-01
P53597	0.00E+00	0.00E+00	0.00E+00	0.00E+00	0.00E+00	6.45E+06	0.00E+00	0.00E+00	8.72E+06	0.00E+00	9.70E+06	0.00E+00	0.00E+00	0.00E+00	0.00E+00	0.00E+00	1.55E+06	8.06E+05	2.30E+06	3.40E+06	2.28E+06	4.27E+06	3.97E-01
Q9P035;Q9P035-2;H3BPZ1;H3BS72;H3BRL8	4.10E+08	2.92E+08	3.26E+08	4.39E+08	4.42E+08	6.05E+08	3.05E+08	4.79E+08	5.69E+08	3.97E+08	6.61E+06	5.04E+08	3.65E+08	2.45E+08	3.86E+08	3.31E+08	3.81E+08	4.12E+08	3.50E+08	1.41E+08	1.05E+08	1.71E+08	3.99E-01
P06576;H0YH81;F8W079	2.83E+07	7.28E+06	1.78E+07	1.06E+07	1.77E+07	2.45E+07	1.22E+07	2.60E+07	3.27E+07	0.00E+00	0.00E+00	1.87E+07	1.13E+07	3.09E+07	0.00E+00	1.31E+07	1.57E+07	1.80E+07	1.33E+07	1.08E+07	7.72E+06	1.34E+07	4.01E-01
Q13011;M0R248	3.69E+07	3.50E+07	2.57E+07	1.14E+07	1.72E+07	2.42E+07	1.86E+07	1.51E+07	1.37E+07	0.00E+00	1.34E+07	2.77E+07	4.18E+07	0.00E+00	2.91E+07	1.67E+07	2.04E+07	2.30E+07	1.78E+07	1.20E+07	9.21E+06	1.45E+07	4.05E-01
Q9Y277;Q9Y277-2;E5RJN6;E5RHZ6;E5RK27;E5RFP6	0.00E+00	1.34E+07	0.00E+00	0.00E+00	1.01E+07	0.00E+00	0.00E+00	8.30E+06	7.63E+06	9.23E+06	1.19E+07	4.67E+06	6.41E+06	0.00E+00	0.00E+00	9.27E+06	5.06E+06	3.97E+06	6.14E+06	5.00E+06	5.65E+06	4.35E+06	4.05E-01
P11216	0.00E+00	7.38E+06	7.10E+06	0.00E+00	0.00E+00	0.00E+00	0.00E+00	7.46E+06	0.00E+00	5.56E+06	0.00E+00	0.00E+00	0.00E+00	5.35E+06	0.00E+00	0.00E+00	2.05E+06	2.74E+06	1.36E+06	3.19E+06	3.79E+06	2.52E+06	4.06E-01
Q14157-4;Q14157-1;Q14157-3;F8W726;Q14157;Q14157-5	0.00E+00	0.00E+00	0.00E+00	0.00E+00	0.00E+00	0.00E+00	7.79E+06	1.31E+07	0.00E+00	0.00E+00	0.00E+00	0.00E+00	0.00E+00	1.99E+07	1.17E+07	1.19E+07	4.03E+06	2.61E+06	5.44E+06	6.58E+06	5.04E+06	7.92E+06	4.08E-01
E9PPJ0;Q13435;A0A087WZZ5;E9PJ04;H0YCG1;E9PJT3	4.73E+07	3.54E+07	3.14E+07	3.67E+07	3.35E+07	4.58E+07	2.95E+07	4.01E+07	5.18E+07	4.62E+07	1.79E+07	3.91E+07	1.31E+07	2.30E+07	4.88E+07	1.87E+07	3.49E+07	3.75E+07	3.23E+07	1.19E+07	6.47E+06	1.58E+07	4.10E-01
P24752	0.00E+00	0.00E+00	1.86E+06	0.00E+00	0.00E+00	0.00E+00	5.18E+06	1.95E+06	0.00E+00	0.00E+00	0.00E+00	0.00E+00	1.47E+06	0.00E+00	0.00E+00	2.50E+06	8.10E+05	1.12E+06	4.97E+05	1.46E+06	1.85E+06	9.61E+05	4.10E-01
Q9UL46;A0A087X1Z3;H0YM70	0.00E+00	0.00E+00	1.34E+07	0.00E+00	9.76E+06	9.46E+06	1.85E+07	1.41E+07	1.06E+07	1.12E+07	0.00E+00	0.00E+00	1.00E+07	0.00E+00	0.00E+00	1.12E+07	6.77E+06	8.17E+06	5.37E+06	6.52E+06	7.32E+06	5.76E+06	4.10E-01
P35580;P35580-5;P35580-2;P35580-3;P35580-4	1.35E+07	0.00E+00	0.00E+00	0.00E+00	0.00E+00	1.15E+07	1.04E+07	3.14E+06	7.76E+06	0.00E+00	0.00E+00	8.94E+06	8.44E+06	1.79E+07	0.00E+00	1.86E+07	6.26E+06	4.82E+06	7.70E+06	6.75E+06	5.94E+06	7.58E+06	4.11E-01
O75643	2.86E+08	4.41E+08	3.91E+08	1.02E+08	2.30E+08	0.00E+00	3.28E+08	3.91E+08	2.60E+08	4.29E+08	2.99E+08	3.03E+08	3.88E+08	2.41E+08	3.60E+08	2.85E+08	2.96E+08	2.71E+08	3.21E+08	1.17E+08	1.53E+08	6.53E+07	4.14E-01
P07900;P07900-2	4.55E+07	2.30E+07	3.07E+07	2.62E+07	1.97E+07	3.61E+07	1.83E+07	3.91E+07	2.68E+07	0.00E+00	0.00E+00	4.85E+06	2.58E+07	7.49E+07	6.25E+06	3.53E+07	2.58E+07	2.98E+07	2.17E+07	1.91E+07	9.75E+06	2.54E+07	4.14E-01
P13804;P13804-2;H0YLU7;H0YK49;H0YNX6;H0YL12;H0YKF0	0.00E+00	7.47E+06	7.85E+06	0.00E+00	0.00E+00	0.00E+00	0.00E+00	0.00E+00	1.33E+07	9.41E+06	8.20E+06	0.00E+00	0.00E+00	0.00E+00	0.00E+00	0.00E+00	2.89E+06	1.91E+06	3.86E+06	4.59E+06	3.55E+06	5.52E+06	4.15E-01
Q01082;A0A087WUZ3;Q01082-3;Q01082-2	0.00E+00	3.25E+07	1.66E+07	0.00E+00	0.00E+00	1.33E+07	1.33E+07	4.40E+07	0.00E+00	0.00E+00	0.00E+00	4.55E+06	2.86E+07	0.00E+00	2.77E+07	1.06E+07	1.20E+07	1.50E+07	8.95E+06	1.43E+07	1.62E+07	1.24E+07	4.18E-01
M0R0F0;P46782;M0QZN2;M0R0R2	0.00E+00	0.00E+00	0.00E+00	0.00E+00	0.00E+00	0.00E+00	0.00E+00	5.77E+06	0.00E+00	1.05E+07	0.00E+00	0.00E+00	0.00E+00	0.00E+00	0.00E+00	5.55E+06	1.36E+06	7.21E+05	2.01E+06	3.10E+06	2.04E+06	3.94E+06	4.27E-01
B4DKB2;P42892-3;P42892-2;P42892-4;P42892	0.00E+00	1.22E+07	0.00E+00	0.00E+00	0.00E+00	0.00E+00	1.74E+07	2.50E+07	6.52E+06	0.00E+00	5.75E+06	8.66E+06	1.31E+07	1.14E+07	1.76E+07	1.90E+07	8.55E+06	6.84E+06	1.03E+07	8.31E+06	1.00E+07	6.35E+06	4.29E-01
O14980	4.75E+07	5.03E+07	5.32E+07	3.49E+07	6.75E+07	0.00E+00	6.78E+07	6.42E+07	3.90E+07	4.85E+07	3.32E+07	3.02E+07	3.48E+07	5.28E+07	4.72E+07	4.48E+07	4.47E+07	4.82E+07	4.13E+07	1.67E+07	2.25E+07	8.18E+06	4.30E-01
Q14974;Q14974-2;J3KTM9	4.26E+08	4.64E+08	4.02E+08	4.93E+08	5.81E+08	6.09E+08	0.00E+00	4.82E+08	4.14E+08	5.66E+08	6.67E+08	3.43E+08	5.76E+08	3.36E+08	5.49E+08	5.17E+08	4.64E+08	4.32E+08	4.96E+08	1.56E+08	1.88E+08	1.19E+08	4.31E-01
P11142;E9PKE3;P11142-2;E9PNE6;E9PN89;A8K7Q2	0.00E+00	1.41E+07	0.00E+00	0.00E+00	1.10E+07	2.48E+07	2.20E+07	2.20E+07	0.00E+00	0.00E+00	0.00E+00	0.00E+00	2.00E+07	0.00E+00	2.16E+07	1.82E+07	9.59E+06	1.17E+07	7.47E+06	1.04E+07	1.07E+07	1.03E+07	4.33E-01
P60900;G3V5Z7;G3V3I1;G3V295;P60900-2;G3V3U4	1.51E+07	3.07E+07	2.76E+07	9.46E+06	7.81E+06	1.46E+07	1.89E+07	1.69E+07	8.02E+06	1.52E+07	2.20E+07	0.00E+00	2.73E+07	0.00E+00	2.69E+07	1.07E+07	1.57E+07	1.76E+07	1.38E+07	9.52E+06	8.04E+06	1.10E+07	4.35E-01
P78371;F5GWF6;F8VQ14;P78371-2	0.00E+00	4.16E+07	3.90E+07	2.86E+07	2.51E+07	4.82E+07	8.15E+07	6.28E+07	0.00E+00	3.45E+07	2.72E+07	3.39E+07	6.62E+07	0.00E+00	4.45E+07	4.47E+07	3.61E+07	4.09E+07	3.14E+07	2.34E+07	2.47E+07	2.26E+07	4.36E-01
P62826;B5MDF5;J3KQE5;F5H018	0.00E+00	1.20E+07	7.97E+06	0.00E+00	0.00E+00	6.84E+06	9.36E+06	7.32E+06	0.00E+00	0.00E+00	0.00E+00	7.60E+06	8.80E+06	0.00E+00	5.58E+06	7.42E+06	4.55E+06	5.43E+06	3.67E+06	4.35E+06	4.76E+06	4.02E+06	4.39E-01

P00492	4.80E+06	0.00E+00	9.65E+06	0.00E+00	0.00E+00	6.30E+06	0.00E+00	0.00E+00	3.59E+06	0.00E+00	6.82E+06	0.00E+00	0.00E+00	0.00E+00	0.00E+00	0.00E+00	1.95E+06	2.59E+06	1.30E+06	3.21E+06	3.82E+06	2.56E+06	4.40E-01
P54136-2;P54136	6.41E+07	6.10E+07	7.26E+07	5.22E+07	5.13E+07	2.38E+07	5.21E+07	6.31E+07	5.68E+07	3.78E+07	5.34E+07	5.57E+07	4.58E+07	6.29E+07	4.58E+07	4.42E+07	5.26E+07	5.50E+07	5.03E+07	1.17E+07	1.46E+07	8.24E+06	4.42E-01
P05023-3;P05023-4;P05023;P05023-2	3.57E+06	0.00E+00	6.00E+06	0.00E+00	4.31E+06	2.78E+06	1.28E+07	7.15E+06	4.68E+06	4.42E+06	3.67E+06	0.00E+00	3.52E+06	0.00E+00	2.70E+06	6.92E+06	3.91E+06	4.58E+06	3.24E+06	3.35E+06	4.19E+06	2.35E+06	4.43E-01
P27695;G3V5Q1;G3V3M6;G3V5M0;G3V359;G3V3C7;H7C4A8	4.34E+07	6.84E+07	3.97E+07	2.15E+07	2.41E+07	0.00E+00	5.34E+07	4.30E+07	3.00E+07	3.18E+07	2.90E+07	3.24E+07	6.56E+07	1.08E+07	1.52E+07	1.84E+07	3.29E+07	3.67E+07	2.91E+07	1.89E+07	2.11E+07	1.69E+07	4.43E-01
Q14152;Q14152-2	0.00E+00	0.00E+00	0.00E+00	0.00E+00	0.00E+00	0.00E+00	1.85E+07	1.26E+07	0.00E+00	0.00E+00	0.00E+00	1.18E+07	1.59E+07	0.00E+00	1.13E+07	1.53E+07	5.33E+06	3.88E+06	6.78E+06	7.29E+06	7.36E+06	7.41E+06	4.45E-01
J3QR09;J3KTE4;P84098;J3QL15	1.37E+07	5.40E+06	1.73E+07	9.71E+06	1.29E+07	3.38E+07	1.28E+07	2.76E+07	8.97E+06	0.00E+00	0.00E+00	0.00E+00	1.52E+07	4.77E+07	0.00E+00	1.91E+07	1.40E+07	1.67E+07	1.14E+07	1.33E+07	9.47E+06	1.66E+07	4.47E-01
P36542;P36542-2	2.22E+08	1.25E+08	1.65E+08	2.13E+08	1.47E+08	2.14E+08	1.21E+08	1.68E+08	1.11E+08	2.49E+08	5.25E+06	2.40E+08	1.36E+08	2.34E+08	0.00E+00	1.63E+08	1.57E+08	1.72E+08	1.42E+08	7.51E+07	4.03E+07	9.98E+07	4.48E-01
P49411	0.00E+00	5.97E+06	0.00E+00	0.00E+00	0.00E+00	0.00E+00	8.52E+06	0.00E+00	0.00E+00	1.06E+07	0.00E+00	0.00E+00	0.00E+00	0.00E+00	7.60E+06	9.28E+06	2.62E+06	1.81E+06	3.43E+06	4.12E+06	3.42E+06	4.81E+06	4.50E-01
O00410;H0Y8C6;O00410-3;O00410-2	5.69E+06	0.00E+00	0.00E+00	0.00E+00	0.00E+00	0.00E+00	0.00E+00	0.00E+00	0.00E+00	0.00E+00	0.00E+00	1.34E+07	3.58E+06	0.00E+00	0.00E+00	0.00E+00	1.42E+06	7.12E+05	2.12E+06	3.58E+06	2.01E+06	4.72E+06	4.50E-01
P09661;H0YMA0;H0YLR3;H0YKK0	5.43E+07	1.71E+07	2.81E+07	1.50E+07	1.60E+07	1.23E+07	0.00E+00	0.00E+00	2.58E+07	1.36E+07	1.60E+07	0.00E+00	0.00E+00	3.79E+07	0.00E+00	0.00E+00	1.48E+07	1.78E+07	1.17E+07	1.57E+07	1.74E+07	1.44E+07	4.51E-01
O95202	0.00E+00	0.00E+00	0.00E+00	0.00E+00	0.00E+00	0.00E+00	4.86E+06	0.00E+00	0.00E+00	0.00E+00	0.00E+00	0.00E+00	7.12E+06	0.00E+00	0.00E+00	5.07E+06	1.07E+06	6.07E+05	1.52E+06	2.34E+06	1.72E+06	2.87E+06	4.52E-01
O75396;A0A087X1A9	0.00E+00	0.00E+00	0.00E+00	0.00E+00	0.00E+00	3.84E+06	2.30E+06	0.00E+00	0.00E+00	0.00E+00	0.00E+00	0.00E+00	0.00E+00	0.00E+00	2.41E+06	0.00E+00	5.34E+05	7.67E+05	3.02E+05	1.19E+06	1.48E+06	8.53E+05	4.53E-01
Q14249	0.00E+00	0.00E+00	0.00E+00	0.00E+00	0.00E+00	0.00E+00	9.81E+06	9.74E+06	0.00E+00	0.00E+00	0.00E+00	0.00E+00	0.00E+00	0.00E+00	0.00E+00	7.94E+06	1.72E+06	2.44E+06	9.92E+05	3.71E+06	4.53E+06	2.81E+06	4.54E-01
F5GWX5;Q14839;Q14839-2	0.00E+00	6.28E+06	0.00E+00	0.00E+00	0.00E+00	4.77E+06	6.02E+06	7.41E+06	0.00E+00	0.00E+00	0.00E+00	3.98E+06	6.41E+06	0.00E+00	0.00E+00	4.74E+06	2.48E+06	3.06E+06	1.89E+06	3.00E+06	3.35E+06	2.69E+06	4.54E-01
P61224-3;P61224;A6NIZ1;F5H823;B7ZB78;F5GZG1;P61224-2;F5GX62;P61224-4;E7ESV4;F5H7Y6;P62834;F5H6R7;F5H004	1.21E+07	9.83E+06	0.00E+00	1.03E+07	0.00E+00	9.69E+06	8.40E+06	1.01E+07	0.00E+00	5.19E+06	9.01E+06	1.06E+07	6.82E+06	6.71E+06	0.00E+00	8.80E+06	6.72E+06	7.56E+06	5.89E+06	4.34E+06	4.78E+06	3.99E+06	4.59E-01
Q9Y6M7-4;Q9Y6M7-3;C9JRP1;Q9Y6M7-2;Q9Y6M7-9;Q9Y6M7-6;Q9Y6M7-13;A0A0A0MST8;Q9Y6M7-12;E9PFN4;Q9Y6M7;Q9Y6M7-8;Q9Y6M7-7;Q9Y6M7-11;Q9Y6M7-14;Q9Y6M7-10;Q9Y6M7-5;H7C3C4	3.79E+08	3.11E+08	3.78E+08	1.57E+08	1.17E+08	2.81E+08	2.75E+08	3.89E+08	4.54E+08	3.27E+08	3.77E+08	1.94E+07	3.45E+08	7.36E+08	2.63E+08	2.52E+08	3.16E+08	2.86E+08	3.47E+08	1.58E+08	1.03E+08	2.03E+08	4.61E-01
P40926;P40926-2;G3XAL0	2.08E+07	2.72E+07	2.28E+07	3.29E+07	4.62E+07	3.74E+07	0.00E+00	1.63E+07	2.76E+07	2.35E+07	4.78E+07	2.20E+07	4.09E+07	2.77E+07	2.36E+07	2.63E+07	2.77E+07	2.54E+07	2.99E+07	1.18E+07	1.41E+07	9.32E+06	4.64E-01
P08107;P08107-2;V9GZ37	0.00E+00	0.00E+00	0.00E+00	0.00E+00	0.00E+00	0.00E+00	7.99E+06	0.00E+00	1.01E+07	0.00E+00	0.00E+00	0.00E+00	0.00E+00	0.00E+00	8.95E+06	0.00E+00	1.69E+06	9.98E+05	2.39E+06	3.66E+06	2.82E+06	4.43E+06	4.67E-01
P14324-2;P14324;A0A087X1D8;A0A087WVN4	6.25E+08	4.94E+08	3.27E+08	4.41E+08	9.28E+08	4.71E+08	4.37E+08	6.24E+08	2.45E+08	6.59E+08	3.16E+08	3.15E+08	8.86E+08	5.98E+08	2.36E+08	4.66E+08	5.04E+08	5.43E+08	4.65E+08	2.07E+08	1.84E+08	2.33E+08	4.69E-01
Q5VTE0;P68104;P68104-2;A0A087WVQ9;A0A087WV01	2.52E+07	1.14E+07	1.57E+07	1.21E+07	5.47E+06	8.20E+06	0.00E+00	1.25E+07	2.75E+07	1.42E+07	0.00E+00	2.58E+07	9.96E+06	2.93E+07	0.00E+00	1.29E+07	1.31E+07	1.13E+07	1.50E+07	9.64E+06	7.43E+06	1.17E+07	4.70E-01
P07954-2;P07954	0.00E+00	0.00E+00	0.00E+00	1.06E+07	0.00E+00	1.16E+07	6.50E+06	1.40E+07	0.00E+00	0.00E+00	0.00E+00	0.00E+00	5.60E+06	1.36E+07	0.00E+00	6.97E+06	4.30E+06	5.33E+06	3.27E+06	5.49E+06	6.06E+06	5.05E+06	4.70E-01
Q16836;E9PF18;Q16836-3;Q16836-2;A0A0A0MSE2	0.00E+00	0.00E+00	0.00E+00	0.00E+00	0.00E+00	3.89E+06	0.00E+00	0.00E+00	0.00E+00	0.00E+00	0.00E+00	6.21E+06	0.00E+00	0.00E+00	3.35E+06	0.00E+00	8.40E+05	4.86E+05	1.19E+06	1.89E+06	1.37E+06	2.34E+06	4.72E-01

P21964-2;P21964;E7EMS6	6.79E+07	3.00E+07	3.50E+07	5.08E+07	4.10E+07	6.24E+07	4.59E+07	5.63E+07	7.02E+07	5.23E+07	0.00E+00	5.99E+07	4.16E+07	6.21E+07	0.00E+00	4.03E+07	4.47E+07	4.87E+07	4.08E+07	2.10E+07	1.32E+07	2.71E+07	4.74E-01
P50454;E9PR70;E9PPV6;E9PKH2;E9PK86;E9PMI5	0.00E+00	1.31E+07	1.82E+07	0.00E+00	0.00E+00	2.19E+07	2.35E+07	3.39E+07	0.00E+00	0.00E+00	0.00E+00	1.12E+07	2.37E+07	0.00E+00	2.10E+07	1.99E+07	1.16E+07	1.38E+07	9.47E+06	1.16E+07	1.28E+07	1.07E+07	4.75E-01
E7EQV9;E7ENU7;P61313;E7EX53	8.46E+06	5.90E+06	6.45E+06	0.00E+00	0.00E+00	0.00E+00	0.00E+00	0.00E+00	0.00E+00	0.00E+00	0.00E+00	1.02E+07	0.00E+00	0.00E+00	0.00E+00	0.00E+00	1.94E+06	2.60E+06	1.27E+06	3.57E+06	3.66E+06	3.60E+06	4.76E-01
K7EJR3;R4GMR5;P48556;K7EJC1	0.00E+00	0.00E+00	0.00E+00	4.19E+07	3.75E+07	0.00E+00	0.00E+00	0.00E+00	6.28E+07	2.51E+07	6.07E+07	0.00E+00	0.00E+00	0.00E+00	0.00E+00	0.00E+00	1.43E+07	9.93E+06	1.86E+07	2.33E+07	1.84E+07	2.80E+07	4.78E-01
D3DRR9;Q86WR7	0.00E+00	0.00E+00	0.00E+00	0.00E+00	0.00E+00	1.34E+07	0.00E+00	0.00E+00	0.00E+00	0.00E+00	0.00E+00	0.00E+00	0.00E+00	0.00E+00	1.77E+07	1.37E+07	2.81E+06	1.68E+06	3.93E+06	6.10E+06	4.75E+06	7.36E+06	4.78E-01
P25788-2;P25788	1.59E+07	0.00E+00	0.00E+00	2.49E+07	3.44E+07	1.15E+07	0.00E+00	0.00E+00	1.94E+07	2.12E+07	0.00E+00	0.00E+00	0.00E+00	0.00E+00	0.00E+00	1.27E+07	8.75E+06	1.08E+07	6.65E+06	1.14E+07	1.34E+07	9.49E+06	4.82E-01
Q9Y265;Q9Y265-2;H7C4G5;E7ETR0	1.30E+07	1.63E+07	2.02E+07	0.00E+00	1.18E+07	2.40E+07	0.00E+00	2.31E+07	1.09E+07	3.13E+06	8.79E+06	9.52E+06	1.22E+07	1.60E+07	1.25E+07	1.45E+07	1.22E+07	1.36E+07	1.09E+07	7.12E+06	9.44E+06	3.97E+06	4.83E-01
D6RFM5;P31040;P31040-2;P31040-3;A0A087X1I3	0.00E+00	0.00E+00	0.00E+00	6.62E+07	3.20E+07	3.96E+07	1.91E+07	0.00E+00	6.68E+07	3.56E+07	8.23E+07	1.05E+07	0.00E+00	4.38E+07	0.00E+00	0.00E+00	2.47E+07	1.96E+07	2.99E+07	2.84E+07	2.47E+07	3.25E+07	4.87E-01
P13647	0.00E+00	0.00E+00	0.00E+00	5.72E+06	4.03E+06	0.00E+00	0.00E+00	0.00E+00	0.00E+00	0.00E+00	0.00E+00	4.24E+06	0.00E+00	0.00E+00	0.00E+00	0.00E+00	8.74E+05	1.22E+06	5.30E+05	1.91E+06	2.30E+06	1.50E+06	4.90E-01
Q9BTV4	4.05E+07	1.05E+08	8.46E+07	6.17E+07	1.21E+08	9.51E+07	1.80E+08	1.05E+08	8.45E+07	1.35E+08	1.21E+08	4.90E+07	1.47E+08	6.46E+07	1.62E+08	1.49E+08	1.07E+08	9.92E+07	1.14E+08	4.14E+07	4.18E+07	4.25E+07	4.93E-01
P35579;P35579-2	1.84E+07	0.00E+00	0.00E+00	2.02E+07	1.18E+07	1.94E+07	1.78E+07	1.46E+07	1.43E+07	2.54E+07	0.00E+00	2.49E+07	2.34E+07	1.88E+07	0.00E+00	2.22E+07	1.44E+07	1.28E+07	1.61E+07	9.36E+06	8.33E+06	1.06E+07	4.93E-01
B4DY09;Q12905	1.36E+07	1.60E+07	0.00E+00	0.00E+00	0.00E+00	2.33E+07	1.79E+07	1.56E+07	0.00E+00	0.00E+00	0.00E+00	1.23E+07	2.05E+07	0.00E+00	1.50E+07	1.34E+07	9.23E+06	1.08E+07	7.66E+06	8.81E+06	9.38E+06	8.53E+06	4.94E-01
P28066;P28066-2	6.03E+06	6.38E+06	9.83E+06	0.00E+00	2.94E+06	8.60E+06	1.51E+07	1.34E+07	5.95E+06	9.55E+06	0.00E+00	2.59E+06	9.64E+06	8.61E+06	0.00E+00	1.23E+07	6.93E+06	7.79E+06	6.08E+06	4.81E+06	5.07E+06	4.72E+06	4.95E-01
Q9NTK5;J3KQ32;Q9NTK5-2	0.00E+00	0.00E+00	0.00E+00	1.41E+07	1.14E+07	1.44E+07	7.40E+06	8.68E+06	1.51E+07	1.12E+07	0.00E+00	1.54E+07	1.25E+07	0.00E+00	8.59E+06	1.03E+07	8.08E+06	7.00E+06	9.15E+06	6.07E+06	6.27E+06	6.09E+06	4.98E-01
Q9Y2S7;B4DEM9	6.28E+06	0.00E+00	6.64E+06	0.00E+00	5.98E+06	0.00E+00	0.00E+00	6.16E+06	1.82E+07	0.00E+00	0.00E+00	0.00E+00	7.87E+06	8.47E+06	0.00E+00	4.79E+06	4.02E+06	3.13E+06	4.91E+06	5.07E+06	3.35E+06	6.48E+06	5.01E-01
P32322-2;P32322;J3QL24;J3QKT4;J3KQ22;E2QRB3;P32322-3;J3QR88;J3QLK9;J3QL32	0.00E+00	0.00E+00	0.00E+00	6.93E+05	2.28E+06	0.00E+00	0.00E+00	0.00E+00	0.00E+00	0.00E+00	1.20E+06	0.00E+00	0.00E+00	0.00E+00	0.00E+00	0.00E+00	2.61E+05	3.72E+05	1.50E+05	6.35E+05	8.10E+05	4.23E+05	5.02E-01
Q9Y295	0.00E+00	0.00E+00	0.00E+00	0.00E+00	0.00E+00	0.00E+00	4.90E+06	0.00E+00	0.00E+00	0.00E+00	0.00E+00	0.00E+00	0.00E+00	0.00E+00	6.06E+06	4.80E+06	9.85E+05	6.12E+05	1.36E+06	2.13E+06	1.73E+06	2.54E+06	5.04E-01
O00194;K7ES41	0.00E+00	0.00E+00	0.00E+00	0.00E+00	0.00E+00	1.93E+06	0.00E+00	0.00E+00	0.00E+00	0.00E+00	0.00E+00	0.00E+00	1.35E+06	0.00E+00	0.00E+00	3.17E+06	4.03E+05	2.41E+05	5.65E+05	9.31E+05	6.82E+05	1.15E+06	5.05E-01
P10768;X6RA14;H7BZT7	0.00E+00	0.00E+00	0.00E+00	0.00E+00	0.00E+00	0.00E+00	6.31E+06	7.45E+06	0.00E+00	6.77E+06	0.00E+00	0.00E+00	4.08E+06	0.00E+00	6.01E+06	5.39E+06	2.25E+06	1.72E+06	2.78E+06	3.08E+06	3.20E+06	3.07E+06	5.09E-01
O95373	0.00E+00	0.00E+00	0.00E+00	0.00E+00	0.00E+00	7.61E+06	0.00E+00	5.36E+06	0.00E+00	0.00E+00	0.00E+00	0.00E+00	5.89E+06	0.00E+00	0.00E+00	0.00E+00	1.18E+06	1.62E+06	7.37E+05	2.57E+06	3.06E+06	2.08E+06	5.10E-01
J3QLR8;Q9Y3D9	0.00E+00	1.88E+07	0.00E+00	0.00E+00	0.00E+00	0.00E+00	1.41E+07	1.05E+07	1.10E+07	0.00E+00	0.00E+00	0.00E+00	0.00E+00	0.00E+00	1.41E+07	0.00E+00	4.28E+06	5.43E+06	3.13E+06	6.78E+06	7.82E+06	5.85E+06	5.15E-01
P11387	0.00E+00	1.69E+07	0.00E+00	0.00E+00	0.00E+00	1.29E+07	1.18E+07	6.56E+06	0.00E+00	0.00E+00	0.00E+00	1.63E+07	6.79E+06	0.00E+00	0.00E+00	7.76E+06	4.94E+06	6.02E+06	3.85E+06	6.40E+06	7.01E+06	6.00E+06	5.17E-01
H3BUX2;O43169;J3KNF8;D6RFH4	0.00E+00	0.00E+00	0.00E+00	0.00E+00	0.00E+00	0.00E+00	4.53E+06	5.67E+06	0.00E+00	0.00E+00	0.00E+00	0.00E+00	4.77E+06	0.00E+00	0.00E+00	0.00E+00	9.36E+05	1.27E+06	5.97E+05	2.02E+06	2.38E+06	1.69E+06	5.22E-01
P30085;Q5T0D2	0.00E+00	2.64E+07	0.00E+00	0.00E+00	4.93E+07	0.00E+00	2.59E+07	2.41E+07	0.00E+00	1.80E+07	1.52E+07	0.00E+00	1.16E+07	1.23E+07	1.53E+07	1.69E+07	1.34E+07	1.57E+07	1.12E+07	1.38E+07	1.85E+07	7.20E+06	5.27E-01
P11586;F5H2F4	4.51E+07	2.21E+07	1.54E+07	0.00E+00	0.00E+00	5.11E+06	0.00E+00	1.29E+07	1.47E+07	0.00E+00	0.00E+00	0.00E+00	1.42E+07	2.40E+07	0.00E+00	1.44E+07	1.05E+07	1.26E+07	8.42E+06	1.26E+07	1.55E+07	9.53E+06	5.29E-01
Q07955-3;Q07955;J3KTL2;Q07955-2	0.00E+00	0.00E+00	0.00E+00	0.00E+00	0.00E+00	0.00E+00	6.20E+06	6.97E+06	0.00E+00	0.00E+00	0.00E+00	0.00E+00	0.00E+00	1.42E+07	0.00E+00	1.11E+07	2.40E+06	1.65E+06	3.16E+06	4.61E+06	3.06E+06	5.91E+06	5.30E-01
P49756	5.51E+06	3.98E+06	0.00E+00	3.48E+06	0.00E+00	0.00E+00	8.53E+06	7.99E+06	0.00E+00	0.00E+00	0.00E+00	0.00E+00	6.55E+06	5.64E+06	0.00E+00	8.18E+06	3.12E+06	3.69E+06	2.55E+06	3.47E+06	3.51E+06	3.58E+06	5.30E-01
Q53GQ0;E9PI21	1.99E+07	2.03E+07	2.60E+07	0.00E+00	0.00E+00	0.00E+00	0.00E+00	0.00E+00	2.50E+07	1.78E+07	1.47E+07	0.00E+00	0.00E+00	1.90E+07	0.00E+00	1.79E+07	1.00E+07	8.28E+06	1.18E+07	1.07E+07	1.16E+07	1.02E+07	5.30E-01
P33993;P33993-3;P33993-2	0.00E+00	0.00E+00	0.00E+00	0.00E+00	1.54E+07	1.09E+07	1.00E+07	0.00E+00	0.00E+00	1.75E+07	1.14E+07	0.00E+00	8.73E+06	0.00E+00	1.72E+07	0.00E+00	5.70E+06	4.54E+06	6.85E+06	7.05E+06	6.46E+06	7.86E+06	5.31E-01
Q9UNM6;A0A087WUL9;Q9UNM6-2;J3KNQ3;H0YD73;E9PL38	0.00E+00	0.00E+00	1.04E+07	1.14E+07	1.72E+07	1.12E+07	0.00E+00	1.18E+07	1.02E+07	0.00E+00	0.00E+00	0.00E+00	1.54E+07	9.00E+06	0.00E+00	1.09E+07	6.72E+06	7.75E+06	5.68E+06	6.41E+06	6.74E+06	6.35E+06	5.38E-01
Q15181;Q5SQT6	4.33E+07	6.27E+07	8.85E+07	4.53E+07	6.82E+07	6.40E+07	1.23E+08	9.70E+07	3.71E+07	4.50E+07	8.68E+07	4.29E+07	6.69E+07	5.41E+07	9.58E+07	9.84E+07	7.00E+07	7.40E+07	6.59E+07	2.55E+07	2.73E+07	2.49E+07	5.41E-01
Q5JP53;P07437;Q5ST81	0.00E+00	0.00E+00	0.00E+00	0.00E+00	0.00E+00	0.00E+00	2.99E+07	2.11E+07	0.00E+00	2.32E+07	1.69E+07	0.00E+00	0.00E+00	0.00E+00	1.95E+07	1.99E+07	8.15E+06	6.37E+06	9.94E+06	1.12E+07	1.20E+07	1.08E+07	5.41E-01
Q99613;Q99613-2;B5ME19	0.00E+00	0.00E+00	0.00E+00	1.30E+07	0.00E+00	0.00E+00	0.00E+00	1.03E+07	0.00E+00	1.25E+07	2.00E+07	0.00E+00	0.00E+00	0.00E+00	7.24E+06	0.00E+00	3.94E+06	2.92E+06	4.97E+06	6.51E+06	5.44E+06	7.67E+06	5.47E-01

Q5SZE2;Q5SZE3;H0YKH6;Q5SZE4;Q5SZE1;Q96G23;H0YNU7	0.00E+00	0.00E+00	1.95E+06	0.00E+00	2.04E+06	0.00E+00	0.00E+00	0.00E+00	0.00E+00	0.00E+00	0.00E+00	2.56E+06	2.22E+06	0.00E+00	1.78E+06	0.00E+00	6.59E+05	4.98E+05	8.20E+05	1.02E+06	9.23E+05	1.15E+06	5.47E-01
P51398-2;P51398-3;P51398;V9GZ03;V9GYJ3;V9GY11;V9GYJ9;V9GYL9	0.00E+00	0.00E+00	0.00E+00	0.00E+00	0.00E+00	0.00E+00	1.76E+07	1.55E+07	0.00E+00	0.00E+00	0.00E+00	1.65E+07	0.00E+00	0.00E+00	0.00E+00	0.00E+00	3.10E+06	4.14E+06	2.07E+06	6.68E+06	7.69E+06	5.84E+06	5.53E-01
P30086	1.69E+07	9.61E+06	0.00E+00	1.16E+07	1.43E+07	1.39E+07	1.02E+07	1.75E+07	1.80E+07	1.89E+07	0.00E+00	8.43E+06	6.87E+06	1.35E+07	0.00E+00	1.26E+07	1.08E+07	1.17E+07	9.78E+06	6.35E+06	5.55E+06	7.30E+06	5.54E-01
P52597	0.00E+00	0.00E+00	0.00E+00	0.00E+00	7.96E+06	0.00E+00	0.00E+00	0.00E+00	0.00E+00	6.63E+06	9.31E+06	0.00E+00	0.00E+00	0.00E+00	0.00E+00	0.00E+00	1.49E+06	9.95E+05	1.99E+06	3.25E+06	2.81E+06	3.76E+06	5.57E-01
Q8IVF2-3;Q8IVF2	0.00E+00	0.00E+00	0.00E+00	0.00E+00	0.00E+00	1.43E+08	1.55E+08	0.00E+00	0.00E+00	0.00E+00	0.00E+00	0.00E+00	0.00E+00	6.60E+07	0.00E+00	9.82E+07	2.88E+07	3.72E+07	2.05E+07	5.47E+07	6.89E+07	3.90E+07	5.61E-01
C9JRR5;Q3SYG4-5;F8WCG5;Q3SYG4-6;Q3SYG4-2;Q3SYG4-4;Q3SYG4-7;Q3SYG4-3;Q3SYG4	5.95E+07	6.38E+07	5.66E+07	7.21E+07	4.72E+07	4.56E+07	0.00E+00	4.66E+07	5.99E+07	5.59E+07	3.68E+07	4.97E+07	2.53E+07	4.40E+07	4.29E+07	3.56E+07	4.63E+07	4.89E+07	4.38E+07	1.70E+07	2.19E+07	1.13E+07	5.63E-01
Q5T6W5;P61978-3;P61978;P61978-2;Q5T6W2;Q5T6W1	2.31E+07	2.27E+07	2.35E+07	1.53E+07	1.78E+07	1.30E+07	0.00E+00	0.00E+00	0.00E+00	2.22E+07	1.41E+07	2.70E+07	0.00E+00	0.00E+00	1.34E+07	1.58E+07	1.30E+07	1.44E+07	1.16E+07	9.89E+06	9.68E+06	1.05E+07	5.80E-01
Q01105-3;Q01105-4;Q01105-2;Q01105;A0A087X027;P0DME0	5.43E+08	3.51E+08	3.75E+08	3.82E+08	3.91E+08	6.55E+06	3.06E+08	3.62E+08	3.31E+08	3.41E+08	3.55E+08	4.31E+08	3.53E+08	4.20E+08	3.67E+08	3.67E+08	3.55E+08	3.40E+08	3.71E+08	1.07E+08	1.51E+08	3.60E+07	5.80E-01
P42704	2.84E+08	2.72E+08	1.91E+08	2.78E+08	2.88E+08	2.63E+08	1.84E+08	1.84E+08	2.62E+08	3.05E+08	2.83E+08	0.00E+00	2.21E+08	2.62E+08	1.85E+08	2.56E+08	2.32E+08	2.43E+08	2.22E+08	7.45E+07	4.75E+07	9.68E+07	5.86E-01
P21796;C9JI87	2.22E+07	3.94E+07	3.21E+07	4.16E+07	2.92E+07	7.35E+07	6.23E+07	4.88E+07	3.20E+07	4.00E+07	3.92E+07	2.53E+07	5.41E+07	0.00E+00	6.56E+07	5.11E+07	4.10E+07	4.36E+07	3.84E+07	1.83E+07	1.73E+07	2.01E+07	5.87E-01
P26373;J3QSB4;P26373-2	0.00E+00	0.00E+00	0.00E+00	4.47E+06	0.00E+00	6.33E+06	0.00E+00	0.00E+00	3.02E+06	3.20E+06	0.00E+00	0.00E+00	0.00E+00	0.00E+00	0.00E+00	0.00E+00	1.06E+06	1.35E+06	7.78E+05	2.02E+06	2.55E+06	1.44E+06	5.90E-01
P43304-2;P43304	8.87E+07	5.00E+07	8.55E+07	0.00E+00	5.93E+07	6.41E+07	4.53E+07	4.68E+07	5.87E+07	4.18E+07	5.94E+07	4.57E+07	7.29E+07	0.00E+00	5.74E+07	4.91E+07	5.15E+07	5.50E+07	4.81E+07	2.43E+07	2.77E+07	2.17E+07	5.92E-01
P12236;I7HJJ0;P12235	7.07E+06	0.00E+00	9.64E+06	0.00E+00	2.05E+07	4.76E+06	1.19E+07	1.96E+07	0.00E+00	0.00E+00	1.12E+07	0.00E+00	2.93E+07	2.13E+07	1.49E+07	1.79E+07	1.05E+07	9.18E+06	1.18E+07	9.40E+06	7.90E+06	1.11E+07	5.92E-01
P08758;D6RBL5;D6RBE9	0.00E+00	0.00E+00	0.00E+00	0.00E+00	0.00E+00	0.00E+00	3.96E+06	0.00E+00	0.00E+00	0.00E+00	0.00E+00	0.00E+00	0.00E+00	0.00E+00	9.67E+06	0.00E+00	8.52E+05	4.95E+05	1.21E+06	2.55E+06	1.40E+06	3.42E+06	5.93E-01
P51858-2;P51858;P51858-3	0.00E+00	0.00E+00	0.00E+00	1.55E+07	1.09E+07	3.91E+06	0.00E+00	0.00E+00	0.00E+00	0.00E+00	1.39E+07	4.31E+06	0.00E+00	0.00E+00	0.00E+00	0.00E+00	3.03E+06	3.78E+06	2.28E+06	5.41E+06	6.09E+06	4.93E+06	5.95E-01
P08779	0.00E+00	0.00E+00	1.14E+07	0.00E+00	8.27E+06	0.00E+00	0.00E+00	8.60E+06	0.00E+00	8.93E+06	9.29E+06	0.00E+00	0.00E+00	0.00E+00	0.00E+00	0.00E+00	2.90E+06	3.53E+06	2.28E+06	4.49E+06	4.95E+06	4.22E+06	5.95E-01
P46977;P46977-2	2.22E+08	1.94E+08	1.47E+08	3.45E+08	2.61E+08	3.97E+08	1.80E+08	9.67E+07	2.19E+08	2.61E+08	2.17E+08	1.92E+08	3.49E+08	2.38E+08	2.92E+08	2.46E+08	2.41E+08	2.30E+08	2.52E+08	7.72E+07	1.00E+08	4.96E+07	5.97E-01
P02786;G3V0E5	7.35E+06	3.04E+07	3.97E+07	2.05E+07	3.44E+07	4.19E+07	0.00E+00	4.87E+07	2.03E+07	2.27E+07	3.23E+07	1.25E+07	4.06E+07	2.89E+07	5.00E+07	4.92E+07	3.00E+07	2.79E+07	3.21E+07	1.52E+07	1.72E+07	1.37E+07	5.97E-01
P08133;P08133-2	0.00E+00	0.00E+00	1.60E+07	0.00E+00	0.00E+00	0.00E+00	1.35E+07	2.42E+07	0.00E+00	0.00E+00	0.00E+00	0.00E+00	1.32E+07	0.00E+00	1.47E+07	8.12E+06	5.61E+06	6.72E+06	4.51E+06	8.08E+06	9.74E+06	6.49E+06	6.01E-01
Q99497;K7ELW0;K7EN27	3.22E+07	2.89E+07	4.46E+07	0.00E+00	0.00E+00	1.80E+07	0.00E+00	2.14E+07	0.00E+00	0.00E+00	0.00E+00	2.36E+07	3.10E+07	0.00E+00	3.33E+07	2.29E+07	1.60E+07	1.81E+07	1.38E+07	1.57E+07	1.69E+07	1.52E+07	6.01E-01
P62701;A6NH36	1.55E+09	1.55E+09	1.51E+09	1.35E+09	1.51E+09	1.63E+07	1.35E+09	1.24E+09	6.92E+08	1.37E+09	1.41E+09	1.12E+09	1.35E+09	8.22E+08	1.37E+09	1.07E+09	1.20E+09	1.26E+09	1.15E+09	4.02E+08	5.15E+08	2.74E+08	6.03E-01
P08238	0.00E+00	0.00E+00	0.00E+00	0.00E+00	0.00E+00	1.07E+07	5.40E+06	0.00E+00	0.00E+00	0.00E+00	0.00E+00	1.43E+07	0.00E+00	0.00E+00	0.00E+00	1.29E+07	2.70E+06	2.01E+06	3.39E+06	5.14E+06	3.98E+06	6.30E+06	6.07E-01
C9JSP1;C9JQ75;C9J0M4;P51572;P51572-2;C9JMD7	3.52E+07	4.25E+07	2.54E+07	0.00E+00	0.00E+00	7.01E+07	7.13E+07	7.37E+07	0.00E+00	0.00E+00	0.00E+00	4.87E+07	5.24E+07	3.42E+07	4.96E+07	7.14E+07	3.59E+07	3.98E+07	3.20E+07	2.87E+07	3.04E+07	2.84E+07	6.07E-01
P30084	0.00E+00	3.35E+07	0.00E+00	6.85E+07	2.24E+07	0.00E+00	6.93E+07	2.33E+07	5.22E+07	2.84E+07	3.43E+07	1.32E+07	2.95E+07	4.15E+07	3.46E+07	2.89E+07	3.00E+07	2.71E+07	3.28E+07	2.13E+07	2.87E+07	1.13E+07	6.09E-01
P19367-4;P19367-2;P19367;P19367-3	7.92E+06	0.00E+00	0.00E+00	1.40E+07	1.42E+07	1.37E+07	0.00E+00	1.32E+07	0.00E+00	0.00E+00	0.00E+00	0.00E+00	1.17E+07	1.98E+07	0.00E+00	1.56E+07	6.88E+06	7.87E+06	5.88E+06	7.46E+06	6.82E+06	8.40E+06	6.10E-01
P00387-2;P00387;P00387-3;B1AHF3	0.00E+00	0.00E+00	0.00E+00	0.00E+00	0.00E+00	1.51E+07	2.19E+07	8.80E+06	0.00E+00	0.00E+00	0.00E+00	4.72E+07	1.13E+07	0.00E+00	0.00E+00	1.47E+07	7.44E+06	5.73E+06	9.16E+06	1.28E+07	8.65E+06	1.65E+07	6.11E-01
A0A087X2D0;P84103-2;P84103	6.29E+08	5.08E+08	5.31E+08	5.95E+08	4.83E+08	5.10E+08	3.91E+08	4.47E+08	5.83E+08	5.66E+08	7.60E+08	4.81E+08	5.64E+08	3.96E+08	4.65E+08	4.77E+08	5.24E+08	5.12E+08	5.36E+08	9.24E+07	7.61E+07	1.10E+08	6.11E-01
P05141	1.57E+08	1.67E+08	1.06E+08	1.33E+08	2.96E+08	3.56E+08	5.25E+08	4.48E+08	1.53E+08	1.57E+08	1.61E+08	1.98E+08	2.73E+08	8.78E+07	4.42E+08	4.21E+08	2.55E+08	2.74E+08	2.37E+08	1.41E+08	1.58E+08	1.31E+08	6.19E-01
P68371	3.57E+07	0.00E+00	2.21E+07	3.20E+07	3.63E+07	3.96E+07	5.53E+07	3.32E+07	2.72E+07	3.67E+07	4.87E+07	4.28E+07	3.55E+07	0.00E+00	5.56E+07	4.09E+07	3.39E+07	3.18E+07	3.59E+07	1.59E+07	1.58E+07	1.69E+07	6.20E-01

P06744;P06744-2;A0A0A0MTS2;K7EQ48	0.00E+00	2.10E+07	0.00E+00	1.04E+07	1.71E+07	0.00E+00	1.30E+07	1.64E+07	0.00E+00	0.00E+00	0.00E+00	2.42E+07	2.55E+07	0.00E+00	2.51E+07	2.59E+07	1.12E+07	9.74E+06	1.26E+07	1.10E+07	8.63E+06	1.35E+07	6.22E-01
Q15365	5.06E+07	3.61E+07	4.64E+07	7.16E+07	4.33E+07	8.31E+07	0.00E+00	6.33E+07	4.02E+07	4.77E+07	6.25E+07	3.35E+07	4.43E+07	3.57E+07	4.10E+07	5.13E+07	4.69E+07	4.93E+07	4.45E+07	1.86E+07	2.53E+07	9.34E+06	6.23E-01
P04843;B7Z4L4	3.18E+07	3.26E+07	2.92E+07	1.76E+07	1.51E+07	0.00E+00	3.39E+07	3.15E+07	1.48E+07	2.98E+07	2.61E+07	1.84E+07	2.58E+07	2.99E+07	3.27E+07	3.36E+07	2.52E+07	2.40E+07	2.64E+07	9.48E+06	1.20E+07	6.68E+06	6.27E-01
Q9BSJ8;Q9BSJ8-2	2.03E+08	2.68E+08	4.32E+08	0.00E+00	0.00E+00	2.22E+08	5.06E+08	4.67E+08	0.00E+00	2.58E+08	1.19E+08	3.49E+08	3.69E+08	5.52E+07	2.67E+08	3.40E+08	2.41E+08	2.62E+08	2.20E+08	1.68E+08	1.97E+08	1.43E+08	6.30E-01
Q06830;A0A0A0MSI0	7.99E+06	0.00E+00	0.00E+00	0.00E+00	0.00E+00	0.00E+00	0.00E+00	0.00E+00	0.00E+00	0.00E+00	0.00E+00	1.73E+07	0.00E+00	0.00E+00	0.00E+00	0.00E+00	1.58E+06	9.98E+05	2.16E+06	4.65E+06	2.82E+06	6.12E+06	6.32E-01
Q9NUJ1;Q9NUJ1-2;Q9NUJ1-3	1.49E+07	3.80E+07	1.56E+07	2.13E+07	3.86E+07	1.80E+07	6.36E+07	3.87E+07	3.75E+07	4.88E+07	3.42E+07	0.00E+00	4.70E+07	2.84E+07	3.54E+07	4.93E+07	3.31E+07	3.11E+07	3.51E+07	1.61E+07	1.68E+07	1.61E+07	6.35E-01
Q04637-5;E7EX73;Q04637-4;E9PGM1;Q04637-3;E7EUU4;Q04637;Q04637-8;Q04637-9;Q04637-6;Q04637-7;A0A0A0MR52;C9JF13	4.77E+07	3.81E+07	4.50E+07	2.40E+07	3.56E+07	0.00E+00	2.86E+07	3.71E+07	3.37E+07	1.53E+07	2.01E+07	3.95E+07	3.84E+07	2.83E+07	2.91E+07	2.81E+07	3.05E+07	3.20E+07	2.91E+07	1.19E+07	1.51E+07	8.36E+06	6.39E-01
Q9BQG0;Q9BQG0-2;I3L1L3	1.96E+07	1.06E+07	1.08E+07	6.07E+06	0.00E+00	2.33E+07	7.15E+06	1.40E+07	1.56E+07	1.72E+07	0.00E+00	1.85E+07	7.72E+06	0.00E+00	9.86E+06	8.69E+06	1.06E+07	1.15E+07	9.69E+06	7.16E+06	7.51E+06	7.18E+06	6.39E-01
P07686;Q5URX0;H0YA83;H0Y9B6	2.68E+07	1.92E+07	3.20E+07	4.20E+07	3.24E+07	0.00E+00	1.35E+07	2.12E+07	4.73E+07	2.83E+07	1.61E+07	2.05E+07	2.27E+07	2.80E+07	2.71E+07	1.89E+07	2.48E+07	2.34E+07	2.61E+07	1.11E+07	1.30E+07	9.66E+06	6.41E-01
Q9NYU2-2;Q9NYU2	0.00E+00	0.00E+00	0.00E+00	1.06E+07	0.00E+00	1.15E+07	0.00E+00	1.76E+07	0.00E+00	0.00E+00	1.33E+07	0.00E+00	0.00E+00	2.86E+07	0.00E+00	1.51E+07	6.05E+06	4.97E+06	7.13E+06	8.92E+06	7.14E+06	1.08E+07	6.44E-01
M0R0G9;M0QXK2;M0R221;M0R2B8;M0R268;P09012;M0QZG7;P08579	0.00E+00	0.00E+00	0.00E+00	0.00E+00	5.68E+06	0.00E+00	0.00E+00	0.00E+00	2.73E+06	0.00E+00	0.00E+00	0.00E+00	0.00E+00	0.00E+00	0.00E+00	0.00E+00	5.25E+05	7.10E+05	3.41E+05	1.53E+06	2.01E+06	9.65E+05	6.47E-01
C9JRH2;C9JMJ4;C9J3R0;C9JQZ4;C9JW69;P18754;P18754-2	0.00E+00	0.00E+00	0.00E+00	0.00E+00	0.00E+00	1.80E+07	1.18E+07	2.35E+07	0.00E+00	0.00E+00	0.00E+00	0.00E+00	1.72E+07	0.00E+00	3.18E+07	2.62E+07	8.03E+06	6.66E+06	9.40E+06	1.15E+07	9.71E+06	1.36E+07	6.49E-01
P24539;Q5QNZ2	0.00E+00	0.00E+00	1.34E+07	0.00E+00	0.00E+00	1.02E+07	9.88E+06	7.25E+06	0.00E+00	0.00E+00	0.00E+00	8.88E+06	1.06E+07	0.00E+00	0.00E+00	1.10E+07	4.45E+06	5.09E+06	3.81E+06	5.35E+06	5.68E+06	5.30E+06	6.50E-01
P25787	0.00E+00	0.00E+00	0.00E+00	0.00E+00	0.00E+00	1.10E+07	0.00E+00	0.00E+00	0.00E+00	0.00E+00	0.00E+00	0.00E+00	0.00E+00	0.00E+00	9.19E+06	9.35E+06	1.84E+06	1.37E+06	2.32E+06	3.98E+06	3.88E+06	4.29E+06	6.51E-01
P49257	0.00E+00	0.00E+00	0.00E+00	1.22E+07	0.00E+00	1.28E+07	0.00E+00	1.15E+07	0.00E+00	0.00E+00	0.00E+00	0.00E+00	0.00E+00	1.61E+07	0.00E+00	9.11E+06	3.86E+06	4.57E+06	3.15E+06	6.05E+06	6.31E+06	6.13E+06	6.56E-01
P35613-3;A0A087WUV8;P35613-4;P35613-2;P35613;A0A087X2B5	7.43E+06	0.00E+00	1.08E+07	2.54E+07	8.50E+06	0.00E+00	7.31E+06	4.17E+06	1.28E+07	7.10E+06	7.49E+06	1.26E+07	0.00E+00	0.00E+00	5.46E+06	6.03E+06	7.19E+06	7.94E+06	6.44E+06	6.47E+06	8.05E+06	4.85E+06	6.58E-01
P11177-3;P11177-2;P11177;C9J634	1.56E+07	0.00E+00	1.24E+07	1.42E+07	0.00E+00	9.68E+06	0.00E+00	1.17E+07	1.33E+07	8.45E+06	1.04E+07	1.94E+07	0.00E+00	1.75E+07	7.28E+06	0.00E+00	8.75E+06	7.95E+06	9.54E+06	6.82E+06	6.81E+06	7.21E+06	6.58E-01
Q16822;B4DW73;H0YML5	7.78E+07	6.66E+07	4.85E+07	6.51E+07	6.10E+07	8.20E+07	7.65E+07	8.36E+07	7.55E+07	1.00E+08	5.19E+07	0.00E+00	1.00E+08	1.22E+08	7.14E+07	8.92E+07	7.32E+07	7.01E+07	7.63E+07	2.71E+07	1.20E+07	3.74E+07	6.66E-01
Q96AG4	2.14E+07	3.47E+07	0.00E+00	4.43E+07	4.12E+07	3.13E+07	3.47E+07	2.88E+07	3.54E+07	4.77E+07	0.00E+00	4.45E+07	5.89E+07	4.59E+07	0.00E+00	3.61E+07	3.16E+07	2.96E+07	3.36E+07	1.79E+07	1.39E+07	2.20E+07	6.68E-01
P36578;H3BM89	0.00E+00	2.47E+07	0.00E+00	1.07E+07	1.55E+07	0.00E+00	2.73E+07	1.96E+07	9.28E+06	1.99E+07	1.03E+07	9.54E+06	2.26E+07	0.00E+00	2.05E+07	2.28E+07	1.33E+07	1.22E+07	1.44E+07	9.64E+06	1.13E+07	8.27E+06	6.70E-01
P55060-3;P55060;P55060-4	0.00E+00	7.16E+06	0.00E+00	0.00E+00	0.00E+00	0.00E+00	6.41E+06	4.41E+06	0.00E+00	0.00E+00	0.00E+00	0.00E+00	0.00E+00	0.00E+00	7.35E+06	5.31E+06	1.92E+06	2.25E+06	1.58E+06	3.00E+06	3.19E+06	2.98E+06	6.73E-01
Q8TEX9;Q8TEX9-2;H0YN14	0.00E+00	7.47E+05	0.00E+00	0.00E+00	0.00E+00	0.00E+00	0.00E+00	0.00E+00	0.00E+00	0.00E+00	0.00E+00	0.00E+00	3.86E+05	0.00E+00	0.00E+00	0.00E+00	7.08E+04	9.34E+04	4.82E+04	2.04E+05	2.64E+05	1.36E+05	6.74E-01
M0QZS6;Q9UBE0	1.13E+07	0.00E+00	0.00E+00	0.00E+00	0.00E+00	7.73E+06	0.00E+00	0.00E+00	0.00E+00	0.00E+00	0.00E+00	4.06E+06	0.00E+00	0.00E+00	0.00E+00	8.33E+06	1.96E+06	2.38E+06	1.55E+06	3.75E+06	4.50E+06	3.09E+06	6.75E-01
Q07666-3;Q07666-2;Q07666	4.60E+06	0.00E+00	0.00E+00	4.38E+06	5.60E+06	0.00E+00	0.00E+00	0.00E+00	0.00E+00	0.00E+00	0.00E+00	1.56E+07	6.45E+06	0.00E+00	0.00E+00	0.00E+00	2.29E+06	1.82E+06	2.76E+06	4.27E+06	2.54E+06	5.67E+06	6.76E-01
O75521-2;O75521;F8WAW4;F1LLU7;C9JB63	9.36E+06	0.00E+00	0.00E+00	0.00E+00	0.00E+00	6.44E+06	8.58E+06	0.00E+00	1.46E+07	0.00E+00	0.00E+00	0.00E+00	0.00E+00	0.00E+00	9.29E+06	9.29E+06	3.60E+06	3.05E+06	4.15E+06	5.04E+06	4.28E+06	5.95E+06	6.78E-01
P54709;C9JXZ1;C9JA36;P54709-2	1.80E+07	2.29E+07	2.74E+07	4.18E+07	4.66E+07	8.78E+06	1.56E+07	2.00E+07	2.94E+07	3.42E+07	3.31E+07	1.94E+07	2.37E+07	0.00E+00	2.01E+07	2.12E+07	2.39E+07	2.52E+07	2.26E+07	1.16E+07	1.30E+07	1.08E+07	6.79E-01

P50990;P50990-2;P50990-3	0.00E+00	0.00E+00	0.00E+00	0.00E+00	0.00E+00	0.00E+00	9.06E+06	4.53E+06	0.00E+00	0.00E+00	0.00E+00	0.00E+00	5.58E+06	0.00E+00	6.02E+06	7.64E+06	2.05E+06	1.70E+06	2.41E+06	3.28E+06	3.37E+06	3.37E+06	6.82E-01
P04632;K7ELJ7;K7EM73;K7EIV0;A0A075B7C0;U3KQE2;K7EKD8	8.05E+06	0.00E+00	0.00E+00	0.00E+00	0.00E+00	0.00E+00	5.59E+06	4.65E+06	0.00E+00	0.00E+00	0.00E+00	0.00E+00	0.00E+00	7.47E+06	7.94E+06	9.26E+06	2.68E+06	2.29E+06	3.08E+06	3.71E+06	3.29E+06	4.28E+06	6.82E-01
Q93009-3;Q93009;H3BND8	0.00E+00	2.50E+07	5.03E+07	3.36E+07	2.48E+07	3.71E+07	2.73E+07	1.56E+07	0.00E+00	4.48E+07	0.00E+00	3.94E+07	3.10E+07	1.58E+07	3.04E+07	2.59E+07	2.51E+07	2.67E+07	2.34E+07	1.54E+07	1.49E+07	1.68E+07	6.84E-01
H0YA96;H0Y8G5;Q14103-4;Q14103-3;Q14103-2;Q14103;D6RF44;D6RAF8;D6RBQ9	0.00E+00	0.00E+00	0.00E+00	0.00E+00	0.00E+00	0.00E+00	0.00E+00	5.27E+06	0.00E+00	0.00E+00	0.00E+00	0.00E+00	3.70E+06	0.00E+00	2.74E+06	1.62E+06	8.33E+05	6.58E+05	1.01E+06	1.64E+06	1.86E+06	1.50E+06	6.85E-01
Q96AY3	0.00E+00	0.00E+00	1.67E+07	1.63E+07	1.93E+07	1.03E+07	1.14E+07	1.37E+07	1.96E+07	1.68E+07	1.40E+07	0.00E+00	1.04E+07	1.63E+07	7.39E+06	1.45E+07	1.17E+07	1.09E+07	1.24E+07	6.64E+06	7.35E+06	6.28E+06	6.87E-01
Q12907;D6RBV2;D6RIU4	4.66E+08	2.79E+08	4.58E+08	6.11E+08	4.78E+08	6.94E+08	3.82E+08	4.22E+08	3.76E+08	5.37E+08	4.14E+08	5.16E+08	5.11E+08	6.61E+06	5.90E+08	5.74E+08	4.57E+08	4.74E+08	4.41E+08	1.58E+08	1.29E+08	1.90E+08	6.89E-01
P07355;P07355-2;H0YN42;H0YMD0;H0YMU9;A6NMY6;H0YM50;H0YKS4;H0YNP5;H0YN28;H0YL33	0.00E+00	4.58E+06	5.11E+06	0.00E+00	0.00E+00	0.00E+00	3.87E+06	5.28E+06	0.00E+00	5.57E+06	3.79E+06	0.00E+00	3.99E+06	0.00E+00	5.48E+06	4.04E+06	2.61E+06	2.35E+06	2.86E+06	2.43E+06	2.55E+06	2.46E+06	6.93E-01
P25685;P25685-2;M0R080	1.34E+08	1.33E+08	1.15E+08	1.37E+08	1.07E+08	1.36E+08	8.47E+07	1.06E+08	2.24E+08	1.59E+08	1.97E+08	0.00E+00	9.65E+07	1.78E+08	8.10E+07	1.04E+08	1.25E+08	1.19E+08	1.30E+08	5.20E+07	1.90E+07	7.33E+07	6.94E-01
J3KPX7;Q99623;Q99623-2;F5GY37;F5H3X6;F5GWA7	0.00E+00	0.00E+00	0.00E+00	2.06E+07	0.00E+00	2.50E+07	2.83E+07	1.90E+07	0.00E+00	2.89E+07	0.00E+00	2.96E+07	0.00E+00	2.48E+07	0.00E+00	3.21E+07	1.30E+07	1.16E+07	1.44E+07	1.38E+07	1.27E+07	1.56E+07	6.97E-01
H3BSJ9;H3BRG4;P22695;H3BP04	0.00E+00	0.00E+00	0.00E+00	0.00E+00	3.33E+06	7.20E+06	4.58E+06	4.31E+06	2.94E+06	3.71E+06	1.33E+06	0.00E+00	0.00E+00	0.00E+00	9.78E+06	6.74E+06	2.74E+06	2.43E+06	3.06E+06	3.13E+06	2.81E+06	3.59E+06	7.00E-01
Q96CS3	2.25E+07	1.90E+07	2.03E+07	3.03E+07	2.38E+07	1.98E+07	0.00E+00	1.64E+07	1.35E+07	2.07E+07	3.08E+07	2.43E+07	1.74E+07	2.40E+07	1.08E+07	2.28E+07	1.98E+07	1.90E+07	2.05E+07	7.45E+06	8.74E+06	6.43E+06	7.00E-01
Q9NVI7-2;Q9NVI7;Q9NVI7-3;H0Y2W2	0.00E+00	0.00E+00	0.00E+00	0.00E+00	0.00E+00	0.00E+00	5.40E+06	4.27E+06	0.00E+00	5.51E+06	0.00E+00	0.00E+00	0.00E+00	0.00E+00	0.00E+00	8.64E+06	1.49E+06	1.21E+06	1.77E+06	2.79E+06	2.26E+06	3.38E+06	7.02E-01
P42285	1.91E+06	0.00E+00	1.12E+06	0.00E+00	0.00E+00	1.09E+06	1.52E+06	0.00E+00	7.67E+05	0.00E+00	0.00E+00	0.00E+00	0.00E+00	2.66E+06	0.00E+00	8.63E+05	6.21E+05	7.05E+05	5.36E+05	8.43E+05	7.95E+05	9.35E+05	7.03E-01
P43686-2;P43686	1.46E+08	1.18E+08	1.47E+08	1.68E+08	1.48E+08	2.07E+08	1.54E+08	1.44E+08	1.47E+08	1.28E+08	1.44E+08	1.58E+08	1.50E+08	1.61E+08	1.56E+08	1.57E+08	1.52E+08	1.54E+08	1.50E+08	1.91E+07	2.57E+07	1.07E+07	7.04E-01
Q9NZM1-6;Q9NZM1;Q9NZM1-3;Q9NZM1-2;Q9NZM1-5	2.54E+07	3.16E+07	0.00E+00	0.00E+00	0.00E+00	2.99E+07	2.88E+07	1.39E+07	0.00E+00	0.00E+00	0.00E+00	3.15E+07	2.32E+07	0.00E+00	2.78E+07	2.46E+07	1.48E+07	1.62E+07	1.34E+07	1.41E+07	1.44E+07	1.45E+07	7.04E-01
O14818;O14818-2;H0Y586;Q8TAA3-2;Q8TAA3-5;Q8TAA3	2.42E+07	3.03E+07	0.00E+00	3.08E+07	4.29E+07	0.00E+00	3.93E+07	3.64E+07	3.90E+07	0.00E+00	3.48E+07	0.00E+00	0.00E+00	0.00E+00	5.49E+07	4.38E+07	2.35E+07	2.55E+07	2.16E+07	2.00E+07	1.68E+07	2.37E+07	7.07E-01
J3QS39;J3QTR3;F5H6Q2;P62987;F5GYU3;F5H2Z3;F5H265;B4DV12;F5H388;P62979;F5H747;F5GXK7;J3QKN0;J3QLP7;Q5PY61;P0CG47;J3QRK5;Q96C32;P0CG48;J3QSA3;K7EMA8;F5GZ39;M0R1V7;A0A087WV77;J3KSM4;M0R1M6;M0R2S1	4.43E+06	0.00E+00	0.00E+00	0.00E+00	0.00E+00	0.00E+00	0.00E+00	0.00E+00	2.42E+06	0.00E+00	0.00E+00	4.46E+06	0.00E+00	0.00E+00	0.00E+00	0.00E+00	7.07E+05	5.54E+05	8.61E+05	1.58E+06	1.57E+06	1.68E+06	7.12E-01
P42126-2;P42126;Q96DC0;H3BS70	0.00E+00	3.01E+06	0.00E+00	0.00E+00	0.00E+00	4.19E+06	0.00E+00	3.25E+06	0.00E+00	0.00E+00	0.00E+00	4.34E+06	0.00E+00	0.00E+00	0.00E+00	3.37E+06	1.13E+06	1.31E+06	9.64E+05	1.77E+06	1.83E+06	1.80E+06	7.12E-01
A0A0A0MRF4;P49406;S4R3W9	6.40E+06	0.00E+00	0.00E+00	0.00E+00	0.00E+00	0.00E+00	0.00E+00	0.00E+00	0.00E+00	0.00E+00	0.00E+00	0.00E+00	3.63E+06	0.00E+00	0.00E+00	0.00E+00	6.27E+05	8.00E+05	4.54E+05	1.79E+06	2.26E+06	1.28E+06	7.13E-01
Q92522	5.24E+07	8.18E+06	1.19E+08	5.72E+06	0.00E+00	1.09E+07	0.00E+00	8.25E+06	4.42E+07	0.00E+00	1.03E+07	1.32E+07	0.00E+00	4.70E+07	1.10E+07	3.10E+07	2.26E+07	2.56E+07	1.96E+07	3.13E+07	4.15E+07	1.87E+07	7.14E-01
Q99798;A2A274	4.01E+08	5.02E+08	1.80E+08	2.88E+08	4.65E+08	7.38E+07	5.66E+08	4.55E+08	2.12E+08	3.93E+08	3.16E+08	2.16E+08	4.53E+08	2.10E+08	5.28E+08	3.84E+08	3.53E+08	3.66E+08	3.39E+08	1.43E+08	1.71E+08	1.21E+08	7.14E-01

P13639	1.83E+07	1.27E+07	1.46E+07	7.83E+06	1.01E+07	1.71E+07	1.38E+07	1.38E+07	1.55E+07	2.02E+07	5.61E+06	1.36E+07	8.97E+06	1.23E+07	1.63E+07	9.74E+06	1.31E+07	1.35E+07	1.28E+07	3.95E+06	3.41E+06	4.64E+06	7.19E-01
Q9UJZ1;A0A087WYB4;Q9UJZ1-2	0.00E+00	8.82E+06	0.00E+00	4.59E+06	1.82E+07	8.97E+06	1.17E+07	1.29E+07	5.39E+06	7.00E+06	6.83E+06	0.00E+00	1.31E+07	0.00E+00	1.41E+07	1.03E+07	7.61E+06	8.15E+06	7.08E+06	5.69E+06	6.36E+06	5.32E+06	7.20E-01
Q01518;Q01518-2;Q5T0R7;Q5T0R6;Q5T0R5;Q5T0R4;Q5T0R3;Q5T0R2;Q5T0R1;Q5T0R9	1.66E+07	0.00E+00	0.00E+00	2.48E+07	0.00E+00	2.64E+07	1.31E+07	0.00E+00	0.00E+00	2.88E+07	0.00E+00	0.00E+00	2.11E+07	0.00E+00	1.41E+07	0.00E+00	9.05E+06	1.01E+07	8.00E+06	1.13E+07	1.16E+07	1.17E+07	7.23E-01
F8VZJ2;H0YHX9;Q13765;E9PAV3-2;E9PAV3;F8W0W4;F8W1N5;F8VNW4	1.06E+07	0.00E+00	5.43E+06	6.55E+06	0.00E+00	1.91E+07	1.36E+07	2.07E+07	5.36E+06	7.03E+06	3.56E+06	1.28E+07	7.59E+06	0.00E+00	1.42E+07	1.57E+07	8.89E+06	9.50E+06	8.28E+06	6.63E+06	7.94E+06	5.51E+06	7.27E-01
P54819-2;F8W1A4;P54819;F8VZG5;F8VY04;P54819-3;P54819-6;P54819-5;P54819-4;G3V213	1.74E+07	0.00E+00	0.00E+00	0.00E+00	1.04E+07	1.52E+07	8.09E+06	1.15E+07	1.65E+07	1.52E+07	0.00E+00	6.92E+06	0.00E+00	1.36E+07	0.00E+00	0.00E+00	7.18E+06	7.83E+06	6.53E+06	7.08E+06	7.07E+06	7.52E+06	7.28E-01
F8W914;Q9NQC3-2;Q9NQC3-5;Q9NQC3-3;H7C106	0.00E+00	0.00E+00	0.00E+00	0.00E+00	7.15E+07	0.00E+00	0.00E+00	0.00E+00	0.00E+00	3.88E+07	0.00E+00	0.00E+00	0.00E+00	4.30E+07	2.13E+07	0.00E+00	1.09E+07	8.94E+06	1.29E+07	2.16E+07	2.53E+07	1.88E+07	7.29E-01
P02768;H0YA55;P02768-3;A0A087WWT3;P02768-2;C9JKR2;D6RHD5;B7WNR0	0.00E+00	0.00E+00	0.00E+00	0.00E+00	0.00E+00	0.00E+00	0.00E+00	5.81E+06	0.00E+00	0.00E+00	0.00E+00	0.00E+00	0.00E+00	0.00E+00	0.00E+00	3.43E+06	5.78E+05	7.26E+05	4.29E+05	1.64E+06	2.05E+06	1.21E+06	7.30E-01
O43143	8.68E+06	1.00E+07	0.00E+00	0.00E+00	0.00E+00	0.00E+00	0.00E+00	0.00E+00	0.00E+00	0.00E+00	0.00E+00	9.95E+06	8.41E+06	0.00E+00	0.00E+00	6.40E+06	2.72E+06	2.34E+06	3.09E+06	4.23E+06	4.34E+06	4.38E+06	7.34E-01
P61981	0.00E+00	0.00E+00	0.00E+00	0.00E+00	0.00E+00	5.30E+06	0.00E+00	0.00E+00	0.00E+00	0.00E+00	0.00E+00	8.87E+06	0.00E+00	0.00E+00	0.00E+00	0.00E+00	8.86E+05	6.63E+05	1.11E+06	2.51E+06	1.87E+06	3.14E+06	7.35E-01
Q8NFV4-6;Q8NFV4-4;Q8NFV4	0.00E+00	0.00E+00	0.00E+00	0.00E+00	0.00E+00	0.00E+00	7.54E+06	0.00E+00	0.00E+00	0.00E+00	0.00E+00	0.00E+00	0.00E+00	0.00E+00	0.00E+00	4.51E+06	7.53E+05	9.42E+05	5.63E+05	2.13E+06	2.66E+06	1.59E+06	7.35E-01
P62191-2;P62191	0.00E+00	0.00E+00	0.00E+00	9.83E+06	2.36E+07	1.40E+07	2.02E+07	3.29E+07	6.21E+06	2.38E+07	0.00E+00	1.07E+07	1.93E+07	3.03E+07	0.00E+00	2.70E+07	1.36E+07	1.26E+07	1.47E+07	1.19E+07	1.24E+07	1.21E+07	7.39E-01
P50395;P50395-2;Q5SX87	1.27E+07	0.00E+00	1.03E+07	1.12E+07	0.00E+00	0.00E+00	0.00E+00	1.09E+07	1.32E+07	0.00E+00	9.58E+06	1.38E+07	0.00E+00	0.00E+00	0.00E+00	0.00E+00	5.10E+06	5.63E+06	4.57E+06	6.06E+06	6.06E+06	6.43E+06	7.39E-01
Q13724-2;Q13724;C9J8D4	0.00E+00	3.00E+08	5.77E+07	1.47E+08	3.76E+08	0.00E+00	2.02E+08	2.57E+08	1.68E+08	1.77E+08	2.26E+08	1.79E+08	2.17E+08	2.01E+08	1.45E+08	1.63E+08	1.76E+08	1.67E+08	1.85E+08	9.84E+07	1.41E+08	2.79E+07	7.40E-01
P22314-2;P22314	2.00E+07	3.11E+07	3.61E+07	0.00E+00	0.00E+00	1.59E+07	2.21E+07	2.45E+07	0.00E+00	1.57E+07	0.00E+00	2.54E+07	4.26E+07	0.00E+00	4.32E+07	4.57E+07	2.01E+07	1.87E+07	2.16E+07	1.67E+07	1.31E+07	2.05E+07	7.43E-01
Q9HB71;Q9HB71-3	2.56E+07	3.05E+07	2.83E+07	0.00E+00	0.00E+00	3.98E+07	5.38E+07	5.49E+07	0.00E+00	0.00E+00	0.00E+00	3.77E+07	6.91E+07	0.00E+00	4.80E+07	4.52E+07	2.70E+07	2.91E+07	2.50E+07	2.41E+07	2.10E+07	2.81E+07	7.46E-01
P61019;E9PKL7;P61019-2	6.05E+07	7.27E+07	6.72E+07	5.96E+07	7.07E+07	5.93E+07	6.63E+07	5.72E+07	8.40E+07	3.85E+07	6.19E+07	9.95E+07	5.44E+07	4.77E+07	7.03E+07	7.64E+07	6.54E+07	6.42E+07	6.66E+07	1.43E+07	5.80E+06	2.01E+07	7.49E-01
P22626;P22626-2;A0A087WUI2	1.07E+07	1.08E+07	0.00E+00	1.81E+07	1.31E+07	0.00E+00	0.00E+00	9.33E+06	2.03E+07	1.73E+07	0.00E+00	0.00E+00	0.00E+00	1.38E+07	0.00E+00	0.00E+00	7.09E+06	7.75E+06	6.44E+06	7.82E+06	6.93E+06	9.05E+06	7.50E-01
E9PC52;Q16576;Q16576-2;Q5JP02	0.00E+00	1.44E+07	0.00E+00	2.92E+06	0.00E+00	0.00E+00	1.04E+08	5.25E+07	5.35E+06	3.37E+07	1.06E+07	0.00E+00	1.86E+07	1.11E+06	7.66E+07	7.28E+07	2.45E+07	2.17E+07	2.73E+07	3.36E+07	3.78E+07	3.12E+07	7.50E-01
P53396-2;P53396;P53396-3	0.00E+00	5.25E+06	4.07E+06	3.70E+06	0.00E+00	1.00E+07	0.00E+00	0.00E+00	4.50E+06	7.32E+06	0.00E+00	0.00E+00	2.47E+06	0.00E+00	0.00E+00	4.54E+06	2.62E+06	2.88E+06	2.35E+06	3.15E+06	3.62E+06	2.83E+06	7.51E-01
P49915;P49915-2	0.00E+00	0.00E+00	0.00E+00	0.00E+00	0.00E+00	1.49E+07	5.45E+06	0.00E+00	0.00E+00	6.74E+06	0.00E+00	0.00E+00	0.00E+00	7.89E+06	0.00E+00	0.00E+00	2.19E+06	2.55E+06	1.83E+06	4.35E+06	5.35E+06	3.40E+06	7.54E-01
Q96I99;H0Y852;E9PDQ8;Q96I99-2	0.00E+00	0.00E+00	0.00E+00	0.00E+00	6.62E+06	7.39E+06	0.00E+00	5.27E+06	0.00E+00	0.00E+00	0.00E+00	0.00E+00	4.87E+06	1.40E+07	0.00E+00	5.92E+06	2.75E+06	2.41E+06	3.10E+06	4.16E+06	3.38E+06	5.04E+06	7.54E-01
A0A087WTV6;Q96C36;A0A087WZR9;J3KR12	0.00E+00	2.20E+07	0.00E+00	5.61E+07	2.81E+07	0.00E+00	5.32E+07	2.80E+07	4.42E+07	0.00E+00	2.06E+07	4.18E+07	2.22E+07	4.64E+07	1.69E+07	2.03E+07	2.50E+07	2.34E+07	2.66E+07	1.92E+07	2.28E+07	1.62E+07	7.57E-01
P23246-2;P23246	0.00E+00	0.00E+00	0.00E+00	0.00E+00	0.00E+00	0.00E+00	3.66E+06	0.00E+00	0.00E+00	0.00E+00	0.00E+00	0.00E+00	2.31E+06	0.00E+00	0.00E+00	0.00E+00	3.73E+05	4.57E+05	2.89E+05	1.05E+06	1.29E+06	8.18E+05	7.60E-01
P56537;B7ZBH1;P56537-2	0.00E+00	0.00E+00	0.00E+00	0.00E+00	0.00E+00	0.00E+00	0.00E+00	2.25E+06	0.00E+00	0.00E+00	0.00E+00	0.00E+00	0.00E+00	0.00E+00	3.56E+06	0.00E+00	3.63E+05	2.82E+05	4.44E+05	1.02E+06	7.96E+05	1.26E+06	7.61E-01

Q9Y5K5-2;Q9Y5K5-4;Q9Y5K5-3;Q9Y5K5;Q5LJA5;Q5LJA9;H0Y4E0;H0Y6Y4;Q5LJB0;H0Y636	0.00E+00	0.00E+00	0.00E+00	0.00E+00	4.93E+06	4.51E+06	0.00E+00	0.00E+00	3.30E+06	0.00E+00	9.66E+06	0.00E+00	0.00E+00	0.00E+00	0.00E+00	0.00E+00	1.40E+06	1.18E+06	1.62E+06	2.80E+06	2.19E+06	3.45E+06	7.66E-01
Q04695;F5GWP8;K7EPJ9	0.00E+00	0.00E+00	0.00E+00	0.00E+00	0.00E+00	1.08E+07	6.28E+06	0.00E+00	0.00E+00	0.00E+00	0.00E+00	0.00E+00	0.00E+00	0.00E+00	5.73E+06	7.05E+06	1.87E+06	2.13E+06	1.60E+06	3.49E+06	4.13E+06	2.98E+06	7.71E-01
O75323;H7C333	0.00E+00	0.00E+00	0.00E+00	0.00E+00	0.00E+00	0.00E+00	1.01E+07	0.00E+00	0.00E+00	8.27E+06	0.00E+00	0.00E+00	6.00E+06	0.00E+00	0.00E+00	0.00E+00	1.53E+06	1.27E+06	1.78E+06	3.37E+06	3.59E+06	3.36E+06	7.71E-01
P62195-2;P62195;J3QSA9;J3QQM1	1.45E+07	0.00E+00	1.45E+07	1.69E+07	0.00E+00	1.28E+07	0.00E+00	2.30E+07	1.67E+07	0.00E+00	2.18E+07	1.12E+07	0.00E+00	1.61E+07	9.65E+06	1.64E+07	1.08E+07	1.02E+07	1.15E+07	8.23E+06	8.98E+06	7.97E+06	7.72E-01
P23368;P23368-2	1.25E+08	6.51E+07	9.91E+07	8.49E+07	1.04E+08	1.10E+08	6.72E+07	9.44E+07	1.25E+08	0.00E+00	8.55E+07	2.53E+08	1.10E+08	4.25E+07	5.94E+07	1.39E+08	9.76E+07	9.36E+07	1.02E+08	5.41E+07	2.05E+07	7.62E+07	7.77E-01
P35232;C9JW96;C9JZ20;E7ESE2;E9PCW0	6.06E+06	0.00E+00	6.04E+06	1.02E+07	0.00E+00	3.35E+06	0.00E+00	0.00E+00	6.75E+06	5.23E+06	0.00E+00	8.34E+06	0.00E+00	0.00E+00	5.18E+06	4.22E+06	3.46E+06	3.20E+06	3.71E+06	3.50E+06	3.88E+06	3.31E+06	7.80E-01
Q96HE7	0.00E+00	0.00E+00	0.00E+00	0.00E+00	0.00E+00	7.95E+06	0.00E+00	0.00E+00	0.00E+00	0.00E+00	0.00E+00	0.00E+00	0.00E+00	0.00E+00	0.00E+00	5.27E+06	8.26E+05	9.94E+05	6.59E+05	2.31E+06	2.81E+06	1.86E+06	7.83E-01
E9PIE4;Q9Y6C9	0.00E+00	0.00E+00	0.00E+00	0.00E+00	0.00E+00	0.00E+00	1.19E+07	1.64E+07	0.00E+00	0.00E+00	0.00E+00	5.66E+06	9.04E+06	0.00E+00	0.00E+00	7.48E+06	3.16E+06	3.54E+06	2.77E+06	5.30E+06	6.66E+06	3.93E+06	7.83E-01
O15260-2;Q5T8U5;O15260;O15260-3;B7Z1G8	5.07E+07	1.25E+08	7.82E+07	3.25E+07	6.03E+07	6.38E+07	9.53E+07	7.73E+07	2.83E+07	1.46E+08	4.64E+07	1.19E+08	9.97E+07	0.00E+00	9.64E+07	9.18E+07	7.57E+07	7.29E+07	7.85E+07	3.89E+07	2.83E+07	4.91E+07	7.83E-01
P60174-1;P60174;P60174-4	0.00E+00	0.00E+00	3.30E+07	1.89E+07	0.00E+00	6.09E+07	8.06E+07	4.51E+07	0.00E+00	2.10E+07	1.95E+07	2.58E+07	2.98E+07	0.00E+00	5.95E+07	5.34E+07	2.80E+07	2.98E+07	2.61E+07	2.57E+07	3.06E+07	2.18E+07	7.86E-01
P62906	1.31E+07	0.00E+00	0.00E+00	1.59E+08	6.13E+07	7.95E+07	4.22E+07	2.66E+07	9.37E+07	8.23E+07	1.57E+08	1.80E+07	0.00E+00	5.37E+07	0.00E+00	3.60E+07	5.14E+07	4.77E+07	5.51E+07	5.20E+07	5.33E+07	5.40E+07	7.88E-01
P02533	0.00E+00	0.00E+00	6.19E+06	1.95E+07	1.75E+07	1.24E+07	0.00E+00	1.43E+07	0.00E+00	0.00E+00	0.00E+00	0.00E+00	2.12E+07	1.89E+07	0.00E+00	1.96E+07	8.09E+06	8.73E+06	7.46E+06	9.03E+06	8.21E+06	1.03E+07	7.89E-01
P12004	1.17E+07	1.18E+07	0.00E+00	0.00E+00	0.00E+00	8.28E+06	0.00E+00	0.00E+00	0.00E+00	1.41E+07	0.00E+00	0.00E+00	1.13E+07	0.00E+00	0.00E+00	0.00E+00	3.57E+06	3.97E+06	3.18E+06	5.58E+06	5.58E+06	5.94E+06	7.89E-01
Q9Y230;Q9Y230-2;M0R0Y3	0.00E+00	1.34E+07	1.81E+07	0.00E+00	0.00E+00	3.01E+07	2.88E+07	3.20E+07	0.00E+00	0.00E+00	0.00E+00	6.06E+07	3.02E+07	0.00E+00	1.61E+07	3.57E+07	1.66E+07	1.53E+07	1.78E+07	1.83E+07	1.41E+07	2.26E+07	7.94E-01
O75947;O75947-2	0.00E+00	0.00E+00	0.00E+00	0.00E+00	0.00E+00	6.51E+06	1.58E+07	1.16E+07	0.00E+00	0.00E+00	0.00E+00	0.00E+00	1.44E+07	0.00E+00	1.42E+07	1.24E+07	4.69E+06	4.24E+06	5.13E+06	6.53E+06	6.36E+06	7.11E+06	7.95E-01
P28074-3;P28074	5.80E+07	7.71E+07	6.06E+07	3.80E+07	6.38E+07	6.51E+07	5.92E+07	6.33E+07	3.37E+07	6.98E+07	4.55E+07	6.08E+07	8.66E+07	0.00E+00	8.67E+07	7.85E+07	5.92E+07	6.06E+07	5.77E+07	2.19E+07	1.09E+07	3.00E+07	7.98E-01
P18124;A8MUD9	9.47E+06	1.71E+07	1.04E+07	7.71E+06	2.14E+07	1.30E+07	3.05E+07	3.43E+07	2.56E+07	2.09E+07	1.65E+07	1.28E+07	1.73E+07	1.64E+07	0.00E+00	2.50E+07	1.74E+07	1.80E+07	1.68E+07	8.80E+06	9.98E+06	8.11E+06	8.00E-01
Q9Y490	1.88E+07	1.65E+07	0.00E+00	0.00E+00	1.75E+07	0.00E+00	1.11E+07	0.00E+00	2.02E+07	0.00E+00	0.00E+00	1.93E+07	0.00E+00	2.05E+07	0.00E+00	1.36E+07	8.59E+06	7.99E+06	9.20E+06	9.16E+06	8.82E+06	1.01E+07	8.01E-01
O75400-2;O75400-3;O75400	2.53E+06	0.00E+00	0.00E+00	0.00E+00	3.56E+06	4.59E+06	0.00E+00	2.84E+06	3.53E+06	0.00E+00	0.00E+00	0.00E+00	3.13E+06	4.60E+06	0.00E+00	4.26E+06	1.82E+06	1.69E+06	1.94E+06	1.95E+06	1.90E+06	2.12E+06	8.08E-01
K7ENH2;K9J957;P61289;P61289-3;P61289-2;A0A087WTV2;B3KQ25;K7ESG5	3.45E+07	2.24E+07	2.86E+07	2.88E+07	2.05E+07	4.61E+07	1.31E+07	3.33E+07	4.29E+07	1.73E+07	1.79E+07	5.75E+07	2.38E+07	1.28E+07	3.38E+07	3.35E+07	2.92E+07	2.84E+07	3.00E+07	1.24E+07	1.00E+07	1.51E+07	8.14E-01
P38117;P38117-2;M0QY67	0.00E+00	1.20E+07	0.00E+00	7.00E+06	0.00E+00	0.00E+00	1.01E+07	7.09E+06	0.00E+00	1.86E+07	1.41E+07	0.00E+00	9.81E+06	0.00E+00	0.00E+00	0.00E+00	4.92E+06	4.53E+06	5.31E+06	6.32E+06	5.10E+06	7.70E+06	8.14E-01
P55884;P55884-2;C9JQN7	3.86E+07	1.12E+07	4.20E+07	2.47E+07	6.78E+06	1.03E+07	0.00E+00	9.27E+06	0.00E+00	2.30E+07	1.61E+07	1.56E+07	0.00E+00	1.55E+07	2.36E+07	3.61E+07	1.71E+07	1.79E+07	1.62E+07	1.34E+07	1.55E+07	1.21E+07	8.19E-01
P54886-2;P54886	7.64E+07	1.26E+08	9.61E+07	5.06E+07	5.34E+07	0.00E+00	9.32E+07	7.18E+07	8.05E+07	8.75E+07	7.66E+07	5.08E+07	1.27E+08	5.37E+07	6.24E+07	5.93E+07	7.28E+07	7.10E+07	7.47E+07	3.09E+07	3.78E+07	2.47E+07	8.21E-01
P46940;H0YLE8	0.00E+00	0.00E+00	6.97E+07	0.00E+00	0.00E+00	4.05E+07	2.18E+07	2.93E+07	0.00E+00	0.00E+00	0.00E+00	5.93E+07	4.28E+07	0.00E+00	5.10E+07	3.18E+07	2.16E+07	2.02E+07	2.31E+07	2.49E+07	2.56E+07	2.59E+07	8.22E-01
P20340-2;P20340;H7BYW1;P20340-4;H0YGL6;Q9NRW1;C9JU14;J3KR73;Q9NRW1-2	0.00E+00	0.00E+00	0.00E+00	7.13E+06	0.00E+00	5.74E+06	0.00E+00	0.00E+00	0.00E+00	3.41E+06	0.00E+00	1.30E+07	0.00E+00	0.00E+00	0.00E+00	0.00E+00	1.83E+06	1.61E+06	2.05E+06	3.75E+06	3.00E+06	4.58E+06	8.23E-01
P45954-2;P45954	1.94E+07	8.32E+06	1.88E+07	0.00E+00	0.00E+00	0.00E+00	0.00E+00	0.00E+00	1.01E+07	1.35E+07	1.58E+07	0.00E+00	0.00E+00	0.00E+00	0.00E+00	0.00E+00	5.38E+06	5.82E+06	4.93E+06	7.63E+06	8.69E+06	6.98E+06	8.26E-01
Q96RP9;C9IZ01;Q96RP9-2;F8WAU4	2.38E+07	4.09E+07	3.79E+07	0.00E+00	0.00E+00	3.59E+07	4.22E+07	5.36E+07	0.00E+00	8.27E+06	1.47E+07	4.32E+07	5.25E+07	0.00E+00	4.40E+07	5.23E+07	2.81E+07	2.93E+07	2.69E+07	2.10E+07	1.98E+07	2.33E+07	8.27E-01
E9PLD0;Q9H0U4;Q92928	0.00E+00	0.00E+00	0.00E+00	4.60E+06	0.00E+00	0.00E+00	0.00E+00	4.18E+06	0.00E+00	0.00E+00	0.00E+00	0.00E+00	0.00E+00	6.79E+06	0.00E+00	0.00E+00	9.73E+05	1.10E+06	8.49E+05	2.15E+06	2.03E+06	2.40E+06	8.27E-01
Q16762	0.00E+00	8.64E+06	0.00E+00	0.00E+00	0.00E+00	6.58E+06	5.09E+06	9.39E+06	0.00E+00	0.00E+00	0.00E+00	0.00E+00	8.89E+06	0.00E+00	5.81E+06	1.11E+07	3.47E+06	3.71E+06	3.22E+06	4.28E+06	4.17E+06	4.67E+06	8.28E-01
Q9HAV7	0.00E+00	0.00E+00	0.00E+00	0.00E+00	0.00E+00	0.00E+00	2.64E+06	2.54E+06	0.00E+00	0.00E+00	0.00E+00	0.00E+00	2.25E+06	0.00E+00	0.00E+00	1.97E+06	5.87E+05	6.47E+05	5.27E+05	1.06E+06	1.20E+06	9.79E+05	8.29E-01
Q92616	0.00E+00	0.00E+00	0.00E+00	0.00E+00	0.00E+00	6.52E+06	0.00E+00	7.38E+06	0.00E+00	7.91E+06	0.00E+00	0.00E+00	0.00E+00	0.00E+00	0.00E+00	9.16E+06	1.94E+06	1.74E+06	2.13E+06	3.50E+06	3.23E+06	3.96E+06	8.30E-01

Q03252;J9JID7	7.27E+07	4.80E+07	6.35E+07	4.29E+07	1.89E+07	0.00E+00	5.02E+07	4.20E+07	5.05E+07	3.73E+07	4.47E+07	3.11E+07	1.38E+07	6.08E+07	3.93E+07	4.42E+07	4.12E+07	4.23E+07	4.02E+07	1.86E+07	2.33E+07	1.39E+07	8.31E-01
Q16891-2;Q16891;B9A067;Q16891-3;Q16891-4;H7C463;C9J406	0.00E+00	0.00E+00	0.00E+00	0.00E+00	0.00E+00	0.00E+00	1.86E+06	0.00E+00	0.00E+00	0.00E+00	0.00E+00	0.00E+00	0.00E+00	0.00E+00	2.55E+06	0.00E+00	2.76E+05	2.33E+05	3.18E+05	7.63E+05	6.59E+05	9.00E+05	8.32E-01
Q96TA1-2;Q96TA1	0.00E+00	0.00E+00	0.00E+00	9.64E+06	9.35E+06	1.31E+07	9.33E+06	0.00E+00	1.71E+07	0.00E+00	7.90E+06	0.00E+00	0.00E+00	1.18E+07	0.00E+00	9.97E+06	5.51E+06	5.18E+06	5.84E+06	6.04E+06	5.67E+06	6.75E+06	8.35E-01
P09543-2;P09543	0.00E+00	9.93E+06	0.00E+00	4.67E+06	0.00E+00	0.00E+00	0.00E+00	0.00E+00	5.06E+06	0.00E+00	0.00E+00	6.84E+06	0.00E+00	0.00E+00	0.00E+00	0.00E+00	1.66E+06	1.82E+06	1.49E+06	3.15E+06	3.66E+06	2.79E+06	8.39E-01
P25205;J3KQ69;P25205-2	0.00E+00	0.00E+00	0.00E+00	0.00E+00	0.00E+00	0.00E+00	3.40E+06	0.00E+00	0.00E+00	0.00E+00	0.00E+00	0.00E+00	0.00E+00	0.00E+00	0.00E+00	2.53E+06	3.71E+05	4.25E+05	3.16E+05	1.03E+06	1.20E+06	8.94E+05	8.40E-01
F8W9J4;Q03001;Q03001-8;E7ERU0;E9PHM6;F6QMI7;E9PEB9	0.00E+00	0.00E+00	1.88E+06	0.00E+00	0.00E+00	0.00E+00	0.00E+00	0.00E+00	0.00E+00	0.00E+00	0.00E+00	0.00E+00	1.40E+06	0.00E+00	0.00E+00	0.00E+00	2.05E+05	2.35E+05	1.75E+05	5.67E+05	6.65E+05	4.95E+05	8.40E-01
Q7Z6Z7-2;Q7Z6Z7-3;Q7Z6Z7	0.00E+00	0.00E+00	0.00E+00	7.57E+06	3.47E+06	0.00E+00	0.00E+00	0.00E+00	0.00E+00	4.72E+06	0.00E+00	4.33E+06	0.00E+00	0.00E+00	0.00E+00	0.00E+00	1.26E+06	1.38E+06	1.13E+06	2.38E+06	2.78E+06	2.10E+06	8.42E-01
O00231;O00231-2;J3QRY4	0.00E+00	0.00E+00	0.00E+00	6.26E+06	9.50E+06	0.00E+00	6.02E+06	0.00E+00	0.00E+00	6.34E+06	0.00E+00	0.00E+00	9.28E+06	0.00E+00	9.51E+06	0.00E+00	2.93E+06	2.72E+06	3.14E+06	4.04E+06	3.90E+06	4.44E+06	8.44E-01
Q16222-2;Q16222-3;Q16222	0.00E+00	0.00E+00	0.00E+00	1.20E+08	4.24E+07	4.45E+07	2.29E+07	1.96E+07	3.51E+07	2.68E+07	7.83E+07	1.17E+07	1.49E+07	3.65E+07	0.00E+00	1.93E+07	2.95E+07	3.11E+07	2.78E+07	3.19E+07	4.01E+07	2.38E+07	8.45E-01
P48668;P02538	0.00E+00	0.00E+00	0.00E+00	0.00E+00	0.00E+00	6.97E+06	5.48E+06	4.65E+06	0.00E+00	0.00E+00	0.00E+00	7.34E+06	0.00E+00	0.00E+00	5.74E+06	6.57E+06	2.30E+06	2.14E+06	2.46E+06	3.12E+06	3.02E+06	3.42E+06	8.46E-01
O00264;O00264-2	7.39E+06	1.17E+07	1.07E+07	0.00E+00	0.00E+00	0.00E+00	2.16E+07	1.89E+07	0.00E+00	8.02E+06	0.00E+00	0.00E+00	1.21E+07	1.13E+07	1.55E+07	1.73E+07	8.40E+06	8.79E+06	8.02E+06	7.64E+06	8.55E+06	7.18E+06	8.48E-01
Q86VP6;Q86VP6-2	0.00E+00	0.00E+00	0.00E+00	0.00E+00	0.00E+00	0.00E+00	0.00E+00	2.29E+07	0.00E+00	0.00E+00	0.00E+00	0.00E+00	2.25E+06	6.96E+06	0.00E+00	1.94E+07	3.22E+06	2.86E+06	3.58E+06	7.24E+06	8.08E+06	6.84E+06	8.51E-01
P46777;Q5T7N0	0.00E+00	0.00E+00	0.00E+00	0.00E+00	6.37E+06	8.84E+06	0.00E+00	0.00E+00	0.00E+00	0.00E+00	0.00E+00	1.94E+07	0.00E+00	0.00E+00	0.00E+00	0.00E+00	2.16E+06	1.90E+06	2.42E+06	5.29E+06	3.58E+06	6.85E+06	8.51E-01
P51991-2;P51991	2.70E+07	2.39E+07	0.00E+00	0.00E+00	0.00E+00	5.06E+07	3.14E+07	4.36E+07	0.00E+00	0.00E+00	1.43E+07	6.91E+07	2.86E+07	9.06E+06	2.52E+07	4.71E+07	2.31E+07	2.21E+07	2.42E+07	2.15E+07	2.02E+07	2.41E+07	8.53E-01
P30048-2;P30048	1.38E+08	1.92E+08	1.59E+08	6.17E+07	6.90E+07	1.48E+08	9.82E+07	1.08E+08	1.16E+08	7.23E+07	9.01E+07	1.96E+08	1.98E+08	1.78E+07	1.46E+08	1.78E+08	1.24E+08	1.22E+08	1.27E+08	5.40E+07	4.53E+07	6.46E+07	8.54E-01
P63104;E7EX29;B0AZS6;E7ESK7;P63104-2	0.00E+00	4.03E+06	0.00E+00	0.00E+00	0.00E+00	0.00E+00	6.65E+06	5.79E+06	3.14E+06	0.00E+00	3.33E+06	4.13E+06	4.01E+06	0.00E+00	0.00E+00	0.00E+00	1.94E+06	2.06E+06	1.83E+06	2.42E+06	2.93E+06	1.98E+06	8.55E-01
Q02218-2;Q02218;E9PDF2;E9PCR7;E9PFG7	8.19E+06	1.94E+07	2.04E+07	4.86E+06	1.48E+07	0.00E+00	5.90E+06	9.23E+06	4.88E+06	1.16E+07	1.75E+07	1.16E+07	0.00E+00	0.00E+00	1.53E+07	1.66E+07	1.00E+07	1.04E+07	9.69E+06	6.96E+06	7.24E+06	7.16E+06	8.55E-01
O76003	2.06E+07	2.47E+07	4.68E+07	1.02E+07	0.00E+00	1.16E+07	0.00E+00	3.37E+07	0.00E+00	2.72E+07	1.38E+07	3.30E+07	2.74E+07	0.00E+00	1.07E+07	2.47E+07	1.78E+07	1.84E+07	1.71E+07	1.42E+07	1.64E+07	1.29E+07	8.57E-01
P27348;E9PG15	0.00E+00	1.73E+07	0.00E+00	2.66E+07	3.44E+07	0.00E+00	1.66E+07	2.05E+07	1.45E+07	1.86E+07	2.26E+07	1.59E+07	1.89E+07	1.48E+07	1.79E+07	0.00E+00	1.49E+07	1.44E+07	1.54E+07	1.02E+07	1.32E+07	6.76E+06	8.58E-01
Q7KZF4	1.10E+07	0.00E+00	0.00E+00	6.92E+06	1.37E+07	0.00E+00	8.82E+06	9.84E+06	1.19E+07	0.00E+00	1.63E+07	0.00E+00	0.00E+00	0.00E+00	1.36E+07	1.32E+07	6.57E+06	6.28E+06	6.87E+06	6.34E+06	5.54E+06	7.44E+06	8.61E-01
Q92542-2;Q92542;H0Y6T7;Q5T205;H0Y3Z4	9.80E+08	5.65E+08	7.10E+08	5.90E+08	3.85E+08	5.46E+08	8.89E+06	7.75E+08	7.42E+08	4.01E+08	3.54E+08	7.91E+08	5.54E+08	5.73E+08	5.29E+08	4.54E+08	5.60E+08	5.70E+08	5.50E+08	2.23E+08	2.88E+08	1.54E+08	8.64E-01
P10809	3.03E+07	2.13E+07	3.30E+07	3.76E+07	1.76E+07	0.00E+00	2.49E+07	2.05E+07	4.38E+07	3.46E+07	3.50E+07	0.00E+00	0.00E+00	3.51E+07	2.44E+07	2.20E+07	2.38E+07	2.31E+07	2.44E+07	1.38E+07	1.16E+07	1.65E+07	8.67E-01
Q12906-5;Q12906-4;Q12906-2;Q12906-6;Q12906-3;Q12906;Q12906-7;K7EKJ9	2.54E+07	2.57E+07	2.47E+07	3.23E+07	4.34E+07	3.10E+07	0.00E+00	3.37E+07	2.48E+07	2.74E+07	3.51E+07	2.60E+07	2.61E+07	2.54E+07	2.33E+07	2.20E+07	2.66E+07	2.70E+07	2.62E+07	8.98E+06	1.25E+07	3.95E+06	8.69E-01
O60506-4;O60506-3;O60506-2;O60506;B7Z645;O60506-5	0.00E+00	0.00E+00	0.00E+00	5.09E+06	0.00E+00	0.00E+00	0.00E+00	0.00E+00	0.00E+00	0.00E+00	6.45E+06	0.00E+00	0.00E+00	0.00E+00	0.00E+00	0.00E+00	7.21E+05	6.36E+05	8.06E+05	1.99E+06	1.80E+06	2.28E+06	8.71E-01
P09622-2;P09622-3;E9PEX6;P09622	0.00E+00	0.00E+00	0.00E+00	0.00E+00	0.00E+00	0.00E+00	4.57E+06	0.00E+00	0.00E+00	0.00E+00	0.00E+00	0.00E+00	0.00E+00	0.00E+00	5.76E+06	0.00E+00	6.46E+05	5.71E+05	7.20E+05	1.78E+06	1.62E+06	2.04E+06	8.74E-01
P51665;H3BNT7;H3BTM8	0.00E+00	0.00E+00	0.00E+00	0.00E+00	0.00E+00	4.84E+06	6.19E+06	6.58E+06	0.00E+00	0.00E+00	0.00E+00	4.84E+06	4.55E+06	0.00E+00	4.22E+06	5.83E+06	2.32E+06	2.20E+06	2.43E+06	2.77E+06	3.08E+06	2.64E+06	8.75E-01
P49720;A0A087WUL2	4.16E+06	7.77E+06	0.00E+00	0.00E+00	0.00E+00	6.30E+06	5.55E+06	5.67E+06	0.00E+00	0.00E+00	0.00E+00	8.44E+06	8.08E+06	0.00E+00	8.96E+06	6.34E+06	3.83E+06	3.68E+06	3.98E+06	3.68E+06	3.20E+06	4.32E+06	8.78E-01
C9JJ34;C9JDM3;P43487-2;P43487;F6WQW2;C9JIC6;C9JXG8;C9JGV6	3.65E+07	0.00E+00	0.00E+00	2.59E+07	1.65E+07	2.34E+07	0.00E+00	2.62E+07	1.92E+07	3.38E+07	0.00E+00	2.02E+07	2.69E+07	2.31E+07	0.00E+00	1.37E+07	1.66E+07	1.61E+07	1.71E+07	1.28E+07	1.44E+07	1.21E+07	8.79E-01

P39656-3;P39656;P39656-2;U3KQ84	6.83E+06	0.00E+00	0.00E+00	0.00E+00	0.00E+00	0.00E+00	0.00E+00	0.00E+00	0.00E+00	5.48E+06	0.00E+00	0.00E+00	0.00E+00	0.00E+00	0.00E+00	0.00E+00	7.69E+05	8.54E+05	6.85E+05	2.12E+06	2.42E+06	1.94E+06	8.79E-01
Q8NC51-4;Q8NC51-3;Q8NC51-2;Q8NC51	4.74E+07	1.95E+07	0.00E+00	4.54E+07	0.00E+00	0.00E+00	6.93E+07	2.70E+07	2.48E+07	2.67E+07	3.02E+07	2.81E+07	0.00E+00	2.86E+07	2.69E+07	3.15E+07	2.53E+07	2.61E+07	2.46E+07	1.92E+07	2.61E+07	1.02E+07	8.82E-01
Q9NR30-2;Q9NR30	9.11E+06	1.36E+07	1.01E+07	7.05E+06	8.39E+06	7.12E+06	0.00E+00	0.00E+00	5.18E+06	6.37E+06	6.59E+06	4.64E+06	9.96E+06	6.29E+06	9.26E+06	9.24E+06	7.06E+06	6.92E+06	7.19E+06	3.53E+06	4.75E+06	2.02E+06	8.84E-01
P41250	0.00E+00	1.14E+07	0.00E+00	0.00E+00	0.00E+00	1.12E+07	2.20E+07	1.43E+07	0.00E+00	0.00E+00	0.00E+00	8.30E+06	1.59E+07	0.00E+00	1.20E+07	1.80E+07	7.06E+06	7.36E+06	6.77E+06	7.88E+06	8.53E+06	7.76E+06	8.87E-01
P40429;M0QYS1;Q6NVV1;Q8J015	0.00E+00	0.00E+00	0.00E+00	0.00E+00	0.00E+00	0.00E+00	1.22E+07	5.76E+06	0.00E+00	0.00E+00	0.00E+00	0.00E+00	0.00E+00	0.00E+00	1.07E+07	9.85E+06	2.40E+06	2.24E+06	2.56E+06	4.47E+06	4.49E+06	4.75E+06	8.91E-01
Q04828;A0A0A0MT30;A6NHU4;H0Y804;B4DK69;S4R3Z2;P42330;A0A0A0MSS8;P52895	0.00E+00	1.04E+07	0.00E+00	0.00E+00	0.00E+00	0.00E+00	7.09E+06	1.10E+07	8.66E+06	0.00E+00	0.00E+00	0.00E+00	8.03E+06	0.00E+00	9.15E+06	0.00E+00	3.39E+06	3.56E+06	3.23E+06	4.60E+06	5.03E+06	4.47E+06	8.93E-01
Q14566	1.94E+07	8.67E+06	8.66E+06	0.00E+00	0.00E+00	1.87E+07	0.00E+00	2.41E+07	1.46E+07	0.00E+00	0.00E+00	2.42E+07	1.16E+07	0.00E+00	1.82E+07	1.61E+07	1.03E+07	9.94E+06	1.06E+07	9.30E+06	9.76E+06	9.47E+06	8.95E-01
P30040;F8VY02	0.00E+00	0.00E+00	0.00E+00	0.00E+00	0.00E+00	1.03E+07	0.00E+00	0.00E+00	8.50E+06	0.00E+00	0.00E+00	0.00E+00	0.00E+00	0.00E+00	0.00E+00	0.00E+00	1.17E+06	1.28E+06	1.06E+06	3.22E+06	3.63E+06	3.00E+06	8.96E-01
M0QYT0;P14866-2;M0QXS5;M0R076	1.42E+07	0.00E+00	0.00E+00	5.84E+07	1.34E+07	1.29E+07	1.35E+07	1.43E+07	0.00E+00	2.53E+07	0.00E+00	3.08E+07	0.00E+00	2.66E+07	0.00E+00	3.50E+07	1.53E+07	1.58E+07	1.47E+07	1.66E+07	1.83E+07	1.60E+07	8.97E-01
B4DJV2;O75390	2.85E+07	3.41E+07	2.29E+07	0.00E+00	2.63E+07	3.15E+07	3.86E+07	2.89E+07	0.00E+00	0.00E+00	0.00E+00	0.00E+00	6.41E+07	4.63E+07	5.03E+07	3.88E+07	2.56E+07	2.63E+07	2.49E+07	2.05E+07	1.17E+07	2.76E+07	8.98E-01
P61247;D6RAT0;D6RG13;H0Y8L7;D6RB09;E9PFI5;H0Y9Y4;D6R9B6	0.00E+00	7.99E+06	0.00E+00	0.00E+00	0.00E+00	0.00E+00	0.00E+00	1.13E+07	5.80E+06	0.00E+00	1.62E+07	0.00E+00	0.00E+00	0.00E+00	0.00E+00	0.00E+00	2.58E+06	2.41E+06	2.75E+06	5.05E+06	4.56E+06	5.81E+06	8.99E-01
Q5T093;Q5T092;A0A0A0MR06;Q5T091;O15258;Q9P0H9	0.00E+00	0.00E+00	0.00E+00	0.00E+00	0.00E+00	0.00E+00	0.00E+00	1.10E+07	0.00E+00	0.00E+00	0.00E+00	9.20E+06	0.00E+00	0.00E+00	0.00E+00	0.00E+00	1.26E+06	1.38E+06	1.15E+06	3.47E+06	3.90E+06	3.25E+06	9.00E-01
P04179-3;P04179-4;P04179;F5H3C5;F5H4R2;P04179-2;F5GYZ5	0.00E+00	0.00E+00	1.85E+07	0.00E+00	2.18E+07	1.64E+07	1.32E+07	1.15E+07	0.00E+00	1.71E+07	0.00E+00	1.25E+07	1.68E+07	0.00E+00	1.51E+07	1.57E+07	9.91E+06	1.02E+07	9.65E+06	8.27E+06	8.98E+06	8.11E+06	9.05E-01
F2Z393;P37837	0.00E+00	0.00E+00	0.00E+00	0.00E+00	0.00E+00	0.00E+00	1.04E+07	0.00E+00	0.00E+00	0.00E+00	0.00E+00	0.00E+00	0.00E+00	0.00E+00	0.00E+00	8.78E+06	1.20E+06	1.30E+06	1.10E+06	3.29E+06	3.69E+06	3.10E+06	9.06E-01
P33176	0.00E+00	4.02E+06	0.00E+00	0.00E+00	0.00E+00	0.00E+00	0.00E+00	0.00E+00	0.00E+00	0.00E+00	0.00E+00	0.00E+00	0.00E+00	3.38E+06	0.00E+00	0.00E+00	4.63E+05	5.02E+05	4.23E+05	1.27E+06	1.42E+06	1.20E+06	9.06E-01
Q7L1Q6-2;Q7L1Q6;Q7L1Q6-4;Q7L1Q6-3;C9IZ80	3.80E+07	1.14E+08	1.84E+08	1.10E+08	1.87E+08	1.28E+08	1.82E+08	1.54E+08	7.40E+07	4.09E+08	1.25E+08	9.20E+07	8.21E+07	0.00E+00	1.52E+08	1.17E+08	1.34E+08	1.37E+08	1.32E+08	8.96E+07	5.09E+07	1.21E+08	9.06E-01
P04792;F8WE04	0.00E+00	0.00E+00	0.00E+00	0.00E+00	0.00E+00	0.00E+00	0.00E+00	3.83E+06	0.00E+00	0.00E+00	0.00E+00	0.00E+00	0.00E+00	0.00E+00	0.00E+00	4.53E+06	5.23E+05	4.79E+05	5.66E+05	1.43E+06	1.36E+06	1.60E+06	9.08E-01
H7C312;P23919-2;P23919;G5E9E9;H7C3A4;H7BZ20	0.00E+00	0.00E+00	0.00E+00	0.00E+00	0.00E+00	6.08E+06	7.24E+06	4.02E+06	0.00E+00	0.00E+00	0.00E+00	6.54E+06	6.20E+06	0.00E+00	0.00E+00	6.08E+06	2.26E+06	2.17E+06	2.35E+06	3.08E+06	3.12E+06	3.25E+06	9.09E-01
O43809;H3BND3;H3BV41	0.00E+00	1.21E+08	1.24E+07	9.42E+07	2.63E+08	0.00E+00	4.33E+08	3.76E+08	1.76E+08	2.24E+08	1.96E+08	2.70E+07	7.88E+07	1.39E+08	2.65E+08	2.55E+08	1.66E+08	1.62E+08	1.70E+08	1.32E+08	1.73E+08	8.42E+07	9.10E-01
P18206-2;P18206;Q5JQ13	0.00E+00	0.00E+00	0.00E+00	0.00E+00	0.00E+00	8.79E+06	5.78E+06	6.41E+06	0.00E+00	0.00E+00	0.00E+00	0.00E+00	0.00E+00	0.00E+00	1.14E+07	7.71E+06	2.51E+06	2.62E+06	2.39E+06	4.01E+06	3.72E+06	4.53E+06	9.11E-01
Q9NX63;C9JRZ6;F8WAR4	0.00E+00	0.00E+00	0.00E+00	0.00E+00	0.00E+00	0.00E+00	4.40E+06	0.00E+00	0.00E+00	0.00E+00	0.00E+00	0.00E+00	0.00E+00	0.00E+00	0.00E+00	3.77E+06	5.11E+05	5.50E+05	4.71E+05	1.40E+06	1.56E+06	1.33E+06	9.15E-01
P53618;E9PP73	2.41E+07	2.01E+07	1.29E+07	4.09E+07	3.01E+07	2.74E+07	0.00E+00	1.38E+07	2.12E+07	3.36E+07	3.89E+07	0.00E+00	3.09E+07	1.59E+07	1.46E+07	1.96E+07	2.15E+07	2.12E+07	2.18E+07	1.20E+07	1.25E+07	1.24E+07	9.15E-01
P17844-2;P17844;J3KTA4;J3KRZ1	2.52E+07	7.38E+06	9.30E+06	6.34E+06	0.00E+00	1.03E+07	0.00E+00	7.74E+06	6.35E+06	0.00E+00	2.50E+06	9.15E+06	1.08E+07	0.00E+00	2.76E+07	6.32E+06	8.06E+06	8.29E+06	7.84E+06	8.13E+06	7.87E+06	8.92E+06	9.17E-01
Q9Y5M8;H7C4H2	3.96E+06	6.00E+06	2.71E+06	3.90E+06	0.00E+00	4.12E+06	0.00E+00	0.00E+00	1.48E+06	2.84E+06	2.63E+06	1.57E+06	3.76E+06	1.48E+06	4.32E+06	3.37E+06	2.63E+06	2.59E+06	2.68E+06	1.76E+06	2.32E+06	1.10E+06	9.18E-01
O00571;O00571-2	0.00E+00	0.00E+00	0.00E+00	3.40E+06	0.00E+00	0.00E+00	0.00E+00	0.00E+00	0.00E+00	0.00E+00	3.95E+06	0.00E+00	0.00E+00	0.00E+00	0.00E+00	0.00E+00	4.59E+05	4.25E+05	4.93E+05	1.26E+06	1.20E+06	1.40E+06	9.18E-01
O76031	3.51E+08	2.82E+08	3.77E+08	4.80E+08	3.48E+08	4.01E+08	2.61E+08	3.40E+08	5.41E+08	2.86E+08	4.25E+08	4.59E+08	3.11E+08	2.95E+08	2.68E+08	2.90E+08	3.57E+08	3.55E+08	3.59E+08	8.35E+07	6.82E+07	1.01E+08	9.21E-01
P25705;P25705-2;P25705-3;K7EK77	7.58E+06	6.40E+06	0.00E+00	0.00E+00	7.09E+06	1.08E+07	0.00E+00	9.93E+06	0.00E+00	0.00E+00	0.00E+00	2.00E+07	1.00E+07	0.00E+00	0.00E+00	9.30E+06	5.07E+06	5.23E+06	4.92E+06	6.00E+06	4.56E+06	7.50E+06	9.21E-01
Q9P0L0;Q9P0L0-2	1.95E+07	1.66E+07	3.11E+07	2.74E+07	2.13E+07	6.19E+07	1.34E+08	7.82E+07	0.00E+00	1.25E+07	8.03E+06	4.69E+07	3.51E+07	2.24E+07	1.81E+08	1.05E+08	5.00E+07	4.87E+07	5.13E+07	5.06E+07	4.08E+07	6.18E+07	9.22E-01
P06748-2;P06748;P06748-3	0.00E+00	7.78E+06	0.00E+00	0.00E+00	0.00E+00	0.00E+00	0.00E+00	0.00E+00	0.00E+00	0.00E+00	0.00E+00	0.00E+00	8.96E+06	0.00E+00	0.00E+00	0.00E+00	1.05E+06	9.72E+05	1.12E+06	2.87E+06	2.75E+06	3.17E+06	9.22E-01

Q92688-2;Q92688;Q5T6W8	0.00E+00	0.00E+00	0.00E+00	0.00E+00	0.00E+00	0.00E+00	1.37E+07	1.67E+07	0.00E+00	0.00E+00	0.00E+00	0.00E+00	1.88E+07	0.00E+00	0.00E+00	1.46E+07	3.98E+06	3.80E+06	4.17E+06	7.20E+06	7.08E+06	7.80E+06	9.22E-01
Q04760-2;Q04760	0.00E+00	0.00E+00	0.00E+00	1.10E+07	1.89E+07	1.29E+07	9.65E+06	1.13E+07	0.00E+00	0.00E+00	0.00E+00	1.66E+07	1.70E+07	0.00E+00	1.43E+07	1.28E+07	7.78E+06	7.96E+06	7.59E+06	7.44E+06	7.14E+06	8.22E+06	9.24E-01
Q15366-6;Q15366-3;Q15366;Q15366-2;F8VXH9;Q15366-7;F8VZX2;Q15366-8;Q15366-4;Q15366-5;F8W0G4;H3BRU6	3.83E+06	0.00E+00	0.00E+00	0.00E+00	0.00E+00	0.00E+00	0.00E+00	3.73E+06	0.00E+00	0.00E+00	0.00E+00	4.46E+06	0.00E+00	0.00E+00	0.00E+00	3.81E+06	9.90E+05	9.46E+05	1.03E+06	1.78E+06	1.75E+06	1.92E+06	9.25E-01
Q4TT38;Q13084;A2IDC6	0.00E+00	4.94E+06	4.76E+06	0.00E+00	0.00E+00	0.00E+00	0.00E+00	0.00E+00	0.00E+00	0.00E+00	0.00E+00	0.00E+00	0.00E+00	0.00E+00	5.06E+06	5.51E+06	1.27E+06	1.21E+06	1.32E+06	2.27E+06	2.25E+06	2.45E+06	9.27E-01
P26639;P26639-2	0.00E+00	0.00E+00	0.00E+00	0.00E+00	0.00E+00	1.19E+07	9.83E+06	9.53E+06	0.00E+00	0.00E+00	0.00E+00	8.09E+06	7.67E+06	0.00E+00	9.69E+06	7.62E+06	4.02E+06	3.91E+06	4.13E+06	4.81E+06	5.44E+06	4.47E+06	9.29E-01
O75489	7.00E+07	3.85E+07	5.67E+07	2.88E+07	3.50E+07	1.99E+07	2.68E+07	1.18E+07	6.06E+07	4.40E+07	5.60E+07	8.52E+06	3.72E+06	5.68E+07	2.54E+07	2.51E+07	3.55E+07	3.59E+07	3.50E+07	2.02E+07	1.92E+07	2.24E+07	9.29E-01
Q92945;A0A087WTP3;M0R0I5	0.00E+00	6.49E+06	0.00E+00	0.00E+00	0.00E+00	0.00E+00	0.00E+00	0.00E+00	0.00E+00	0.00E+00	0.00E+00	0.00E+00	5.71E+06	0.00E+00	0.00E+00	0.00E+00	7.63E+05	8.11E+05	7.14E+05	2.09E+06	2.29E+06	2.02E+06	9.30E-01
B1AK87;P47756-2;P47756;B1AK88;B1AK85	0.00E+00	0.00E+00	0.00E+00	0.00E+00	0.00E+00	7.68E+06	1.40E+07	1.96E+07	0.00E+00	1.79E+07	0.00E+00	0.00E+00	0.00E+00	1.13E+07	0.00E+00	1.48E+07	5.33E+06	5.16E+06	5.51E+06	7.54E+06	7.80E+06	7.80E+06	9.30E-01
Q05639	0.00E+00	0.00E+00	0.00E+00	0.00E+00	0.00E+00	6.51E+06	0.00E+00	6.21E+06	0.00E+00	0.00E+00	0.00E+00	0.00E+00	0.00E+00	7.12E+06	0.00E+00	4.60E+06	1.53E+06	1.59E+06	1.46E+06	2.77E+06	2.95E+06	2.79E+06	9.32E-01
Q9H3N1;G3V448	0.00E+00	0.00E+00	0.00E+00	4.97E+07	8.66E+07	5.38E+07	0.00E+00	0.00E+00	3.29E+07	0.00E+00	0.00E+00	6.26E+07	3.57E+07	6.98E+07	0.00E+00	0.00E+00	2.44E+07	2.38E+07	2.51E+07	3.10E+07	3.45E+07	2.95E+07	9.33E-01
P16435;E7EMD0;H0Y4R2	0.00E+00	4.79E+06	8.25E+06	0.00E+00	0.00E+00	4.91E+06	0.00E+00	4.55E+06	0.00E+00	0.00E+00	0.00E+00	4.94E+06	5.77E+06	0.00E+00	4.42E+06	6.37E+06	2.75E+06	2.81E+06	2.69E+06	2.97E+06	3.22E+06	2.93E+06	9.36E-01
Q99436	0.00E+00	2.38E+07	3.95E+07	1.90E+07	0.00E+00	3.04E+07	1.53E+07	2.49E+07	0.00E+00	1.61E+07	0.00E+00	5.12E+07	3.68E+07	0.00E+00	2.37E+07	1.98E+07	1.88E+07	1.91E+07	1.84E+07	1.59E+07	1.39E+07	1.88E+07	9.36E-01
Q15907;P62491-2;H3BMH2;Q15907-2;H3BSC1;P62491	0.00E+00	0.00E+00	0.00E+00	0.00E+00	0.00E+00	0.00E+00	6.07E+06	0.00E+00	0.00E+00	0.00E+00	0.00E+00	0.00E+00	0.00E+00	0.00E+00	6.80E+06	0.00E+00	8.05E+05	7.59E+05	8.50E+05	2.20E+06	2.15E+06	2.40E+06	9.38E-01
H0YH88;H0YHC3;F8W020;F8W118;F8VXI6;F8VUX1;F8VY35;P55209-3;A0A0A6YYI0;F8VV59;F8W543;B7Z9C2;P55209-2;F8W0J6;F5H4R6;H0YIV4;P55209	0.00E+00	8.73E+06	0.00E+00	0.00E+00	5.53E+06	6.56E+06	6.95E+06	1.10E+07	5.18E+06	5.44E+06	0.00E+00	6.38E+06	5.20E+06	1.05E+07	0.00E+00	7.24E+06	4.92E+06	4.85E+06	4.99E+06	3.82E+06	4.33E+06	3.53E+06	9.43E-01
P05455;E7ERC4;E9PGX9;E9PFL9	0.00E+00	0.00E+00	0.00E+00	0.00E+00	0.00E+00	0.00E+00	5.29E+06	5.82E+06	0.00E+00	0.00E+00	0.00E+00	0.00E+00	0.00E+00	0.00E+00	4.37E+06	7.50E+06	1.44E+06	1.39E+06	1.48E+06	2.63E+06	2.57E+06	2.87E+06	9.46E-01
Q7L014	1.01E+07	0.00E+00	0.00E+00	0.00E+00	0.00E+00	0.00E+00	0.00E+00	0.00E+00	0.00E+00	1.11E+07	0.00E+00	0.00E+00	0.00E+00	0.00E+00	0.00E+00	0.00E+00	1.33E+06	1.26E+06	1.39E+06	3.63E+06	3.57E+06	3.93E+06	9.47E-01
Q9Y305-2;Q9Y305;Q9Y305-4;Q9Y305-3	0.00E+00	0.00E+00	0.00E+00	0.00E+00	0.00E+00	0.00E+00	4.24E+06	0.00E+00	0.00E+00	0.00E+00	0.00E+00	0.00E+00	3.89E+06	0.00E+00	0.00E+00	0.00E+00	5.09E+05	5.31E+05	4.86E+05	1.39E+06	1.50E+06	1.38E+06	9.52E-01
O00203-3;O00203	1.04E+07	1.54E+07	1.38E+07	1.47E+07	0.00E+00	0.00E+00	0.00E+00	0.00E+00	0.00E+00	2.67E+07	2.59E+07	0.00E+00	0.00E+00	0.00E+00	0.00E+00	0.00E+00	6.68E+06	6.79E+06	6.58E+06	9.74E+06	7.41E+06	1.22E+07	9.66E-01
P31948;P31948-2;P31948-3;F5H783	0.00E+00	2.05E+07	0.00E+00	0.00E+00	0.00E+00	1.94E+07	4.53E+07	3.28E+07	0.00E+00	0.00E+00	0.00E+00	0.00E+00	2.55E+07	2.41E+07	3.90E+07	3.24E+07	1.49E+07	1.48E+07	1.51E+07	1.66E+07	1.77E+07	1.68E+07	9.67E-01
D6RAN4;P32969;H0Y9V9;E7ESE0	5.97E+06	0.00E+00	0.00E+00	0.00E+00	0.00E+00	0.00E+00	0.00E+00	0.00E+00	0.00E+00	6.33E+06	0.00E+00	0.00E+00	0.00E+00	0.00E+00	0.00E+00	0.00E+00	7.69E+05	7.46E+05	7.92E+05	2.10E+06	2.11E+06	2.24E+06	9.67E-01
P11166;C9JIM8	3.82E+07	2.99E+07	4.67E+07	3.04E+07	2.45E+07	6.31E+07	2.89E+07	4.20E+07	4.69E+07	2.98E+07	3.08E+07	7.88E+06	3.09E+07	0.00E+00	4.40E+07	3.96E+07	3.78E+07	3.80E+07	3.76E+07	1.73E+07	1.26E+07	2.20E+07	9.69E-01
Q13162;H7C3T4	0.00E+00	0.00E+00	7.88E+06	0.00E+00	1.24E+07	0.00E+00	9.59E+06	0.00E+00	0.00E+00	0.00E+00	5.74E+06	0.00E+00	5.86E+06	0.00E+00	1.07E+07	8.38E+06	3.78E+06	3.74E+06	3.83E+06	4.69E+06	5.30E+06	4.37E+06	9.70E-01
Q99832-3;Q99832;Q99832-4;Q99832-2	0.00E+00	0.00E+00	0.00E+00	0.00E+00	0.00E+00	1.46E+07	0.00E+00	0.00E+00	0.00E+00	0.00E+00	0.00E+00	0.00E+00	0.00E+00	0.00E+00	1.38E+07	0.00E+00	1.78E+06	1.82E+06	1.73E+06	4.86E+06	5.16E+06	4.89E+06	9.70E-01
C9JH92;A6NP24;Q08257-3;Q08257	1.57E+07	0.00E+00	1.31E+07	2.29E+07	7.64E+06	1.44E+07	1.85E+07	2.16E+07	1.82E+07	1.23E+07	1.85E+07	1.29E+07	2.14E+07	1.40E+07	0.00E+00	1.55E+07	1.42E+07	1.42E+07	1.41E+07	6.80E+06	7.54E+06	6.50E+06	9.71E-01

P04844-2;P04844;Q5JYR7;Q5JYR4	2.92E+07	4.32E+07	3.44E+07	4.90E+07	6.94E+07	5.20E+07	0.00E+00	2.90E+07	2.97E+07	4.65E+07	4.47E+07	2.96E+07	4.77E+07	2.82E+07	4.23E+07	3.51E+07	3.81E+07	3.83E+07	3.80E+07	1.51E+07	2.05E+07	8.21E+06	9.71E-01
A0A087WTT1;P11940-2;P11940;E7EQV3;E7ERJ7;H0YAR2;Q9H361	1.13E+07	0.00E+00	0.00E+00	2.30E+07	1.18E+07	1.75E+07	1.47E+07	1.74E+07	8.23E+06	1.03E+07	1.92E+07	0.00E+00	2.21E+07	2.39E+07	0.00E+00	1.31E+07	1.20E+07	1.20E+07	1.21E+07	8.49E+06	8.25E+06	9.29E+06	9.71E-01
Q9H9B4;S4R2X2;D6RFI0;D6RDG7	4.58E+07	3.39E+07	5.06E+07	0.00E+00	0.00E+00	6.42E+07	6.76E+07	6.97E+07	0.00E+00	2.01E+07	2.40E+07	7.83E+07	6.11E+07	0.00E+00	5.44E+07	8.97E+07	4.12E+07	4.15E+07	4.09E+07	3.06E+07	2.83E+07	3.47E+07	9.74E-01
P51149;C9J592;C9J8S3	0.00E+00	1.38E+07	0.00E+00	0.00E+00	0.00E+00	1.11E+07	1.33E+07	1.25E+07	0.00E+00	0.00E+00	0.00E+00	0.00E+00	1.88E+07	0.00E+00	1.64E+07	1.45E+07	6.27E+06	6.33E+06	6.21E+06	7.52E+06	6.81E+06	8.65E+06	9.75E-01
J3KTF8;P52565;J3QQX2;J3KRE2	1.46E+07	0.00E+00	0.00E+00	1.13E+07	1.03E+07	1.06E+07	0.00E+00	1.26E+07	0.00E+00	1.28E+07	0.00E+00	1.77E+07	0.00E+00	2.76E+07	0.00E+00	0.00E+00	7.34E+06	7.41E+06	7.27E+06	8.54E+06	6.28E+06	1.08E+07	9.75E-01
P48735;P48735-2;H0YL11	0.00E+00	0.00E+00	0.00E+00	9.90E+06	1.08E+07	8.95E+06	0.00E+00	0.00E+00	0.00E+00	9.21E+06	9.02E+06	0.00E+00	0.00E+00	6.16E+06	0.00E+00	5.86E+06	3.74E+06	3.71E+06	3.78E+06	4.54E+06	5.14E+06	4.21E+06	9.75E-01
Q9UJS0;Q9UJS0-2	8.47E+06	1.35E+07	1.33E+07	6.14E+06	6.14E+06	0.00E+00	7.92E+06	9.01E+06	1.33E+07	0.00E+00	0.00E+00	1.21E+07	9.72E+06	1.05E+07	8.18E+06	1.01E+07	8.02E+06	8.05E+06	7.98E+06	4.59E+06	4.31E+06	5.16E+06	9.75E-01
Q8IZL8;C9JFV4;E7EV54;I3L445	1.03E+07	0.00E+00	0.00E+00	0.00E+00	0.00E+00	1.05E+07	1.84E+07	8.13E+06	1.81E+07	0.00E+00	0.00E+00	1.03E+07	0.00E+00	0.00E+00	1.02E+07	7.97E+06	5.87E+06	5.92E+06	5.82E+06	6.70E+06	6.99E+06	6.87E+06	9.77E-01
Q3ZCQ8;Q3ZCQ8-2;M0R0C3;M0R2F8;Q3ZCQ8-3	0.00E+00	0.00E+00	0.00E+00	0.00E+00	0.00E+00	9.09E+05	0.00E+00	0.00E+00	0.00E+00	0.00E+00	0.00E+00	9.45E+05	0.00E+00	0.00E+00	0.00E+00	0.00E+00	1.16E+05	1.14E+05	1.18E+05	3.17E+05	3.21E+05	3.34E+05	9.78E-01
Q16850;C9IYR8;H7C0D0;Q16850-2	0.00E+00	1.43E+07	0.00E+00	1.11E+07	1.89E+07	0.00E+00	1.60E+07	1.96E+07	1.05E+07	1.34E+07	9.66E+06	0.00E+00	1.14E+07	1.08E+07	1.20E+07	1.14E+07	9.96E+06	1.00E+07	9.91E+06	6.58E+06	8.69E+06	4.16E+06	9.79E-01
E9PLK3;P55786;P55786-2	0.00E+00	4.14E+07	0.00E+00	0.00E+00	0.00E+00	2.32E+07	5.59E+07	4.48E+07	0.00E+00	0.00E+00	0.00E+00	3.31E+07	3.41E+07	0.00E+00	4.33E+07	5.73E+07	2.08E+07	2.07E+07	2.10E+07	2.29E+07	2.38E+07	2.36E+07	9.79E-01
P09211;A8MX94	0.00E+00	0.00E+00	0.00E+00	0.00E+00	0.00E+00	8.23E+06	0.00E+00	0.00E+00	0.00E+00	0.00E+00	0.00E+00	0.00E+00	0.00E+00	0.00E+00	0.00E+00	7.95E+06	1.01E+06	1.03E+06	9.93E+05	2.76E+06	2.91E+06	2.81E+06	9.80E-01
Q6PI48	2.30E+07	2.91E+07	3.03E+07	0.00E+00	1.82E+07	0.00E+00	4.21E+07	3.91E+07	2.93E+07	3.93E+07	2.44E+07	0.00E+00	2.09E+07	1.56E+07	2.51E+07	2.58E+07	2.26E+07	2.27E+07	2.26E+07	1.34E+07	1.60E+07	1.14E+07	9.80E-01
Q9P2J5;Q9P2J5-2	0.00E+00	9.27E+06	0.00E+00	0.00E+00	0.00E+00	1.50E+07	4.16E+07	3.39E+07	0.00E+00	0.00E+00	0.00E+00	1.86E+07	2.56E+07	0.00E+00	2.37E+07	3.32E+07	1.26E+07	1.25E+07	1.26E+07	1.49E+07	1.67E+07	1.41E+07	9.82E-01
X6RJP6;P37802;P37802-2	0.00E+00	0.00E+00	0.00E+00	0.00E+00	0.00E+00	0.00E+00	0.00E+00	6.59E+06	0.00E+00	6.41E+06	0.00E+00	0.00E+00	0.00E+00	0.00E+00	0.00E+00	0.00E+00	8.12E+05	8.24E+05	8.01E+05	2.22E+06	2.33E+06	2.26E+06	9.84E-01
O00154-2;K7EKP8;O00154-3;O00154-6;O00154-4;O00154-5;O00154-7;O00154	8.48E+07	6.13E+07	8.14E+07	0.00E+00	0.00E+00	9.53E+07	1.09E+08	9.80E+07	0.00E+00	3.05E+07	0.00E+00	1.66E+08	9.92E+07	3.70E+07	8.16E+07	1.19E+08	6.65E+07	6.62E+07	6.67E+07	5.04E+07	4.32E+07	5.99E+07	9.84E-01
P48047;H7C0C1	0.00E+00	0.00E+00	0.00E+00	0.00E+00	0.00E+00	2.32E+06	0.00E+00	0.00E+00	0.00E+00	0.00E+00	0.00E+00	0.00E+00	0.00E+00	2.27E+06	0.00E+00	0.00E+00	2.87E+05	2.90E+05	2.84E+05	7.84E+05	8.20E+05	8.02E+05	9.88E-01
Q9H9J2	2.09E+07	6.61E+07	5.59E+07	2.54E+07	5.36E+07	0.00E+00	7.96E+07	6.88E+07	4.98E+07	4.40E+07	4.13E+07	0.00E+00	6.45E+07	5.52E+07	5.38E+07	6.09E+07	4.62E+07	4.63E+07	4.62E+07	2.34E+07	2.77E+07	2.02E+07	9.92E-01
P07814;V9GYZ6	0.00E+00	3.46E+07	2.22E+07	0.00E+00	0.00E+00	5.57E+07	6.72E+07	5.50E+07	0.00E+00	0.00E+00	0.00E+00	5.12E+07	7.44E+07	0.00E+00	5.38E+07	5.64E+07	2.94E+07	2.93E+07	2.95E+07	2.91E+07	2.79E+07	3.23E+07	9.92E-01
